# Modifications of the Triaminoaryl Metabophore of Flupirtine and Retigabine Aimed at Avoiding Quinone Diimine Formation

**DOI:** 10.1021/acsomega.1c07103

**Published:** 2022-02-25

**Authors:** Konrad W. Wurm, Frieda-Marie Bartz, Lukas Schulig, Anja Bodtke, Patrick J. Bednarski, Andreas Link

**Affiliations:** Institute of Pharmacy, University of Greifswald, Friedrich-Ludwig-Jahn-Str. 17, 17489 Greifswald, Germany

## Abstract

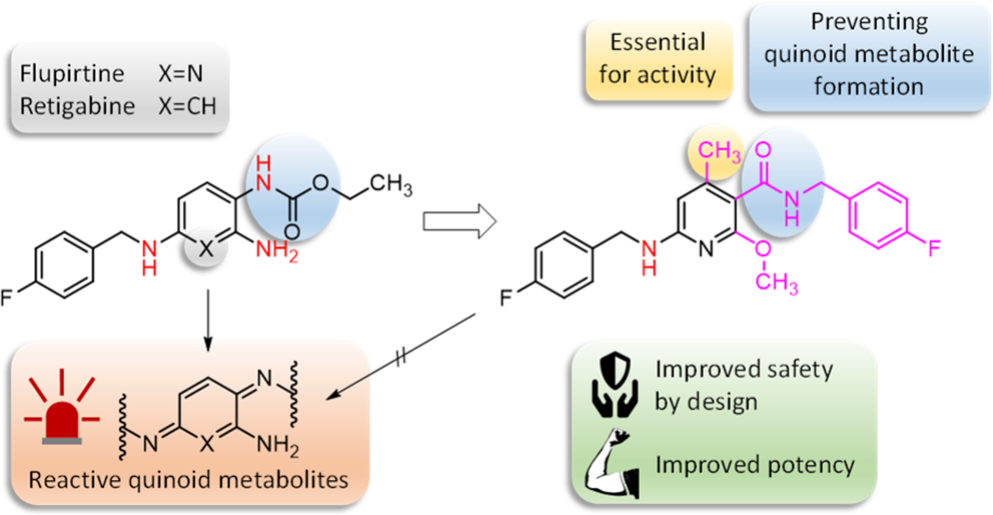

The potassium channel opening drugs flupirtine and retigabine have been withdrawn from the market due to occasional drug-induced liver injury (DILI) and tissue discoloration, respectively. While the mechanism underlying DILI after prolonged flupirtine use is not entirely understood, evidence indicates that both drugs are metabolized in an initial step to reactive *ortho*- and/or *para*-azaquinone diimines or *ortho*- and/or *para*-quinone diimines, respectively. Aiming to develop safer alternatives for the treatment of pain and epilepsy, we have attempted to separate activity from toxicity by employing a drug design strategy of avoiding the detrimental oxidation of the central aromatic ring by shifting oxidation toward the formation of benign metabolites. In the present investigation, an alternative retrometabolic design strategy was followed. The nitrogen atom, which could be involved in the formation of both *ortho*- or *para*-quinone diimines of the lead structures, was shifted away from the central ring, yielding a substitution pattern with nitrogen substituents in the meta position only. Evaluation of K_V_7.2/3 opening activity of the 11 new specially designed derivatives revealed surprisingly steep structure–activity relationship data with inactive compounds and an activity cliff that led to the identification of an apparent “magic methyl” effect in the case of *N*-(4-fluorobenzyl)-6-[(4-fluorobenzyl)amino]-2-methoxy-4-methylnicotinamide. This flupirtine analogue showed potent K_V_7.2/3 opening activity, being six times as active as flupirtine itself, and by design is devoid of the potential for azaquinone diimine formation.

## Introduction

**Figure 1:**
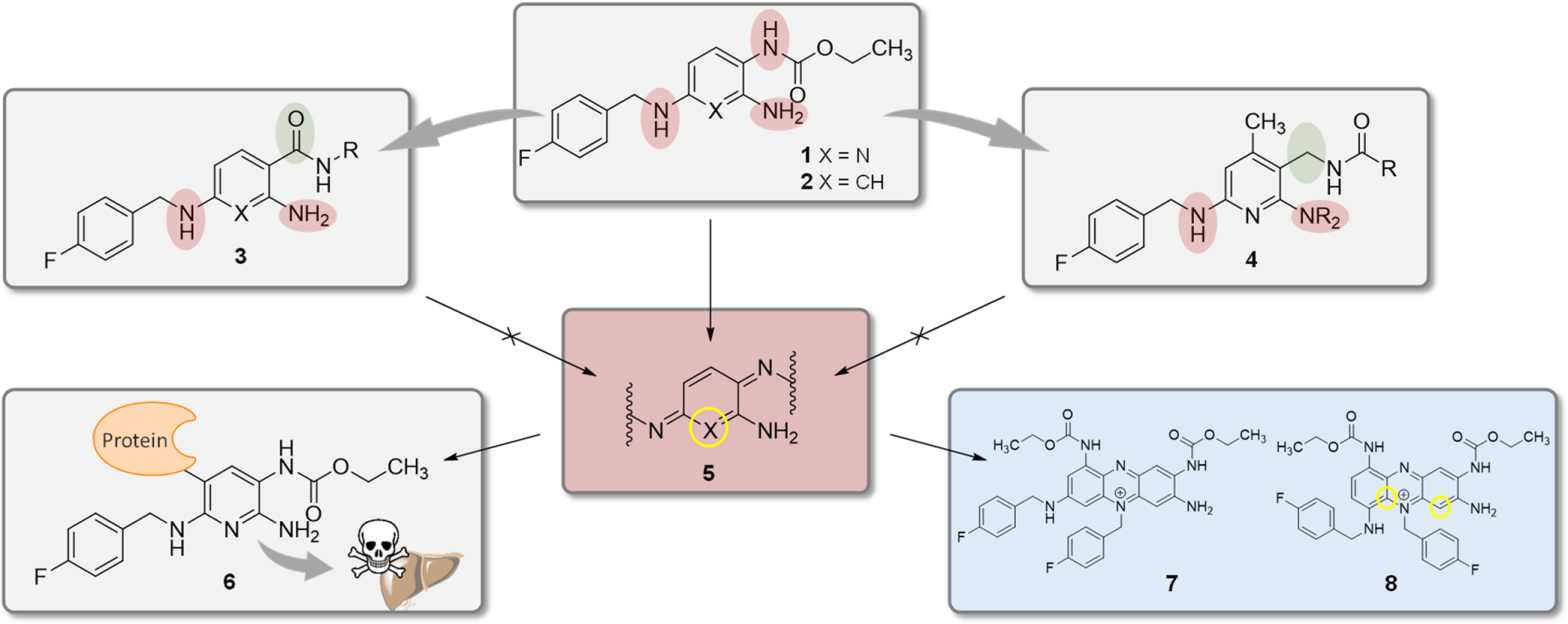
Structures of flupirtine (**1**) and retigabine (**2**), their proposed toxification pathway to phenazinium salts (**7**, **8**), and hapten-protein adducts **6** via quinone diimines **5**, and selected structural modifications carried out in this work (type **3** and **4**).

Reactive drug metabolites are well-known causes of drug-induced liver injury (DILI) and other adverse drug reactions and are thus an important determinant of drug toxicity and failure.^1,2^ Of 68 drugs that were retroactively recalled due to idiosyncratic toxicity or addressed in boxed warnings by the Food and Drug Administration, an association with the formation of reactive metabolites was found in 62–69% of the cases.^3^ Two current examples that reflect this problem are the K_V_7 channel openers flupirtine (**1**) and retigabine (**2**, USAN: ezogabine). The structural closely related compounds with a triaminoaryl scaffold are oxidized to reactive quinone diimine or azaquinone diimine metabolites (**5**) under different conditions and with different consequences (Figure 1).

In the case of flupirtine, oxidation is mainly catalyzed enzymatically and leads to rare but severe hepatotoxic reactions.^4−6^ The pathomechanism underlying the toxicity of flupirtine has not been fully clarified. An in vitro assay showed the formation of glutathione conjugates after enzymatic oxidation by peroxidases,^5^ and in a clinical study, mercapturic acid derivatives were detected in the urine of healthy human subjects treated with flupirtine,^7^ both of which are indicators for the formation of reactive azaquinone diimine metabolites (**5**). After covalent binding to endogenous macromolecules, reactive metabolites such as azaquinone diimines can either directly cause cellular damage or form haptens (**6**) that are able to trigger toxic autoimmune responses.^8^ The rarity of the hepatotoxic reactions under flupirtine treatment as well as the lack of a clear dose dependency rather suggests the hapten hypothesis and the involvement of the adaptive immune system. This assumption is backed by histological findings and the identification of a certain human leukocyte antigen gene as a genetic risk factor for flupirtine-induced hepatotoxicity.^9,10^ The hypothesis that pharmacogenetic causes, such as polymorphisms of metabolizing enzymes, contribute to flupirtine-induced hepatotoxicity could not be confirmed in a clinical study.^7^

In contrast, the oxidation of retigabine does not take place hepatically but probably in a reaction catalyzed by or in association with melanin.^11,12^ Via quinone diimines as intermediates, retigabine can dimerize to phenazinium structures (**7**, **8**), which are suspected of causing blue discoloration of various tissues.^12,13^ For these retigabine dimers, Groseclose et al. postulate compound **7** as a possible structure based on high-resolution and high-accuracy mass spectrometry data. In our opinion, however, isomer **8** is more likely to be formed because the formation of this structure is not associated with a carbamate migration to the free primary amino group. Flupirtine dimers are also known in the literature, but their structure is clearly different. Essentially, the structure of these dimers can be described as a flupirtine molecule linked to the pyridine ring of another flupirtine molecule via its amino group.^5^ Cyclization and oxidation to a phenazinium-like structure comparable to **8** were not observed. A hypothetical flupirtine analogue of dimer **8** would contain the two former pyridine nitrogen atoms at the positions highlighted with yellow circles in Figure 1, which would result in a highly unlikely direct connection of the positively charged phenazinium nitrogen atom with another positively charged nitrogen atom. Furthermore, in contrast to retigabine, there is no evidence that the reported dimers of flupirtine are responsible for the reported adverse drug reaction. In Summary, the blue tissue discoloration caused by retigabine as well as the hepatotoxicity of flupirtine finally led to the withdrawal of the respective drugs from the market, which is regrettable since their mechanism of action is unique, meaning that the proven therapeutical potential of K_V_7 channel openers is currently underexploited.^14,15^

K_V_7 channels (also referred to as KCNQ channels) are voltage-dependent homo- or heterotetrameric potassium channels distributed in both the central and peripheral nervous systems.^16−18^ Their activation is accompanied by a potassium efflux, as a result of which the membrane potential of neurons is hyperpolarized, leading to reduced transmission of action potentials.^19^ The important role of K_V_7 channels in controlling neuronal excitability makes them validated targets in the treatment of pain and epilepsy.^20^ As a consequence, the failure of the two existing K_V_7 channel openers flupirtine and retigabine, which were in therapeutic use in humans, left a treatment gap that has not yet been filled. Flupirtine was a valuable alternative to non-steroidal anti-inflammatory drugs and weak opioids because it caused neither gastrointestinal bleeding nor adverse effects such as addiction, constipation, or respiratory depression, notoriously associated with opioids.^21^ Retigabine has been shown to be effective for the adjunctive treatment of partial-onset seizures, and no adequate replacement for retigabine is available, especially in epilepsy forms where pathogenesis is based on a mutation of the K_V_7 channels.^22,23^ In addition to these approved indications, K_V_7 channel openers are supposed to have therapeutic potential in other medical areas, such as amyotrophic lateral sclerosis, traumatic brain injuries, or depression.^24−26^

**Figure 2:**
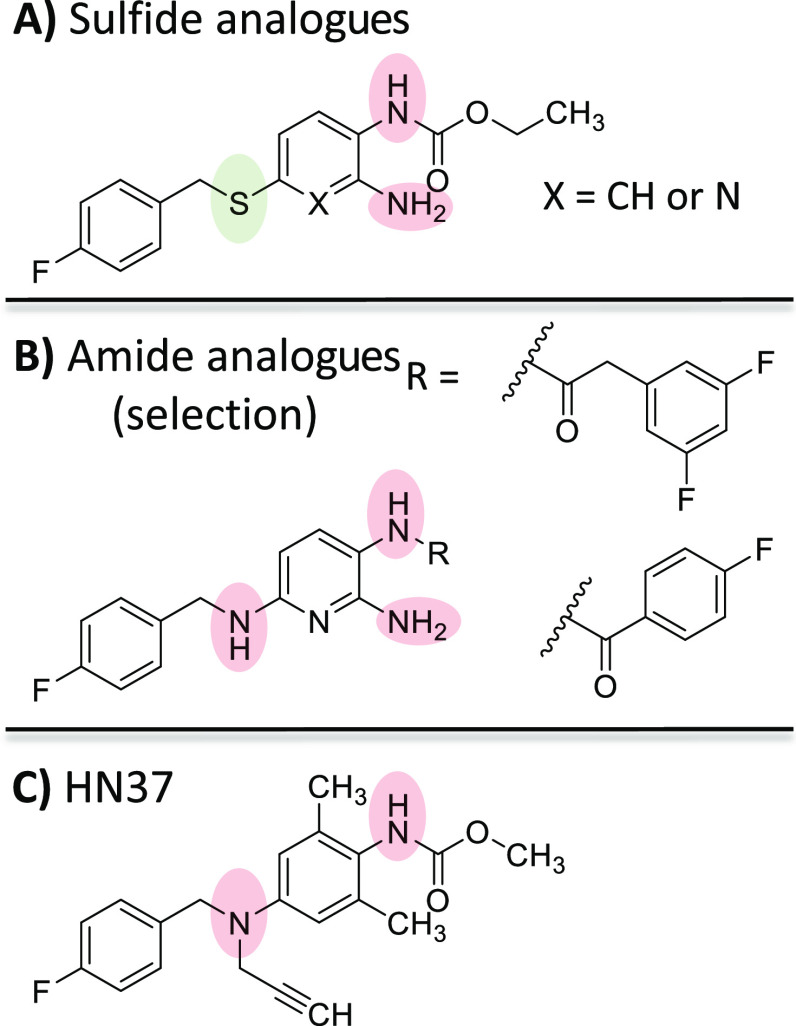
(A) Sulfide analogues of flupirtine (**1**) and retigabine (**2**) synthesized in a previous work.^27^ (B) Selection of amide analogues (not inverted) synthesized in a recent structure–activity relationship (SAR) study.^29^ (C) HN37, a drug candidate under clinical trial in China.^28^

Because the adverse drug reactions caused by flupirtine and retigabine are not related to the mechanism of action, it appeared promising to investigate chemical modifications of these clinically validated, drug-like leads in a retrometabolic drug design approach. In previous work, sulfide analogues (Figure 2A) were synthesized for this purpose in order to redirect the oxidation by shifting the highest occupied molecular orbital from the central aromatic ring to the sulfide substituent and thus avoiding the formation of quinone diimine metabolites.^27^ In the present study, we follow the alternative approach of changing the substitution pattern of the central aromatic ring in such a way that there are no longer two nitrogen atoms in the ortho or para position, removing the possibility for unwanted oxidative formation of quinone diimines. This is an advantage over the recently reported clinical candidate HN37 (Figure 2C), where the para-substitution pattern is identical to the lead structure **2**, and thus, the formation of charged *para*-quinone diimines is not fully excluded, per se.^28^ Since amide derivatives have proven to be very active in our previous work (Figure 2B), inverted amide derivatives were synthesized in which the carbonyl carbon atom is directly attached to an optionally methylated, central aromatic ring instead of the nitrogen atom (**3**, Figure 1).^27,29^ Another approach was the insertion of a methylene spacer to separate the carbamate/amide nitrogen atom from the central pyridine ring (**4**, Figure 1). A small set of substances was synthesized for both strategies in order to verify whether the applied retrometabolic drug design approach can lead to active K_V_7 channel openers.

## Results and Discussion

### Analogue design and synthesis

As mentioned above, in the case of the inverted amide scaffold (**3**), the formation of azaquinone diimines is not possible due to the meta position of the remaining amino substituents. In order to verify this hypothesis and exclude a risk for the formation of other quinoid metabolite species, the compounds were evaluated with XenoSite. XenoSite is a deep learning approach to predict the formation of quinone species in the drug metabolism, which considers both one- and two-step quinone formation and enables accurate quinone formation predictions in non-obvious cases. It is able to predict atom pairs that form quinones with an area under the curve (AUC) accuracy of 97.6% and identify molecules that form quinones with 88.2% AUC.^30^ A visualization of the results of compound **36b** as a representative example for this substance class is contained in Table 1, which underlines that in contrast to flupirtine and retigabine, no risk for the formation of quinoid metabolites is to be feared for the inverted amide scaffold.

**Table 1:**
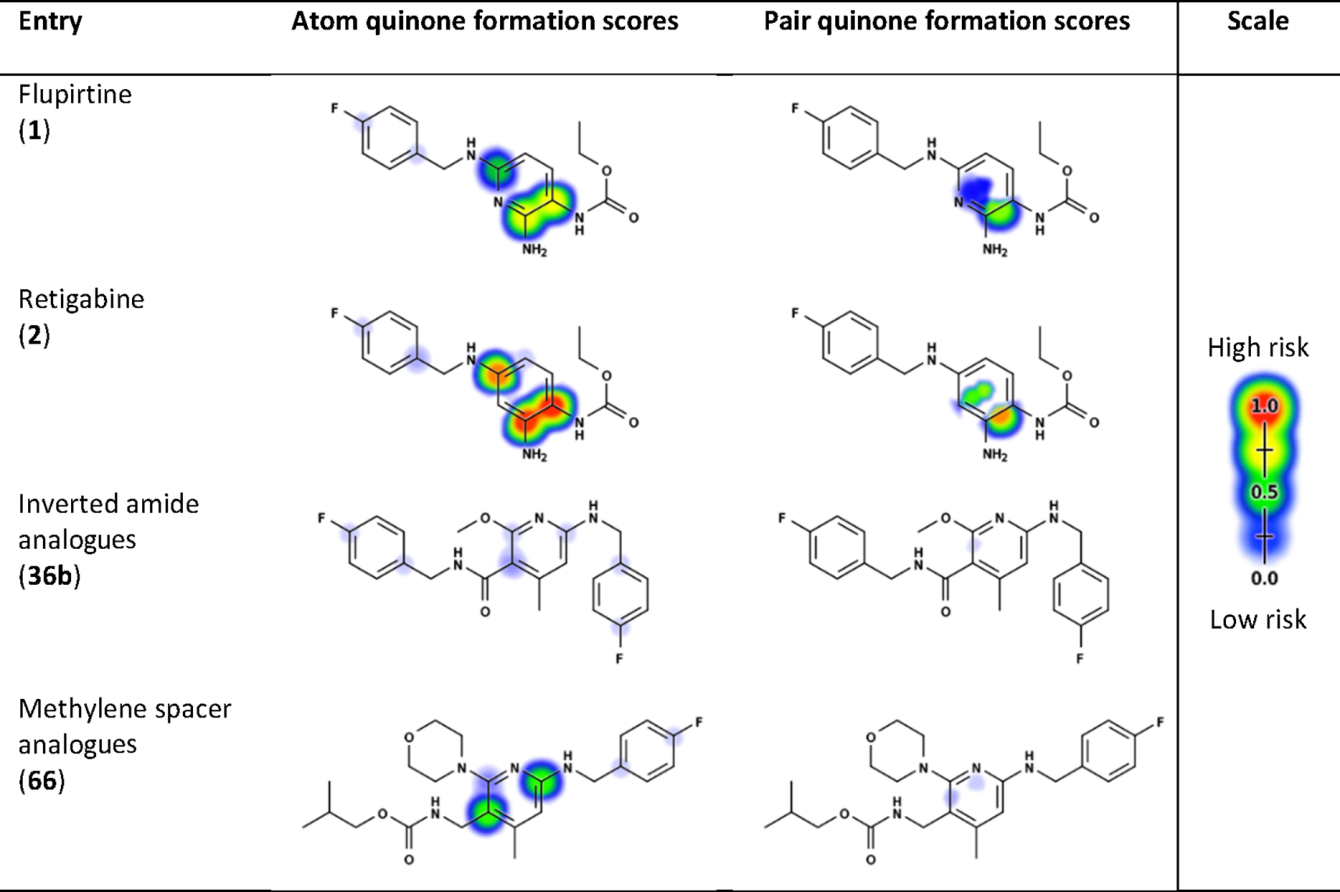
XenoSite-Predicted Probabilities for the Formation of Quinoid Metabolites of Flupirtine (**1**), Retigabine (**2**), a Representative Inverted Amide Analogue (**36b**), and a Representative Methylene Spacer Analogue (**66**)

When initially designing the inverted amide analogues, only the amide side chain was varied in order to keep as closely as possible to the structure of flupirtine and retigabine. For the design of the side chains, we partly used findings from an earlier SAR study that examined, inter alia, non-inverted amide derivatives of flupirtine and retigabine.^29^ Both aliphatic and aromatic side chains were evaluated in the present work. Mainly a fluorobenzyl substituent was used as an aromatic amide side chain since similar residues proved successful in the previous work mentioned above (Figure 2B). Aliphatic side chains, such as a butyramide residue, in particular, have also proven to be active in the case of the non-inverted amide derivatives. The *N*-propylamide and *N*-butylamide side chains used in the following are meant to be analogues of the butyramide side chain of the non-inverted amide derivatives and were of interest because they more closely resemble the ethyl carbamate structure of flupirtine and retigabine compared to the aromatic substituents. In addition, a branched 2-methylpropyl side chain was also used since findings from the non-inverted amide analogues suggest that bulkier substituents in the amide region lead to improved activity.

**Scheme 1:**
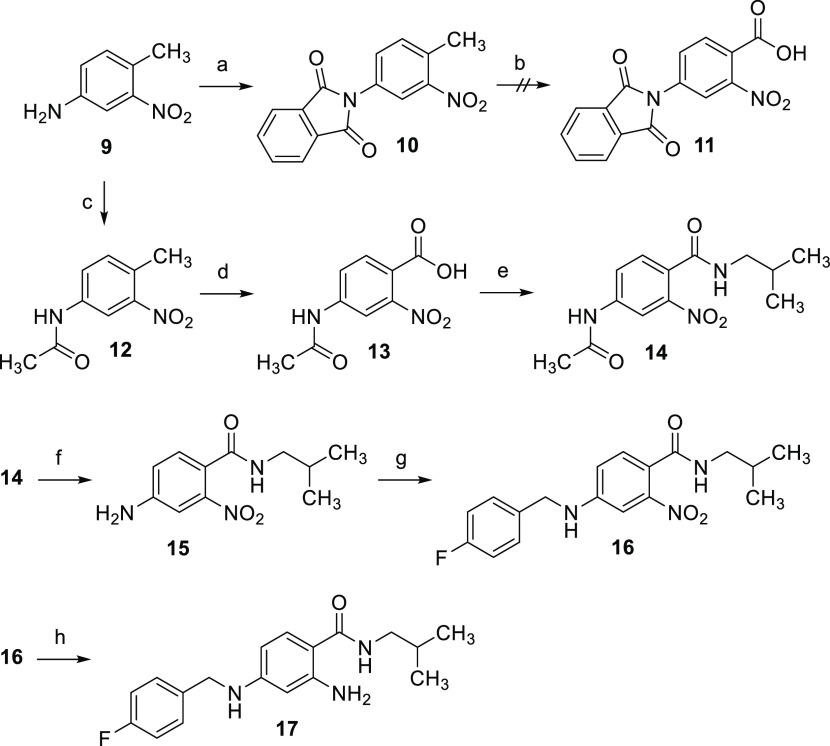
Synthesis of Compound **17** as a Representative of Target Modification Type **3** (a) Phthalic anhydride, TEA, toluene, 120 °C, 4 h, 82%; (b) KMnO_4_, H_2_O, 80 °C; (c) acetic anhydride, AcOH, RT, 15 min, 87%; (d) KMnO_4_, H_2_O, 80 °C, 5 h, 52%; (e) isobutylamine, CDI, THF, RT, 17 h, 74%; (f) HCl, H_2_O, 100 °C, 2 h, 96%, (g) (1) 4-fluorobenzaldehyde, toluene, 120 °C, 5 h (2) NaBH_4_, 1,4-dioxane, MeOH, RT, 1.5 h, 37%; (h) SnCl_2_, EtOAc, 80 °C, 30 min, 68%.

A retigabine derivative with a 2-methylpropyl amide side chain (**17**) was realized in a six-step synthesis (Scheme 1). The starting material was the commercially available compound **9**, whose amino group was protected in the first step. Both inclusion of the amino function into a phthalimido group and acetylation were evaluated as possible protecting strategies in **10** and **12**, respectively. However, only the acetamido group proved to be functional since the phthalimido group was not stable in the subsequent KMnO_4_ oxidation to carboxylic acid **13**. After the oxidation step, the amide coupling was carried out by using 1,1′-carbonyldiimidazole (CDI) as a coupling reagent. The acetylamino group of the resulting benzamide **14** was then deacetylated by acidic hydrolysis of the acetanilide function, yielding **15**. This was followed by reductive amination to obtain compound **16**, and in the end, a reduction of the nitro group with SnCl_2_ was carried out to give the target compound **17**.

**Scheme 2:**
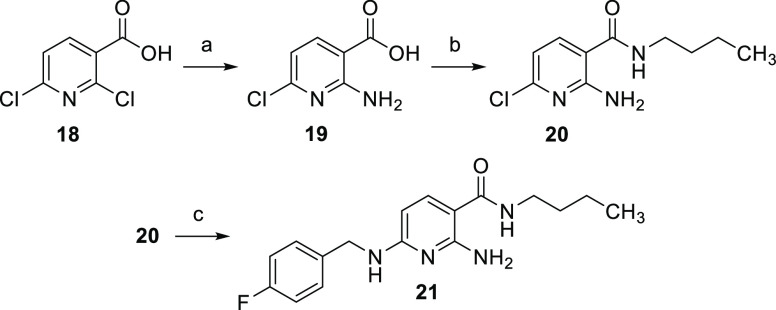
Synthesis of Compound **21** as a Representative of Target Modification Type **3** (a) NaN_3_, CuI, ethylenediamine, K_2_CO_3_, EtOH, 95 °C, 11.5 h, 87%; (b) *n*-butylamine, CDI, DCM, RT, 48 h, 55%; (c) 4-fluorobenzylamine, 160 °C, 3 h, 16%.

An inverted amide analogue of flupirtine was obtained in a shorter, three-step synthesis (Scheme 2). 2,6-Dichloronicotinic acid (**18**) was the starting material in this route. In the first step of the synthesis, the primary amino group was introduced regioselectively in position 2. This was achieved in a copper-catalyzed, Ullmann-type reaction with sodium azide as a nitrogen source. In accordance with the mechanism proposed in the literature, the reaction is assumed to proceed via a five-membered, cyclic transition state formed after oxidative addition, which involves the carboxyl group as well as the copper ion and explains the observed regioselectivity. An aryl azide, formed as an intermediate after reductive elimination, was reduced directly in situ to the corresponding primary amine **19**.^31^ It is also possible to introduce the amino group through a simple nucleophilic substitution with ammonia. However, this variant turned out to be inferior to the copper-catalyzed reaction since it was not as regioselective. This made a chromatographic separation of the regioisomers necessary and resulted in a significantly lower yield of only 57% compared to 87% for the copper-catalyzed reaction. In the next step, the amide coupling was carried out as described for the synthesis of retigabine derivative **17** with CDI as the coupling reagent, yielding **20**. The final nucleophilic substitution reaction to introduce the 4-fluorobenzylamino residue gave compound **21** but unfortunately in a poor yield of 16%; 4-fluorobenzylamine is only a moderate nucleophile and, moreover, presumably decomposes at the high temperature required for the reaction.

In addition to the synthesis of the two inverted amide derivatives with minimal structural changes in comparison to lead **1** and **2**, the next step was to introduce advantageous substructures inspired by derivatives of flupirtine and retigabine known from the literature. For example, the effect of an additional methyl group in the ortho position to the amide function was investigated, which is included in several potent K_V_7 channel openers (not shown).^28,32^ Furthermore, a replacement of the primary amino group by an alkoxy residue and the introduction of a fluorinated phenyl ring into the amide side chain proved to be advantageous for the K_V_7.2/3 channel opening activity in our previous work.^29^ Both structural changes were also applied to the inverted amide derivatives. However, the 4-fluorobenzylaminoaryl scaffold incorporated in flupirtine and retigabine was kept unchanged in all synthesized compounds.

**Scheme 3:**
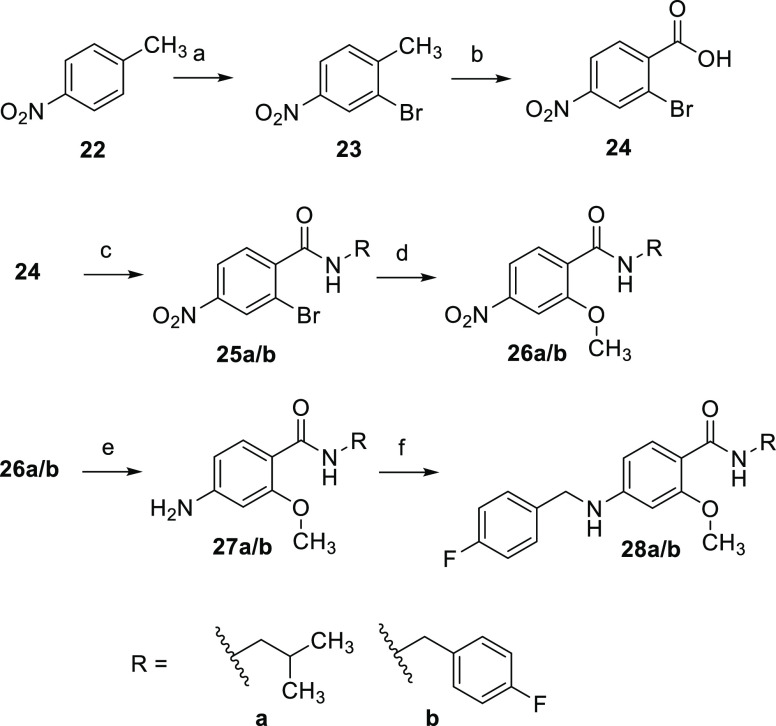
Synthesis of Compounds **28a** and **b** (a) Br_2_, Fe, 80 °C, 1.5 h, 55%; (b) KMnO_4_, H_2_O, pyridine, 100 °C, 17 h, 18%; (c) (1) SOCl_2_, toluene, 120 °C, 2.5–8 h, (2) isobutylamine or 4-fluorobenzylamine, (TEA), DCM, 0 °C to RT, 2.5–16 h, 42–72%; (d) MeOH, K_2_CO_3_, CuI, ethylenediamine, N_2_, 95 °C, 15 h, 46–51%; (e) SnCl_2_, EtOAc, 80 °C, 30 min, 98–100%; (f) (1) 4-fluorobenzaldehyde, toluene, 120 °C, 5–7 h, (2) NaBH_4_, 1,4-dioxane, MeOH, RT, 17–24 h, 41–69%.

As depicted in Scheme 3, the syntheses of two methoxy-substituted derivatives with an aliphatic or aromatic amide side chain were conducted starting from 4-nitrotoluene (**22**). After regioselective bromination, the methyl group of **23** was oxidized to a carboxyl group with KMnO_4_ as the oxidizing agent to obtain **24**. This was followed by the amide coupling in which the acyl chloride was formed in situ by reaction with thionyl chloride and subsequently reacted with the corresponding amines to give the desired amides **25a/b**. The methoxy function was introduced in a copper-catalyzed C–O cross-coupling reaction with methanol in a dual function as a solvent and reactant. Finally, the aromatic nitro compound **26a/b** was reduced to aniline **27a/b**, followed by reductive amination, which provided the target compounds **28a/b**.

**Scheme 4:**
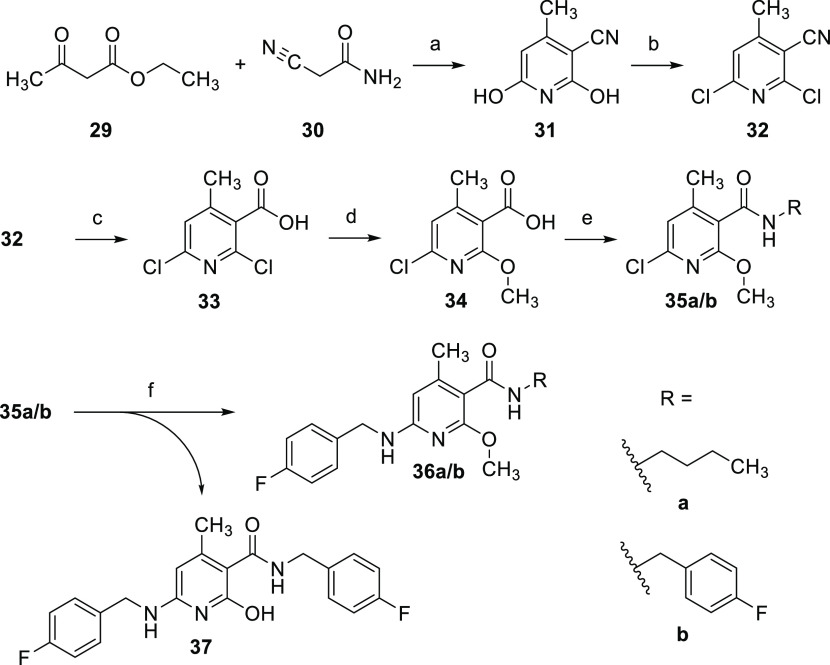
Synthesis of Compounds **36a** and **b** (a) KOH, MeOH, 65 °C, 2 h, 75%; (b) benzyltrimethylammonium chloride, POCl_3_, 90 °C, 16 h, 55%; (c) H_2_SO_4_, HNO_3_, 105 °C, 2 h, 77%; (d) MeOH, NaH, THF, 0–70 °C, 7 h, 96%; (e) (1) (COCl)_2_, DMF, THF, 0 °C to RT, 2–3 h, (2) *n*-butylamine or 4-fluorobenzylamine, (TEA), DCM or THF, 0 °C to RT, 16 h, 53–61%; (f) 4-fluorobenzylamine, 165 °C, microwave, 1 h, 7–23%.

An additional methyl group in the ortho position to the amide function could easily be introduced by the Guareschi reaction, in which acetoacetic ester reacts with cyanoacetamide in a condensation reaction to form a pyridine derivative. The resulting dihydroxypyridine **31** was subsequently chlorinated by treatment with phosphoryl chloride, yielding **32** (Scheme 4). Thereafter, the nitrile group was hydrolyzed to obtain carboxylic acid **33**. This worked best with a mixture of concentrated sulfuric acid and concentrated nitric acid. While such mixtures are known nitrating agents, nitration of the electron-poor pyridine ring was not to be expected here. The subsequent introduction of the methoxy group was achieved by nucleophilic substitution with sodium methoxide produced in situ by the reaction of sodium hydride with methanol. As described in the literature for similar cases, the substitution reaction proceeded regioselectively with the exclusive formation of product **34** substituted at position 2.^33^ Yap et al. suggest that this regioselectivity could be explained by the formation of a six-membered, cyclic transition state involving the carboxyl group and the sodium methoxide, which favors position 2.^33^ In the next step, the amide coupling was again carried out via an acyl chloride, which in contrast to the synthesis of **25a/b** was not generated with thionyl chloride but with oxalyl chloride. Finally, the 4-fluorobenzylamino residue was introduced as described for **21** by using microwave heating instead of conventional heating in order to obtain the final compounds **36a/b**. The low yields of 7–20% can be partly explained by ether cleavage, resulting in the formation of a byproduct identified as **37** in the case of the synthesis of **36b**.

**Scheme 5:**
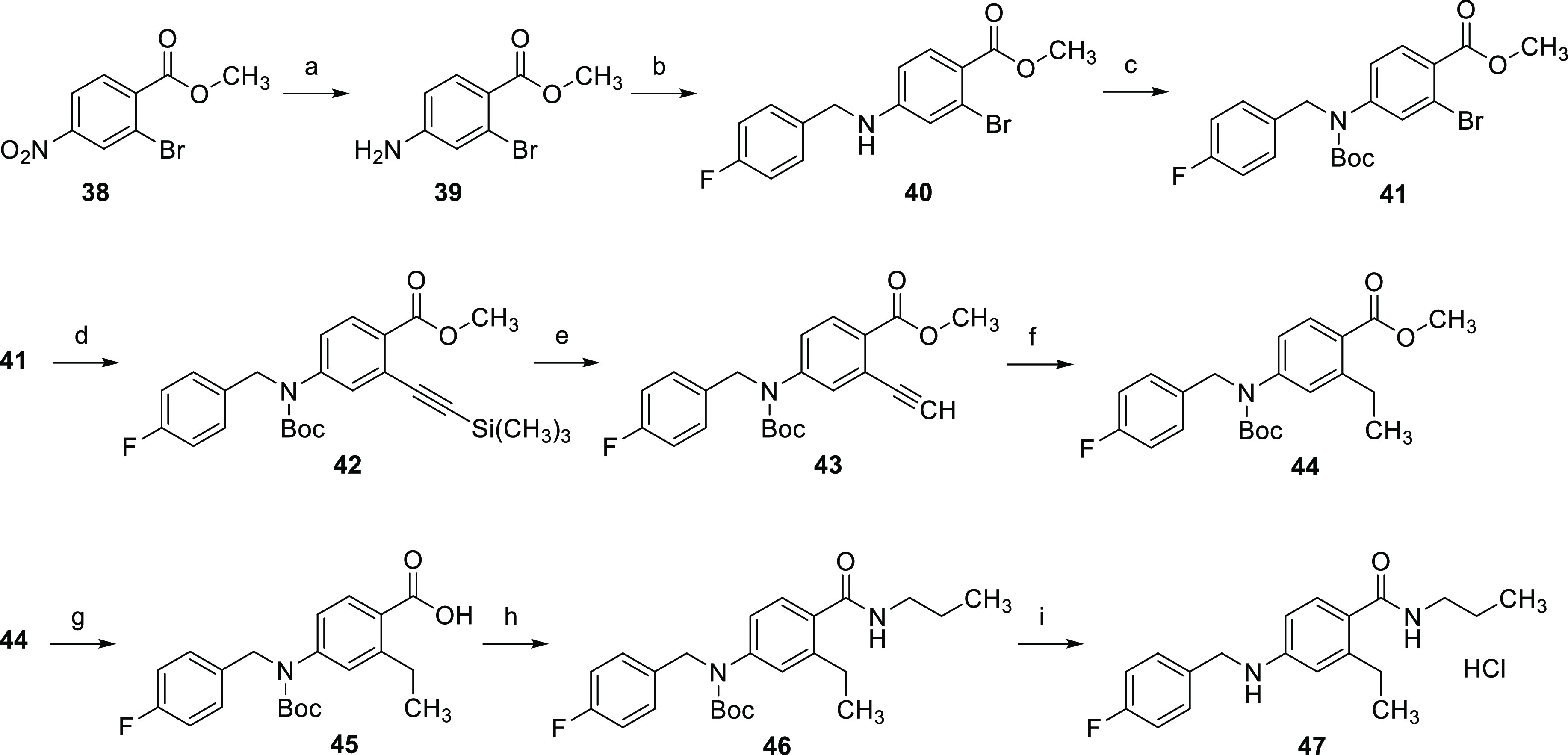
Synthesis of Compound **47** (a) SnCl_2_, EtOAc, 70 °C, 4 h, 91%; (b) (1) 4-fluorobenzaldehyde, toluene, 120 °C, 8 h, (2) NaBH_4_, 1,4-dioxane, MeOH, 0 °C to RT, 17 h, 87%; (c) Boc_2_O, TEA, 4-DMAP, DCM, RT, 16 h, 87%; (d) trimethylsilylacetylene, CuI, Pd(PPh_3_)_4_, TEA, DMF, 60 °C, 4 h, 76%; (e) K_2_CO_3_, MeOH, RT, 1 h, 84%; (f) H_2_, Pd/C, MeOH, 40 °C, 16 h, 86%; (g) KOH, MeOH, H_2_O, 40 °C 24 h, 91%; (h) *n*-propylamine, HATU, DIPEA, DMF, RT, 8 h, 78%; (i) (1) TFA, DCM, RT, 6 h, (2) HCl, EtOAc, 0 °C, 30 min, 90%.

An ethyl group was also evaluated as an alternative to the methoxy group; Scheme 5 shows the synthesis of a corresponding compound. The starting point was commercially available ester **38**. A reduction of the nitro group with SnCl_2_ and subsequent reductive amination of **39** with NaBH_4_ gave the 4-fluorobenzylamino-substituted compound **40**. After Boc protection of the amino group, the coupling of trimethylsilylacetylene was performed via a Sonogashira reaction to yield **42**. The trimethylsilyl protective group was then selectively cleaved with K_2_CO_3_ in order to subsequently reduce the resulting terminal alkyne by catalytic hydrogenation to obtain **44**. This was followed by hydrolysis of the methyl ester under alkaline conditions. The amide coupling, which gave **46**, was performed with a coupling reagent. Since the previously used methods for amide coupling via acyl chlorides or CDI resulted in yields that could be improved (42–72%), 1-[bis(dimethylamino)methylene]-1*H*-1,2,3-triazolo[4,5-*b*]pyridinium 3-oxide hexafluorophosphate (HATU) was evaluated here as an alternative coupling reagent, which enabled a good yield of 78%. The use of CDI instead of HATU would, however, probably also have been possible. In the last step, the Boc protective group was cleaved with trifluoroacetic acid (TFA), and final product **47** was isolated by precipitation of the hydrochloride salt.

**Figure 3:**
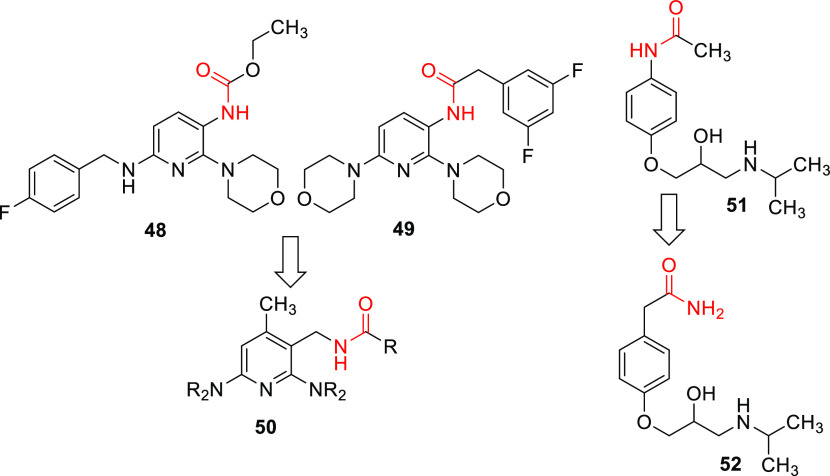
Design of flupirtine analogues containing a methylene spacer (**48**, **49**, **50**) and structural changes made in atenolol (**52**) compared to practolol (**51**).

As illustrated in Figure 3, the second retrometabolic drug design approach was the introduction of a methylene spacer between the carbamate or amide nitrogen atom and the central aromatic ring in **50**. The analogues with a methylene spacer here differed to a greater extent from flupirtine and retigabine in terms of their substituents since two morpholino-substituted flupirtine derivatives with nanomolar EC_50_ values (**48**, **49**) synthesized in a previous work served as templates (Figure 3).^29^ As already described for the inverted amides, an additional methyl group was introduced in position 4 of the pyridine ring. A similar retrometabolic drug design approach was used successfully in the development of atenolol (**52**) from practolol (**51**). In order to prevent the formation of a toxic *N*-hydroxyaniline metabolite after cleavage of the anilide structure, an analogously functioning methylene spacer was introduced in **52**.^34,35^ In contrast to the compounds synthesized here, however, the amide was additionally inverted. The introduction of a methylene spacer in **50** prevents the formation of azaquinone diimine metabolites due to the meta position of the remaining amino substituents. These compounds were also tested with XenoSite for the risk of quinoid metabolite formation.^30^ A visualization of the results of compound **66** as a representative example for this scaffold is contained in Table 1. The risk for the formation of quinoid metabolites is significantly reduced compared to flupirtine and retigabine but not completely excluded as with the inverted amide scaffold. The formation of an azaquinone imine methide metabolite is theoretically conceivable in this case, although not very likely, as suggested by the in silico prediction.

**Scheme 6:**
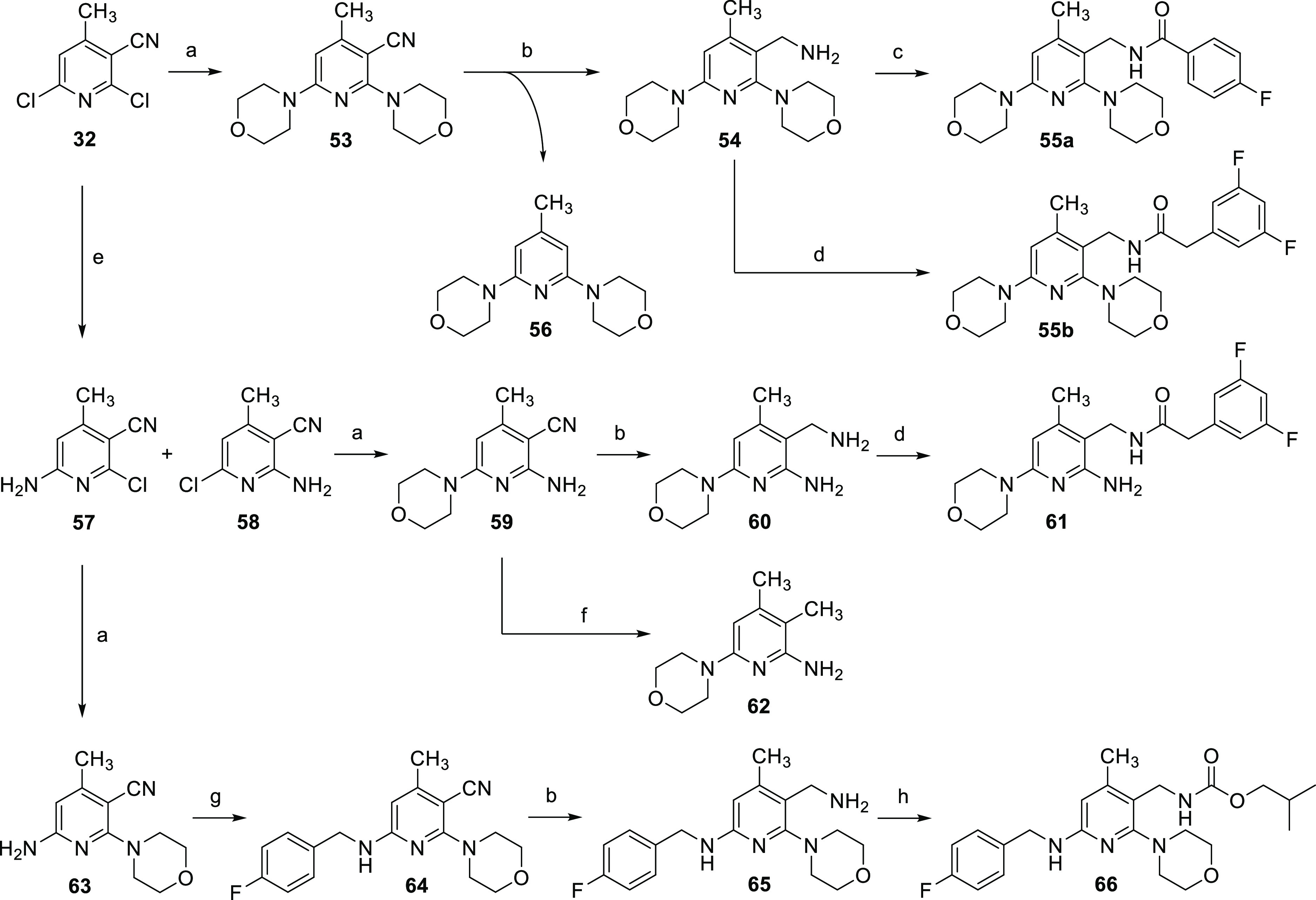
Synthesis of Compound **55a–66** as a Representative of Target Modification Type **4** (a) Morpholine, 2-propanol, 170 °C, pressure reactor, 3–5 h, 82–89%; (b) Ni, H_2_, NH_3_, MeOH, 50 °C, 5–6 h, 34–52%; (c) 4-fluorobenzoyl chloride, TEA, DCM, 0 °C to RT, 16 h, 79%; (d) 2-(3,5-difluorophenyl)acetic acid, CDI, THF, RT, 18 h, 69–84%; (e) NH_3_, 2-propanol, 70 °C, pressure reactor, 9 h, 14% (**57**), 20% (**58**); (f) H_2_, Pd/C, MeOH, 40 °C, 48 h, 79%.; (g) (1) 4-fluorobenzaldehyde, DCM, RT, 16 h, (2) MeOH, NaBH_3_CN, RT, 25 h, 42%; (h) isobutyl chloroformate, TEA, DCM, 0 °C to RT, 16 h, 64%.

As described in Scheme 6, the syntheses of the compounds with methylene spacers were carried out starting from nitrile **32**. Different amino substituents were introduced via nucleophilic substitution reactions. The introduction of the second amino substituent required high temperatures, which made the use of a pressure reactor necessary. The pressure reactor was also used to introduce the primary amino function of **57** and **58** by heating chloropyridine **32** in a saturated solution of ammonia in 2-propanol. The resulting mixture of isomers was then separated by chromatography. Since the regioisomers could not be clearly assigned on the basis of their ^1^H- and ^13^C NMR spectra, a heteronuclear multiple bond correlation (HMBC) spectrum was recorded in each case, which is contained in the Supporting Information. This enabled the isomers to be differentiated since in the case of isomer **57**, a coupling between the protons of the amino group and the CH carbon atom of the pyridine ring was visible, which was missing in the HMBC spectrum of isomer **58**. The 4-fluorobenzylamino-substituted intermediate **64** was obtained via reductive amination of **63**. After both amino substituents had been introduced, the nitrile group was reduced to a primary amino group in each case. The first attempt to carry out the reduction of **59** by means of catalytic hydrogenation with the use of Pd/C as a catalyst failed and instead gave compound **62**. As a consequence, Raney nickel was used as an alternative catalyst, and ammonia was added to the reaction mixture. With this reduction procedure, the desired primary amines **54**, **60**, and **65** could be obtained. However, byproducts were formed in all the reactions. Upon reduction of **53**, a specific byproduct was isolated and identified as compound **56**. In the last step, the primary amino group was acylated or carbamylated; this was done either by coupling the corresponding carboxylic acid with CDI as the coupling reagent (**55b/61**) or by reaction with 4-fluorobenzoyl chloride and isobutyl chloroformate, respectively (**55a/66**).

### Biological Evaluation

**Figure 4:**
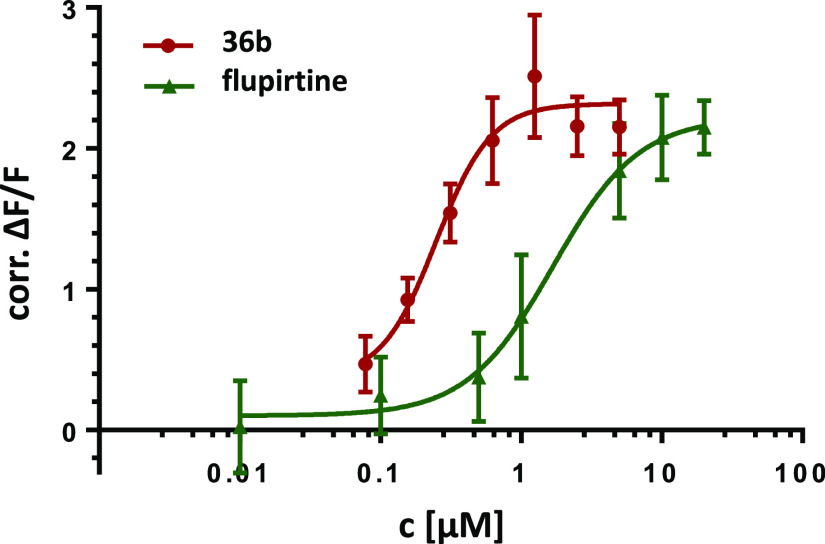
Comparison of the concentration–activity curves of **36b** and flupirtine (**1**).

To evaluate the biological activity, the synthesized analogues were tested on HEK293 cells overexpressing the K_V_7.2/3 channel. The measurement was carried out in a fluorescence-based assay in which a thallium-sensitive dye generated the fluorescence signal. Due to its similarity to potassium, thallium can also pass through K_V_7 channels. The intensity of the fluorescence signal correlates with the amount of intracellular thallium and thus with the ability of the compounds to open K_V_7 channels. To determine the potency, the fluorescence intensity was measured as a function of the compound concentration. The efficacy was determined relative to the maximum fluorescence signal induced by flupirtine. A representative concentration–activity curve is shown in Figure 4. For a summary of the activity data, see Table 2.

**Table 2:** K_V_7.2/3 Channel Opening Activity, In Vitro Toxicity, and log *D*_7.4_ Values of the Synthesized Compounds **17–66** in Comparison to Flupirtine (**1**) and Retigabine (**2**)^a^

		HEK-293 cells		TAMH cells		HEP-G2 cells	
entry	log *D*_7.4_	EC_50_^b^ [μM]	efficacy [%]	LD_50_^c^ [μM]	LD_25_^d^ [μM]	LD_50_^c^ [μM]	LD_25_^d^ [μM]
**1**	2.1	1.837 ± 0.844	100	487 ± 51	103 ± 47	547 ± 111	74 ± 40
**2**	2.1	0.147 ± 0.012^f^	134 ± 16^f^	>400^f^	>400^f^	>400^f^	269 ± 166^f^
**17**	2.7	e		>125	>125	>125	56 ± 40
**21**	2.9	e		149 ± 7	145 ± 7	149 ± 11	148 ± 11
**28a**	3.8	e		>63	43 ± 27	42 ± 7	31 ± 13
**28b**	4.3	e		>8	>8	>8	>8
**36a**	3.7	3.515 ± 0.724	65 ± 9	>63	17 ± 1	>63	24 ± 1
**36b**	4.1	0.310 ± 0,119	105 ± 12	>63	>63	>16	>16
**47**	3.2	e		>125	140 ± 14	77 ± 15	60 ± 9
**55a**	3.5	e		>31	22 ± 7	>31	22 ± 6
**55b**	3.5	e		>250	24 ± 5	>250	20 ± 7
**61**	2.6	e		>63	>63	>63	>63
**66**	4.8	e		>125	52 ± 61	>125	24 ± 27

a log *D*_7.4_ values were estimated by using an HPLC-based method. EC_50_ values were obtained in HEK293 cells overexpressing K_V_7.2/3 channels. LD values were determined by the MTT assay after 24 h exposure. EC_50_ and LD values are means and SDs of ≥3 independent determinations, respectively.

b Concentration required to reach half-maximal channel opening activity.

c Concentration required to reduce cell viability by 50% compared to untreated controls.

d Concentration required to reduce cell viability by 25% compared to untreated controls.

e Inactive up to a concentration of 10 μM.

f Retigabine (**2**) values were taken from our recently published work.^27^

The two inverted amide analogues **17** and **21** with structural changes affecting only the amide side chain were found to be inactive in the biological testing. Inactive in this context means that no channel opening activity was detected up to a concentration of 10 μM. Even the compounds in which the primary amino group was replaced with a methoxy function (**28a/b**) turned out to be inactive. This is noteworthy because previous work had shown alkoxy substitution to be very beneficial for activity.^29^ The ethyl substituent in place of the primary amino group in compound **47** also did not result in any improvement in terms of activity. In addition, the experimentally determined log *D*_7.4_ values of the three last-mentioned analogues (**28a/b**, **47**) are in the range of 3.2–4.3, that is, compared to flupirtine and retigabine, whose log *D*_7.4_ value is 2.1, the compounds are thus significantly more lipophilic. Increased lipophilicity, specifically indicated by log *D*_7.4_ values greater than 3.5, has proven advantageous in terms of activity in the past but was not confirmed in the present cases.^27^ This initially raised concerns that the change to the inverted amide scaffold is a too severe structural intervention to maintain K_V_7.2/3 channel opening activity.

Surprisingly, the two analogues with an additional methyl group in position 4 of the pyridine ring (**36a/b**) were found to be quite active, whereby **36b** with an EC_50_ value of 0.310 μM is even about six times more potent than flupirtine with similar efficacy. Figure 4 shows the direct comparison of the concentration–activity curves of **36b** and flupirtine, illustrating the improved activity of **36b**.

**Figure 5:**
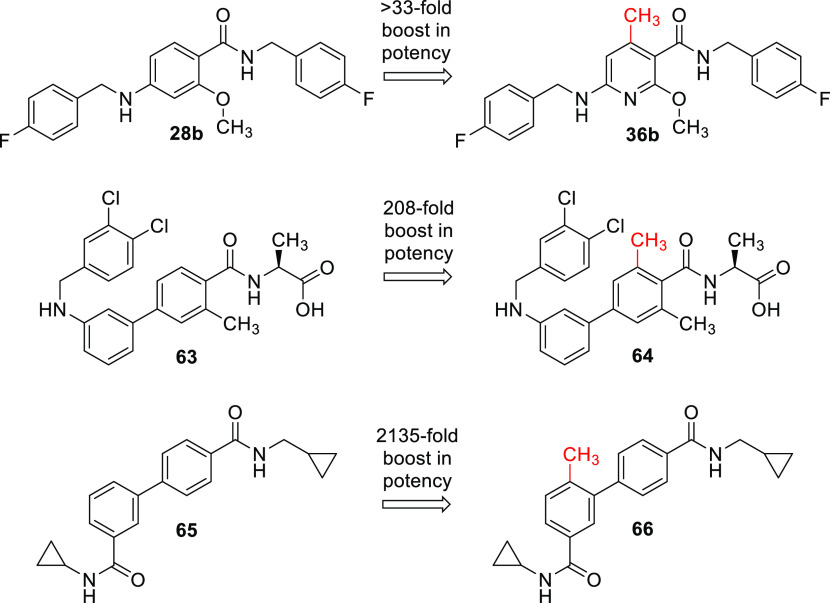
Drastically improved activity by additional *ortho* methyl groups in present example (**36b**) and literature cases of an S1P_1_ antagonist (**64**) and an inhibitor of the p38α MAP3 kinase (**66**).

Of particular interest is the comparison between compound **36b** with nanomolar potency and compound **28b**, which is inactive up to a concentration of 10 μM (Figure 5). Both analogues differ in two structural features, one of which is a central phenyl ring present in compound **28b** instead of a pyridine ring incorporated in compound **36b**. However, this difference was not expected to have a negative effect on the activity of **28b** since, in the case of **1** and **2**, the phenyl ring in **2** has even improved potency and efficacy compared to **1**, rendering retigabine (**2**) the more active analogue of flupirtine (**1**). The key difference is, therefore, probably the additional methyl group of **36b**, causing the switch from inactive to active; such a significant difference in biological activity between two compounds resulting from a single structural modification is generally referred to as an activity cliff.^36^ Since the modification is a methyl group, it can additionally be referred to as a “magic methyl” effect in this particular case. However, it cannot be excluded that other ortho substituents instead of the methyl group would produce a comparable effect. For this reason, we choose the term “apparent magic methyl effect” in the following. Different causes are discussed to be responsible for such effects.^37−39^ For compound **36b**, since the methyl group is attached to an aromatic ring in the ortho position to an amide function, it may be for steric reasons, in combination with the methoxy group flanking the amide function on the other side, that a rotation of the amide group out of the plane of the aromatic ring occurs. On one hand, this may lead to a change in the distribution of electrons between the ring and the amide group. On the other hand, a twisted conformation may better fit the binding pocket’s active conformation, thus decreasing the conformational reordering required upon binding.^37^ A beneficial conformational preorganization through an increase in shape complementarity between the unbound substrate and the active site of the protein in the bound state caused by an ortho methyl group has been described, for example, in the binding of an inhibitor (**66**) to the p38α MAP3 kinase, where the corresponding conformational information provided by an X-ray structure has been published.^40^ A similar drastic improvement in activity through an ortho magic methyl group in the vicinity of an amide function was discovered, for example, in the development of an S1P_1_ antagonist (**64**).^41^

Compound **36a** is a structural analogue of **36b**, which differs only in the replacement of the benzylic amide side chain with an unbranched aliphatic side chain. However, this modification is associated with a significant loss of potency by a factor of 11 and a drop of efficacy to 65%. On one hand, this could be due to the reduced lipophilicity; on the other hand, it would also be conceivable that an aromatic or at least sterically more demanding side chain ensures a better affinity for the K_V_7.2/3 channel binding pocket.

Regrettably, the second retrometabolic design approach, which was based on the insertion of a methylene spacer, was unsuccessful. The four synthesized compounds (**55a/b**, **61**, **66**) were invariably inactive at a concentration of up to 10 μM in spite of the potentially advantageous methyl group. If it is taken into account that the double morpholino-substituted derivative **49**, which served as the lead structure for two of the analogues, had an EC_50_ value of 0.011 μM, the inactivity of compounds **55a/b**, **61**, and **66** indicates that the introduction of the methylene spacer was responsible for drastically impaired affinity for the binding pocket.

In addition to the activity, basic toxicological properties of the newly synthesized compounds were also investigated by using the 3-(4,5-dimethylthiazol-2-yl)-2,5-diphenyltetrazolium bromide (MTT) cell viability assay on mouse TAMH and human HEP-G2 hepatic cell lines (see Table 2). Both cell lines are established for the evaluation of hepatotoxicity.^42,43^ However, it should be noted that the values determined are not predictive for idiosyncratic toxicity since no direct dose–effect relationship can be observed for this type of toxicity.^44^ These toxic reactions typically occur even at subcritical concentrations and after a latency period as it was also observed for the flupirtine-induced hepatotoxicity, where evidence supports the theory of immune-mediated toxicity following hapten-protein adduct formation.^5,6,9,10^ This is also underlined by the results of the cell viability assay, where the drug flupirtine itself does not appear to be acutely hepatotoxic with LD_50_ values in the range of 500 μM. Retigabine showed even weaker cytotoxicity compared to flupirtine, although the in silico prediction revealed a very high probability of forming quinone diimine metabolites (Table 1). This is not a contradiction since the metabolization to quinone diimines probably requires different conditions in the case of retigabine. In contrast to flupirtine, which undergoes mainly the oxidative hepatic metabolism, retigabine is mostly *N*-glucuronidated, as has been shown earlier.^5^ However, as already mentioned, Groseclose and Castellino provided clear albeit indirect evidence for the oxidation of retigabine to quinone diimines.^12^ The reaction is probably not catalyzed by liver enzymes but possibly linked to the presence of melanin, and thus, the resulting reactive quinone diimine metabolites (Figure 1) do not primarily affect the liver but other organs such as the skin and eyes.

Despite the moderate to low in vitro toxicity of flupirtine and retigabine, it is relevant how the new analogues perform in a toxicity test since intrinsic, dose-related hepatotoxicity must also be avoided when developing new analogues of flupirtine and retigabine. A limitation in the in vitro toxicity testing was the occasional poor water solubility of the analogues. For many compounds, it was not possible to determine an LD_50_ value up to the solubility limit. For this reason and to enable comparability, in addition to the more common LD_50_ values, LD_25_ values were calculated, which express the concentration that reduced the cell viability to 75%. When comparing the LD_25_ values, most analogues were found to be more toxic compared with flupirtine and retigabine. Unfortunately, no LD_25_ value could be determined for the most active compound **36b** due to its poor aqueous solubility. Exceptions were the inverted amide analogues **17**, **21**, and **47**, which had higher LD_25_ values than flupirtine and retigabine, especially with the TAMH cell line. Since these substances are also the three least lipophilic analogues, apart from **61**, the main reason for the impaired toxicity of several other compounds is perhaps the increased lipophilicity compared to flupirtine and retigabine. The results are in agreement with the literature that suggests a connection between increased lipophilicity and increased risk for DILI.^45,46^ However, it should also be noted that a certain degree of lipophilicity of the substances is necessary in order to enable the penetration of the blood–brain barrier through passive diffusion.^47^ Since this initial work was only about a proof of concept of the retrometabolic design approach, balancing the lipophilicity was not yet the main focus of our research and remains an optimization approach for further work to improve water solubility and toxicological properties.

Finally, basic pharmacokinetic properties and the drug-likeness of the most active compound **36b** were estimated with in silico methods. Using SwissADME,^48^ the drug-likeness of **36b** according to the Lipinski rules was evaluated as given. Other drug-likeness criteria (Ghose, Veber, Egan) are fully met as well. As the log *P* value and other key figures such as the calculated low ratio of sp^3^ hybridized carbon atoms (0.18) suggest, SwissADME classified **36b** as moderately to poorly water-soluble with a predicted log *S* value in the range from −8.9 to −5.0. This is in line with our experience from biological testing, where a maximum concentration of 63 μM could be achieved in the MTT assay without compound **36b** precipitating in the assay buffer. Furthermore, the substance is classified as blood–brain barrier permeant by SwissADME, which is crucial for the effect on K_V_7 channels in the central nervous system. In addition, high gastrointestinal absorption and no impairment by the P-glycoprotein transporter are predicted, which should positively affect bioavailability. The metabolic stability was estimated with PredMS,^49^ which is a random forest model for predicting the metabolic stability of drug candidates in human liver microsomes. According to the PredMS algorithm, **36b** is assessed as being metabolically stable (≥50% remaining at 30 min).

### Computational Chemistry

The elaboration of a hypothetical binding mode and a more detailed investigation of the apparent magic methyl effect in the case of **36b** were carried out by means of a molecular docking study. The basis for the intended simulations was the recently published cryogenic electron microscopy (Cryo-EM) structure of a homotetrameric K_V_7.2 channel in complex with retigabine.^50^ To obtain conclusive docking results, the model of a heterotetrameric K_V_7.2/3 channel was created by homology modeling based on the mentioned Cryo-EM data since the channel opening activity was also tested on the Kv7.2/3 channel subtype.

**Figure 6:**
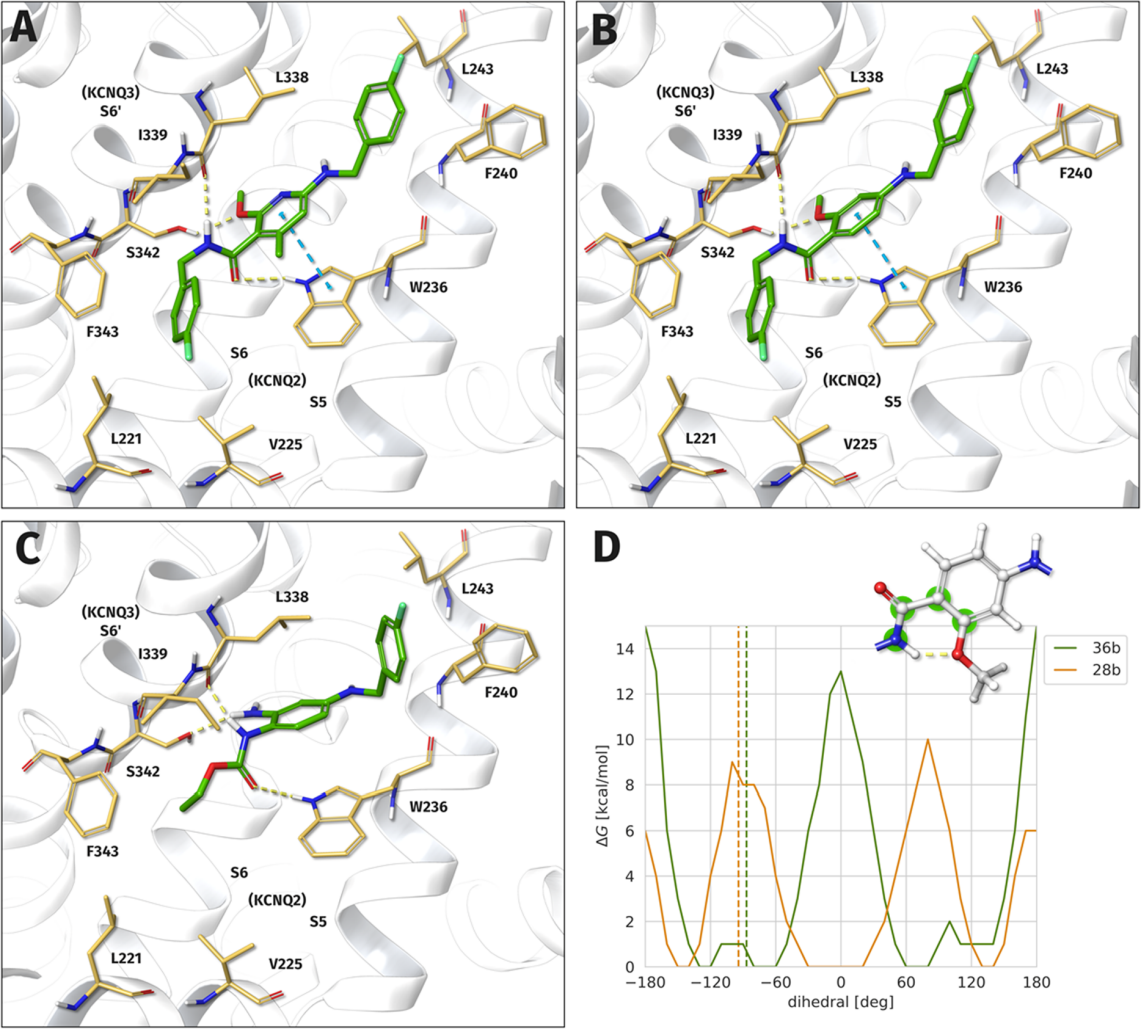
Predicted binding modes of **36b** (A) and **28b** (B) in comparison to retigabine (**2**, C) in the S5, S6, S6′-binding pocket of the heterotetrameric K_V_7.2/3 potassium channel. For **36b**, an improved binding free energy was determined compared to **28b** (|ΔΔ*G*| = 1.261 kcal/mol). All three compounds form hydrogen bonds to W236, L338, and S342 as well as hydrophobic interactions on both sides. Replacing the carbamate group of **2** with an amide slightly displaces the center aromatic ring, which then additionally forms a π–π interaction with W236. A bulky hydrophobic group at the amide substituent is able to occupy the hydrophobic cavity of L221, V225, and F343. The ortho methyl group of **36b** significantly increases the rotational energy barrier of the amide group (D, dihedral atoms highlighted in green), predicted by REMD simulations in TIP3P water. The dihedral values for the binding modes are represented as dashed lines. For **28b**, planar conformations are stabilized by an intramolecular hydrogen bond, while for conformations with a dihedral value of around (−)150°, both oxygen atoms can be bridged by a water molecule. Compared to **36b**, the binding mode dihedral value is energetically unfavorable. The Gibbs-free-energy values were estimated by Boltzmann inversion of the corresponding dihedral frequencies.

The binding poses of compounds **28b** and **36b** predicted from molecular docking are shown in Figure 6 in comparison with retigabine (**2**). The binding of all three substances is characterized by hydrophobic interactions of the lateral molecule parts and hydrogen bonds in the central area to amino acids W236, L338, and S342. However, replacing the ethyl carbamate of retigabine with an inverted amide structure appears to cause a slight shift of the central aromatic ring, which allows for an additional π–π-interaction with W236. It can also be seen that a bulky amide side chain enables a better fit in the area of the hydrophobic cavity formed by L221, V225, and F343, which could explain the improved activity of the *N*-(4-fluorobenzyl)nicotinamide analogue **36b** in comparison to the *N*-butylnicotinamide analogue **36a**.

No specific interaction of the additional methyl group with the binding site was observed in the predicted docking pose of **36b**. However, the amide function of **28b** and **36b** is clearly rotated by approximately 90° in relation to the central aromatic ring. Both findings support the hypothesis of an apparent magic methyl effect caused by a conformational preorganization of the ligand in the case of **36b**. Additional evidence was provided by a replica-exchange molecular dynamics simulation performed to calculate the rotational energy barrier of the amide group. The results are visualized in Figure 6D, which shows the one-dimensional Gibbs-free-energy landscape of **28b** and **36b** as a function of the dihedral angle. As can be seen, planar conformations are energetically favorable for compound **28b** since these are also stabilized by an intramolecular hydrogen bond involving the amide function and the adjacent methoxy group. In contrast, the additional methyl group and the resulting ortho-disubstitution of the amide function in the case of **36b** lead to an energetic preference for a dihedral angle of approximately 90°. This dihedral angle matches the conformation in the bound state obtained from molecular docking, thus improving the binding energy of **36b** by reducing the entropic cost of conformational reordering required for this interaction.

## Conclusions

In conclusion, it can be stated that the concept of a retrometabolic drug design could be successfully applied to flupirtine (**1**) and retigabine (**2**). Unfortunately, the strategy to insert a methylene spacer failed, but replacing the carbamate group of **1** and **2** with an inverted amide resulted in a compound (**36b**) six times as potent as flupirtine. However, further structural modifications, such as the introduction of an essential methyl group, which is postulated to cause an improved binding by conformational preorganization, were necessary to obtain active compounds. Additional work is required to balance compound lipophilicity by addition of an ionizable/polar moiety in order to obtain substances with attenuated toxicological properties while at the same time maintaining the improved K_V_7.2/3 channel opening activity of **36b**. If these changes are successfully implemented, the inverted amide scaffold represents a promising approach to obtain safer replacements for flupirtine and retigabine, which, as a result of their structural design, are not at risk of forming reactive quinone diimine metabolites.

## Experimental Section

### Chemistry

All solvents and reagents were obtained from commercial suppliers (Sigma Aldrich, VWR, or ABCR) and were used without purification. Anhydrous solvents were purchased from Acros Organics, except tetrahydrofuran (THF), which was dried by refluxing over sodium and benzophenone (until a permanent dark-blue coloration was visible), followed by distillation under anhydrous conditions. Microwave-assisted syntheses were performed using an Anton Paar Monowave 300 reactor operated in the closed vessel mode using G10-vials with a 6 mL total capacity; temperature control was performed by means of an integrated IR sensor, and the stirring speed was 600 rpm. NMR spectra were recorded with a Bruker Avance III device at 400 MHz (^1^H NMR) and 100 MHz (^13^C NMR). Chemical shifts were referenced to tetramethylsilane as an internal standard in deuterated solvents and reported in parts per million (ppm). Coupling constants (*J*) are reported in Hz using the abbreviations br = broad, s = singlet, d = doublet, t = triplet, q = quartet, m = multiplet, and combinations thereof. Mid-infrared spectra were recorded on an ALPHA FT-IR instrument from Bruker Optics equipped with a diamond attenuated total reflectance (ATR) accessory unit and are indicated in terms of absorption frequency [cm^–1^]. HRAM-MS data were measured using a Shimadzu LCMS-IT-TOF or a Bruker maXis LC-QTOF-MS system, each with electrospray ionization (ESI). Melting points were measured using a Melting Point M-565 apparatus from Büchi. The purity of the final compounds was determined by high-performance liquid chromatography (HPLC) (applying the 100% method at 220 nm). All final compounds are >95% pure by HPLC analysis. Preparative and analytical HPLC procedures were performed using Shimadzu devices CBM-20A, LC-20A P, SIL-20A, and FRC-10A with an SPD 20A UV/vis detector and an ELSD-LT II. In the analytical mode, a LiChroCART (250 × 4 mm) was used, and in the preparative mode, a Hibar RT (250 × 25 mm) HPLC column was used, both containing LiChrospher 100 RP-18e (5 μm). An eluent of methanol/water (75:25) with 0.1% formic acid was used unless stated otherwise. Thin-layer chromatography was executed on silica gel 60 F_254_ aluminum plates purchased from Merck. Column chromatography was performed on a glass column using silica gel 60 from Carl Roth with a particle size of 20–45 μm. For flash chromatography, the Sepacore system from Büchi was used in combination with 25 or 50 g Biotage SNAP columns.

### General Procedure A: Reductive Amination

The amine component (1.0 equiv, 22–142 mmol/L) was suspended in dry toluene. 4 Å molecular sieves (0.1 g/mL) and 4-fluorobenzaldehyde (1.0–1.2 equiv) were added. The reaction mixture was stirred at 120 °C for 5–8 h. Afterward, it was filtered hot to remove the molecular sieves. The filtrate was cooled to room temperature and concentrated under reduced pressure. The residue was dissolved in 25 mL of a mixture of 1,4-dioxane and methanol (4:1). The resulting solution was cooled to 0 °C, and sodium borohydride (2.8–5.0 equiv) was added in portions over a period of 1 h under stirring. Thereafter, stirring at room temperature was continued for 16 h before the reaction was quenched by adding 100 mL of water. The resulting mixture was extracted with ethyl acetate (250 mL). The organic phase was washed with brine, dried over Na_2_SO_4_, filtrated, and concentrated under reduced pressure. The crude residue was purified by silica gel column chromatography to obtain the title compound.

### General Procedure B: Reduction of Nitrile Functions to Primary Amino Groups

The nitrile compound (30–50 mmol/L) was dissolved in a saturated solution of ammonia in methanol (30 mL). 500 mg of a Raney nickel suspension in water (50%) was washed with methanol and added to the reaction mixture. The suspension was carefully set under a hydrogen atmosphere (balloon pressure) and stirred at 50 °C. After 6 h, the catalyst was removed by filtration, and the filtrate was concentrated under reduced pressure. The resulting residue was dissolved in ethyl acetate and filtrated through a pad of silica gel, which was subsequently rinsed with additional 250 mL of ethyl acetate. The obtained filtrate contained a side product. Afterward, the silica gel pad was rinsed with 100 mL of a mixture of methanol and conc. aq ammonia solution (9:1) to elute the desired product. The filtrate was concentrated under reduced pressure to obtain the title compound.

### 2-(4-Methyl-3-nitrophenyl)isoindoline-1,3-dione (**10**)

4-Methyl-3-nitroaniline (16.76 g, 110.2 mmol), phthalic anhydride (19.58 g, 132.2 mmol, 1.2 equiv), and triethylamine (TEA) (18.32 mL, 132.2 mmol, 1.2 equiv) were dissolved in toluene (300 mL). The mixture was stirred at 120 °C, and water was removed by using a Dean–Stark apparatus. After 4 h, it was cooled to room temperature, and the resulting precipitate was filtered off. The filter cake was washed successively with a saturated aq NaHCO_3_ solution and boiling ethyl acetate. Afterward, the resulting solid was recrystallized from dimethyl sulfoxide (DMSO) and washed with water to obtain **10** as a colorless solid (25.34 g, 89.8 mmol, 82%): *R*_*f*_ = 0.69 (ethyl acetate/*n*-hexane 1:1); mp 231 °C; ^1^H NMR (400 MHz, DMSO-*d*_6_): δ (ppm) = 8.18 (d, *J* = 2.1 Hz, 1H); 8.00 (m, 2H), 7.93 (m, 2H), 7.77 (dd, *J* = 8.2, 2.1 Hz, 1H), 7.68 (dd, *J* = 8.1, 1.0 Hz, 1H), 2.59 (s, 3H); ^13^C NMR (100 MHz, DMSO-*d*_6_): δ (ppm) = 166.6, 148.6, 134.9, 133.2, 132.7, 132.0, 131.5, 130.7, 123.6, 123.0, 19.4; IR (ATR): ν̃ = 2990 (w, ν_C–H_), 1728 (s, ν_C=O_), 1531 (m, ν_N–O_).

### *N*-(4-Methyl-3-nitrophenyl)acetamide (**12**)

4-Methyl-3-nitroaniline (11.85 g, 77.9 mmol) was dissolved in a mixture of acetic anhydride (29.4 mL, 311.5 mmol, 4.0 equiv) and acetic acid (95 mL). The reaction mixture was stirred at room temperature. After 15 min, acetic acid and excessive acetic anhydride were removed under reduced pressure. The resulting residue was recrystallized (ethanol/water) to obtain **12** as a beige solid (13.13 g, 67.6 mmol, 87%): *R*_*f*_ = 0.31 (ethyl acetate/*n*-hexane 1:1); mp 150 °C; ^1^H NMR (400 MHz, DMSO-*d*_6_): δ (ppm) = 10.30 (s, 1H), 8.36 (d, *J* = 2.3 Hz, 1H), 7.70 (dd, *J* = 8.3, 2.3 Hz, 1H), 7.42 (d, *J* = 8.4 Hz, 1H), 2.46 (s, 3H), 2.07 (s, 3H); ^13^C NMR (100 MHz, DMSO-*d*_6_): δ (ppm) = 168.9, 148.6, 138.2, 126.9, 133.0, 123.5, 114.1, 24.0, 19.1; IR (ATR): ν̃ = 3353 (m, ν_N–H_), 3042, 2993 (w, ν_C–H_), 1673 (s, ν_C=O_), 1600 (m, δ_N–H_); 1524 (s, ν_N–O_).

### 4-Acetamido-2-nitrobenzoic Acid (**13**)

*N*-(4-Methyl-3-nitrophenyl)acetamide (13.00 g, 66.9 mmol) was suspended in water and stirred at 80 °C. KMnO_4_ was added in eight portions at 30 min intervals (in total 33.85 g, 214.2 mmol, 3.2 equiv). After the last addition, the reaction mixture was continued to stir for 1 h at 80 °C. Afterward, the reaction mixture was cooled to room temperature, and the pH was adjusted to 11–12 by the addition of a 10% aq NaOH solution. Thereafter, a conc. aq H_2_O_2_ solution was added until the violet color of the mixture disappeared. The resulting brown precipitate was filtered off, and the filtrate was cooled to 0 °C. By the addition of conc. aq HCl to the filtrate, the product was precipitated and subsequently filtered off to obtain **13** as a colorless solid (7.81 g, 34.8 mmol, 52%): *R*_*f*_ = 0.32 (ethyl acetate/toluene/AcOH 5:5:1); mp 223 °C; ^1^H NMR (400 MHz, DMSO-*d*_6_): δ (ppm) = 13.57 (s, 1H), 10.61 (s, 1H), 8.19 (d, *J* = 2.0 Hz, 1H), 7.86 (d, *J* = 8.5 Hz, 1H), 7.78 (dd, *J* = 8.5, 2.1 Hz, 1H), 2.11 (s, 3H); ^13^C NMR (100 MHz, DMSO-*d*_6_): δ (ppm) = 169.5, 165.1, 149.8, 142.8, 131.4, 121.3, 119.9, 112.8, 24.1; IR (ATR): ν̃ = 3346 (m, ν_N–H_), 3300–2500 (b, ν_O–H_), 1720, 1671 (s, ν_C=O_), 1613 (m, δ_N–H_); 1528 (s, ν_N–O_).

### 4-Acetamido-*N*-isobutyl-2-nitrobenzamide (**14**)

4-Acetamido-2-nitrobenzoic acid (500 mg, 2.23 mmol) was dissolved in THF (20 mL). CDI (722 mg, 4.46 mmol, 2.0 equiv) was added in one portion, and the mixture was stirred at room temperature. After 1 h, isobutylamine (670 μL, 6.69 mmol, 3.0 equiv) was added, and the reaction mixture was stirred at room temperature for additional 16 h. Afterward, ethyl acetate (100 mL) was added. The resulting solution was extracted successively with a saturated aq NaHCO_3_ solution (100 mL) and a 2 M aq HCl solution (100 mL). The organic phase was washed with brine, dried over Na_2_SO_4_, filtrated, and concentrated under reduced pressure. The crude residue was purified by recrystallization (methanol/water) to obtain **14** as a light-yellow solid (460 mg, 1.65 mmol, 74%): *R*_*f*_ = 0.50 (ethyl acetate/*n*-hexane 4:1); mp 186 °C; ^1^H NMR (400 MHz, DMSO-*d*_6_): δ (ppm) = 10.49 (s, 1H), 8.57 (t, *J* = 5.9 Hz, 1H), 8.31 (d, *J* = 2.1 Hz, 1H), 7.80 (dd, *J* = 8.4, 2.1 Hz, 1H), 7.53 (d, *J* = 8.3 Hz, 1H), 3.02 (dd, *J* = 6.8, 5.9 Hz, 2H), 2.10 (s, 3H), 1.80 (m, 1H), 0.90 (d, *J* = 6.7 Hz, 6H); ^13^C NMR (100 MHz, DMSO-*d*_6_): δ (ppm) = 169.3, 165.2, 147.6, 140.8, 129.7, 126.9, 122.4, 113.5, 46.7, 28.0, 24.1, 20.2; IR (ATR): ν̃ = 3257 (m, ν_N–H_), 2969 (w, ν_C–H_), 1679, 1631 (s, ν_C=O_), 1603 (m, δ_N–H_); 1539 (s, ν_N–O_).

### 4-Amino-*N*-isobutyl-2-nitrobenzamide (**15**)

4-Acetamido-*N*-isobutyl-2-nitrobenzamide (800 mg, 2.86 mmol) was suspended in a 20% aq HCl solution (50 mL). The mixture was stirred at 100 °C. After 2 h, it was cooled to 0 °C, and KOH pellets were added to adjust the pH to 7. The resulting aq suspension was extracted with ethyl acetate (2 × 100 mL). The combined organic phases were washed with brine, dried over Na_2_SO_4_, filtrated, and concentrated under reduced pressure. The crude residue was purified by recrystallization (methanol/water) to obtain **15** as a yellow solid (650 mg, 2.74 mmol, 96%): *R*_*f*_ = 0.64 (ethyl acetate/*n*-hexane 1:1); mp 155 °C; ^1^H NMR (400 MHz, DMSO-*d*_6_): δ (ppm) = 8.32 (t, *J* = 5.8 Hz, 1H), 7.27 (d, *J* = 8.4 Hz, 1H), 6.97 (d, *J* = 2.3 Hz, 1H), 6.76 (dd, *J* = 8.4, 2.3 Hz, 1H), 6.04 (s, 2H), 2.97 (dd, *J* = 6.9, 5.9 Hz, 2H), 1.77 (m, 1H), 0.88 (d, *J* = 6.7 Hz, 6H); ^13^C NMR (100 MHz, DMSO-*d*_6_): δ (ppm) = 165.4, 150.9, 149.6, 129.9, 115.9, 118.4, 107.5, 46.5, 28.1, 20.2; IR (ATR): ν̃ = 3422, 3289 (m, ν_N–H_), 2958 (w, ν_C–H_), 1631 (s, ν_C=O_), 1603 (m, δ_N–H_); 1544 (m, ν_N–O_).

### 4-[(4-Fluorobenzyl)amino]-*N*-isobutyl-2-nitrobenzamide (**16**)

The synthesis was carried out according to general procedure A. 4-Amino-*N*-isobutyl-2-nitrobenzamide (260 mg, 1.10 mmol), 4-fluorobenzaldehyde (142 μL, 1.32 mmol, 1.2 equiv), toluene (50 mL), and 4 Å molecular sieves (5.0 g) were used in the first reaction step. In the second step, the reduction was performed by using 146 mg of sodium borohydride (3.85 mmol, 3.5 equiv). The purification by silica gel column chromatography (ethyl acetate/*n*-hexane 4:6) afforded **16** as a yellow solid (140 mg, 0.41 mmol, 37%): *R*_*f*_ = 0.33 (ethyl acetate/*n*-hexane 1:1); mp 106 °C; ^1^H NMR (400 MHz, DMSO-*d*_6_): δ (ppm) = 8.33 (t, *J* = 5.8 Hz, 1H), 7.38 (m, 2H), 7.30 (d, *J* = 8.5 Hz, 1H), 7.21 (t, *J* = 6.1 Hz, 1H), 7.16 (m, 2H), 7.0 (d, *J* = 2.3 Hz, 1H), 6.79 (dd, *J* = 8.5, 2.4 Hz, 1H), 4.35 (d, *J* = 6.0 Hz, 2H), 2.96 (dd, *J* = 6.9, 5.9 Hz, 2H), 1.76 (m, 1H), 0.86 (d, *J* = 6.7 Hz, 6H); ^13^C NMR (100 MHz, DMSO-*d*_6_): δ (ppm) = 165.2, 161.2 (d, *J* = 242.4 Hz), 150.0, 149.7, 135.0 (d, *J* = 3.0 Hz), 129.8, 129.1 (d, *J* = 8.1 Hz), 118.5, 115.2 (d, *J* = 21.3 Hz), 114.3, 106.4, 46.5, 45.1, 28.1, 20.1; IR (ATR): ν̃ = 3286 (m, ν_N–H_), 2959 (w, ν_C–H_), 1626 (s, ν_C=O_), 1547 (m, ν_N–O_).

### 2-Amino-4-[(4-fluorobenzyl)amino]-*N*-isobutylbenzamide (**17**)

4-[(4-Fluorobenzyl)amino]-*N*-isobutyl-2-nitrobenzamide (340 mg, 0.98 mmol) was dissolved in ethyl acetate (20 mL). SnCl_2_ (929 mg, 4.90 mmol, 5.0 equiv) was added, and the mixture was stirred at 80 °C for 30 min. Afterward, it was cooled to room temperature, and a sat. aq NaHCO_3_ solution (100 mL) was added. The resulting mixture was extracted with ethyl acetate (2 × 100 mL). The combined organic phases were washed with brine, dried over Na_2_SO_4_, filtrated, and concentrated under reduced pressure. The crude residue was purified by recrystallization (methanol/water) to obtain **17** as a slightly gray solid (210 mg, 0.67 mmol, 68%): *R*_*f*_ = 0.60 (ethyl acetate/*n*-hexane 3:2); mp 127 °C; ^1^H NMR (400 MHz, DMSO-*d*_6_): δ (ppm) = 7.70 (t, *J* = 5.8 Hz, 1H), 7.34 (m, 2H), 7.26 (d, *J* = 8.8 Hz, 1H), 7.13 (m, 2H), 6.34 (s, 2H), 6.15 (t, *J* = 6.2 Hz, 1H), 5.83 (dd, 8.7, 2.3 Hz, 1H), 5.76 (d, *J* = 2.3 Hz, 1H), 4.21 (d, *J* = 6.1 Hz, 2H), 2.95 (dd, *J* = 7.0, 5.8 Hz, 2H), 1.77 (m, 1H), 0.84 (d, *J* = 6.7 Hz, 6H); ^13^C NMR (100 MHz, DMSO-*d*_6_): δ (ppm) = 168.9, 161.0 (d, *J* = 241.8 Hz), 151.5, 151.2, 136.3 (d, *J* = 2.9 Hz), 129.1, 128.8 (d, *J* = 8.0 Hz), 114.9 (d, *J* = 21.1 Hz), 104.2, 101.6, 97.0, 46.1, 45.2, 28.1, 20.2; IR (ATR): ν̃ = 3419, 3288 (m, ν_N–H_), 3040, 2964 (w, ν_C–H_), 1619 (s, ν_C=O_), 1590 (m, δ_N–H_); ESI-HRMS: calcd for [C_18_H_22_N_3_OF + H]^+^, 316.1820; found, 316.1810; cpd purity (220 nm): 100%.

### 2-Amino-6-chloronicotinic Acid (**19**)

Procedure A: 2,6-dichloronicotinic acid (1.00 g, 5.2 mmol) was dissolved in conc. aq ammonia solution (30 mL). The mixture was stirred in a sealed vessel at 130 °C. After 8 h, the reaction mixture was cooled to 0 °C and adjusted to pH 7 with a 6 M aq HCl solution. The formed precipitate was filtered off and purified by silica gel column chromatography using diethyl ether as the mobile phase. The title compound was obtained as a colorless solid (506 mg, 2.95 mmol, 57%). Procedure B: 2,6-dichloronicotinic acid (384 mg, 2.00 mmol) was dissolved in ethanol (30 mL). CuI (76 mg, 0.40 mmol, 0.2 equiv), K_2_CO_3_ (553 mg, 4.00 mmol, 2.0 equiv), NaN_3_ (520 mg, 8.00 mmol, 4.0 equiv), and ethylenediamine (27 μL, 0.40 mmol, 0.2 equiv) were added. The mixture was set under an atmosphere of argon and stirred at 95 °C. After 11.5 h, it was cooled to room temperature, and water (30 mL) was added. Subsequently, the resulting solution was neutralized by the addition of a 1 M aq HCl solution. The resulting mixture was extracted with ethyl acetate (3 × 100 mL). The combined organic phases were washed with brine, dried over Na_2_SO_4_, filtrated, and concentrated under reduced pressure. The crude residue was purified by silica gel column chromatography [dichloromethane (DCM)/MeOH 9:1] to obtain **19** as a colorless solid (300 mg, 1.74 mmol, 87%): *R*_*f*_ = 0.69 (toluene/ethyl acetate/AcOH 5:5:1); mp decomp.; ^1^H NMR (400 MHz, DMSO-*d*_6_): δ (ppm) = 13.14 (s, 1H), 8.03 (d, *J* = 8.1 Hz, 1H), 7.55 (s, 2H), 6.63 (d, *J* = 8.1 Hz, 1H); ^13^C NMR (100 MHz, DMSO-*d*_6_): δ (ppm) = 167.9, 159.7, 153.2, 143.2, 111.0, 104.5; IR (ATR): ν̃ = 3446, 3281 (m, ν_N–H_), 3300–2500 (b, ν_O–H_), 1672 (s, ν_C=O_), 1624 (m, δ_N–H_).

### 2-Amino-*N*-butyl-6-chloronicotinamide (**20**)

2-Amino-6-chloronicotinic acid (310 mg, 1.81 mmol) was suspended in DCM (20 mL). CDI (322 mg, 1.99 mmol, 1.1 equiv) was added in one portion, and the mixture was stirred for 15 min at room temperature. Afterward, *n*-butylamine (179 μL, 1.81 mmol, 1.0 equiv) was added, and the mixture was continued to stir at room temperature. After 24 h, another 89 μL of *n*-butylamine (0.90 mmol, 0.5 equiv) was added, and the reaction was continued under the same conditions for additional 24 h. Thereafter, the mixture was concentrated under reduced pressure. The crude residue was purified by silica gel column chromatography (ethyl acetate/*n*-hexane 1:1) and successive recrystallization (methanol/water), which yielded **20** as a colorless solid (227 mg, 1.00 mmol, 55%): *R*_*f*_ = 0.47 (ethyl acetate/*n*-hexane 1:3); mp 140 °C; ^1^H NMR (400 MHz, DMSO-*d*_6_): δ (ppm) = 8.42 (t, *J* = 5.6 Hz, 1H), 7.91 (d, *J* = 8.1 Hz, 1H), 7.47 (s, 2H), 6.62 (d, *J* = 8.0 Hz, 1H), 3.21 (td, *J* = 7.1, 5.6 Hz, 2H), 1.47 (m, 2H), 1.31 (m, 2H), 0.89 (t, *J* = 7.3 Hz, 3H); ^13^C NMR (100 MHz, DMSO-*d*_6_): δ (ppm) = 166.5, 159.0, 150.9, 139.4, 110.0, 108.4, 38.7, 31.0, 19.6, 13.7; IR (ATR): ν̃ = 3454, 3292 (m, ν_N–H_), 2959 (w, ν_C–H_), 1621 (s, ν_C=O_), 1567 (m, δ_N–H_).

### 2-Amino-*N*-butyl-6-[(4-fluorobenzyl)amino]nicotinamide (**21**)

2-Amino-*N*-butyl-6-chloronicotinamide (114 mg, 0.50 mmol) was suspended in 4-fluorobenzylamine (2.86 mL, 25.00 mmol, 50.0 equiv). The mixture was stirred at 160 °C without a solvent. After 3 h, the reaction mixture was cooled to room temperature and used directly for silica gel column chromatography (ethyl acetate/*n*-hexane 1:1). The crude product was further purified by recrystallization (methanol/water), which yielded **21** as a colorless solid (25 mg, 0.08 mmol, 16%): *R*_*f*_ = 0.34 (ethyl acetate/*n*-hexane 1:1); mp 108 °C; ^1^H NMR (400 MHz, DMSO-*d*_6_): δ (ppm) = 7.73 (t, *J* = 5.6 Hz, 1H), 7.60 (d, *J* = 8.6 Hz, 1H), 7.35 (m, 2H), 7.13 (m, 3H), 7.02 (s, 2H), 5.71 (d, *J* = 8.6 Hz, 1H), 4.44 (d, *J* = 6.0 Hz, 2H), 3.15 (td, *J* = 7.1, 5.6 Hz, 2H), 1.44 (tt, *J* = 7.9, 6.4 Hz, 2H), 1.29 (m, 2H), 0.88 (t, *J* = 7.32 Hz, 3H); ^13^C NMR (100 MHz, DMSO-*d*_6_): δ (ppm) = 167.7, 161.0 (d, *J* = 241.8 Hz), 159.3, 159.1, 137.1, 136.7 (d, *J* = 3.0 Hz), 129.2 (d, *J* = 7.9 Hz), 114.8 (d, *J* = 21.1 Hz), 97.1, 96.0, 43.0, 38.4, 31.5, 19.7, 13.7; IR (ATR): ν̃ = 3419, 3400, 3292, (m, ν_N–H_), 2961 (w, ν_C–H_), 1590 (s, ν_C=O_), 1537 (m, δ_N–H_); ESI-HRMS: calcd for [C_17_H_21_N_4_OF + H]^+^, 317.1772; found, 317.1763; cpd purity (220 nm): 100%.

### 2-Bromo-1-methyl-4-nitrobenzene (**23**)

A mixture of 1-methyl-4-nitrobenzene (10.00 g, 72.9 mmol) and iron powder (163 mg, 2.92 mmol, 0.04 equiv) was melted by heating to 80 °C. To the resulting melt, bromine (4.48 mL, 87.5 mmol, 1.2 equiv) was added dropwise under stirring over a period of 10 min. Afterward, the reaction mixture was continued to stir at 80 °C. After 1.5 h, a 10% aq solution of KOH (110 mL) was added. A precipitate formed, which was filtered off. The filter cake was dissolved in DCM (250 mL), and the resulting solution was extracted with water (2 × 250 mL). The organic phase was washed with brine, dried over Na_2_SO_4_, filtrated, and concentrated under reduced pressure. The crude residue was purified by recrystallization (ethanol) to obtain **23** as a colorless solid (8.60 g, 39.8 mmol, 55%): *R*_*f*_ = 0.50 (*n*-hexane); mp 80 °C; ^1^H NMR (400 MHz, DMSO-*d*_6_): δ (ppm) = 8.38 (d, *J* = 2.4 Hz, 1H), 8.16 (dd, *J* = 8.4, 2.4 Hz, 1H), 7.66 (dd, *J* = 8.4, 0.8 Hz, 1H), 2.47 (d, *J* = 0.6 Hz, 3H); ^13^C NMR (100 MHz, DMSO-*d*_6_): δ (ppm) = 146.3, 145.7, 131.8, 126.7, 124.2, 122.5, 22.6; IR (ATR): ν̃ = 3094, 2961 (w, ν_C–H_), 1517 (m, ν_N–O_).

### 2-Bromo-4-nitrobenzoic Acid (**24**)

2-Bromo-1-methyl-4-nitrobenzene (8.50 g, 39.4 mmol) was suspended in a mixture of water (34 mL) and pyridine (68 mL). The mixture was heated to 100 °C, and KMnO_4_ was added in 10 portions at 30 min intervals (in total 34.82 g, 220.4 mmol, 5.6 equiv). After the last 30 min interval, additional KMnO_4_ (3.33 g, 59.0 mmol, 1.5 equiv) was added, and the reaction mixture was continued to stir for 12 h at 100 °C. Afterward, the reaction mixture was cooled to room temperature and filtered through a pad of Celite. The filtrate was cooled to 0 °C, and the product was precipitated by the addition of conc. aq HCl. The resulting precipitate was filtered off and recrystallized (ethyl acetate) to obtain **24** as a colorless solid (1.72 g, 7.0 mmol, 18%): *R*_*f*_ = 0.74 (*n*-butanol/AcOH/water 8:1:1); mp 127 °C; ^1^H NMR (400 MHz, DMSO-*d*_6_): δ (ppm) = 14.06 (s, 1H), 8.48 (d, *J* = 2.2 Hz, 1H), 8.22 (dd, *J* = 8.5, 2.2 Hz, 1H), 7.95 (d, *J* = 8.5 Hz, 1H); ^13^C NMR (100 MHz, DMSO-*d*_6_): δ (ppm) = 166.5, 148.7, 140.1, 131.1, 128.2, 122.8, 119.9; IR (ATR): ν̃ = 3097 (w, ν_C–H_), 1517 (m, ν_N–O_).

### 2-Bromo-*N*-isobutyl-4-nitrobenzamide (**25a**)

2-Bromo-4-nitrobenzoic acid (1.00 g, 4.1 mmol) was dissolved in toluene (50 mL) and treated with thionyl chloride (650 μL, 8.95 mmol, 2.2 equiv) at room temperature. The reaction mixture was stirred at 120 °C for 8 h. Subsequently, the volatiles were removed under reduced pressure. The residue was dissolved in toluene and concentrated under reduced pressure again. The resulting oil was dissolved in DCM (20 mL) and added dropwise to a solution of isobutylamine (1223 μL, 12.21 mmol, 3.0 equiv) in DCM (20 mL) at 0 °C under stirring. Thereafter, the reaction mixture was warmed to room temperature and stirred for 16 h. Afterward, it was extracted successively with a sat. aq NaHCO_3_ solution (50 mL) and a 1 M aq HCl solution (50 mL). The organic phase was washed with brine, dried over Na_2_SO_4_, filtrated, and concentrated under reduced pressure. The crude residue was purified by recrystallization (ethanol/water) to obtain **25a** as a colorless solid (880 mg, 2.92 mmol, 72%): *R*_*f*_ = 0.50 (ethyl acetate/*n*-hexane 3:7); mp 136 °C; ^1^H NMR (400 MHz, DMSO-*d*_6_): δ (ppm) = 8.66 (t, *J* = 5.9 Hz, 1H), 8.45 (d, *J* = 2.2 Hz, 1H), 8.26 (dd, *J* = 8.4, 2.3 Hz, 1H), 7.64 (d, *J* = 8.4 Hz, 1H), 3.08 (dd, *J* = 6.8, 5.9 Hz, 2H), 1.82 (dq, *J* = 13.4, 6.7 Hz, 1H), 0.93 (d, *J* = 6.7 Hz, 6H); ^13^C NMR (100 MHz, DMSO-*d*_6_): δ (ppm) = 165.9, 147.9, 145.3, 129.7, 127.4, 122.8, 119.4, 46.5, 28.0, 20.2; IR (ATR): ν̃ = 3246 (m, ν_N–H_), 3098, 2958 (w, ν_C–H_), 1642 (s, ν_C=O_), 1592 (m, δ_N–H_), 1519 (m, ν_N–O_).

### 2-Bromo-*N*-(4-fluorobenzyl)-4-nitrobenzamide (**25b**)

2-Bromo-4-nitrobenzoic acid (1.50 g, 6.1 mmol) was dissolved in toluene (75 mL) and treated with thionyl chloride (974 μL, 13.41 mmol, 2.2 equiv) at room temperature. The reaction mixture was stirred at 120 °C for 2.5 h. Subsequently, the volatiles were removed under reduced pressure. The residue was dissolved in toluene and concentrated under reduced pressure again. The resulting oil was dissolved in DCM (20 mL) and added dropwise to a solution of 4-fluorobenzylamine (1046 μL, 9.15 mmol, 1.5 equiv) and TEA (1691 μL, 12.20 mmol, 2.0 equiv) in DCM (20 mL) at 0 °C under stirring. Thereafter, the reaction mixture was warmed to room temperature and stirred for 2.5 h. Afterward, it was extracted successively with a sat. aq NaHCO_3_ solution (50 mL) and a 1 M aq HCl solution (50 mL). The organic phase was washed with brine, dried over Na_2_SO_4_, filtrated, and concentrated under reduced pressure. The crude residue was purified by recrystallization (ethanol/water) to obtain **25b** as a beige solid (910 mg, 2.58 mmol, 42%): *R*_*f*_ = 0.72 (ethyl acetate/*n*-hexane 1:1); mp 184 °C; ^1^H NMR (400 MHz, DMSO-*d*_6_): δ (ppm) = 9.20 (t, *J* = 6.0 Hz, 1H), 8.46 (d, *J* = 2.2 Hz, 1H), 8.28 (dd, *J* = 8.4, 2.2 Hz, 1H), 7.70 (d, *J* = 8.4 Hz, 1H), 7.42 (m, 2H), 7.19 (m, 2H), 4.47 (d, *J* = 5.92 Hz, 2H); ^13^C NMR (100 MHz, DMSO-*d*_6_): δ (ppm) = 165.9, 161.3 (d, *J* = 242.6 Hz), 148.1, 144.6, 134.8 (d, *J* = 3.0 Hz), 129.8, 129.4 (d, *J* = 8.1 Hz), 127.5, 122.8, 119.5, 115.1 (d, *J* = 21.4 Hz), 41.8; IR (ATR): ν̃ = 3256 (m, ν_N–H_), 3075, 2941 (w, ν_C–H_), 1638 (s, ν_C=O_), 1588 (m, δ_N–H_), 1521 (m, ν_N–O_).

### *N*-Isobutyl-2-methoxy-4-nitrobenzamide (**26a**)

2-Bromo-*N*-isobutyl-4-nitrobenzamide (1.20 g, 4.0 mmol) was dissolved in methanol (20 mL). CuI (76 mg, 0.40 mmol, 0.1 equiv), K_2_CO_3_ (1.10 g, 8.0 mmol, 2.0 equiv), and ethane-1,2-diamine (13 μL, 0.20 mmol, 0.05 equiv) were added. Subsequently, the reaction mixture was set under a nitrogen atmosphere and stirred at 95 °C. After 15 h, it was filtered hot, and the resulting filtrate was concentrated under reduced pressure. The crude residue was purified by silica gel column chromatography (ethyl acetate/*n*-hexane 3:7) and successive recrystallization (methanol/water) to obtain **26a** as a colorless solid (460 mg, 1.82 mmol, 46%): *R*_*f*_ = 0.65 (ethyl acetate/*n*-hexane 3:7); mp 101 °C; ^1^H NMR (400 MHz, DMSO-*d*_6_): δ (ppm) = 7.87 (m, 1H), 8.34 (t, *J* = 5.6 Hz, 1H), 7.86 (s, 1H), 7.75 (m, 1H), 3.97 (s, 3H), 3.10 (dd, *J* = 6.9, 6.0 Hz, 2H), 1.82 (m, 1H), 0.91 (d, *J* = 6.7 Hz, 6H); ^13^C NMR (100 MHz, DMSO-*d*_6_): δ (ppm) = 164.0, 156.8, 149.1, 130.5, 131.3, 115.4, 106.7, 56.5, 46.5, 28.0, 20.0; IR (ATR): ν̃ = 3314 (m, ν_N–H_), 3118, 2956 (w, ν_C–H_), 1642 (s, ν_C=O_), 1545 (m, δ_N–H_), 1517 (m, ν_N–O_).

### *N*-(4-Fluorobenzyl)-2-methoxy-4-nitrobenzamide (**26b**)

The synthesis was carried out as described for **26a**. 2-Bromo-*N*-(4-fluorobenzyl)-4-nitrobenzamide (800 mg, 2.27 mmol), methanol (25 mL), CuI (43 mg, 0.40 mmol 0.1 equiv), K_2_CO_3_ (627 mg, 4.54 mmol, 2.0 equiv), and ethane-1,2-diamine (7.6 μL, 0.11 mmol, 0.05 equiv) were used. The title compound was obtained as a colorless solid (350 mg, 1.15 mmol, 51%): *R*_*f*_ = 0.58 (ethyl acetate/*n*-hexane 1:1); mp 104 °C; ^1^H NMR (400 MHz, DMSO-*d*_6_): δ (ppm) = 8.93 (t, *J* = 6.0 Hz, 1H), 7.88 (m, 2H), 7.82 (d, *J* = 8.9 Hz, 1H), 7.38 (m, 2H), 7.17 (m, 2H), 4.47 (d, *J* = 6.1 Hz, 2H), 3.99 (s, 3H); ^13^C NMR (100 MHz, DMSO-*d*_6_): δ (ppm) = 164.1, 161.2 (d, *J* = 242.1 Hz), 157.0, 149.3, 135.4 (d, *J* = 3.0 Hz), 130.7, 130.5, 129.0 (d, *J* = 8.1 Hz), 115.4, 115.0 (d, *J* = 21.3 Hz), 106.9, 56.6, 41.9; IR (ATR): ν̃ = 3372 (m, ν_N–H_), 3013, 2946 (w, ν_C–H_), 1641 (s, ν_C=O_), 1605 (m, δ_N–H_), 1508 (m, ν_N–O_).

### 4-Amino-*N*-isobutyl-2-methoxybenzamide (**27a**)

*N*-Isobutyl-2-methoxy-4-nitrobenzamide (440 mg, 1.74 mmol) was dissolved in ethyl acetate (35 mL). SnCl_2_ (1.65 g, 8.7 mmol, 5.0 equiv) was added, and the mixture was stirred at 80 °C for 30 min. Afterward, it was cooled to room temperature, and a sat. aq NaHCO_3_ solution (100 mL) was added. The resulting mixture was extracted with ethyl acetate (2 × 100 mL). The combined organic phases were washed with brine, dried over Na_2_SO_4_, filtrated, and concentrated under reduced pressure to obtain **27a** as a brown oil (380 mg, 1.71 mmol, 98%): *R*_*f*_ = 0.78 (ethyl acetate); ^1^H NMR (400 MHz, DMSO-*d*_6_): δ (ppm) = 7.81 (t, *J* = 5.8 Hz, 1H), 7.60 (d, *J* = 8.4 Hz, 1H), 6.24 (d, *J* = 2.0 Hz, 1H), 6.19 (dd, *J* = 8.4, 2.0 Hz, 1H), 5.68 (s, 2H), 3.82 (s, 3H), 3.09 (dd, *J* = 6.8, 5.8 Hz, 2H), 1.78 (m, 1H), 0.88 (d, *J* = 6.7 Hz, 6H); ^13^C NMR (100 MHz, DMSO-*d*_6_): δ (ppm) = 164.7, 158.8, 153.1, 132.5, 109.2, 106.1, 96.1, 55.5, 46.2, 28.1, 20.1; IR (ATR): ν̃ = 3402, 3339, 3222, (m, ν_N–H_), 2957 (w, ν_C–H_), 1628 (s, ν_C=O_), 1595 (s, δ_N–H_).

### 4-Amino-*N*-(4-fluorobenzyl)-2-methoxybenzamide (**27b**)

The synthesis was carried out as described for **27a**. *N*-(4-Fluorobenzyl)-2-methoxy-4-nitrobenzamide (300 mg, 0.99 mmol) and SnCl_2_ (935 mg, 4.93 mmol, 5.0 equiv) were used. The title compound was obtained as a brown solid (280 mg, 1.02 mmol, 100%): *R*_*f*_ = 0.35 (ethyl acetate/*n*-hexane 6:4); mp 126 °C; ^1^H NMR (400 MHz, DMSO-*d*_6_): δ (ppm) = 8.35 (t, *J* = 6.1 Hz, 1H), 7.63 (d, *J* = 8.5 Hz, 1H), 7.32 (m, 2H), 7.13 (m, 2H), 6.24 (d, *J* = 2.0 Hz, 1H), 6.19 (dd, *J* = 8.6, 2.0 Hz, 1H), 5.72 (s, 2H), 4.44 (d, *J* = 6.1 Hz, 2H), 3.81 (s, 3H); ^13^C NMR (100 MHz, DMSO-*d*_6_): δ (ppm) = 164.9, 161.0 (d, *J* = 241.7 Hz), 159.1, 153.3, 136.6 (d, *J* = 2.9 Hz), 132.6, 129.0 (d, *J* = 8.0 Hz), 114.9 (d, *J* = 21.1 Hz), 108.7, 106.1, 95.9, 55.4, 41.7; IR (ATR): ν̃ = 3446, 3392, 3346 (m, ν_N–H_), 1627 (s, ν_C=O_), 1588 (m, δ_N–H_).

### 4-[(4-Fluorobenzyl)amino]-*N*-isobutyl-2-methoxybenzamide (**28a**)

The synthesis was carried out according to general procedure A. 4-Amino-*N*-isobutyl-2-methoxybenzamide (360 mg, 1.62 mmol), 4-fluorobenzaldehyde (209 μL, 1.94 mmol, 1.2 equiv), toluene (30 mL), and 4 Å molecular sieves (3.0 g) were used in the first reaction step. In the second step, the reduction was performed by using 258 mg of sodium borohydride (6.80 mmol, 4.2 equiv). The purification by silica gel column chromatography (ethyl acetate/*n*-hexane 7:3) afforded **28a** as a colorless solid (220 mg, 0.67 mmol, 41%): *R*_*f*_ = 0.59 (ethyl acetate/*n*-hexane 7:3); mp 154 °C; ^1^H NMR (400 MHz, DMSO-*d*_6_): δ (ppm) = 7.80 (t, *J* = 5.8 Hz, 1H), 7.61 (d, *J* = 8.5 Hz, 1H), 7.39 (m, 2H), 7.15 (m, 2H), 6.83 (t, *J* = 6.1 Hz, 1H), 6.25 (d, *J* = 2.1 Hz, 1H), 6.22 (dd, *J* = 8.5, 2.1 Hz, 1H), 4.31 (d, *J* = 6.0 Hz, 2H), 3.80 (s, 3H), 3.08 (dd, *J* = 6.8, 5.9 Hz, 2H), 1.77 (m, 1H), 0.87 (d, *J* = 6.7 Hz, 6H); ^13^C NMR (100 MHz, DMSO-*d*_6_): δ (ppm) = 164.7, 161.1 (d, *J* = 242.0 Hz), 158.7, 152.3, 135.8 (d, *J* = 3.1 Hz), 132.3, 129.1 (d, *J* = 8.1 Hz), 115.0 (d, *J* = 21.2 Hz), 109.6, 104.5, 95.0, 55.5, 46.2, 45.3, 28.1, 20.1; IR (ATR): ν̃ = 3404, 3299 (m, ν_N–H_), 3068, 2954 (w, ν_C–H_), 1629 (s, ν_C=O_), 1593 (s, δ_N–H_); ESI-HRMS: calcd for [C_19_H_23_N_2_O_2_F + H]^+^, 331.1816; found, 331.1803; cpd purity (220 nm): 100%.

### *N*-(4-Fluorobenzyl)-4-[(4-fluorobenzyl)amino]-2-methoxybenzamide (**28b**)

The synthesis was carried out according to general procedure A. 4-Amino-*N*-(4-fluorobenzyl)-2-methoxybenzamide (250 mg, 0.91 mmol), 4-fluorobenzaldehyde (196 μL, 1.08 mmol, 1.2 equiv), toluene (20 mL), and 4 Å molecular sieves (2.0 g) were used in the first reaction step. In the second step, the reduction was performed by using 96 mg of sodium borohydride (2.55 mmol, 2.8 equiv). The purification by silica gel column chromatography (ethyl acetate/*n*-hexane 1:1) afforded **28b** as a colorless solid (240 mg, 0.63 mmol, 69%): *R*_*f*_ = 0.42 (ethyl acetate/*n*-hexane 3:2); mp 180 °C; ^1^H NMR (400 MHz, DMSO-*d*_6_): δ (ppm) = 8.35 (t, *J* = 6.1 Hz, 1H), 7.64 (d, *J* = 8.4 Hz, 1H), 7.39 (m, 2H), 7.31 (m, 2H), 7.13 (m, 4H), 6.87 (t, *J* = 6.0 Hz, 1H), 6.24 (m, 2H), 4.43 (d, *J* = 6.1 Hz, 2H), 4.32 (d, *J* = 6.0 Hz, 2H), 3.79 (s, 3H); ^13^C NMR (100 MHz, DMSO-*d*_6_): δ (ppm) = 164.8, 161.1 (d, *J* = 240.0 Hz), 161.0 (d, *J* = 240.0 Hz), 158.9, 152.6, 136.5 (d, *J* = 2.9 Hz), 135.8 (d, *J* = 2.9 Hz), 132.5, 129.2 (d, *J* = 8.1 Hz), 129.0 (d, *J* = 8.1 Hz), 115.1 (d, *J* = 18.5 Hz), 114.8 (d, *J* = 18.5 Hz), 109.2, 104.5, 94.9, 55.4, 45.3, 41.7; IR (ATR): ν̃ = 3401, 3324 (m, ν_N–H_), 3022, 2922 (w, ν_C–H_), 1591 (s, ν_C=O_), 1542 (m, δ_N–H_); ESI-HRMS: calcd for [C_22_H_20_N_2_O_2_F_2_ + H]^+^, 383.1566; found, 383.1564; cpd purity (220 nm): 100%.

### 2,6-Dihydroxy-4-methylnicotinonitrile (**31**)

2-Cyanoacetamide (28.0 g, 400 mmol), ethyl 3-oxobutanoate (50.5 mL, 400.0 mmol, 1.0 equiv), and KOH (28.0 g, 508 mmol, 1.3 equiv) were dissolved in methanol (200 mL). The reaction mixture was stirred at 65 °C. After 2 h, it was cooled to room temperature. A colorless precipitate, which was formed while cooling, was filtered off and washed with ethanol. Afterward, the filter cake was redissolved in hot water. The resulting solution was acidified with conc. aq HCl, and again a precipitate formed, which was filtered off and washed with ethanol to obtain **31** as a colorless solid (45.1 g, 300 mmol, 75%): *R*_*f*_ = 0.70 (*n*-butanol/AcOH/water 8:1:1); mp decomp.; ^1^H NMR (400 MHz, DMSO-*d*_6_): δ (ppm) = 5.25 (s, 1H), 2.09 (s, 3H); ^13^C NMR HMBC (100 MHz, DMSO-*d*_6_): δ (ppm) = 163.4 (5), 162.9 (1), 157.1 (3), 119.2 (7), 95.6 (2), 82.7 (4), 20.6 (6); IR (ATR): ν̃ = 2221 (m, ν_C≡N_).

### 2,6-Dichloro-4-methylnicotinonitrile (**32**)

2,6-Dihydroxy-4-methylnicotinonitrile (1.25 g, 8.3 mmol) and benzyltrimethylammonium chloride (7.50 g, 40.4 mmol, 4.9 equiv) were dissolved in phosphorus oxychloride (8.0 mL, 87.7 mmol, 10.5 equiv). The reaction mixture was stirred at 90 °C in an apparatus equipped with a reflux condenser and a CaCl_2_ drying tube. After 16 h, it was cooled to room temperature and carefully poured on ice water. A colorless precipitate formed, which was filtered off and washed with water. The crude product was recrystallized from methanol/water to obtain the title product as a colorless solid (857 mg, 4.58 mmol, 55%): *R*_*f*_ = 0.74 (ethyl acetate/*n*-hexane 1:3); mp 112 °C; ^1^H NMR (400 MHz, DMSO-*d*_6_): δ (ppm) = 7.82 (q, *J* = 0.7 Hz, 1H), 2.54 (d, *J* = 0.7 Hz, 3H); ^13^C NMR (100 MHz, DMSO-*d*_6_): δ (ppm) = 158.2, 152.2, 150.8, 124.8, 113.7, 110.1, 20.1; IR (ATR): ν̃ = 3058, 2978 (w, ν_C–H_), 2221 (m, ν_C≡N_).

### 2,6-Dichloro-4-methylnicotinic Acid (**33**)

2,6-Dichloro-4-methylnicotinonitrile (5.89 g, 31.5 mmol) was suspended in a mixture of conc. sulfuric acid (13.0 mL) and conc. nitric acid (4.5 mL). The mixture was stirred at 105 °C. After 2 h, it was cooled to room temperature and carefully poured on ice water (250 mL). The resulting aq suspension was extracted with ethyl acetate (3 × 250 mL). The combined organic phases were washed with brine, dried over Na_2_SO_4,_ and concentrated under reduced pressure. The resulting residue was redissolved in a 1 M aq NaOH solution (100 mL). Afterward, the solution was filtered, and the filtrate was acidified with conc. aq HCl. A precipitate formed, which was filtered off and washed with water to obtain **33** as a colorless solid (5.01 g, 24.3 mmol, 77%): *R*_*f*_ = 0.59 (*n*-butanol/AcOH/water 8:1:1); mp 141 °C; ^1^H NMR (400 MHz, DMSO-*d*_6_): δ (ppm) = 14.22 (s, 1H), 7.61 (s, 1H), 2.35 (s, 3H); ^13^C NMR (100 MHz, DMSO-*d*_6_): δ (ppm) = 166.0, 150.7, 148.7, 144.6, 130.5, 124.9, 18.7; IR (ATR): ν̃ = 3300–2500 (b, ν_O–H_), 1699 (s, ν_C=O_).

### 6-Chloro-2-methoxy-4-methylnicotinic Acid (**34**)

A 60% suspension of NaH in mineral oil (2.43 g, 60.7 mmol, 2.5 equiv) was suspended in dry THF (50 mL) under an atmosphere of argon. The mixture was cooled to 0 °C. Methanol (1080 μL, 26.70 mmol, 1.1 equiv) and a solution of 2,6-dichloro-4-methylnicotinic acid (5.00 g, 24.3 mmol) in dry THF (50 mL) were added successively. The reaction mixture was warmed to 70 °C and stirred for 7 h while maintaining the temperature. Subsequently, the reaction mixture was cooled to room temperature and quenched by adding water (100 mL). The resulting aq mixture was adjusted to pH 12 by the addition of a 2 M aq NaOH solution and extracted with ethyl acetate (100 mL). The organic phase was discarded, and the aq phase was adjusted to pH 2–3 with conc. aq HCl. Subsequently, it was extracted with ethyl acetate (2 × 200 mL). The combined organic phases were washed with brine, dried over Na_2_SO_4_, filtrated, and concentrated under reduced pressure to obtain **34** as a beige solid (4.72 g, 23.4 mmol, 96%): *R*_*f*_ = 0.78 (*n*-butanol/AcOH/water 8:1:1); mp 166 °C; ^1^H NMR (400 MHz, DMSO-*d*_6_): δ (ppm) = 13.39 (s, 1H), 7.09 (s, 1H), 3.87 (s, 3H), 2.17 (s, 3H); ^13^C NMR (100 MHz, DMSO-*d*_6_): δ (ppm) = 166.6, 159.4, 149.8, 147.0, 117.4, 118.0, 54.3, 18.4; IR (ATR): ν̃ = 3002, 2954 (w, ν_C–H_), 3300–2500 (b, ν_O–H_), 1686 (s, ν_C=O_).

### *N*-Butyl-6-chloro-2-methoxy-4-methylnicotinamide (**35a**)

6-Chloro-2-methoxy-4-methylnicotinic acid (2.19 g, 10.9 mmol) was dissolved in dry THF (15 mL), and one drop of dry dimethylformamide (DMF) was added. The mixture was cooled to 0 °C, and a solution of oxalyl chloride (1860 μL, 21.75 mmol, 2.0 equiv) in dry THF (15 mL) was added dropwise. After complete addition, the cooling was removed, and the reaction mixture was stirred at room temperature for 2 h. Subsequently, all volatiles were removed under reduced pressure, and the residue was dissolved in 20 mL of dry THF. The resulting solution was added dropwise to a solution of *n*-butylamine (3.22 mL, 32.6 mmol, 3.0 equiv) in dry THF (20 mL) at 0 °C. Afterward, the cooling was removed, and the reaction mixture was stirred at room temperature. After 16 h, ethyl acetate (200 mL) was added. The resulting solution was extracted with water (2 × 100 mL), washed with brine, dried over Na_2_SO_4_, filtrated, and concentrated under reduced pressure. The crude residue was purified by silica gel column chromatography (ethyl acetate/*n*-hexane 3:7), which yielded **35a** as a colorless solid (1.71 g, 6.7 mmol, 61%): *R*_*f*_ = 0.81 (ethyl acetate/*n*-hexane 2:1); mp 57 °C; ^1^H NMR (400 MHz, DMSO-*d*_6_): δ (ppm) = 8.30 (t, *J* = 5.7 Hz, 1H), 7.04 (s, 1H), 3.82 (s, 3H), 3.19 (td, *J* = 6.9, 5.7 Hz, 2H), 2.19 (s, 3H), 1.45 (m, 2H), 1.34 (m, 2H), 0.89 (t, *J* = 7.3 Hz, 3H); ^13^C NMR (100 MHz, DMSO-*d*_6_): δ (ppm) = 164.1, 159.6, 149.7, 146.1, 120.4, 117.8, 54.0, 38.3, 31.0, 19.5, 18.0, 13.6; IR (ATR): ν̃ = 3220 (m, ν_N–H_), 3068, 2961 (w, ν_C–H_), 1645 (s, ν_C=O_), 1624 (m, δ_N–H_).

### 6-Chloro-*N*-(4-fluorobenzyl)-2-methoxy-4-methylnicotinamide (**35b**)

6-Chloro-2-methoxy-4-methylnicotinic acid (500 mg, 2.48 mmol) was dissolved in dry THF (15 mL), and one drop of dry DMF was added. The mixture was cooled to 0 °C, and a solution of oxalyl chloride (638 μL, 7.44 mmol, 3.0 equiv) in dry THF (15 mL) was added dropwise. After complete addition, the cooling was removed, and the reaction mixture was stirred at room temperature for 3 h. Subsequently, all volatiles were removed under reduced pressure. The residue was dissolved in 10 mL of dry DCM. The resulting solution was added dropwise to a solution of 4-fluorobenzylamine (340 μL, 2.98 mmol, 1.2 equiv) and TEA (691 μL, 4.96 mmol, 2.0 equiv) in dry DCM (10 mL) at 0 °C. Afterward, the cooling was removed, and the reaction mixture was stirred at room temperature. After 16 h, additional DCM (100 mL) was added. The resulting solution was extracted successively with a saturated aq NaHCO_3_ solution (100 mL) and a 2 M aq HCl solution (100 mL). The organic phase was washed with brine, dried over Na_2_SO_4_, filtrated, and concentrated under reduced pressure. The crude residue was purified by silica gel column chromatography (ethyl acetate/*n*-hexane 3:2), which yielded **35b** as a colorless solid (402 mg, 1.30 mmol, 53%): *R*_*f*_ = 0.29 (ethyl acetate/*n*-hexane 1:1); mp 127 °C; ^1^H NMR (400 MHz, DMSO-*d*_6_): δ (ppm) = 8.89 (t, *J* = 6.0 Hz, 1H), 7.38 (m, 2H), 7.18 (m, 2H), 7.05 (d, *J* = 0.6 Hz, 1H), 4.42 (d, *J* = 6.0 Hz, 2H), 3.86 (s, 3H), 2.18 (d, *J* = 0.6 Hz, 3H); ^13^C NMR (100 MHz, DMSO-*d*_6_): δ (ppm) = 164.5, 161.2 (d, *J* = 242.1 Hz), 159.6, 149.9, 146.4, 135.3 (d, *J* = 3.0 Hz), 129.1 (d, *J* = 8.1 Hz), 119.9, 117.9, 115.0 (d, *J* = 21.3 Hz), 54.1, 41.5, 18.0; IR (ATR): ν̃ = 3310 (m, ν_N–H_), 3067, 2954 (w, ν_C–H_), 1635 (s, ν_C=O_), 1604 (m, δ_N–H_).

### *N*-Butyl-6-[(4-fluorobenzyl)amino]-2-methoxy-4-methylnicotinamide (**36a**)

In a microwave vessel, *N*-butyl-6-chloro-2-methoxy-4-methylnicotinamide (788 mg, 3.07 mmol) was dissolved in 4-fluorobenzylamine (2.50 mL, 21.77 mmol, 7.1 equiv). The mixture was stirred in a microwave reactor at 165 °C for 1 h in a closed vessel. Subsequently, the reaction mixture was cooled to room temperature, dissolved in ethyl acetate (200 mL), and extracted with water (2 × 100 mL). The organic phase was washed with brine, dried over Na_2_SO_4_, filtrated, and concentrated under reduced pressure. The crude residue was purified by flash chromatography (mobile phase: ethyl acetate/*n*-hexane with 0–60% ethyl acetate) and successive recrystallization (ethanol/water), which yielded **36a** as a colorless solid (73 mg, 0.21 mmol, 7%): *R*_*f*_ = 0.61 (ethyl acetate/*n*-hexane 2:1); mp 132 °C; ^1^H NMR (400 MHz, DMSO-*d*_6_): δ (ppm) = 7.84 (t, *J* = 5.7 Hz, 1H), 7.35 (m, 2H), 7.16 (t, *J* = 5.7 Hz, 1H), 5.88 (s, 1H), 7.11 (m, 2H), 4.42 (d, *J* = 6.1 Hz, 2H), 3.69 (s, 3H), 3.12 (td, *J* = 6.8, 5.7 Hz, 2H), 2.04 (s, 3H), 1.41 (m, 2H), 1.31 (m, 2H), 0.87 (t, *J* = 7.3 Hz, 3H); ^13^C NMR (100 MHz, DMSO-*d*_6_): δ (ppm) = 166.0, 161.0 (d, *J* = 241.65 Hz), 159.3, 156.5, 147.5, 137.1 (d, *J* = 3.0 Hz), 129.0 (d, *J* = 8.0 Hz), 114.8 (d, *J* = 21.1 Hz), 108.3, 100.0, 52.6, 43.5, 38.3, 31.2, 19.5, 18.9, 13.7; IR (ATR): ν̃ = 3281 (m, ν_N–H_), 2944 (w, ν_C–H_), 1601 (s, ν_C=O_), 1544 (m, δ_N–H_); ESI-HRMS: calcd for [C_19_H_25_N_3_O_2_F + H]^+^, 346.1925; found, 346.1929; cpd purity (220 nm): 100%.

### *N*-(4-Fluorobenzyl)-6-[(4-fluorobenzyl)amino]-2-methoxy-4-methylnicotinamide (**36b**)

The synthesis was carried out as described for **36a**. 6-Chloro-*N*-(4-fluorobenzyl)-2-methoxy-4-methylnicotinamide (400 mg, 1.30 mmol) and 4-fluorobenzylamine (1490 μL, 12.96 mmol, 10.0 equiv) were used. The purification by flash chromatography (mobile phase: ethyl acetate/*n*-hexane with 50–100% ethyl acetate) and successive recrystallization (ethanol/water) afforded **36b** as a colorless solid (121 mg, 0.31 mmol, 23%): *R*_*f*_ = 0.80 (ethyl acetate/*n*-hexane 3:2); mp 148 °C; ^1^H NMR (400 MHz, DMSO-*d*_6_): δ (ppm) = 8.44 (t, *J* = 6.2 Hz, 1H), 7.36 (m, 4H), 7.22 (t, *J* = 6.2 Hz, 1H), 7.14 (m, 4H), 5.90 (s, 1H), 4.43 (d, *J* = 6.0 Hz, 2H), 4.35 (d, *J* = 6.1 Hz, 2H), 3.73 (s, 3H), 2.04 (s, 3H); ^13^C NMR (100 MHz, DMSO-*d*_6_): δ (ppm) = 166.3, 161.0 (d, *J* = 240.0 Hz), 161.0 (d, *J* = 240.0 Hz), 159.4, 156.7, 147.8, 137.0 (d, *J* = 2.9 Hz), 136.0 (d, *J* = 2.9 Hz), 129.1 (d, *J* = 8.1 Hz), 128.9 (d, *J* = 8.1 Hz), 114.8 (d, *J* = 21.2 Hz), 114.8 (d, *J* = 21.2 Hz), 107.6, 100.2, 52.7, 43.5, 41.5, 19.0; IR (ATR): ν̃ = 3325 (m, ν_N–H_), 3040, 2920 (w, ν_C–H_), 1605 (s, ν_C=O_), 1537 (m, δ_N–H_). ESI-HRMS: calcd for [C_22_H_22_N_3_O_2_F_2_ + H]^+^, 398.1675; found, 398.1671; cpd purity (220 nm): 100%.

### *N*-(4-Fluorobenzyl)-6-[(4-fluorobenzyl)amino]-4-methyl-2-oxo-1,2-dihydropyridine-3-carboxamide (**37**)

*N*-(4-Fluorobenzyl)-6-[(4-fluorobenzyl)amino]-4-methyl-2-oxo-1,2-dihydropyridine-3-carboxamide was a side product in the synthesis of *N*-(4-fluorobenzyl)-6-[(4-fluorobenzyl)amino]-2-methoxy-4-methylnicotinamide. It was separated from the main product by flash chromatography and was further purified by recrystallization (ethanol/water). The title compound was obtained as a colorless solid (108 mg, 0.28 mmol, 22%): *R*_*f*_ = 0.48 (ethyl acetate); mp 196 °C; ^1^H NMR (400 MHz, DMSO-*d*_6_): δ (ppm) = 10.97 (s, 1H), 10.17 (s, 1H), 7.38 (m, 2H), 7.31 (m, 2H), 7.19 (m, 2H), 7.13 (m, 2H), 7.06 (t, *J* = 5.8 Hz, 1H), 5.45 (s, 1H), 4.40 (d, *J* = 6.1 Hz, 2H), 4.38 (d, *J* = 5.8 Hz, 2H) 2.43 (s, 3H); ^13^C NMR (100 MHz, DMSO-*d*_6_): δ (ppm) = 166.3, 162.8, 161.4 (d, *J* = 242.0 Hz), 161.0 (d, *J* = 241.0 Hz), 150.6, 136.5 (d, *J* = 3.0 Hz), 134.3 (d, *J* = 3.0 Hz), 129.2 (d, *J* = 8.2 Hz), 129.1 (d, *J* = 8.0 Hz), 115.3 (d, *J* = 21.4 Hz), 114.9 (d, *J* = 21.0 Hz), 103.7, 91.8, 43.9, 41.1, 23.8 (one signal is missing, perhaps due to peak overlap); IR (ATR): ν̃ = 3337 (m, ν_N–H_), 3035, 2978 (w, ν_C–H_), 1619 (s, ν_C=O_), 1603 (m, δ_N–H_).

### Methyl 4-Amino-2-bromobenzoate (**39**)

Methyl 2-bromo-4-nitrobenzoate (2.00 g, 7.7 mmol) was dissolved in ethyl acetate (100 mL). SnCl_2_ (7.29 g, 38.5 mmol, 5.0 equiv) was added, and the mixture was stirred at 70 °C for 4 h. Afterward, it was cooled to room temperature, and a sat. aq NaHCO_3_ solution (100 mL) was added. The phases were separated, and the aq phase was extracted with ethyl acetate (2 × 100 mL). The combined organic phases were washed with brine, dried over Na_2_SO_4_, and filtered through a pad of silica gel. The silica gel pad was rinsed with additional ethyl acetate, and the combined filtrates were concentrated under reduced pressure to obtain **39** as a colorless solid (1.61 g, 7.0 mmol, 91%): *R*_*f*_ = 0.34 (ethyl acetate/*n*-hexane 1:4); mp 96 °C; ^1^H NMR (400 MHz, DMSO-*d*_6_): δ (ppm) = 7.63 (d, *J* = 8.6 Hz, 1H), 6.86 (d, *J* = 2.2 Hz, 1H), 6.55 (dd, *J* = 8.6, 2.3 Hz, 1H), 6.14 (s, 2H), 3.73 (s, 3H); ^13^C NMR (100 MHz, DMSO-*d*_6_): δ (ppm) = 165.0, 153.4, 133.5, 123.1, 118.0, 115.5, 111.9, 51.5; IR (ATR): ν̃ = 3408, 3320 (m, ν_N–H_), 3082, 2950 (w, ν_C–H_), 1638 (s, ν_C=O_), 1634 (m, δ_N–H_).

### Methyl 2-Bromo-4-[(4-fluorobenzyl)amino]benzoate (**40**)

The synthesis was carried out according to general procedure A. Methyl 4-amino-2-bromobenzoate (1.63 g, 7.1 mmol), 4-fluorobenzaldehyde (758 μL, 7.06 mmol, 1.0 equiv), toluene (50 mL), and 4 Å molecular sieves (5.0 g) were used in the first reaction step. In the second step, the reduction was performed by using 1.19 g of sodium borohydride (35.3 mmol, 5.0 equiv). The purification by silica gel column chromatography (ethyl acetate/*n*-hexane 2:8) afforded **40** as a colorless solid (2.09 g, 6.17 mmol, 87%): *R*_*f*_ = 0.53 (ethyl acetate/*n*-hexane 2:8); mp 80 °C; ^1^H NMR (400 MHz, DMSO-*d*_6_): δ (ppm) = 7.65 (d, *J* = 8.7 Hz, 1H), 7.37 (m, 2H), 7.26 (t, *J* = 6.0 Hz, 1H), 7.17 (m, 2H), 6.87 (d, *J* = 2.4 Hz, 1H), 6.61 (dd, *J* = 8.8, 2.4 Hz, 1H), 4.33 (d, *J* = 5.9 Hz, 2H), 3.73 (s, 3H); ^13^C NMR (100 MHz, DMSO-*d*_6_): δ (ppm) = 165.0, 161.2 (d, *J* = 242.4 Hz), 152.4, 135.0 (d, *J* = 2.9 Hz), 133.3, 129.1 (d, *J* = 8.1 Hz), 123.2, 116.8, 116.1, 115.2 (d, *J* = 21.3 Hz), 110.6, 51.5, 44.9; IR (ATR): ν̃ = 3354 (m, ν_N–H_), 3024, 2946 (w, ν_C–H_), 1678 (s, ν_C=O_), 1585 (m, δ_N–H_).

### Methyl 2-Bromo-4-[(*tert*-butoxycarbonyl)(4-fluorobenzyl)amino]benzoate (**41**)

Methyl 2-bromo-4-[(4-fluorobenzyl)amino]benzoate (2.00 g, 5.9 mmol) was dissolved in DCM (50 mL). TEA (1230 μL, 8.87 mmol, 1.5 equiv), di-*tert*-butyl dicarbonate (1.94 g, 8.9 mmol, 1.5 equiv), and 4-dimethylaminopyridine (72 mg, 0.59 mmol, 0.1 equiv) were added successively. The reaction mixture was stirred at room temperature for 16 h. Afterward, it was concentrated under reduced pressure. The resulting crude residue was directly subjected to silica gel column chromatography (ethyl acetate/*n*-hexane 2:3) to afford **41** as a colorless oil (2.26 g, 5.2 mmol, 87%): *R*_*f*_ = 0.19 (ethyl acetate/*n*-hexane 1:9); ^1^H NMR (400 MHz, DMSO-*d*_6_): δ (ppm) = 7.72 (d, *J* = 8.5 Hz, 1H), 7.67 (d, *J* = 2.1 Hz, 1H), 7.36 (dd, *J* = 8.5, 2.2 Hz, 1H), 7.24 (m, 2H), 7.14 (m, 2H), 4.91 (s, 2H), 3.82 (s, 3H), 1.40 (s, 9H); ^13^C NMR (100 MHz, DMSO-*d*_6_): δ (ppm) = 165.5, 161.3 (d, *J* = 243.0 Hz), 153.2, 145.5, 133.9 (d, *J* = 3.0 Hz), 131.1, 130.6, 129.0 (d, *J* = 8.3 Hz), 128.2, 124.6, 120.1, 115.3 (d, *J* = 21.3 Hz), 81.22, 52.5, 51.2, 27.7; IR (ATR): ν̃ = 2977 (w, ν_C–H_), 1730, 1700 (s, ν_C=O_).

### Methyl 4-[(*tert*-Butoxycarbonyl)(4-fluorobenzyl)amino]-2-[(trimethylsilyl)ethynyl]benzoate (**42**)

Tetrakis(triphenylphosphine)palladium(0) (290 mg, 0.25 mmol, 0.05 equiv) and CuI (96 mg, 0.50 mmol, 0.1 equiv) were set under an argon atmosphere. Methyl 2-bromo-4-[(*tert*-butoxycarbonyl)(4-fluorobenzyl)amino]benzoate (2.20 g, 5.0 mmol) and ethynyl(trimethyl)silane (1070 μL, 7.53 mmol, 1.5 equiv) were dissolved in a mixture of DMF (12 mL) and TEA (8 mL). This solution was degassed using the freeze–pump–thaw method. Afterward, it was added to the mixture of tetrakis(triphenylphosphine)palladium(0) and CuI. The resulting solution was stirred at 60 °C for 4 h. Subsequently, the reaction was quenched by the addition of water (200 mL), and the mixture was extracted with DCM (200 mL). The organic phase was dried over Na_2_SO_4_, filtrated, and concentrated under reduced pressure. The crude residue was purified by silica gel column chromatography (ethyl acetate/*n*-hexane 1:9), which yielded **42** as a dark-red oil (1.73 g, 3.8 mmol, 76%): *R*_*f*_ = 0.39 (ethyl acetate/*n*-hexane 1:9); ^1^H NMR (400 MHz, DMSO-*d*_6_): δ (ppm) = 7.79 (d, *J* = 8.6 Hz, 1H), 7.47 (d, *J* = 2.2 Hz, 1H), 7.36 (dd, *J* = 8.6, 2.3 Hz, 1H), 7.23 (m, 2H), 7.14 (m, 2H), 4.90 (s, 2H), 3.81 (s, 3H), 1.39 (s, 9H), 0.23 (s, 9H); ^13^C NMR (100 MHz, DMSO-*d*_6_): δ (ppm) = 165.4, 161.3 (d, *J* = 242.8 Hz), 153.2, 145.0, 134.0 (d, *J* = 3.0 Hz), 130.6, 130.6, 129.0 (d, *J* = 8.1 Hz), 128.7, 126.0, 122.5, 115.3 (d, *J* = 21.4 Hz), 102.8, 99.6, 80.9, 52.0, 51.2, 27.7, −0.27; IR (ATR): ν̃ = 2958 (w, ν_C–H_), 2157 (w, ν_C≡C_), 1701 (s, ν_C=O_).

### Methyl 4-[(*tert*-Butoxycarbonyl)(4-fluorobenzyl)amino]-2-ethynylbenzoate (**43**)

Methyl 4-[(*tert*-butoxycarbonyl)(4-fluorobenzyl)amino]-2-[(trimethylsilyl)ethynyl]benzoate (1.69 g, 3.7 mmol) and K_2_CO_3_ (512 mg, 3.70 mmol, 1.0 equiv) were dissolved in 20 mL of methanol. The reaction mixture was stirred at room temperature. After 1 h, 100 mL of an ice-cold 1 M aq HCl solution was added, and the resulting mixture was extracted with DCM (100 mL). The organic phase was dried over Na_2_SO_4_, filtrated, and concentrated under reduced pressure. The crude residue was purified by silica gel column chromatography (ethyl acetate/*n*-hexane 1:4), which yielded **43** as a dark-red oil (1.19 g, 3.1 mmol, 84%): *R*_*f*_ = 0.63 (ethyl acetate/*n*-hexane 1:4); ^1^H NMR (400 MHz, DMSO-*d*_6_): δ (ppm) = 7.77 (d, *J* = 8.6 Hz, 1H), 7.48 (d, *J* = 2.3 Hz, 1H), 7.37 (dd, *J* = 8.6, 2.3 Hz, 1H), 7.21 (m, 2H), 7.12 (m, 2H), 4.89 (s, 2H), 4.40 (s, 1H), 3.79 (s, 3H), 1.38 (s, 9H); ^13^C NMR (100 MHz, DMSO-*d*_6_): δ (ppm) = 165.2, 161.3 (d, *J* = 242.8 Hz), 153.2, 145.0, 134.0 (d, *J* = 3.0 Hz), 130.9, 130.5, 128.9 (d, *J* = 8.4 Hz), 128.7, 126.0, 122.2, 115.3 (d, *J* = 21.6 Hz), 85.7, 81.2, 81.0, 52.1, 51.2, 27.7; IR (ATR): ν̃ = 2977 (w, ν_C–H_), 1697 (s, ν_C=O_).

### Methyl 4-[(*tert*-Butoxycarbonyl)(4-fluorobenzyl)amino]-2-ethylbenzoate (**44**)

Methyl 4-[(*tert*-butoxycarbonyl)(4-fluorobenzyl)amino]-2-ethynylbenzoate (1.10 g, 2.9 mmol) was dissolved in methanol (30 mL). Pd/C (10% Pd, 50% water wet, 300 mg) was added, and the suspension was carefully set under a hydrogen atmosphere (balloon pressure) and stirred at 40 °C. After 16 h, the reaction mixture was filtered through a pad of Celite. The filtrate was concentrated under reduced pressure to obtain **44** as a colorless oil (951 mg, 2.46 mmol, 86%): *R*_*f*_ = 0.42 (ethyl acetate/*n*-hexane 1:9); ^1^H NMR (400 MHz, DMSO-*d*_6_): δ (ppm) = 7.70 (d, *J* = 8.4 Hz, 1H), 7.17 (m, 6H), 4.88 (s, 2H), 3.79 (s, 3H), 2.84 (q, *J* = 7.5 Hz, 2H), 1.39 (s, 9H), 1.09 (t, *J* = 7.5 Hz, 3H); ^13^C NMR (100 MHz, DMSO-*d*_6_): δ (ppm) = 166.8, 161.2 (d, *J* = 242.5 Hz), 153.5, 145.8, 145.1, 134.3 (d, *J* = 3.0 Hz), 130.7, 129.0 (d, *J* = 8.2 Hz), 127.2, 125.6, 123.0, 115.2 (d, *J* = 21.3 Hz), 80.5, 51.9, 51.4, 27.8, 26.7, 15.8; IR (ATR): ν̃ = 2975 (w, ν_C–H_), 1698 (s, ν_C=O_).

### 4-[(*tert*-Butoxycarbonyl)(4-fluorobenzyl)amino]-2-ethylbenzoic Acid (**45**)

KOH (512 mg, 9.29 mmol, 4.0 equiv) was dissolved in a mixture of methanol (30 mL) and water (10 mL). Subsequently, methyl 4-[(*tert*-butoxycarbonyl)(4-fluorobenzyl)amino]-2-ethylbenzoate (900 mg, 2.32 mmol) was suspended in the resulting solution. The reaction mixture was stirred at 40 °C for 24 h. After complete conversion, methanol was removed under reduced pressure. The resulting aq solution was cooled to 0 °C and acidified to pH 3–4 with a 2 M aq HCl solution. Afterward, it was extracted with DCM (3 × 100 mL). The combined organic phases were dried over Na_2_SO_4_, filtrated, and concentrated under reduced pressure to obtain **45** as a colorless solid (789 mg, 2.11 mmol, 91%): mp 106 °C; ^1^H NMR (400 MHz, DMSO-*d*_6_): δ (ppm) = 7.71 (d, *J* = 8.4 Hz, 1H), 7.23 (m, 2H), 7.14 (m, 4H), 4.88 (s, 2H), 2.87 (q, *J* = 7.5 Hz, 2H), 1.40 (s, 9H), 1.10 (t, *J* = 7.5 Hz, 3H); ^13^C NMR (100 MHz, DMSO-*d*_6_): δ (ppm) = 168.1, 161.2 (d, *J* = 242.7 Hz), 153.5, 145.7, 144.8, 134.4 (d, *J* = 3.0 Hz), 130.9, 129.0 (d, *J* = 8.2 Hz), 127.2, 126.8, 122.9, 115.2 (d, *J* = 21.4 Hz), 80.5, 51.5, 27.8, 26.8, 15.9; IR (ATR): ν̃ = 2978 (w, ν_C–H_), 3300–2500 (b, ν_O–H_), 1684 (s, ν_C=O_).

### *tert*-Butyl [3-Ethyl-4-(propylcarbamoyl)phenyl](4-fluorobenzyl)carbamate (**46**)

4-[(*tert*-Butoxycarbonyl)(4-fluorobenzyl)amino]-2-ethylbenzoic acid (180 mg, 0.48 mmol), HATU (367 mg, 0.96 mmol, 2.0 equiv), *N*,*N*-diisopropylethylamine (DIPEA) (336 μL, 1.93 mmol, 4.0 equiv), and *n*-propylamine (59 μL, 0.72 mmol, 1.5 equiv) were dissolved in DMF (10 mL) successively. The resulting mixture was stirred at room temperature. After 8 h, the reaction mixture was quenched by the addition of water (100 mL). The resulting suspension was extracted with DCM (100 mL). The organic phase was washed with water (2 × 100 mL), dried over Na_2_SO_4_, filtrated, and concentrated under reduced pressure. The crude residue was purified by silica gel column chromatography (ethyl acetate/*n*-hexane 3:7), which yielded **46** as a colorless oil (156 mg, 0.38 mmol, 78%): *R*_*f*_ = 0.29 (ethyl acetate/*n*-hexane 1:4); ^1^H NMR (400 MHz, DMSO-*d*_6_): δ (ppm) = 8.24 (t, *J* = 5.7 Hz, 1H), 7.14 (m, 7H), 4.84 (s, 2H), 3.15 (td, *J* = 6.9, 5.7 Hz, 2H), 2.64 (q, *J* = 7.5 Hz, 2H), 1.48 (m, 2H), 1.40 (s, 9H), 1.08 (t, *J* = 7.5 Hz, 3H), 0.88 (t, *J* = 7.4 Hz, 3H); ^13^C NMR (100 MHz, DMSO-*d*_6_): δ (ppm) = 168.6, 161.2 (d, *J* = 242.5 Hz), 153.8, 142.5, 142.0, 134.5 (d, *J* = 3.0 Hz), 134.3, 129.0 (d, *J* = 8.3 Hz), 127.4, 126.7, 122.9, 115.2 (d, *J* = 21.3 Hz), 80.2, 51.8, 40.6, 27.9, 25.7, 22.3, 15.7, 11.4; IR (ATR): ν̃ = 3295 (m, ν_N–H_), 2967 (w, ν_C–H_), 1695, 1638 (s, ν_C=O_), 1604 (m, δ_N–H_).

### 2-Ethyl-4-[(4-fluorobenzyl)amino]-*N*-propylbenzamide hydrochloride (**47**)

*tert*-Butyl [3-ethyl-4-(propylcarbamoyl)phenyl](4-fluorobenzyl)carbamate (350 mg, 0.84 mmol) was dissolved in DCM (5 mL) and treated with TFA (5.0 mL, 65.34 mmol, 77.4 equiv). The resulting solution was stirred at room temperature for 6 h. Subsequently, additional DCM (100 mL) was added, and the solution was extracted with a saturated aq NaHCO_3_ solution (100 mL). The organic phase was dried over Na_2_SO_4_, filtrated, and concentrated under reduced pressure. The crude residue was purified by silica gel column chromatography (ethyl acetate/*n*-hexane 3:7), which yielded the product as a colorless oil. To obtain a solid, the hydrochloride salt was formed. For this reason, the residue was dissolved in ethyl acetate. The resulting solution was cooled to 0 °C, and HCl gas was passed through the solution over a period of 30 min to precipitate a hydrochloride salt. The precipitate was filtered off to obtain **47** as a colorless solid (266 mg, 0.76 mmol, 90%): *R*_*f*_ = 0.65 (free base, mobile phase: ethyl acetate/*n*-hexane 3:2); mp decomp.; ^1^H NMR (400 MHz, MeOH-*d*_4_): δ (ppm) = 7.46 (m, 3H), 7.26 (m, 2H), 7.17 (m, 2H), 4.63 (s, 2H), 3.35 (m, 2H), 2.80 (q, *J* = 7.6 Hz, 2H), 1.66 (m, 2H), 1.21 (t, *J* = 7.6 Hz, 3H), 1.01 (t, *J* = 7.4 Hz, 3H); ^13^C NMR (100 MHz, MeOH-*d*_4_): δ (ppm) = 171.7, 165.0 (d, *J* = 248.0 Hz), 146.0, 138.8, 137.8, 133.9 (d, *J* = 8.8 Hz), 130.2, 128.6, 124.6, 121.2, 117.1 (d, *J* = 22.0 Hz), 55.5, 42.8, 27.3, 23.7, 16.0, 12.0; IR (ATR): ν̃ = 3272 (m, ν_N–H_), 2965 (w, ν_C–H_), 1637 (s, ν_C=O_), 1604 (m, δ_N–H_). ESI-HRMS: calcd for [C_19_H_23_N_2_OF + H]^+^, 315.1867; found, 315.1861; cpd purity (220 nm): 100%.

### 4-Methyl-2,6-dimorpholinonicotinonitrile (**53**)

2,6-Dichloro-4-methylnicotinonitrile (1.00 g, 5.4 mmol) was suspended in 2-propanol (100 mL). Morpholine (2790 μL, 14.32 mmol, 6.0 equiv) was added, and the mixture was stirred at 170 °C in a sealed vessel. After 5 h, the mixture was cooled to room temperature and concentrated under reduced pressure. The residue was dissolved in 100 mL of ethyl acetate, and the resulting solution was extracted with water (100 mL). The organic phase was washed with brine and dried over Na_2_SO_4_. 100 mL of *n*-hexane was added to the organic phase, and the mixture was passed through a pad of silica gel. Afterward, the silica gel pad was rinsed with additional 100 mL of an *n*-hexane/ethyl acetate mixture (1:1). The eluates were combined and concentrated under reduced pressure to obtain **53** as a beige solid (1.26 g, 4.4 mmol, 82%): *R*_*f*_ = 0.48 (ethyl acetate/*n*-hexane 1:1); mp 141 °C; ^1^H NMR (400 MHz, DMSO-*d*_6_): δ (ppm) = 6.34 (d, *J* = 0.9 Hz, 1H), 3.69 (m, 4H), 3.65 (m, 4H), 3.55 (m, 4H), 3.49 (m, 4H), 2.28 (d, *J* = 0.7 Hz, 3H); ^13^C NMR (100 MHz, DMSO-*d*_6_): δ (ppm) = 161.4, 157.9, 153.6, 118.5, 100.0, 82.4, 65.9, 65.8, 48.4, 44.3, 20.5; IR (ATR): ν̃ = 2968 (w, ν_C–H_), 2191 (m, ν_C≡N_).

### (4-Methyl-2,6-dimorpholinopyridin-3-yl)methanamine (**54**)

The synthesis was carried out according to general procedure B. 4-Methyl-2,6-dimorpholinonicotinonitrile (250 mg, 0.87 mmol) was used as the reactant. The title compound was obtained as a beige solid (93 mg, 0.32 mmol, 37%): *R*_*f*_ = 0.66 (DCM/MeOH/TEA 9.5:0.5:1); mp 194 °C; ^1^H NMR (400 MHz, DMSO-*d*_6_): δ (ppm) = 7.86 (s, 2H), 6.45 (s, 1H), 3.94 (s, 2H), 3.70 (m, 4H), 3.68 (m, 4H), 3.43 (m, 4H), 2.93 (m, 4H), 2.30 (s, 3H); ^13^C NMR (100 MHz, DMSO-*d*_6_): δ (ppm) = 160.7, 157.7, 150.3, 110.4, 103.7, 66.3, 65.9, 51.3, 44.9, 35.0, 19.6; IR (ATR): ν̃ = 2957 (w, ν_C–H_), 1597 (m, δ_N–H_).

### 4-Fluoro-*N*-[(4-methyl-2,6-dimorpholinopyridin-3-yl)methyl]benzamide (**55a**)

The synthesis was carried out as described for **66**. (4-Methyl-2,6-dimorpholinopyridin-3-yl)methanamine (218 mg, 0.75 mmol), 4-fluorobenzoyl chloride (106 μL, 0.90 mmol, 1.2 equiv), and TEA (208 μL, 1.49 mmol, 2.0 equiv) were used. The purification by silica gel column chromatography (ethyl acetate/*n*-hexane 1:1) and successive recrystallization (methanol/water) afforded **55a** as a colorless solid (243 mg, 0.59 mmol, 79%): *R*_*f*_ = 0.31 (ethyl acetate/*n*-hexane 1:1); mp decomp.; ^1^H NMR (400 MHz, DMSO-*d*_6_): δ (ppm) = 8.40 (t, *J* = 4.2 Hz, 1H), 7.93 (m, 2H), 7.25 (m, 2H), 6.39 (s, 1H), 4.43 (d, *J* = 4.2 Hz, 2H), 3.69 (m, 8H, 15-H), 3.40 (m, 4H), 3.00 (m, 4H), 2.21 (s, 3H); ^13^C NMR (100 MHz, DMSO-*d*_6_): δ (ppm) = 165.3, 163.8 (d, *J* = 248.1 Hz), 160.3, 157.0, 150.5, 130.8 (d, *J* = 2.9 Hz), 130.0 (d, *J* = 8.9 Hz), 115.0 (d, *J* = 21.6 Hz), 112.0, 103.0, 66.3, 66.0, 51.3, 45.2, 37.2, 19.4; IR (ATR): ν̃ = 3261 (m, ν_N–H_), 2957 (w, ν_C–H_), 1624 (s, ν_C=O_), 1598 (s, δ_N–H_); ESI-HRMS: calcd for [C_22_H_27_N_4_O_3_F + H]^+^, 415.2140; found, 415.2123; cpd purity (220 nm): 100%.

### 2-(3,5-Difluorophenyl)-*N*-[(4-methyl-2,6-dimorpholinopyridin-3-yl)methyl]acetamide (**55b**)

The synthesis was carried out as described for **61**. (4-Methyl-2,6-dimorpholinopyridin-3-yl)methanamine (93 mg 0.32 mmol), 2-(3,5-difluorophenyl)acetic acid (66 mg, 0.38 mmol, 1.2 equiv), and CDI (124 mg, 0.76 mmol, 2.4 equiv) were used. The purification by silica gel column chromatography (ethyl acetate/*n*-hexane 1:1) and successive recrystallization (methanol/water) afforded **55b** as a colorless solid (119 mg, 0.27 mmol, 84%): *R*_*f*_ = 0.35 (ethyl acetate/*n*-hexane 1:1); mp 239 °C; ^1^H NMR (400 MHz, DMSO-*d*_6_): δ (ppm) = 8.08 (t, *J* = 4.3 Hz, 1H), 7.09 (m, 1H), 6.96 (m, 2H), 6.38 (s, 1H), 4.18 (d, *J* = 4.2 Hz, 2H), 3.67 (m, 4H), 3.60 (m, 4H), 3.46 (s, 2H), 3.38 (m, 4H), 2.91 (m, 4H), 2.15 (s, 3H); ^13^C NMR (100 MHz, DMSO-*d*_6_): δ (ppm) = 168.9, 162.1 (dd, *J* = 245.5, 13.5 Hz), 160.3, 157.1, 150.5, 140.9 (t, *J* = 9.9 Hz), 112.0 (dd, *J* = 18.3, 6.5 Hz), 111.8, 102.9, 101.8 (t, *J* = 25.7 Hz), 66.2, 65.9, 51.3, 45.2, 41.6, 36.5, 19.2; IR (ATR): ν̃ = 3293 (m, ν_N–H_), 2969 (w, ν_C–H_), 1632 (s, ν_C=O_), 1594 (s, δ_N–H_); ESI-HRMS: calcd for [C_23_H_28_N_4_O_3_F_2_ + H]^+^, 447.2202; found, 447.2199; cpd purity (220 nm): 99%.

### 4,4′-(4-Methylpyridine-2,6-diyl)dimorpholine (**56**)

4,4′-(4-Methylpyridine-2,6-diyl)dimorpholine was a side product in the synthesis of (4-methyl-2,6-dimorpholinopyridin-3-yl)methanamine. It was obtained as a colorless solid by concentrating the ethyl acetate filtrate under reduced pressure (88 mg, 0.33 mmol, 38%): *R*_*f*_ = 0.65 (ethyl acetate/*n*-hexane 1:1); mp 118 °C; ^1^H NMR (400 MHz, DMSO-*d*_6_): δ (ppm) = 5.96 (s, 2H), 3.66 (m, 8H), 3.34 (m, 8H), 2.14 (s, 3H); ^13^C NMR (100 MHz, DMSO-*d*_6_): δ (ppm) = 158.1, 149.3, 97.0, 66.0, 45.2, 21.4; IR (ATR): ν̃ = 3075, 2983 (w, ν_C–H_).

### 6-Amino-2-chloro-4-methylnicotinonitrile (**57**)

6-Amino-2-chloro-4-methylnicotinonitrile is a side product in the synthesis of 2-amino-6-chloro-4-methylnicotinonitrile. It was separated from the main product by flash chromatography. The title compound was obtained as a colorless solid (403 mg, 2.41 mmol, 14%): *R*_*f*_ = 0.49 (ethyl acetate/*n*-hexane 1:3); mp 240 °C; ^1^H NMR (400 MHz, DMSO-*d*_6_): δ (ppm) = 7.37 (s, 2H), 6.33 (s, 1H), 2.29 (s, 3H); ^13^C NMR (100 MHz, DMSO-*d*_6_): δ (ppm) = 160.9, 152.7, 151.9, 116.1, 106.6, 95.1, 19.9; IR (ATR): ν̃ = 3388, 3319 (m, ν_N–H_), 2218 (m, ν_C≡N_), 1644 (m, δ_N–H_).

### 2-Amino-6-chloro-4-methylnicotinonitrile (**58**)

2,6-Dichloro-4-methylnicotinic acid (2.00 g, 10.7 mmol) was suspended in a saturated solution of ammonia in 2-propanol (100 mL). The mixture was stirred in a sealed vessel at 70 °C. After 9 h, the reaction mixture was cooled to room temperature and concentrated under reduced pressure. The crude residue was purified by flash chromatography (ethyl acetate/*n*-hexane, 25–50% ethyl acetate) to obtain **58** as a slightly yellow solid (811 mg, 4.84 mmol, 20%): *R*_*f*_ = 0.44 (ethyl acetate/*n*-hexane 1:1); mp 255 °C; ^1^H NMR (400 MHz, DMSO-*d*_6_): δ (ppm) = 7.26 (s, 2H), 6.68 (s, 1H), 2.32 (s, 3H); ^13^C NMR (100 MHz, DMSO-*d*_6_): δ (ppm) = 160.2, 156.0, 152.8, 115.5, 112.3, 88.9, 19.7; IR (ATR): ν̃ = 3378, 3313 (m, ν_N–H_), 2219 (m, ν_C≡N_), 1642 (m, δ_N–H_).

### 2-Amino-4-methyl-6-morpholinonicotinonitrile (**59**)

2-Amino-6-chloro-4-methylnicotinonitrile (800 mg, 4.77 mmol) was suspended in 2-propanol (100 mL). Morpholine (1240 μL, 14.32 mmol, 3.0 equiv) was added, and the mixture was stirred at 170 °C in a sealed vessel. After 3 h, the mixture was cooled to room temperature and filtered through a pad of Celite. The filtrate was concentrated under reduced pressure to a final volume of about 20 mL. Water was added to form a precipitate, which was filtered off to obtain **59** as an off-white solid (925 mg, 4.26 mmol, 89%): *R*_*f*_ = 0.50 (ethyl acetate/*n*-hexane 1:1); mp 160 °C; ^1^H NMR (400 MHz, DMSO-*d*_6_): δ (ppm) = 6.30 (s, 2H), 6.06 (s, 1H), 3.62 (m, 4H), 3.51 (m, 4H), 2.19 (s, 3H); ^13^C NMR (100 MHz, DMSO-*d*_6_): δ (ppm) = 160.2, 159.0, 152.0, 118.1, 96.4, 77.9, 65.7, 44.3, 20.2; IR (ATR): ν̃ = 3416, 3337 (m, ν_N–H_), 2988 (w, ν_C–H_), 2202 (m, ν_C≡N_), 1643 (m, δ_N–H_).

### 3-(Aminomethyl)-4-methyl-6-morpholinopyridin-2-amine (**60**)

The synthesis was carried out according to general procedure B. 2-Amino-4-methyl-6-morpholinonicotinonitrile (250 mg, 1.2 mmol) was used as the reactant. The title compound was obtained as a beige solid (86 mg, 0.39 mmol, 34%), used for the following reaction without any further purification and characterization.

### *N*-[(2-Amino-4-methyl-6-morpholinopyridin-3-yl)methyl]-2-(3,5-difluorophenyl)acetamide (**61**)

2-(3,5-Difluorophenyl)acetic acid (80 mg, 0.46 mmol, 1.2 equiv) was dissolved in THF (20 mL). CDI (151 mg, 0.93 mmol, 2.4 equiv) was added in one portion, and the mixture was stirred at room temperature. After 16 h, a solution of (4-methyl-2,6-dimorpholinopyridin-3-yl)methanamine (86 mg, 0.39 mmol) in THF (10 mL) was added, and the reaction mixture was continued to stir at room temperature for additional 2 h. Afterward, ethyl acetate (100 mL) was added. The resulting solution was extracted with water (3 × 100 mL), washed with brine, dried over Na_2_SO_4_, filtrated, and concentrated under reduced pressure. The crude residue was purified by silica gel column chromatography (ethyl acetate/*n*-hexane 4:1) and successive recrystallization (methanol/water) to obtain **61** as a colorless solid (101 mg, 0.27 mmol, 69%): *R*_*f*_ = 0.21 (ethyl acetate/*n*-hexane 7:3); mp 234 °C; ^1^H NMR (400 MHz, DMSO-*d*_6_): δ (ppm) = 8.38 (t, *J* = 5.7 Hz, 1H), 7.08 (m, 1H), 6.97 (m, 2H), 5.85 (s, 1H), 5.56 (s, 2H), 4.08 (d, *J* = 5.7 Hz, 2H), 3.63 (m, 4H), 3.48 (s, 2H), 3.29 (m, 4H), 2.17 (s, 3H); ^13^C NMR (100 MHz, DMSO-*d*_6_): δ (ppm) = 169.7, 162.1 (dd, *J* = 245.4 Hz, 13.5 Hz), 157.4, 156.8, 148.0, 140.6 (t, *J* = 9.9 Hz), 112.2 (dd, *J* = 18.2 Hz, 6.4 Hz), 104.7, 101.9 (t, *J* = 25.8 Hz), 97.0, 66.0, 45.3, 41.3 (t, *J* = 2.2 Hz), 34.9, 19.4; IR (ATR): ν̃ = 3480, 3364, 3295 (m, ν_N–H_), 3072, 2966 (w, ν_C–H_), 1634 (s, ν_C=O_), 1620 (m, δ_N–H_); ESI-HRMS: calcd for [C_19_H_22_N_4_O_2_F_2_ + H]^+^, 377.1784; found, 377.1775; cpd purity (220 nm): 100%.

### 3,4-Dimethyl-6-morpholinopyridin-2-amine (**62**)

2-Amino-4-methyl-6-morpholinonicotinonitrile (326 mg, 1.50 mmol) was dissolved in methanol (40 mL), and Pd/C (10% Pd, 50% water wet, 100 mg) was added. The suspension was carefully set under a hydrogen atmosphere (balloon pressure) and stirred at 40 °C. After 48 h, the reaction mixture was filtered through a pad of Celite, and the filtrate was concentrated under reduced pressure. The residue was redissolved in ethyl acetate and filtered through a pad of silica gel. The silica gel pad was rinsed with additional ethyl acetate. Afterward, the filtrate was concentrated under reduced pressure again, and the crude residue was recrystallized (methanol/water) to obtain the title product as a colorless solid (245 mg, 1.8 mmol, 79%): *R*_*f*_ = 0.33 (ethyl acetate/*n*-hexane 1:1); mp 125 °C; ^1^H NMR (400 MHz, DMSO-*d*_6_): δ (ppm) = 5.83 (s, 1H), 5.21 (s, 2H), 3.64 (m, 4H), 3.24 (m, 4H), 2.07 (s, 3H), 1.86 (s, 3H); ^13^C NMR (100 MHz, DMSO-*d*_6_): δ (ppm) = 156.3, 156.1, 146.3, 103.5, 97.1, 66.1, 45.6, 20.0, 11.6; IR (ATR): ν̃ = 3464, 3367 (m, ν_N–H_), 2962 (w, ν_C–H_), 1605 (m, δ_N–H_).

### 6-Amino-4-methyl-2-morpholinonicotinonitrile (**63**)

The synthesis was carried out as described for **59**. 6-Amino-2-chloro-4-methylnicotinonitrile (400 mg, 2.39 mmol) and morpholine (618 μL, 7.16 mmol, 3.0 equiv) were used. **63** was obtained as an off-white solid (455 mg, 2.09 mmol, 87%): *R*_*f*_ = 0.36 (ethyl acetate/*n*-hexane 1:1); mp 135 °C; ^1^H NMR (400 MHz, DMSO-*d*_6_): δ (ppm) = 6.66 (s, 2H), 5.90 (s, 1H), 3.67 (m, 4H), 3.44 (m, 4H), 2.19 (s, 3H); ^13^C NMR (100 MHz, DMSO-*d*_6_): δ (ppm) = 162.9, 160.0, 152.5, 118.9, 100.5, 81.7, 66.0, 48.5, 20.1; IR (ATR): ν̃ = 3446, 3342 (m, ν_N–H_), 2965 (w, ν_C–H_), 2200 (m, ν_C≡N_), 1640 (m, δ_N–H_).

### 6-[(4-Fluorobenzyl)amino]-4-methyl-2-morpholinonicotinonitrile (**64**)

6-Amino-4-methyl-2-morpholinonicotinonitrile (500 mg, 2.29 mmol) and 4-fluorobenzaldehyde (266 μL, 2.52 mmol, 1.1 equiv) were dissolved in DCM (30 mL) and stirred at room temperature. After 16 h, the reaction mixture was concentrated under reduced pressure. The residue was dissolved in methanol (30 mL), NaBH_3_CN (1.15 g, 18.3 mmol, 8.0 equiv) was added in portions over a period of 1 h, and the reaction mixture was continued to stir at room temperature. After 24 h, the reaction mixture was quenched by the addition of 100 mL of water. The mixture was extracted with ethyl acetate (100 mL). The organic phase was washed with brine, dried over Na_2_SO_4_, filtrated, and concentrated under reduced pressure. The crude residue was purified by silica gel column chromatography (ethyl acetate/*n*-hexane 1:3) to obtain **64** as a colorless solid (311 mg, 0.95 mmol, 42%): *R*_*f*_ = 0.70 (ethyl acetate/*n*-hexane 1:1); mp 100 °C; ^1^H NMR (400 MHz, DMSO-*d*_6_): δ (ppm) = 7.82 (s, 1H), 7.32 (m, 2H), 7.13 (m, 2H), 5.98 (s, 1H), 4.45 (d, *J* = 5.9 Hz, 2H), 3.63 (m, 4H), 3.45 (m, 4H), 2.19 (s, 3H); ^13^C NMR (100 MHz, DMSO-*d*_6_): δ (ppm) = 162.3, 161.1 (d, *J* = 241.0 Hz), 158.2, 149.7, 136.2, 129.1 (d, *J* = 8.1 Hz), 118.9, 114.9 (d, *J* = 21.1 Hz), 101.0, 87.3, 65.9, 48.3, 41.8, 20.1; IR (ATR): ν̃ = 3408 (m, ν_N–H_), 2955 (w, ν_C–H_), 2199 (m, ν_C≡N_), 1592 (s, δ_N–H_).

### 5-(Aminomethyl)-*N*-(4-fluorobenzyl)-4-methyl-6-morpholinopyridin-2-amine (**65**)

The synthesis was carried out according to general procedure B. 6-[(4-Fluorobenzyl)amino]-4-methyl-2-morpholinonicotinonitrile (500 mg, 1.54 mmol) was used as the reactant. The title compound was obtained as a beige solid (265 mg, 0.80 mmol, 52%), used for the following reaction without any further purification and characterization.

### Isobutyl ({6-[(4-Fluorobenzyl)amino]-4-methyl-2-morpholinopyridin-3-yl}methyl)carbamate (**66**)

5-(Aminomethyl)-*N*-(4-fluorobenzyl)-4-methyl-6-morpholinopyridin-2-amine (265 mg, 0.80 mmol) was dissolved in DCM (20 mL), and TEA (224 μL, 1.60 mmol, 2.0 equiv) was added in one portion. The reaction mixture was cooled to 0 °C, and a solution of isobutyl chloroformate (125 μL, 0.96 mmol, 1.2 equiv) in DCM (10 mL) was added dropwise over a period of 30 min. Cooling was removed, and the reaction mixture was stirred at room temperature. After 16 h, additional DCM (70 mL) was added, and the solution was extracted with water (3 × 100 mL). The organic phase was dried over Na_2_SO_4_, filtrated, and concentrated under reduced pressure. The resulting crude residue was purified by silica gel column chromatography (ethyl acetate/*n*-hexane 1:1) and successive recrystallization (methanol/water) to obtain **66** as a colorless solid (244 mg, 0.57 mmol, 71%): *R*_*f*_ = 0.62 (ethyl acetate/*n*-hexane 1:1); mp 129 °C; ^1^H NMR (400 MHz, DMSO-*d*_6_): δ (ppm) = 7.34 (m, 2H), 7.10 (m, 2H), 7.06 (s, 1H), 6.85 (t, *J* = 6.2 Hz, 1H), 6.02 (s, 1H), 4.40 (d, *J* = 6.1 Hz, 2H), 4.12 (d, *J* = 4.6 Hz, 2H), 3.73 (d, *J* = 6.7 Hz, 2H), 3.65 (m, 4H), 2.89 (m, 4H), 2.10 (s, 3H), 1.80 (m, 1H), 0.85 (d, *J* = 6.7 Hz, 6H); ^13^C NMR (100 MHz, DMSO-*d*_6_): δ (ppm) = 160.9 (d, *J* = 240.0 Hz), 160.6, 156.3, 149.5, 137.4, 137.4, 129.1 (d, *J* = 7.9 Hz), 114.7 (d, *J* = 21.2 Hz), 110.7, 103.7, 69.5, 66.4, 51.4, 43.4, 37.8, 27.7, 19.0, 18.8; IR (ATR): ν̃ = 3314 (m, ν_N–H_), 2957 (w, ν_C–H_), 1681 (s, ν_C=O_), 1607 (m, δ_N–H_); ESI-HRMS: calcd for [C_23_H_31_N_4_O_3_F + H]^+^, 431.2453; found, 431.2438; cpd purity (220 nm): 100%.

### log *D*_7.4_ Estimation

The log *D*_7.4_ values were estimated using a common HPLC-based method in accordance with guideline OPPTS 830.7570 of the United States Environmental Protection Agency (EPA). The HPLC measurements were performed with a Phenomenex Luna 5 μm Phenyl-Hexyl 100 Å column (150 × 4.6 mm). A mixture of methanol (75%) and Tris/HCl buffer (25%) at pH 7.4 was used as the mobile phase at a flow rate of 1.0 mL/min. First, the capacity factors of seven reference substances recommended in the guideline referenced above (acetophenone, benzene, ethyl benzoate, benzophenone, phenyl benzoate, diphenyl ether, and bibenzyl), which cover the required log *D*_7.4_ range of 1.7–4.8, were determined from their retention times. Uracil was used to measure the unretained peak time. To obtain a calibration function, the logarithm of the capacity factors for the reference substances was then plotted against the corresponding log *P* values taken from the mentioned guideline, which are equivalent to the log *D*_7.4_ values for non-ionizable compounds like the reference substances used for calibration. With this calibration function, it was possible to estimate the log *D*_7.4_ value from the corresponding retention time for each compound to be tested. For the measurement, 2 mg of the reference substances was each dissolved in 1 mL of methanol. A reference mixture was then obtained by combining 50 μL of each reference solution. The reference mixture was measured before and after the substances to be tested. The mean value from both measurements was used for the calibration line. The substances to be tested were injected as a solution in methanol (2 mg/mL), and the retention time was determined as the mean value from two measurements.

### K_V_7.2/3 Channel Opening Assay

The cell culture of the K_V_7.2/3 transfected HEK293 cells (HEK293 KCNQ2/3 cells from SB Drug Discovery, Glasgow, UK) used for the assay and the processing of the obtained data were performed as described in earlier work.^27,29^ Briefly, 60,000 cells/well were seeded into 96-well microtiter plates for fluorescence-based assays (4titude Vision Plate Black 96-Well microtiter plates from ThermoScientic) and incubated for 24 h. After incubation, the medium was changed to 200 μL/well loading buffer containing 5 mmol/L probenecid and incubated for 1 h at room temperature. Subsequently, the test compounds dissolved in DMSO were added to the wells to the desired concentrations and further incubated for 30 min. As a control, the loading buffer with a DMSO concentration corresponding to the substance samples (1% (V/V)) was used. Fluorescence measurements were performed on the Infinite F200 Pro plate reader (Tecan) set at extinction/emission wavelengths of 485 and 535 nm, respectively. After measuring the background fluorescence (baseline) for 20 s, a stimulus buffer (25 mmol/L K^+^, 15 mmol/L Tl^+^) was added just before the fluorescence of each well was measured.

The calculations to obtain dose–response curves were performed in the same manner as in previous investigations.^27,29^ Briefly, the fluorescence intensity was normalized with the baseline signal (*F*/*F*_0_) at each time of signal acquisition. To obtain the corrected negative control, a value of 1 was subtracted from the normalized value of the negative control. Subsequently, the value of the corrected negative control was subtracted from the maximum (corr. *F*/*F*_0_) value of a compound in a defined concentration (corr. Δ*F*/*F*_0_) and then plotted against the logarithmic concentration. The EC_50_ value was calculated as relative values with GraphPad Prism 6 (La Jolla, CA, USA), that is, the inflection points of the sigmoidal curves. The E_max_ value, the maximum response, indicates the intrinsic activity of a compound. This value was determined relative to flupirtine (**1**), where the maximum corr. Δ*F*/*F*_0_ value of flupirtine (**1**) was defined as 100%. The EC_50_ and *E*_max_ value of a compound is the mean of at least three independent experiments ± standard deviation (SD).

### Hepatic Cell Viability Assay

The culturing of the mouse liver TAMH and human liver cancer HEP-G2 cell lines and the MTT assay used to evaluate cell viability were carried out as previously described elsewhere in detail.^27,29^ Briefly, 20,000 cells/well of the TAMH cell line, grown in a serum-free DMEM/F12 medium and supplemented with 5% PANEXIN NTA, 10 mM nicotinamide and 10 μg/mL gentamicin sulfate, and 15,000 cells/well of the HEP-G2 cell line, grown in RPMI 1640 (PAN Biotech), supplemented with 10% heat-inactivated fetal bovine serum and 1% penicillin/streptomycin, were seeded into 96-well microtiter plates and incubated at 37 °C in 5% CO_2_ atmosphere for 24 h. Compounds, dissolved in DMSO, were serially diluted into the respective culture medium to give a 1% (v/v) DMSO solution in five to nine concentrations of the compound. The medium in the wells was then replaced with the medium containing the compounds. Control wells contained an equivalent number of cells and 1% (v/v) DMSO. Wells without cells were used to determine the background optical density (OD) and were treated as the control wells. After 24 or 48 h of incubation with the compound, the respective medium was replaced with a fresh medium supplemented with 10% (v/v) of a 2.5 mg/mL solution of MTT. The plates were incubated for 4 h. Then, the culture medium was carefully aspirated off and 50 μL of DMSO was added to each well to dissolve the formazan crystals. The ODs of each well were measured at λ = 570 nm with a SpectraMax 190 microplate reader.

For determination of cell viability, the ODs of the test compound (T) and control (C) wells were corrected by subtracting the bank value. The calculated T/C_corr._ values were plotted against the log(concentration). The LD_50_ and LD_25_ values were determined by using GraphPad Prism 6. After interpolation of a sigmoidal standard curve, the LD_50_ and LD_25_ values accounted for 50 or 75% of viability, respectively. If even the highest tested concentration of a compound did not reduce cell viability to 75%, the LD_25_ was reported as higher than the highest tested concentration. The values are given as the mean with SD of at least three independent experiments.

### Molecular modeling

All calculations were performed with Maestro and included applications of the Schrödinger release 2020-4 (Maestro, Schrödinger, LLC, New York, NY, 2021). Desmond v6.4 with CUDA support was used for all molecular dynamics simulations.^51^

### Structure Preparation

The cryo-EM structure of the homotetrameric K_V_7.2 potassium channel with retigabine (**2**) (RCSB PDB 7CR2)^50^ was prepared by the Protein Preparation Wizard.^52^ Missing side chains and loops were replaced by Prime,^53,54^ followed by a restraint minimization to a heavy atom root-mean-square deviation value of 0.3 Å using OPLS3e force field parameters.^55^ Both chains B and C were rebuilt to the corresponding K_V_7.3 sequence by homology modeling (energy-based) within the Multiple Sequence Viewer application to obtain the heterotetrameric K_V_7.2/3 structure.

### Molecular Docking

The box center and size for grid generation were estimated by the bound retigabine (**2**) of the previously generated homology model. Glide^56^ molecular docking calculations of **28b** and **36b** were performed using an induced fit approach with an extended sampling protocol. In total, 80 protein models with optimized side chains were generated, while an implicit membrane was placed on the transmembrane helices. No further constraints were applied, and the results were visually inspected.

### T-REMD Simulation

Compounds **26b** and **36b** were solvated in a cubic box of TIP3P water^57^ with a buffer of 1 nm to the solute. A temperature replica-exchange molecular dynamics simulation^58^ was performed for each molecule by using eight replicas on a quadratically distributed temperature range between 310 and 350 K. Prior to the replica-exchange simulations, each system was minimized for 100 ps, and the standard relaxation protocol was performed to equilibrate them to the desired baseline temperature and pressure. Exchange attempts were performed every 1.2 ps, and snapshots were written in 5 ps intervals. In total, 10,000 structures were obtained for each compound after a 50 ns simulation time in the *NVT* ensemble. The time step was set to 2 fs, and the temperature was maintained using a Nosé–Hoover thermostat.^59^ Long-range electrostatics are described by the particle mesh Ewald method, while the short-range interaction cutoffs were set to 1 nm with a 0.1 nm switching function.

## Supporting Information Available

^1^H and ^13^C NMR spectra, HMBC spectra used for the discrimination of regioisomers **57** and **58**, assignment of NMR signals, and data used for log *D*_7.4_ estimation (PDF)
