# Supplementary material for: Modifications of the Triaminoaryl Metabophore of Flupirtine and Retigabine Aimed at Avoiding Quinone Diimine Formation
Source: ACS Omega. 2022 Feb 25;7(9):7989–8012. doi: 10.1021/acsomega.1c07103 (PMC8908504; doi:10.1021/acsomega.1c07103)
Supplement: Supplementary file 1 — ao1c07103_si_001.pdf [file ao1c07103_si_001.pdf]

Supporting Information

Modifications of the triaminoaryl metabophore of flupirtine and retigabine aimed at avoiding quinone diimine formation

Konrad W. Wurm,[a] Frieda-Marie Bartz,[a] Lukas Schulig,[a] Anja Bodtke,[a] Patrick J. Bednarski[a] and Andreas Link*[a]

[a] Konrad W. Wurm, Frieda-Marie Bartz, Dr. L. Schulig, Dr. A. Bodtke, Prof. Dr. P. J. Bednarski, Prof. Dr. A. Link
Institute of Pharmacy
University of Greifswald
Friedrich-Ludwig-Jahn-Str. 17, 17489 Greifswald, Germany
Fax: (+49) (0)3834 4204895
E-mail: link@uni-greifswald.de

Table of contents

1 ^{1}H- and ^{13}C-NMR spectra 2
2 Assignment of NMR signals 53
3 Log$D_{7.4}$ estimation 71
4 Analytical discrimination of regioisomers 57 and 58 75

1 ^{1}H- and ^{13}C-NMR spectra

2-(4-Methyl-3-nitrophenyl)isoindoline-1,3-dione (**10**)

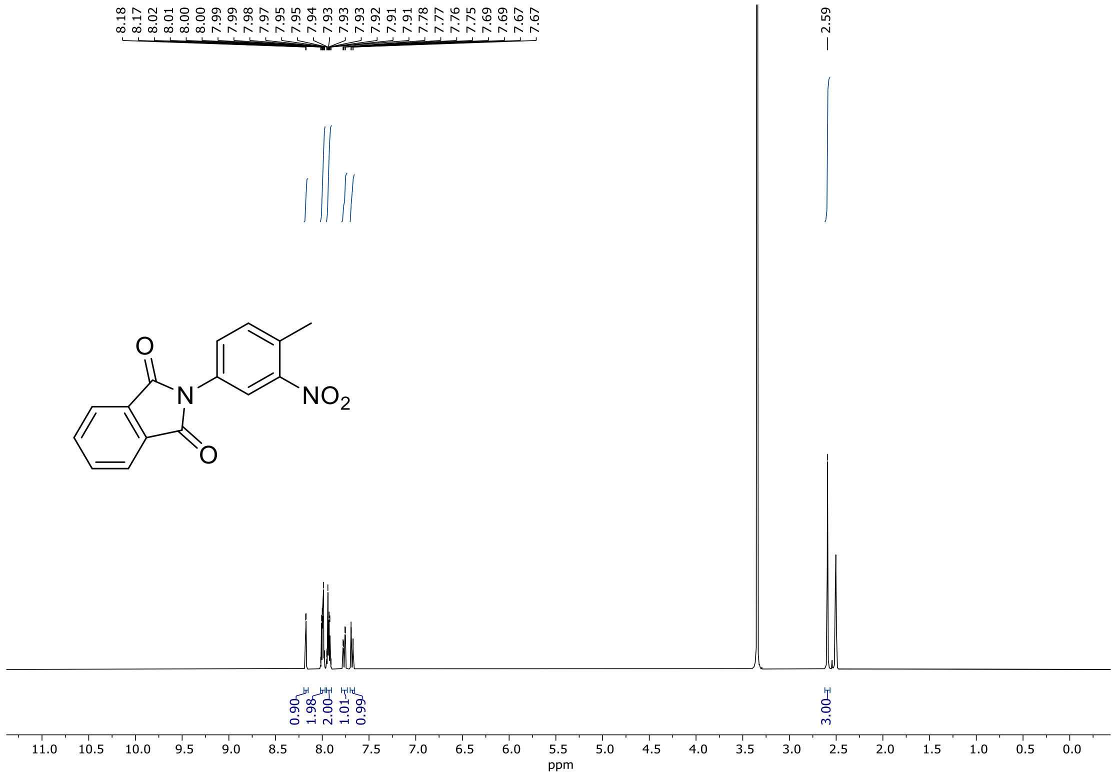

Figure S1: ^{1}H-NMR spectrum of compound **10**.

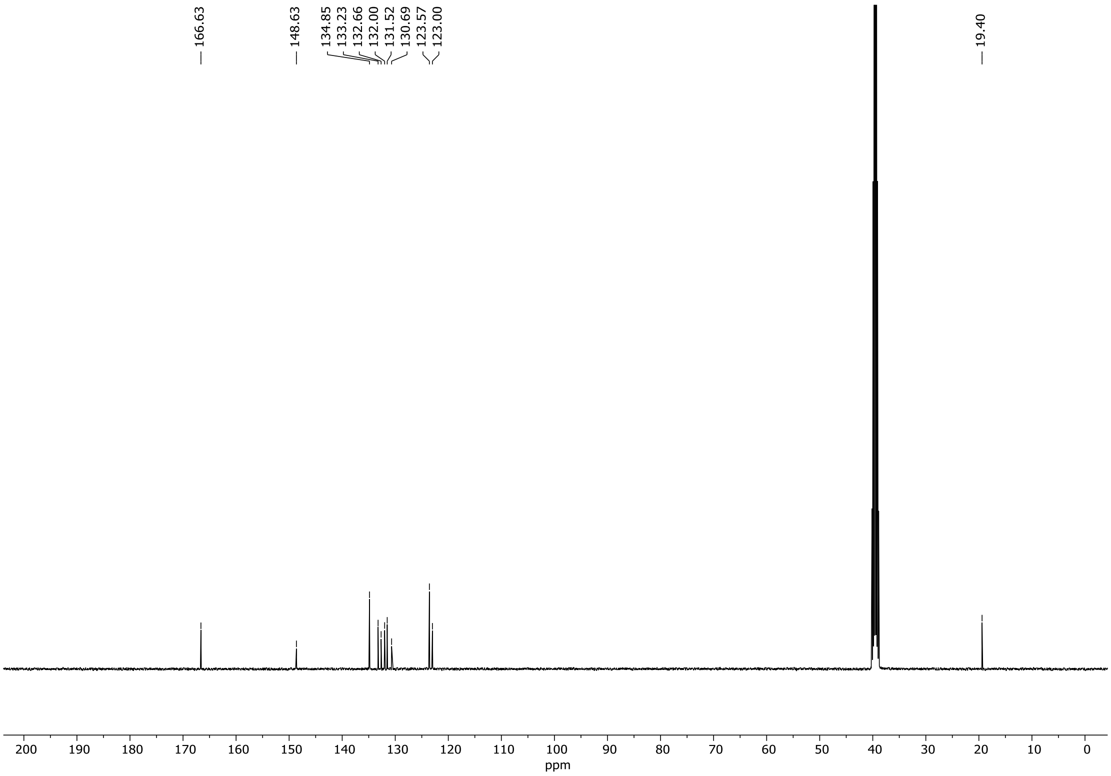

Figure S2: ^{13}C-NMR spectrum of compound **10**.

N-(4-methyl-3-nitrophenyl)acetamide (**12**)

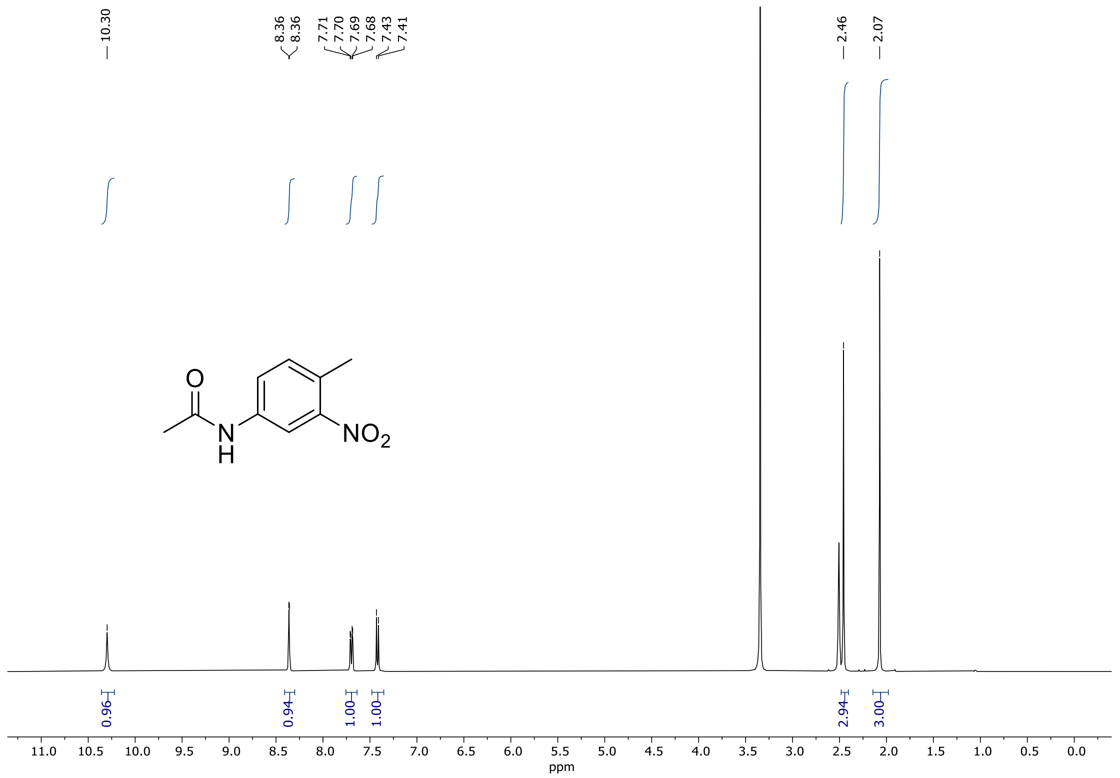

Figure S3: ^{1}H-NMR spectrum of compound **12**.

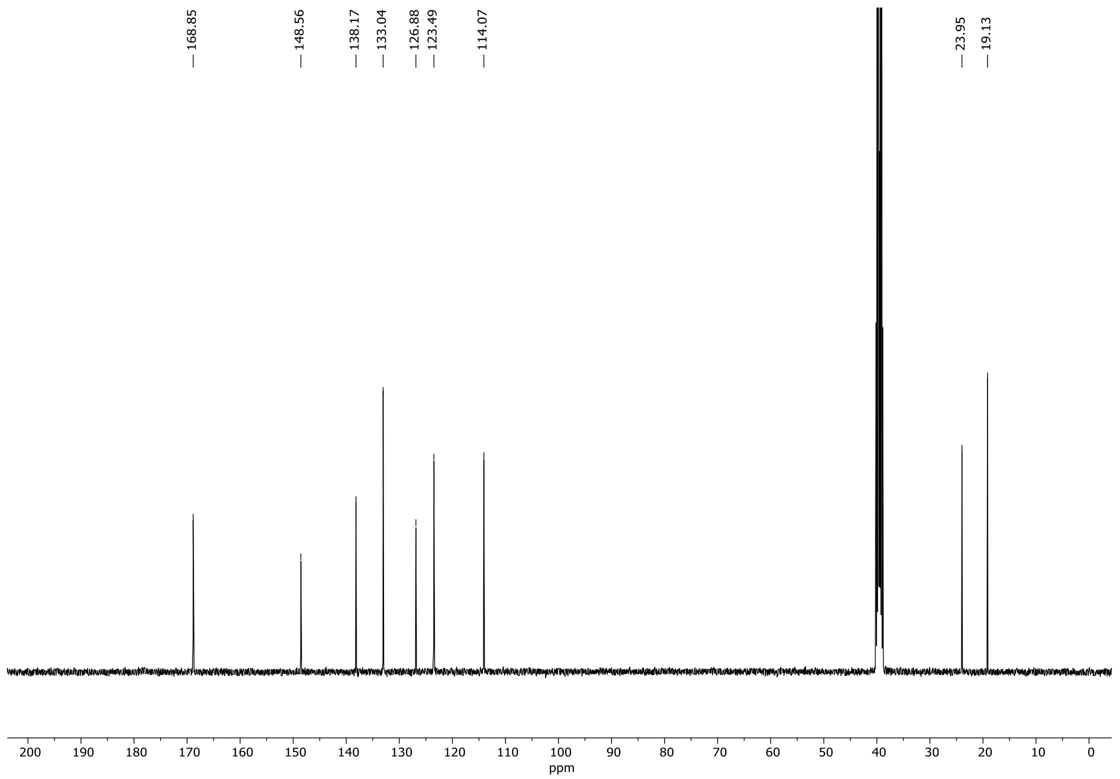

Figure S4: ^{13}C-NMR spectrum of compound **12**.

4-Acetamido-2-nitrobenzoic acid (**13**)

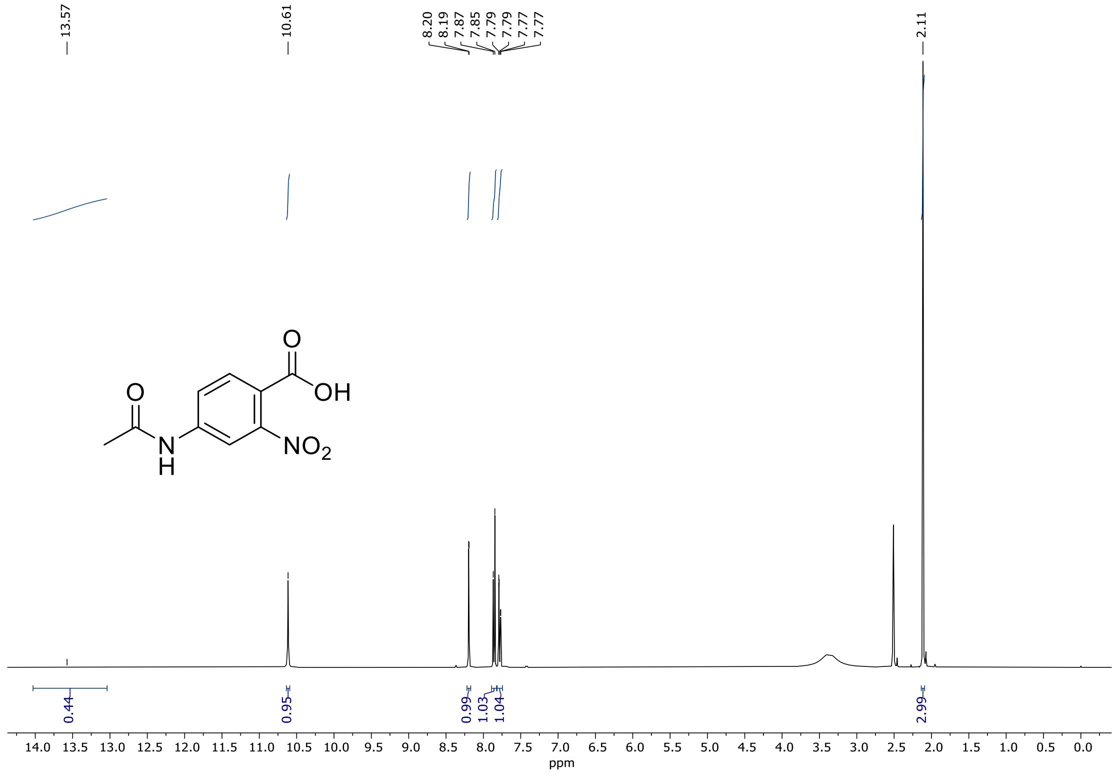

Figure S5: ^{1}H-NMR spectrum of compound **13**.

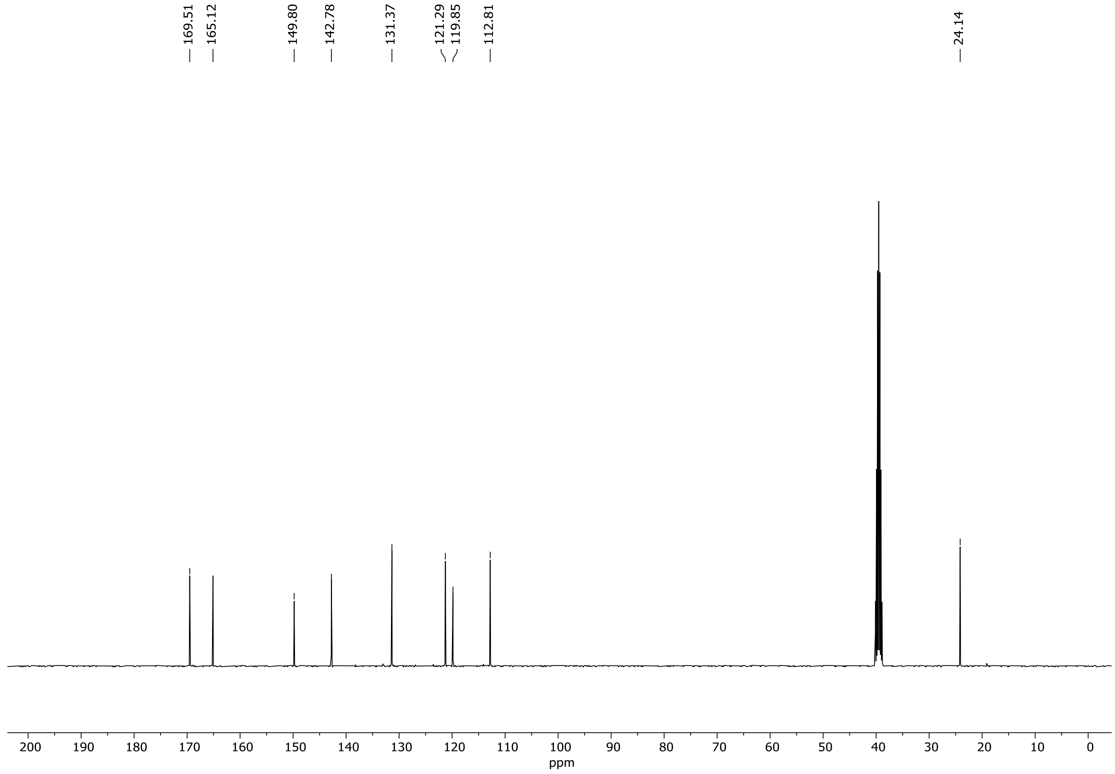

Figure S6: ^{13}C-NMR spectrum of compound **13**.

4-Acetamido-*N*-isobutyl-2-nitrobenzamide (**14**)

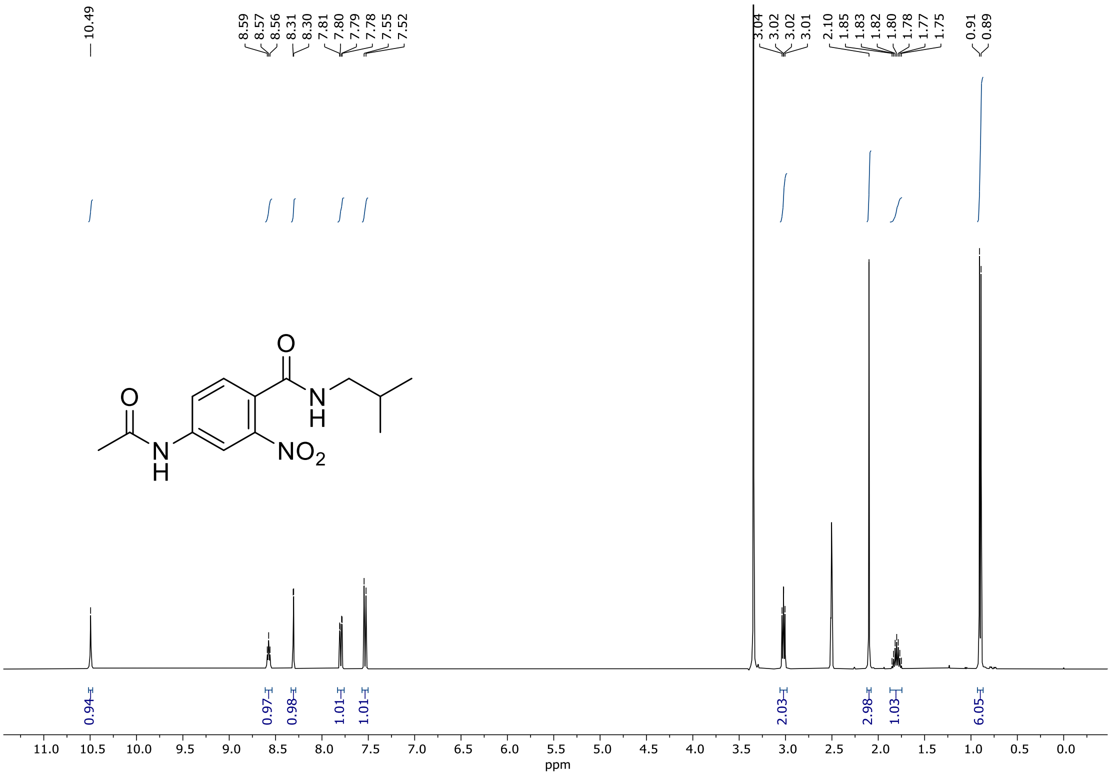

Figure S7: ^{1}H-NMR spectrum of compound **14**.

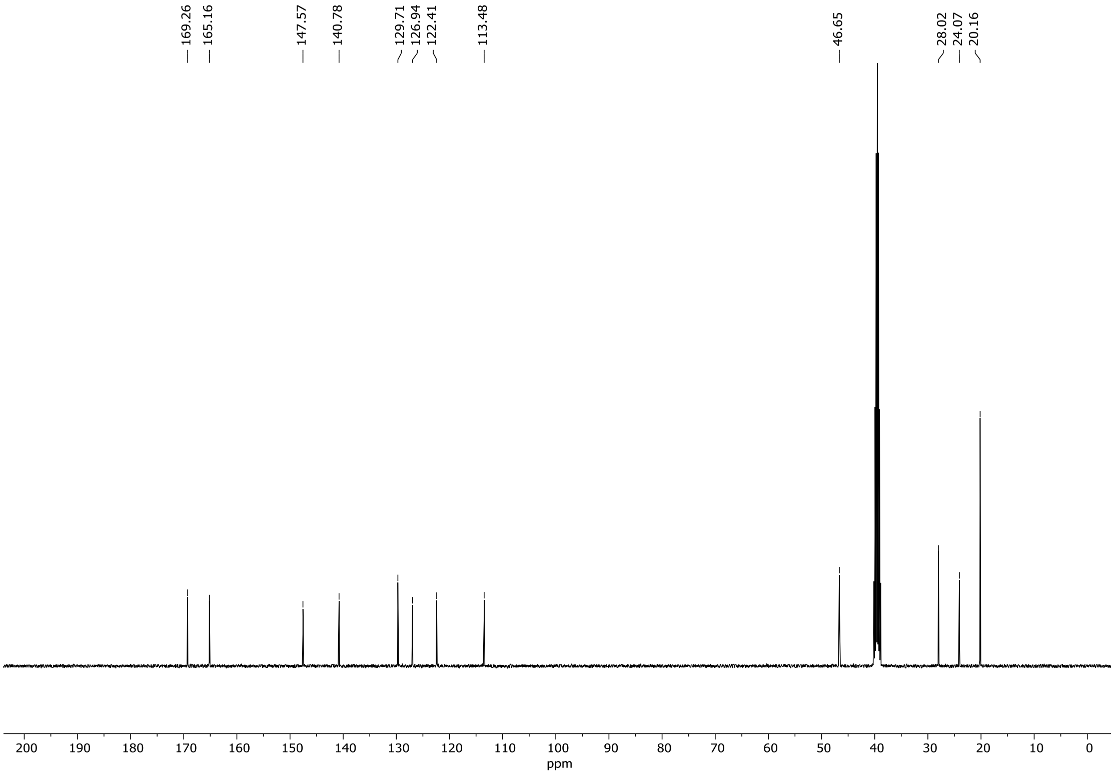

Figure S8: ^{13}C-NMR spectrum of compound **14**.

4-Amino-*N*-isobutyl-2-nitrobenzamide (**15**)

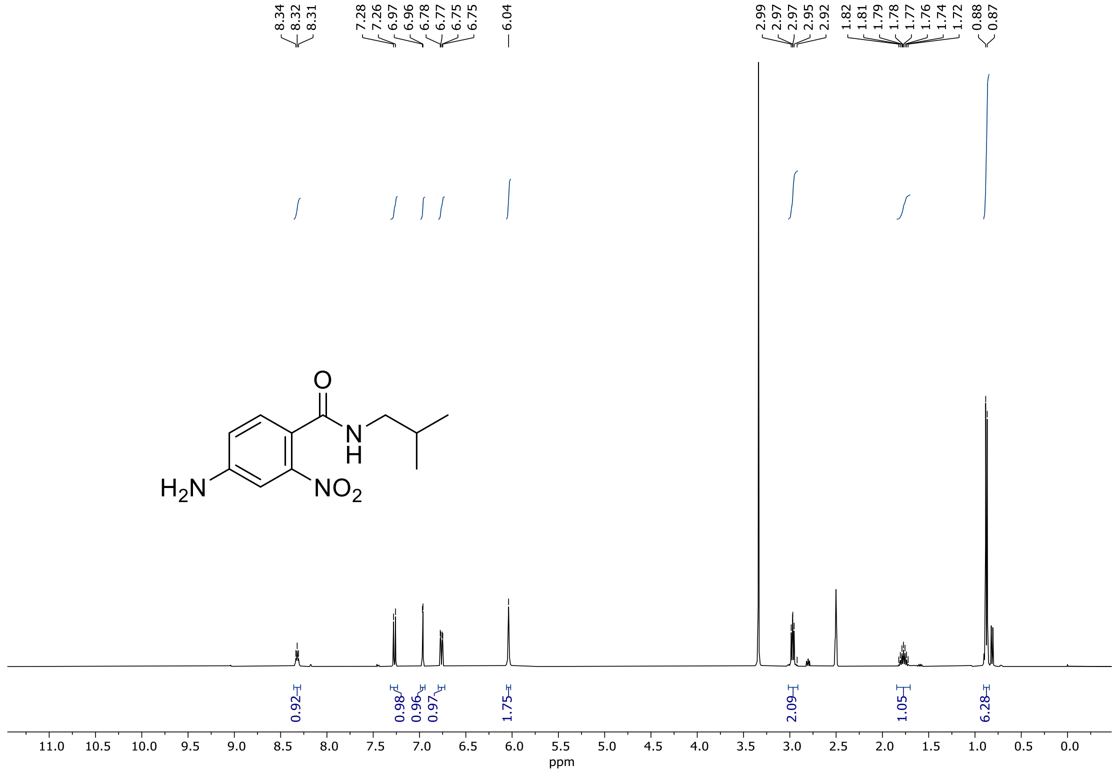

Figure S9: ^{1}H-NMR spectrum of compound **15**.

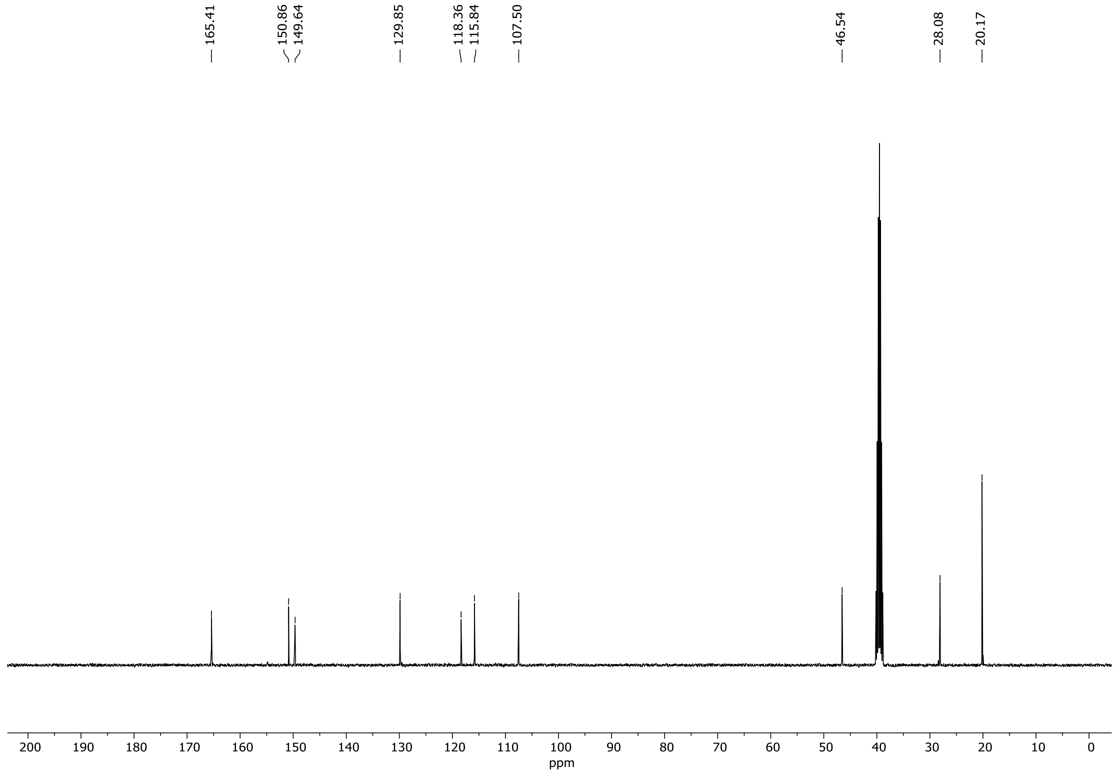

Figure S10: ^{13}C-NMR spectrum of compound **15**.

4-[(4-Fluorobenzyl)amino]-*N*-isobutyl-2-nitrobenzamide (**16**)

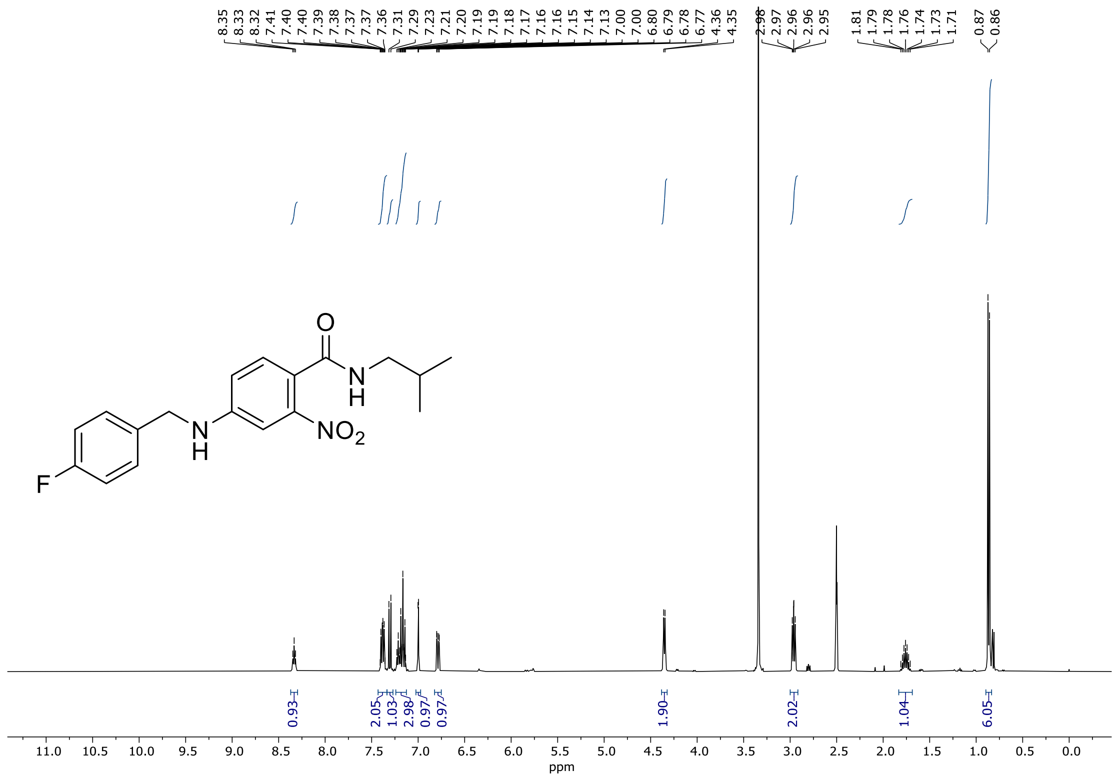

Figure S11: ^{1}H-NMR spectrum of compound **16**.

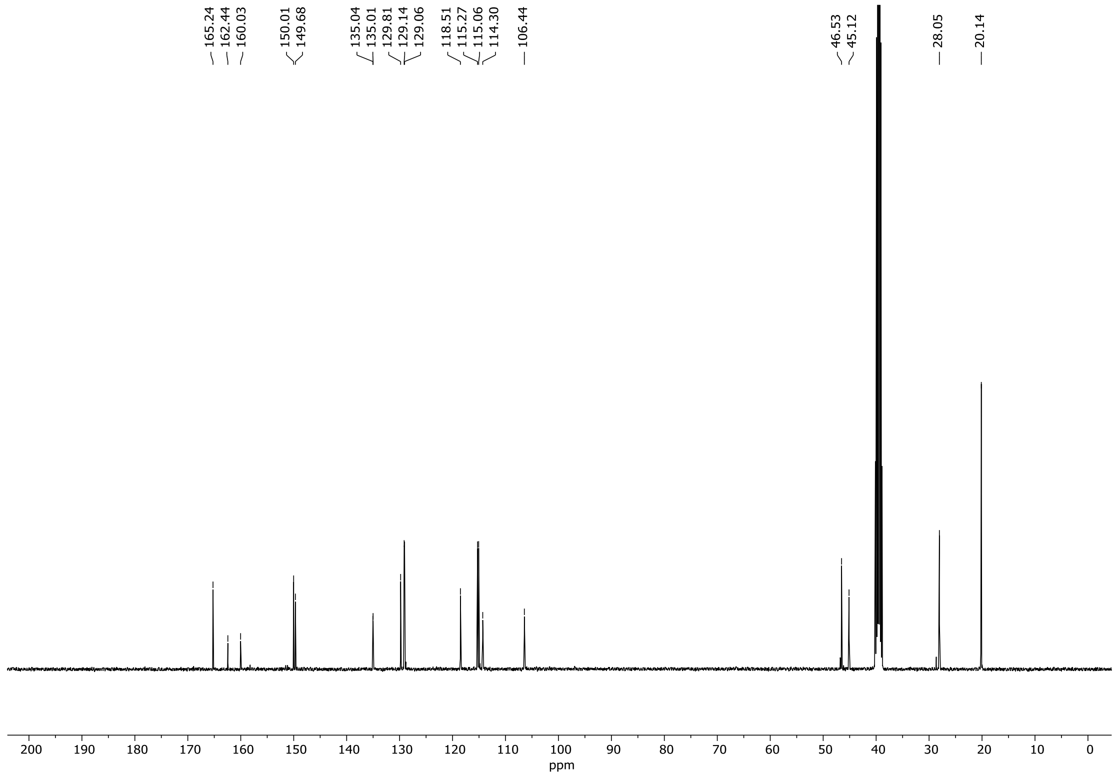

Figure S12: ^{13}C-NMR spectrum of compound **16**.

2-Amino-4-[(4-fluorobenzyl)amino]-*N*-isobutylbenzamide (**17**)

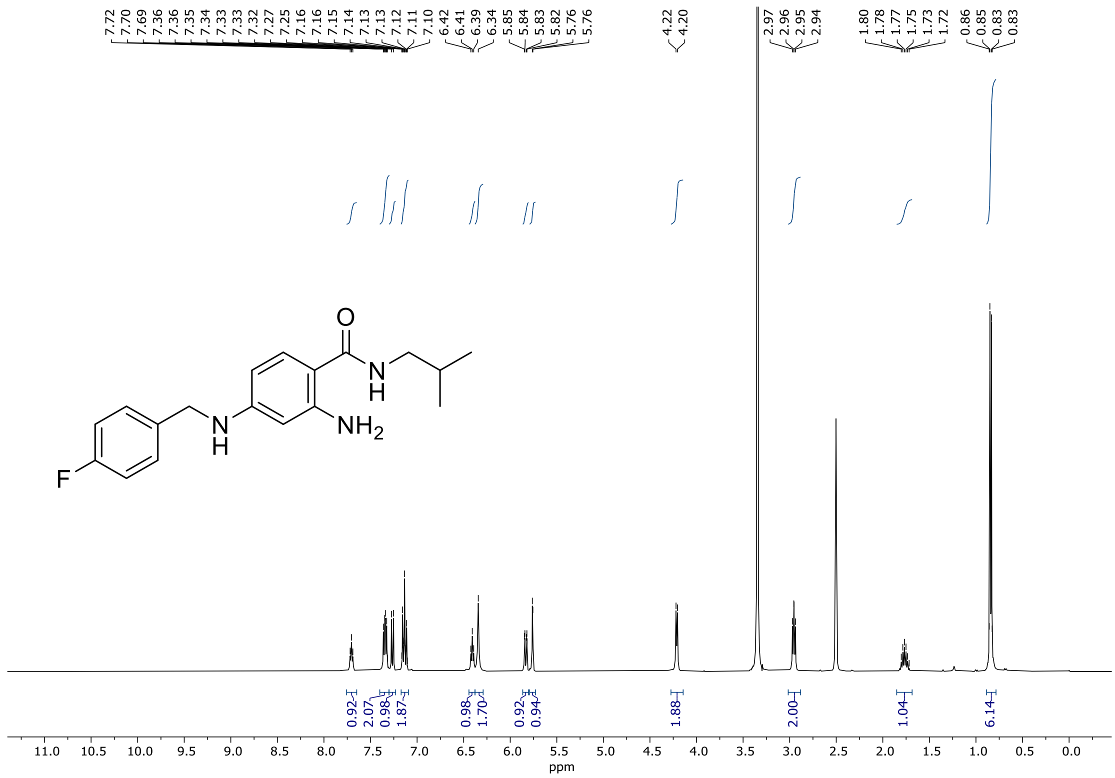

Figure S13: ^{1}H-NMR spectrum of compound **17**.

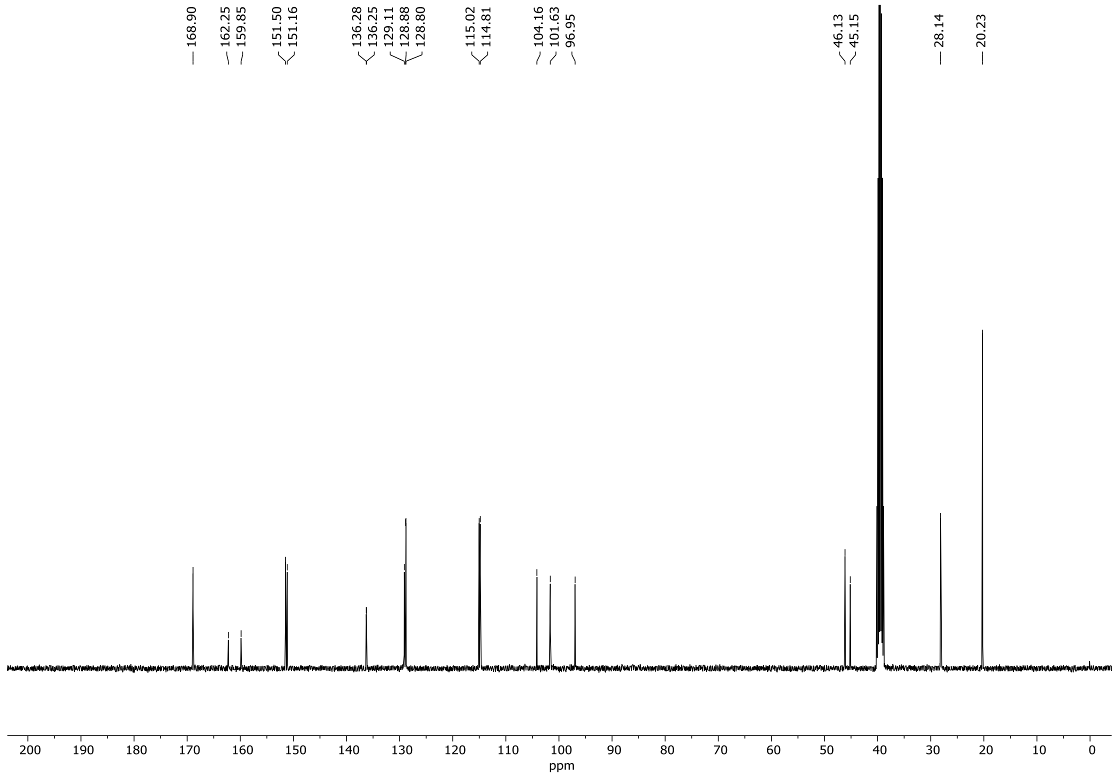

Figure S14: ^{13}C-NMR spectrum of compound **17**.

2-Amino-6-chloronicotinic acid (**19**)

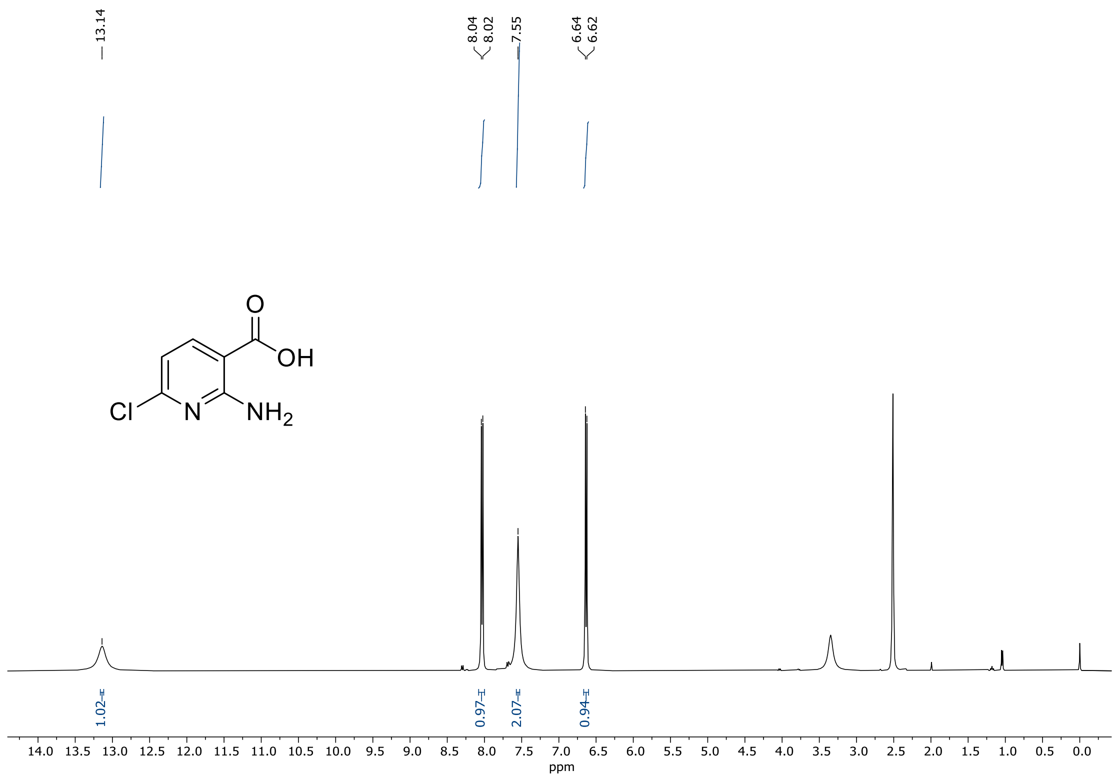

Figure S15: ^{1}H-NMR spectrum of compound **19**.

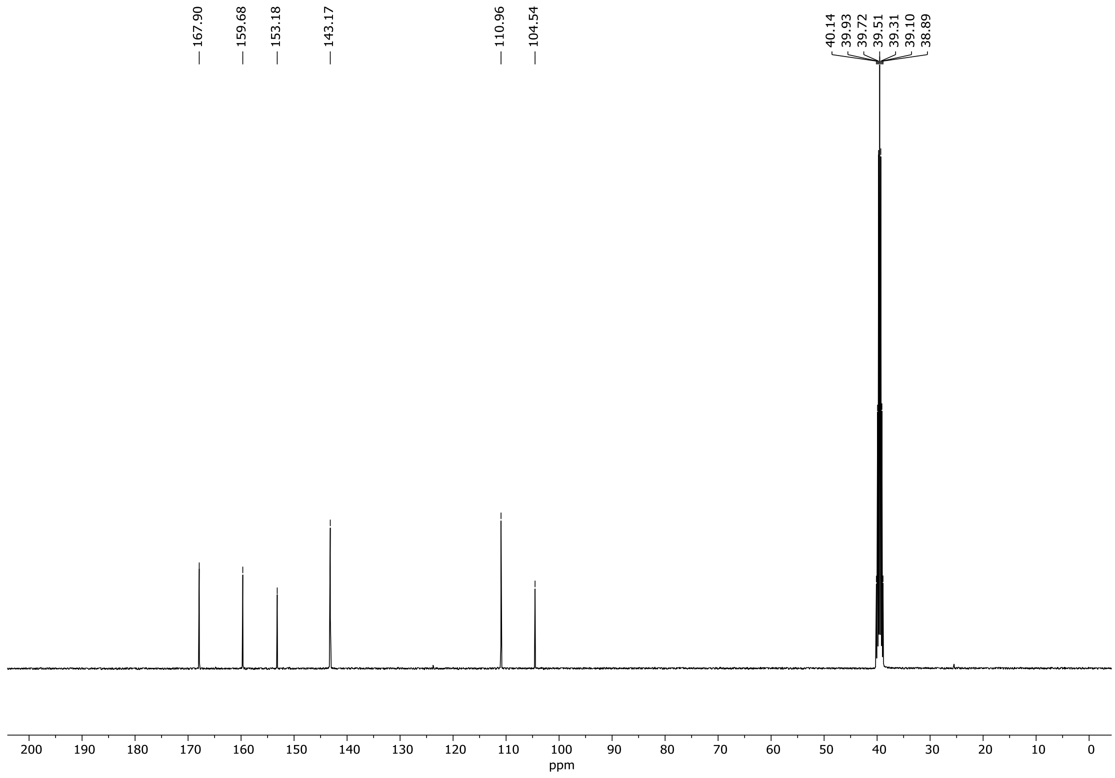

Figure S16: ^{13}C-NMR spectrum of compound **19**.

2-Amino-*N*-butyl-6-chloronicotinamide (**20**)

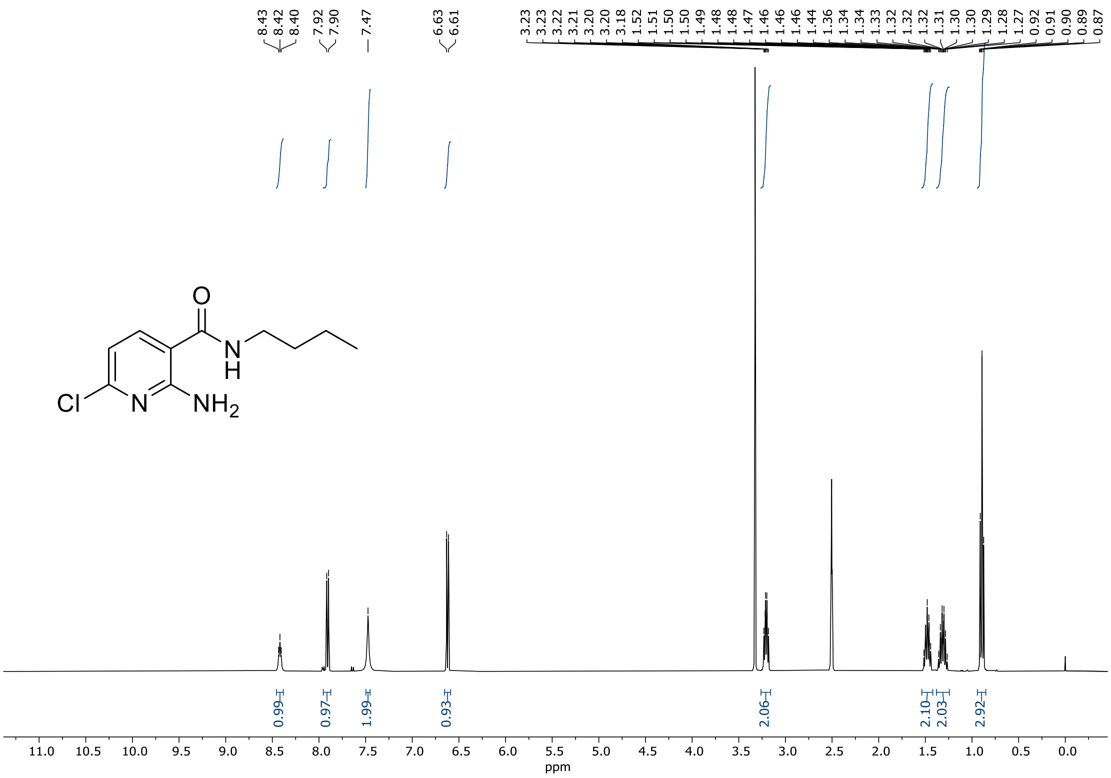

Figure S17: ^{1}H-NMR spectrum of compound **20**.

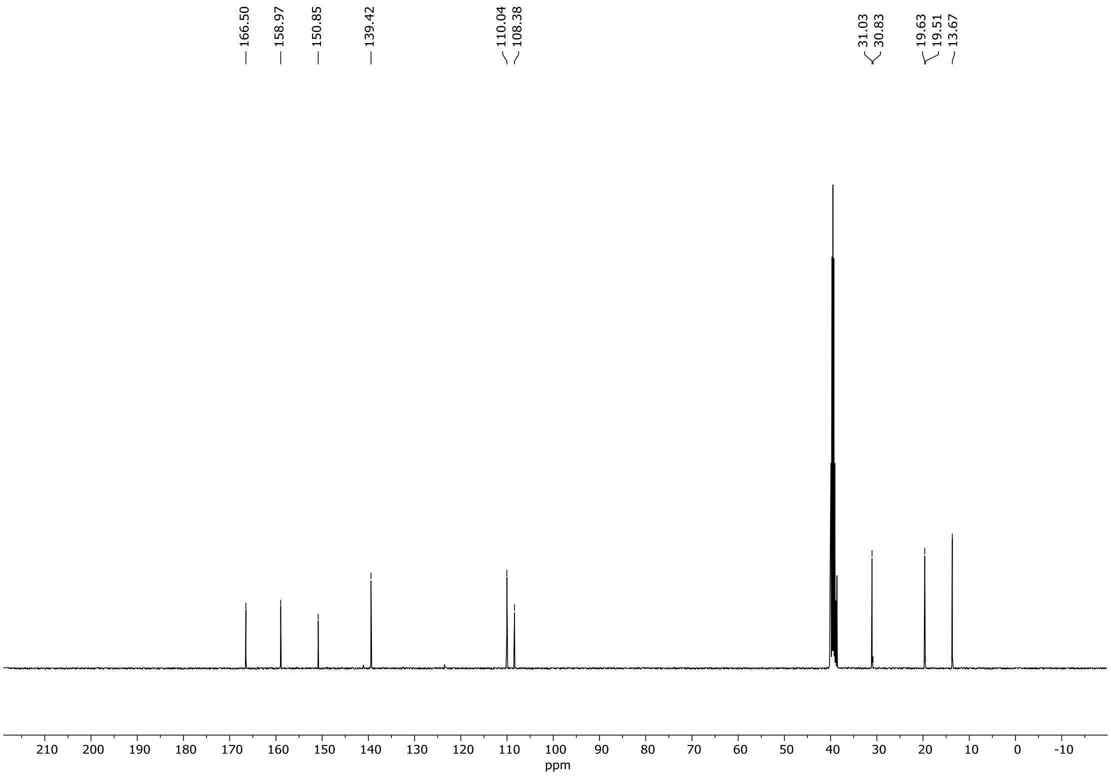

Figure S18: ^{13}C-NMR spectrum of compound **20**.

2-Amino-*N*-butyl-6-[(4-fluorobenzyl)amino]nicotinamide (**21**)

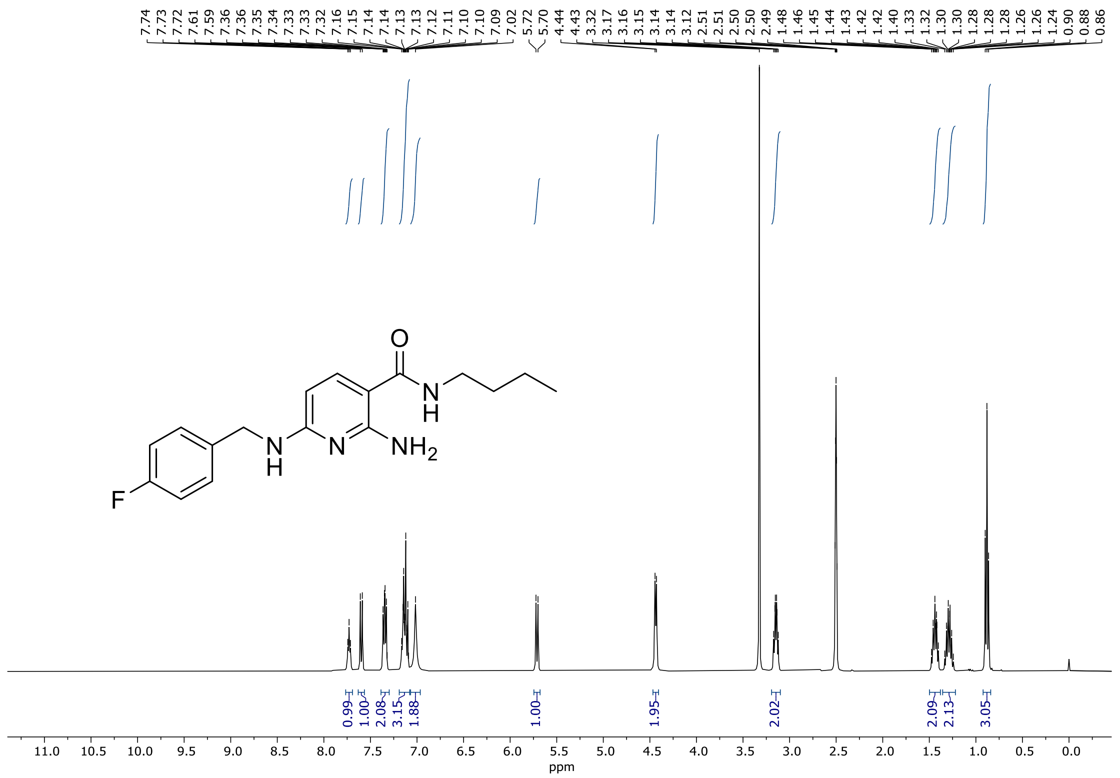

Figure S19: ^{1}H-NMR spectrum of compound **21**.

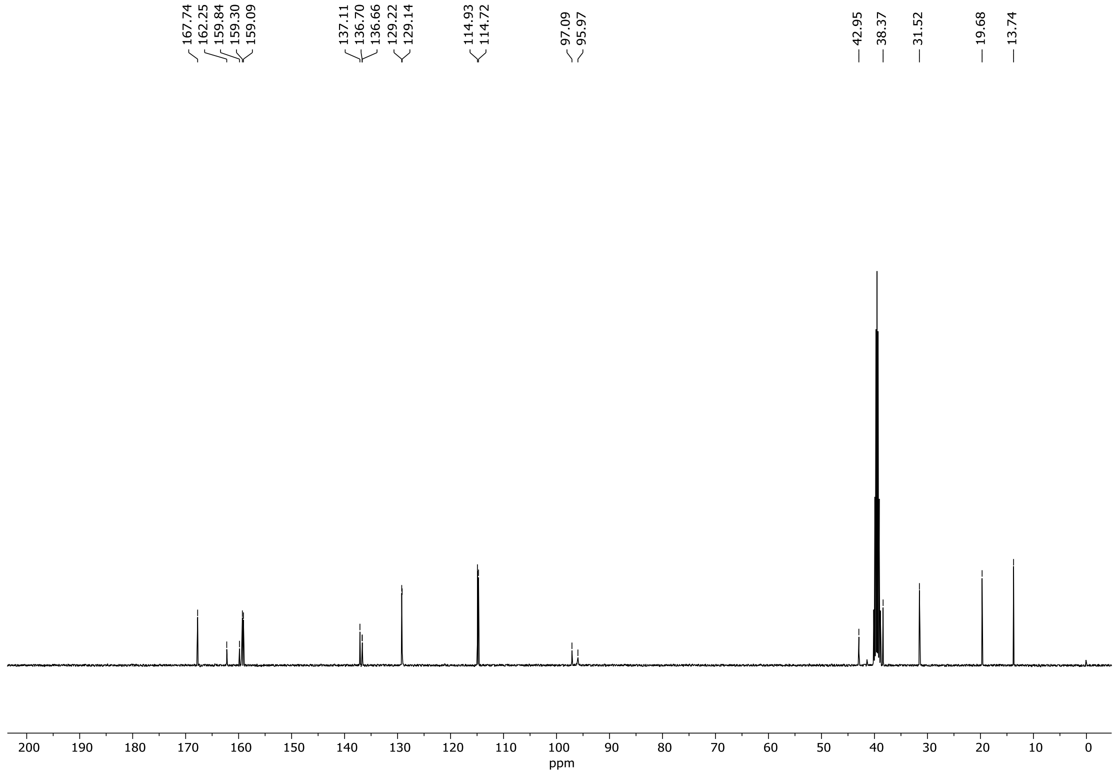

Figure S20: ^{13}C-NMR spectrum of compound **21**.

2-Bromo-1-methyl-4-nitrobenzene (**23**)

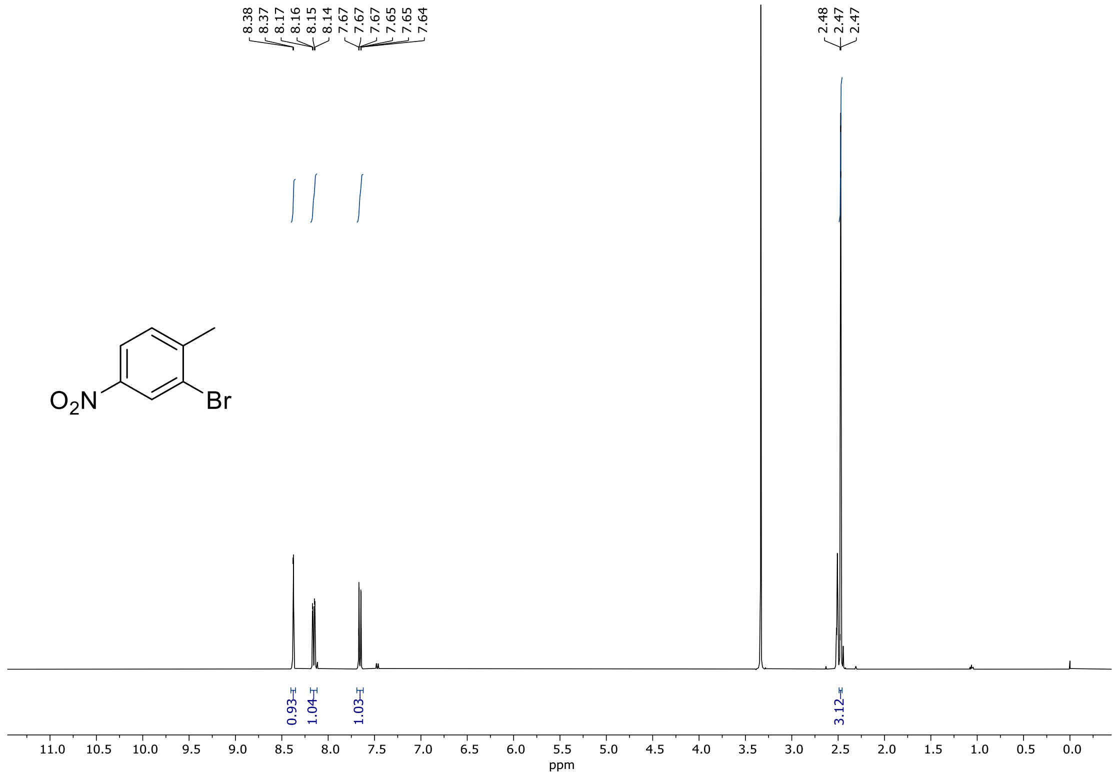

Figure S21: ^{1}H-NMR spectrum of compound **23**.

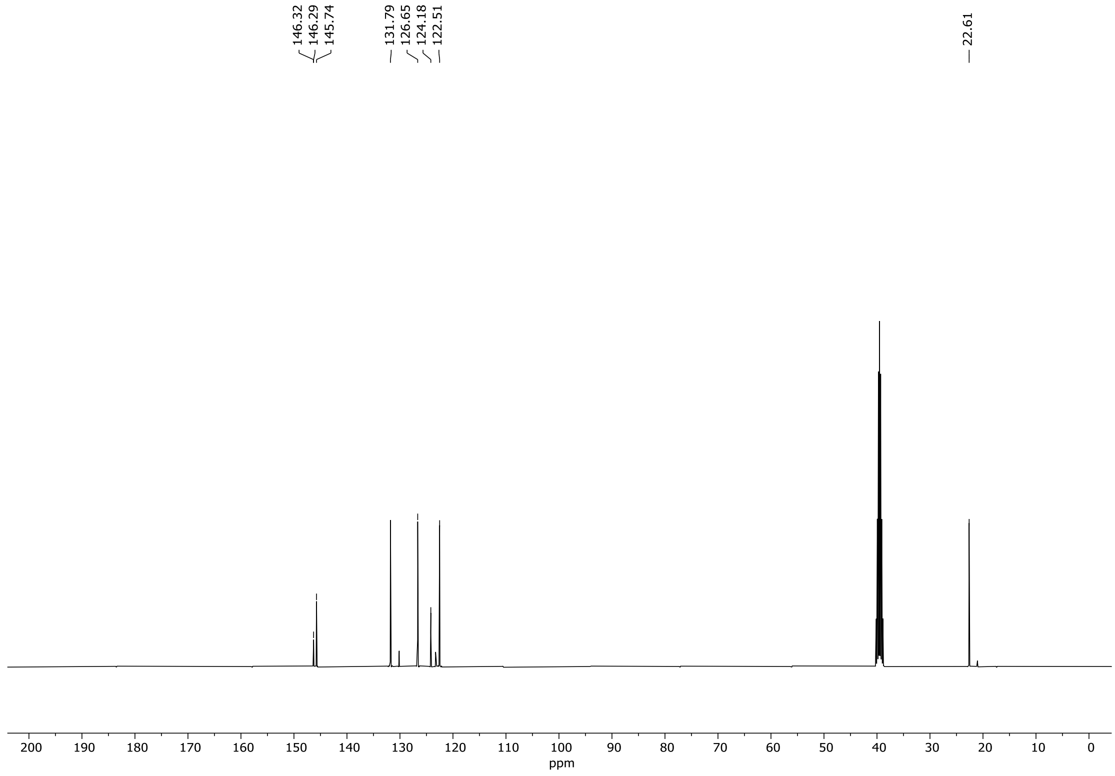

Figure S22: ^{13}C-NMR spectrum of compound **23**.

2-Bromo-4-nitrobenzoic acid (**24**)

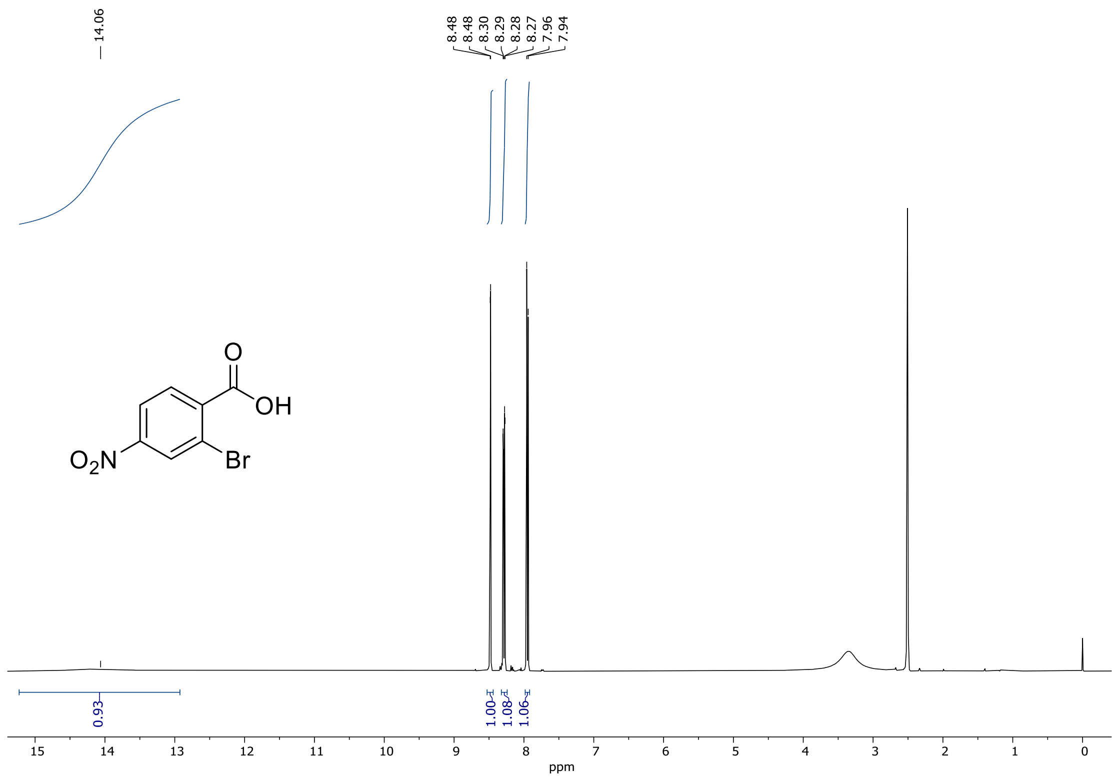

Figure S23: ^{1}H-NMR spectrum of compound **24**.

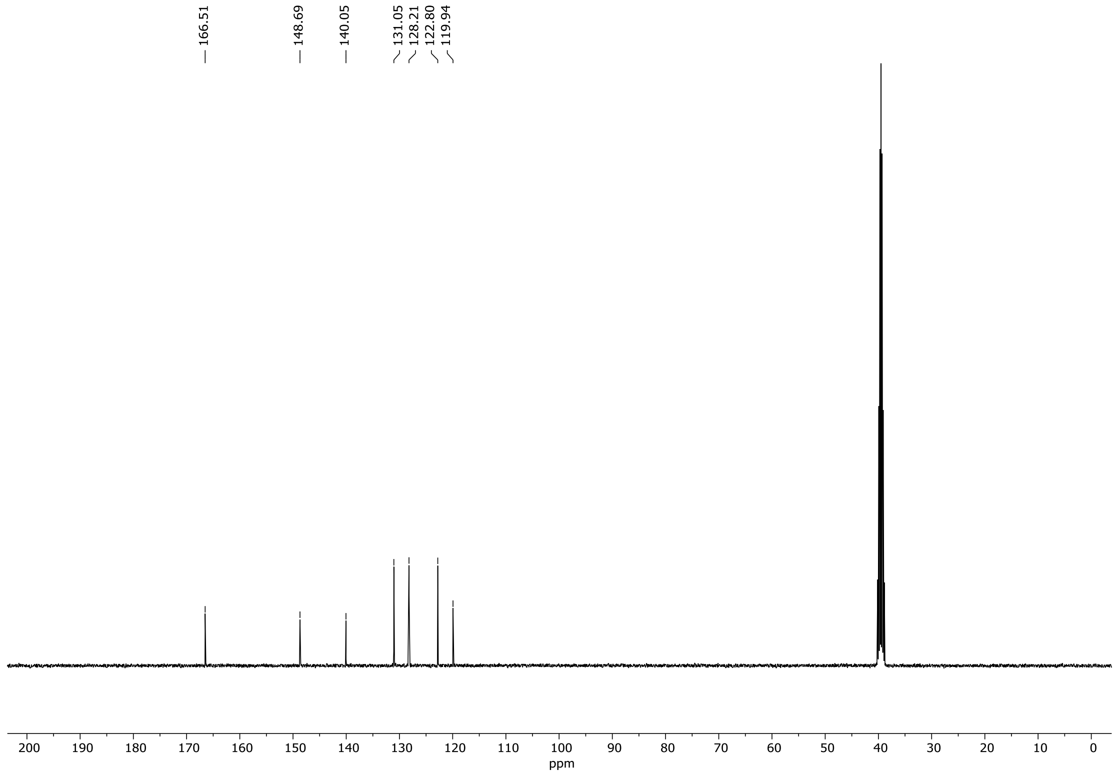

Figure S24: ^{13}C-NMR spectrum of compound **24**.

2-Bromo-*N*-isobutyl-4-nitrobenzamide (**25a**)

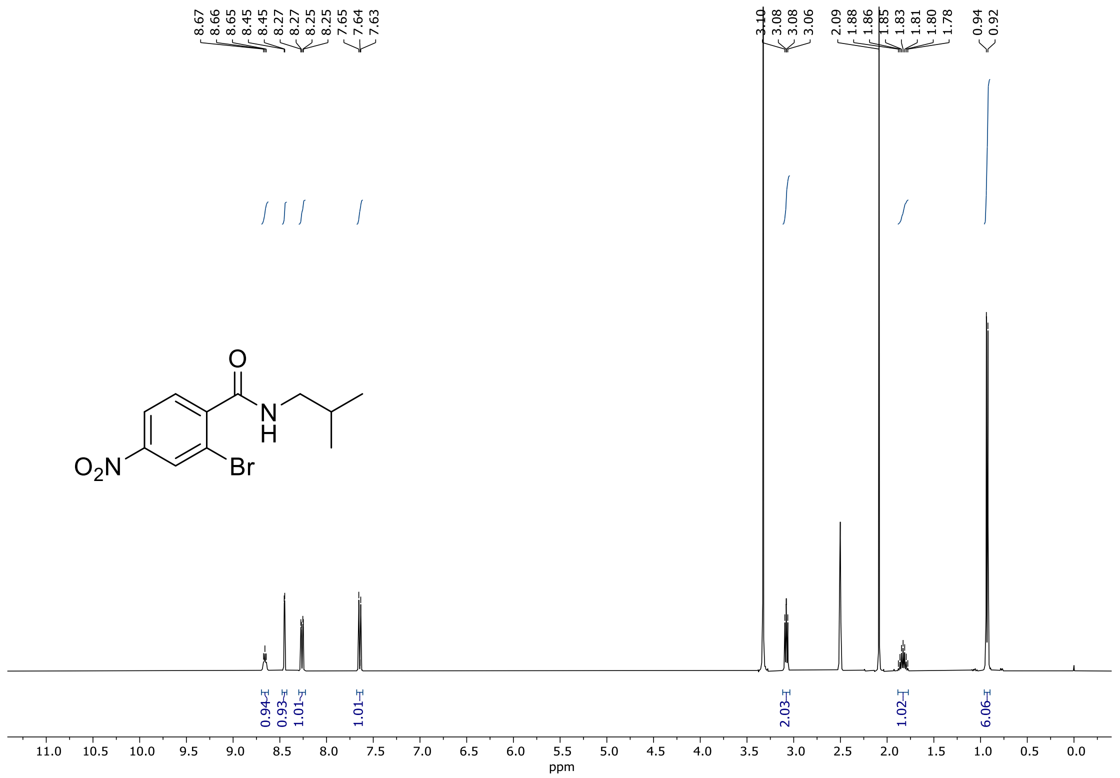

Figure S25: ^{1}H-NMR spectrum of compound **25a**.

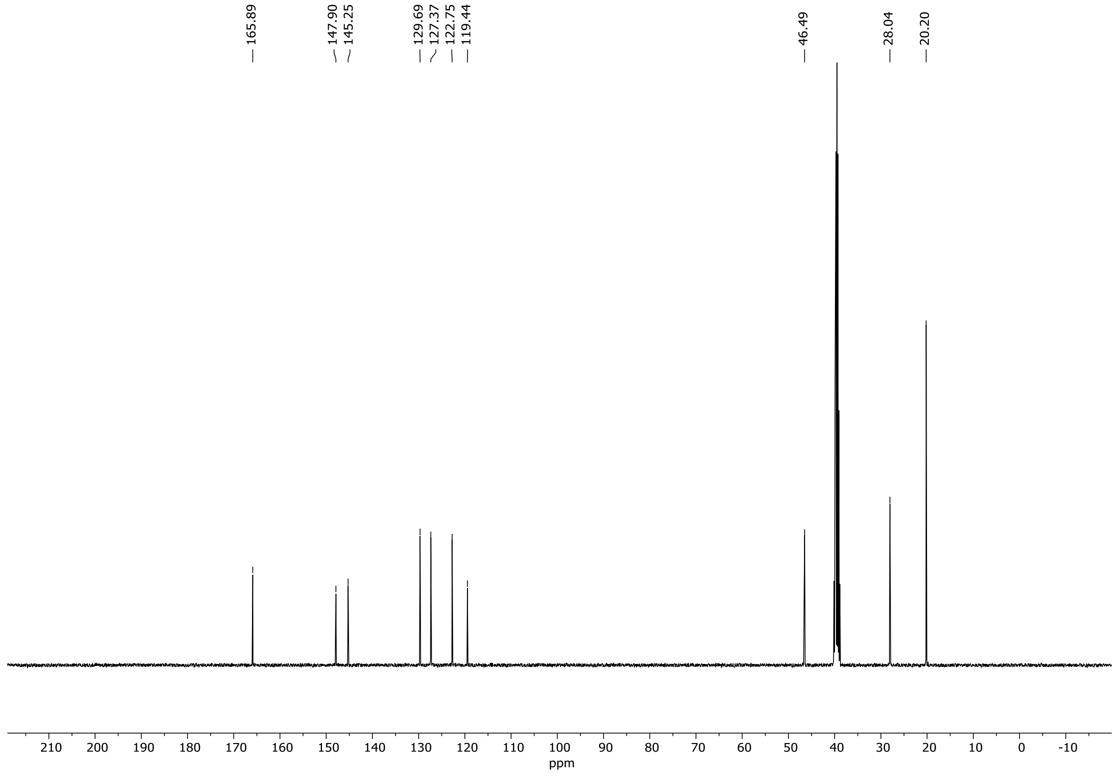

Figure S26: ^{13}C-NMR spectrum of compound **25a**.

2-Bromo-*N*-(4-fluorobenzyl)-4-nitrobenzamide (**25b**)

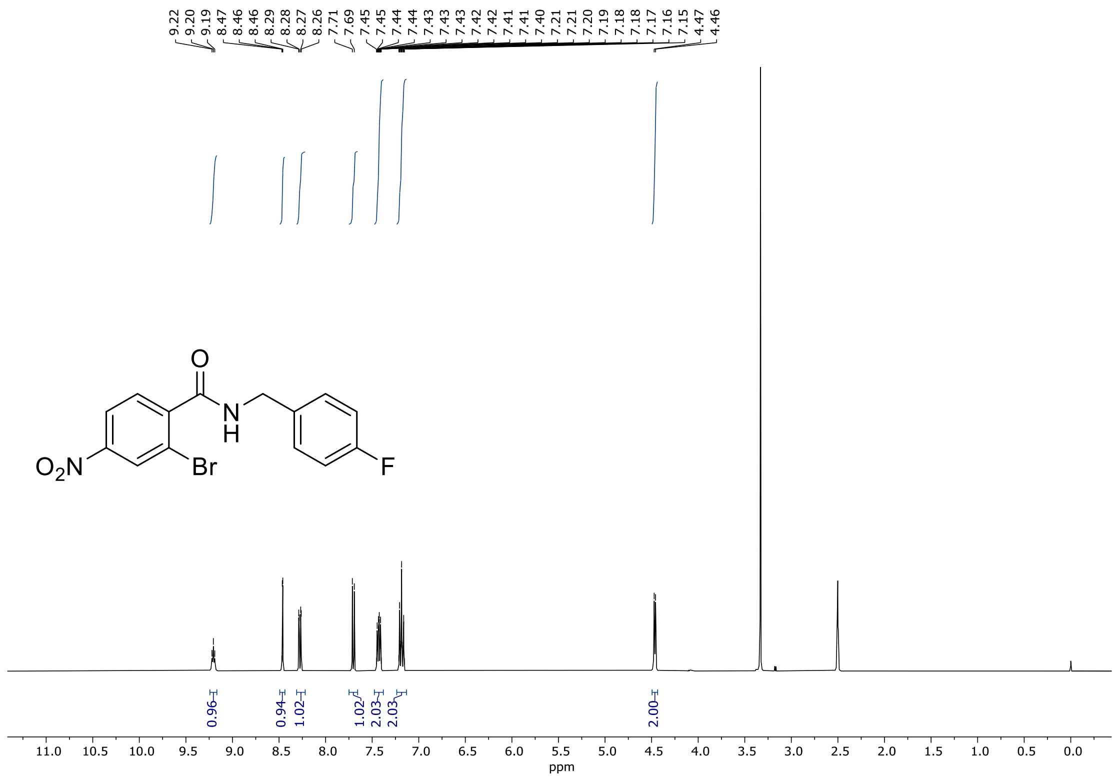

Figure S27: ^{1}H-NMR spectrum of compound **25b**.

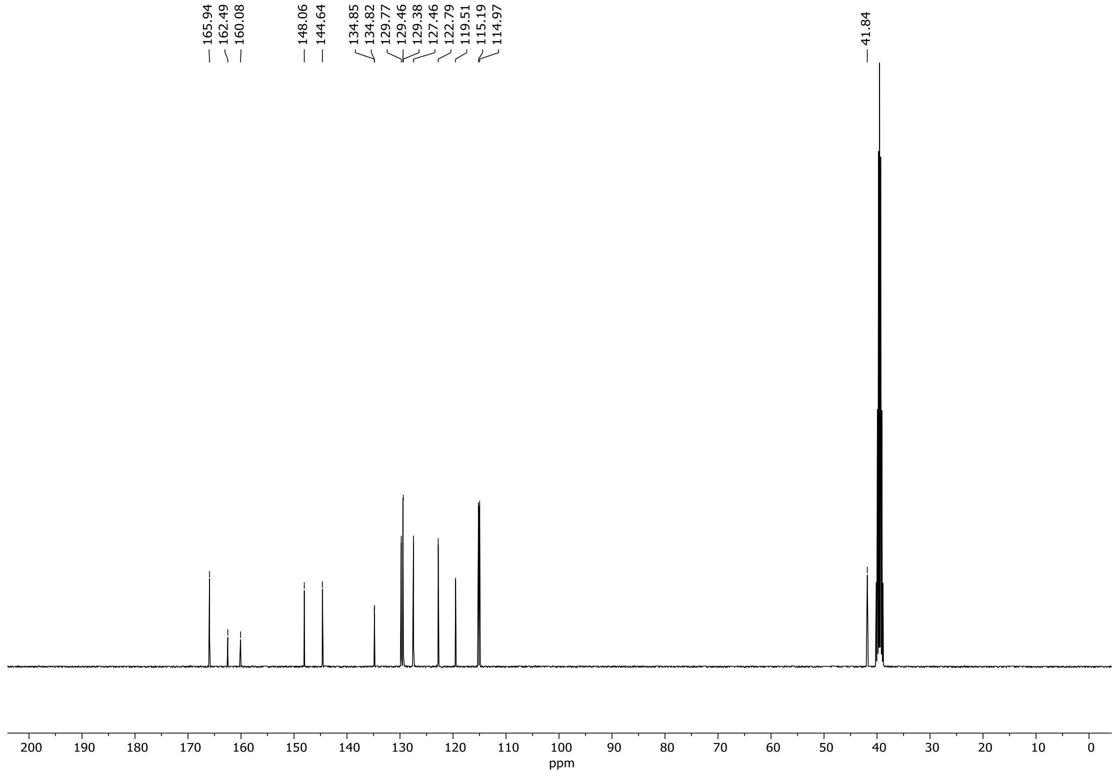

Figure S28: ^{13}C-NMR spectrum of compound **25b**.

N-Isobutyl-2-methoxy-4-nitrobenzamide (**26a**)

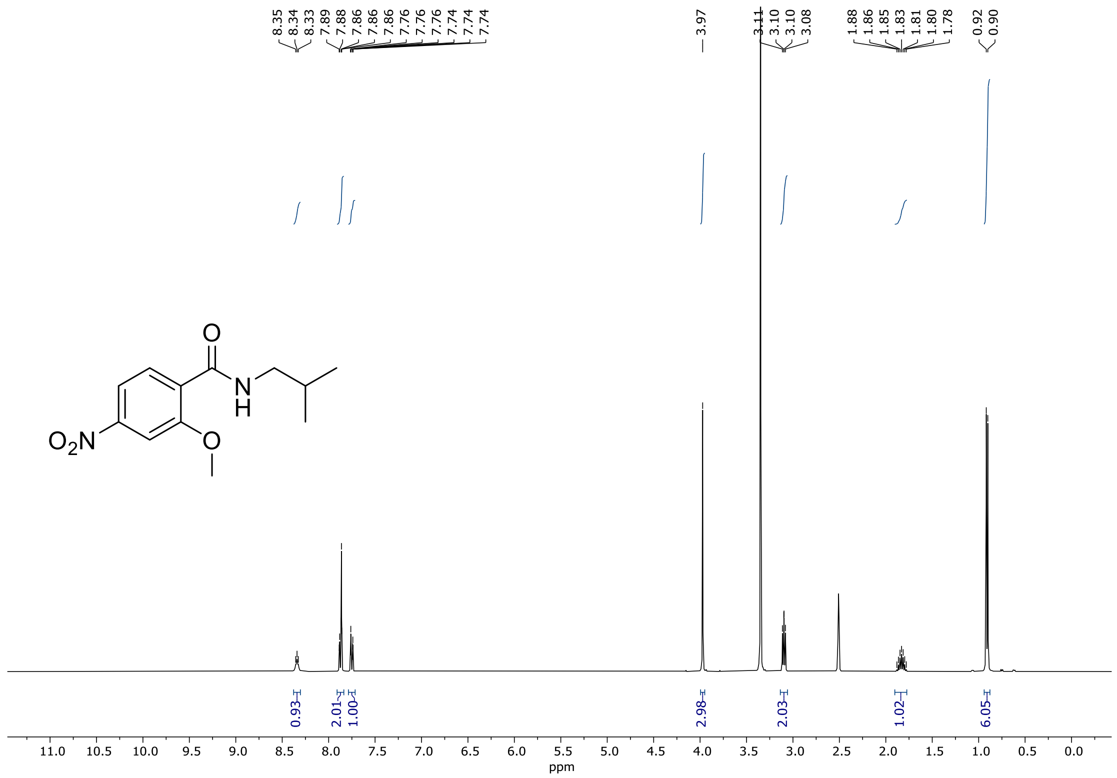

Figure S29: ^{1}H-NMR spectrum of compound **26a**.

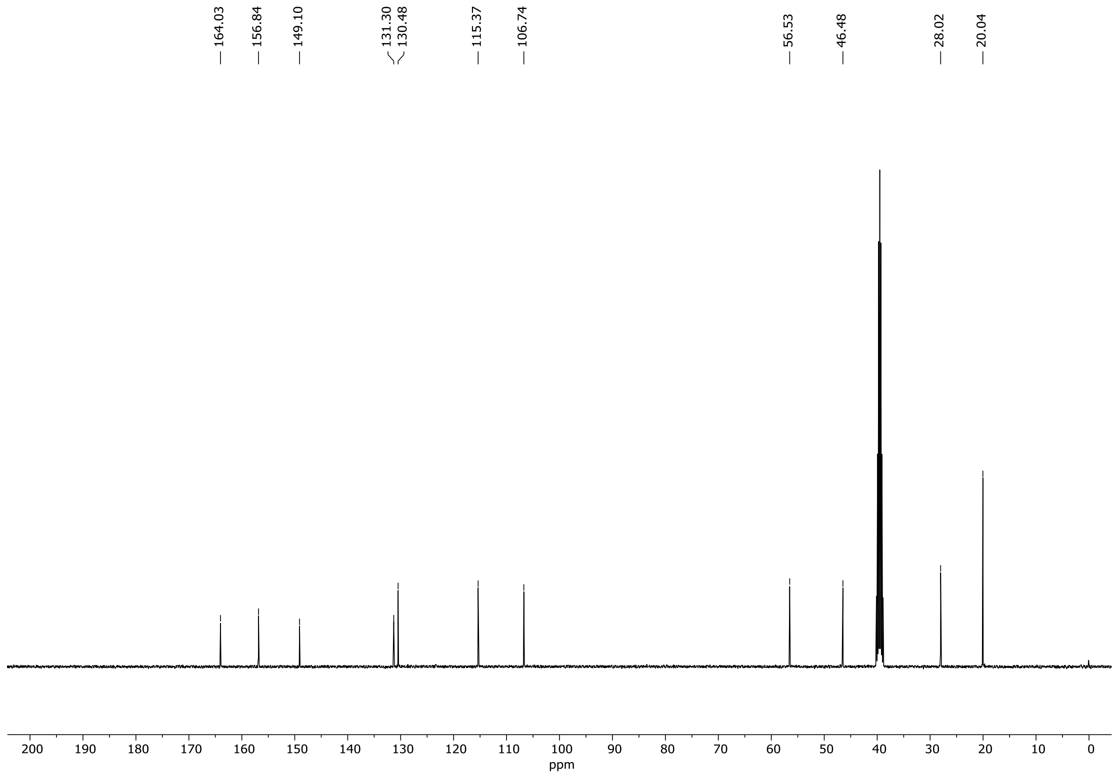

Figure S30: ^{13}C-NMR spectrum of compound **26a**.

N-(4-Fluorobenzyl)-2-methoxy-4-nitrobenzamide (**26b**)

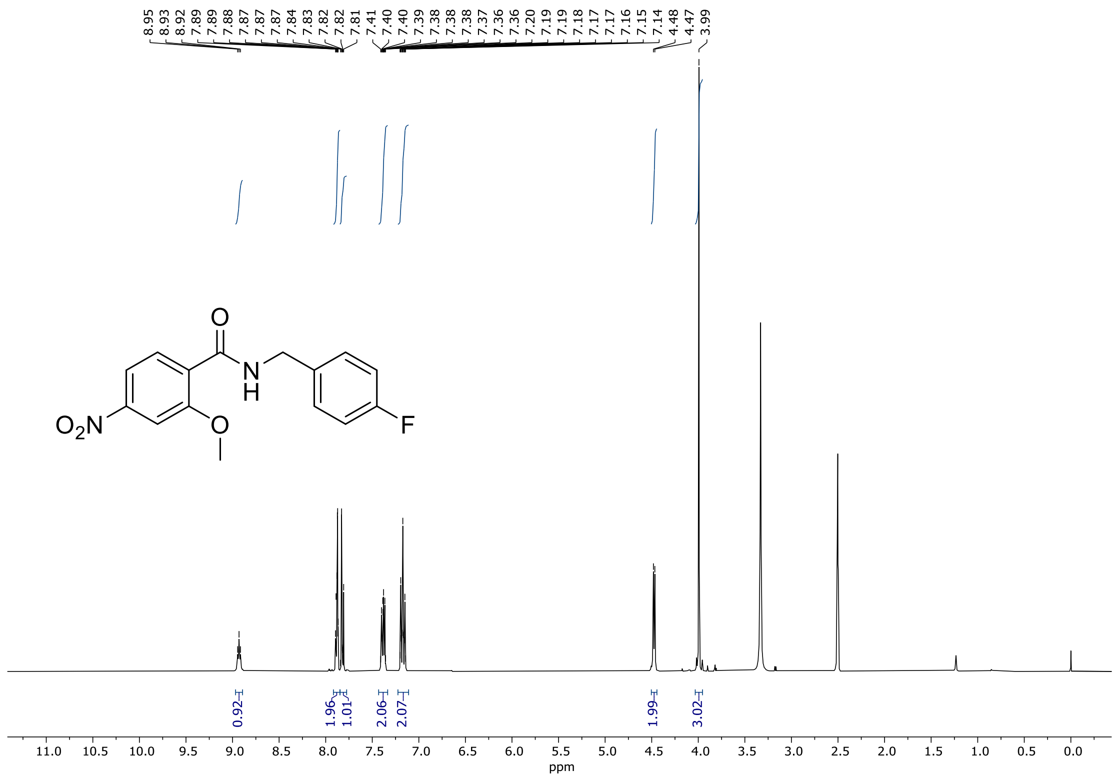

Figure S31: ^{1}H-NMR spectrum of compound **26b**.

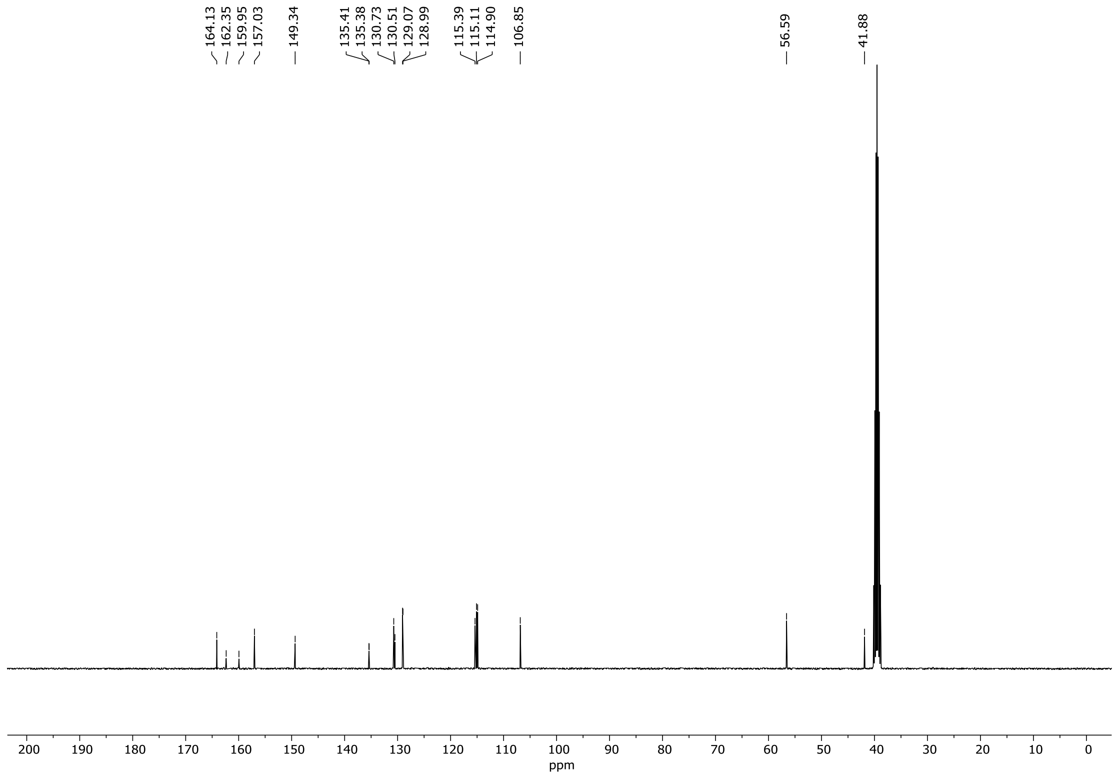

Figure S32: ^{13}C-NMR spectrum of compound **26b**.

4-Amino-*N*-isobutyl-2-methoxybenzamide (**27a**)

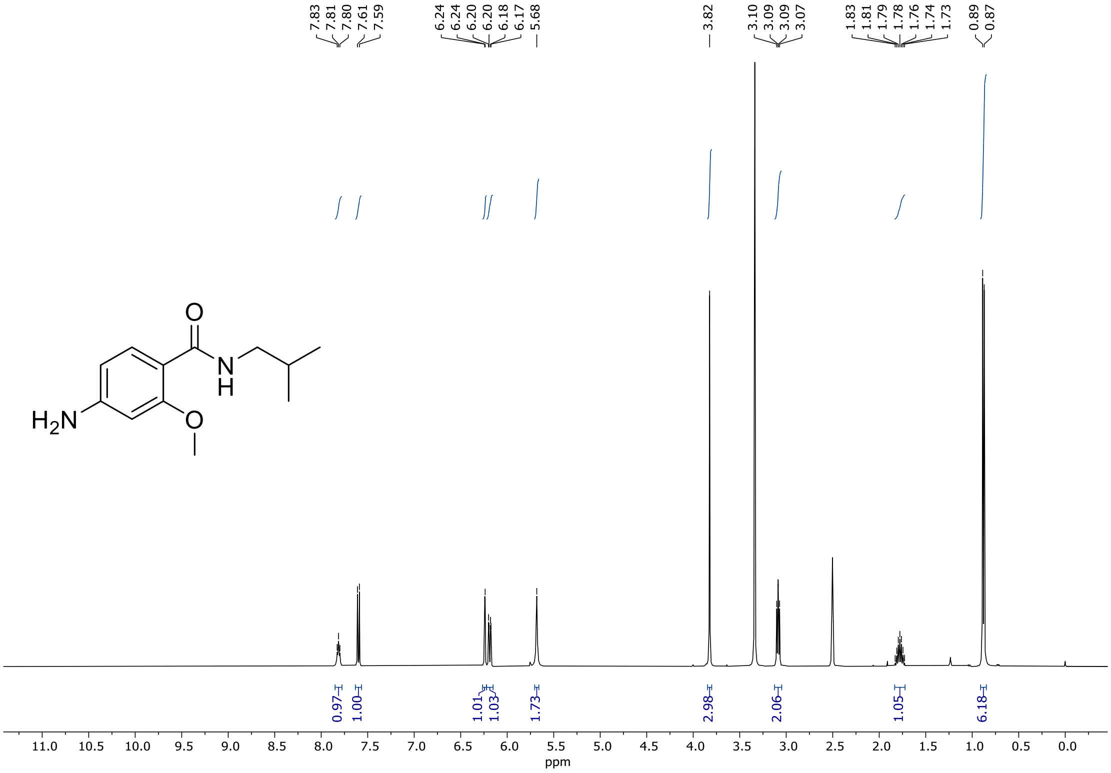

Figure S33: ^{1}H-NMR spectrum of compound **27a**.

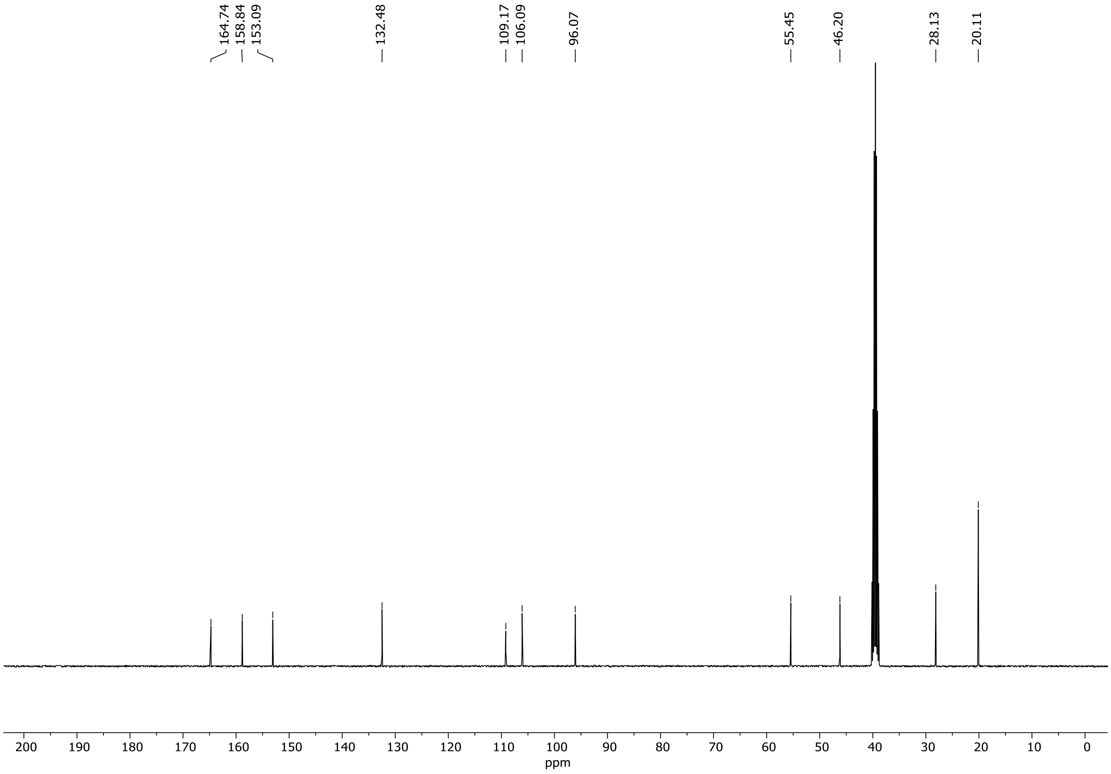

Figure S34: ^{13}C-NMR spectrum of compound **27a**.

4-Amino-*N*-(4-fluorobenzyl)-2-methoxybenzamide (**27b**)

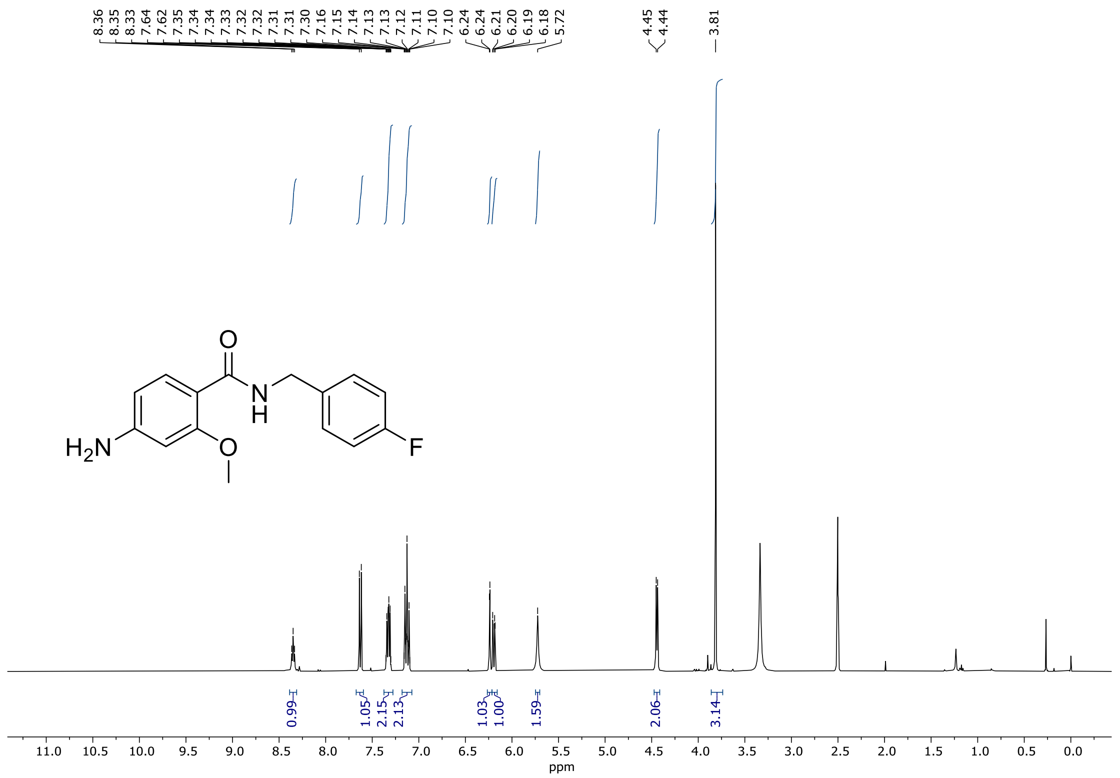

Figure S35: ^{1}H-NMR spectrum of compound **27b**.

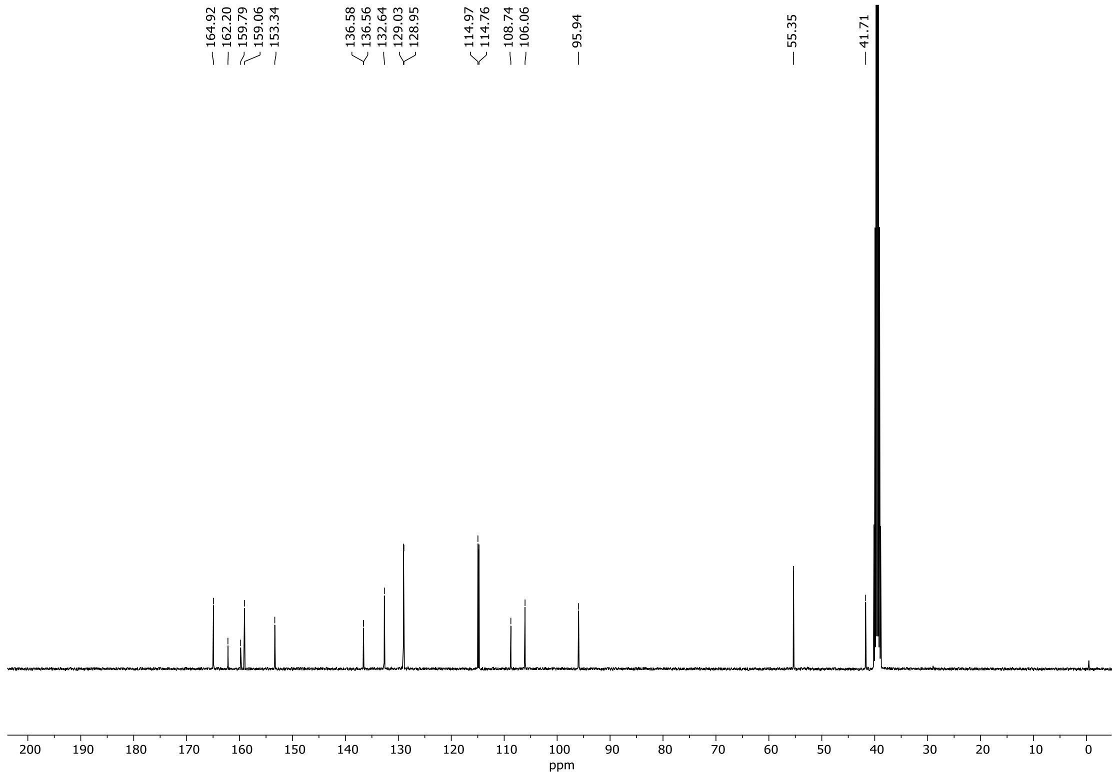

Figure S36: ^{13}C-NMR spectrum of compound **27b**.

4-[(4-Fluorobenzyl)amino]-*N*-isobutyl-2-methoxybenzamide (**28a**)

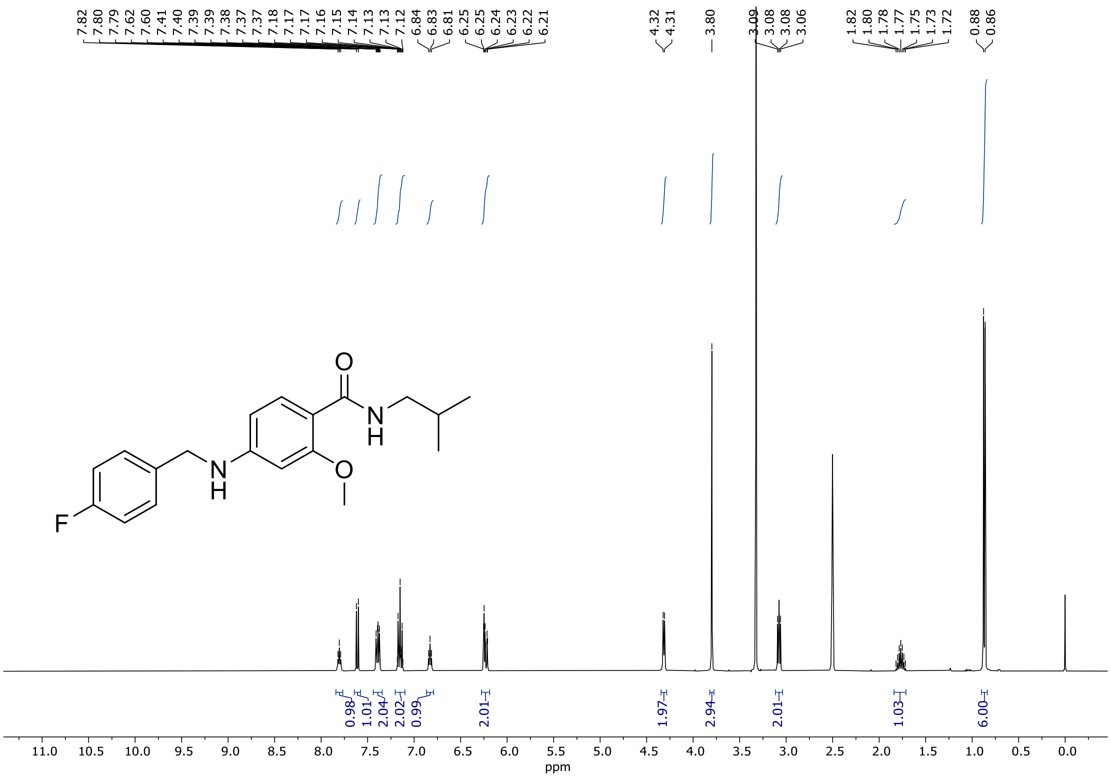

Figure S37: ^{1}H-NMR spectrum of compound **28a**.

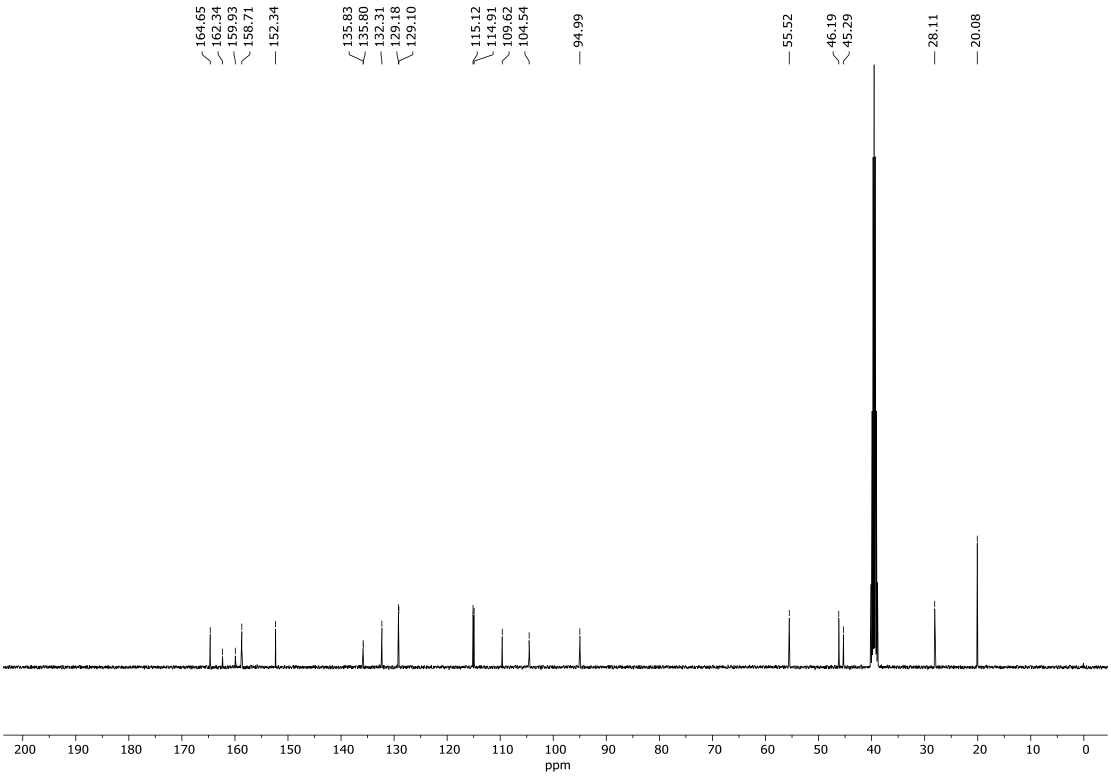

Figure S38: ^{13}C-NMR spectrum of compound **28a**.

N-(4-Fluorobenzyl)-4-[(4-fluorobenzyl)amino]-2-methoxybenzamide (**28b**)

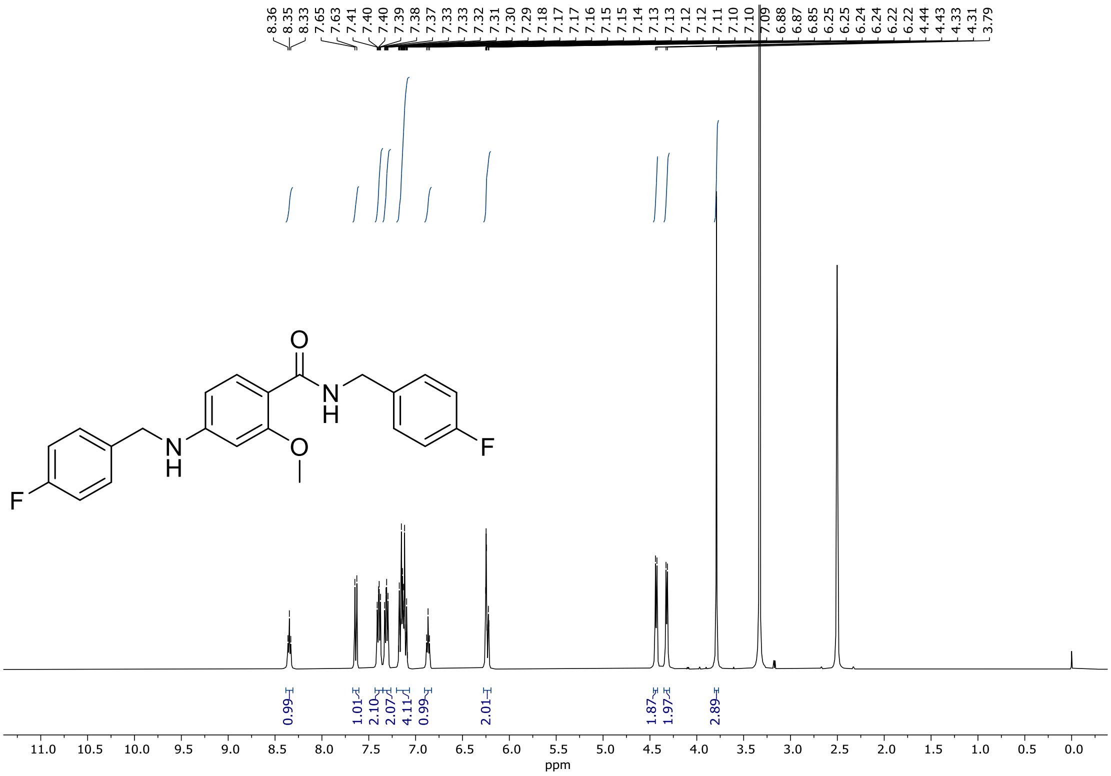

Figure S39: ^{1}H-NMR spectrum of compound **28b**.

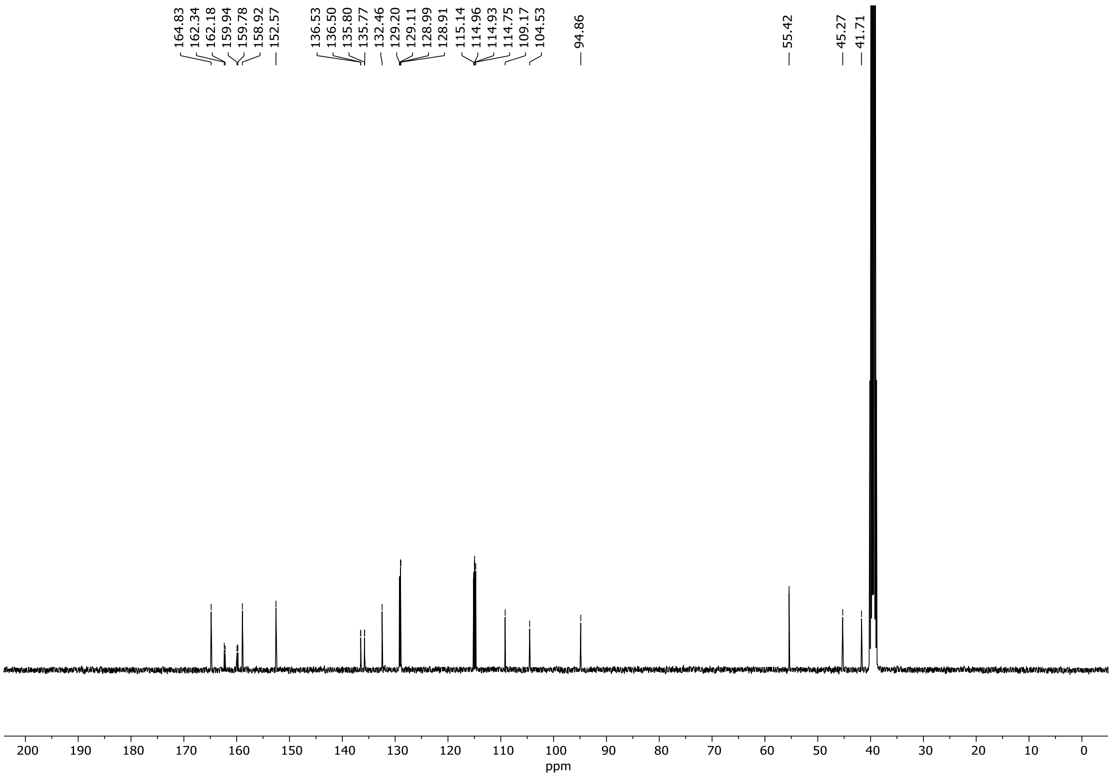

Figure S40: ^{13}C-NMR spectrum of compound **28b**.

2,6-Dihydroxy-4-methylnicotinonitrile (**31**)

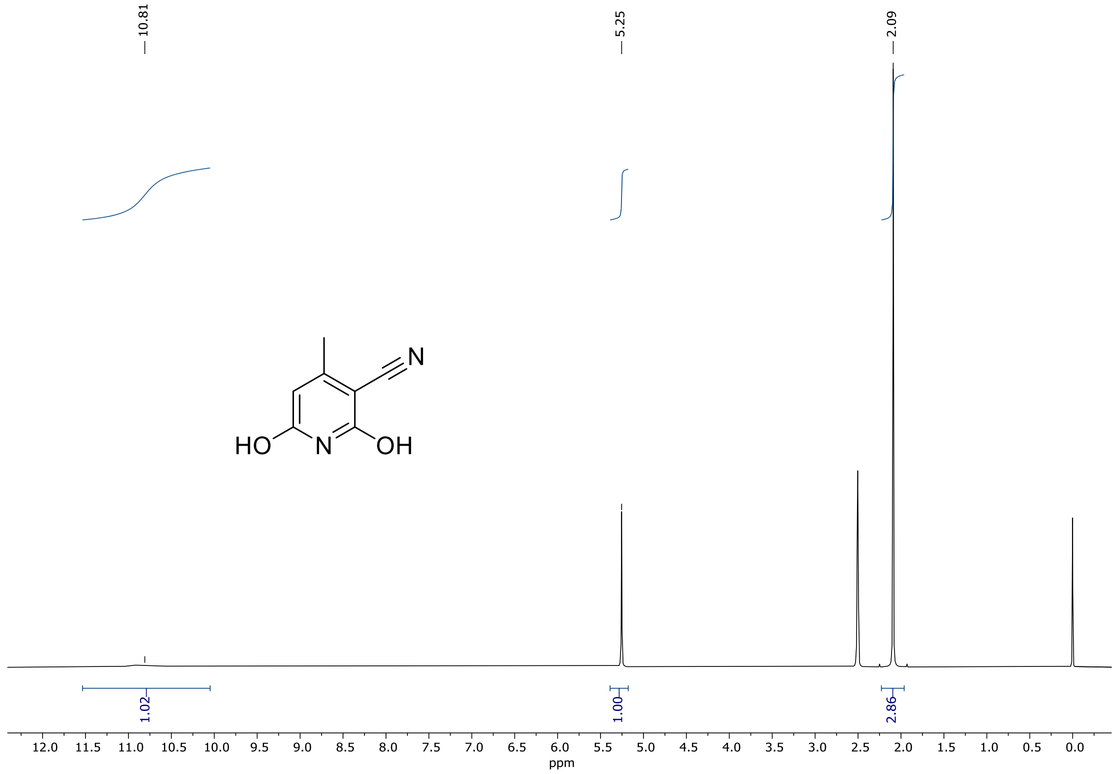

Figure S41: ^{1}H-NMR spectrum of compound **31**.

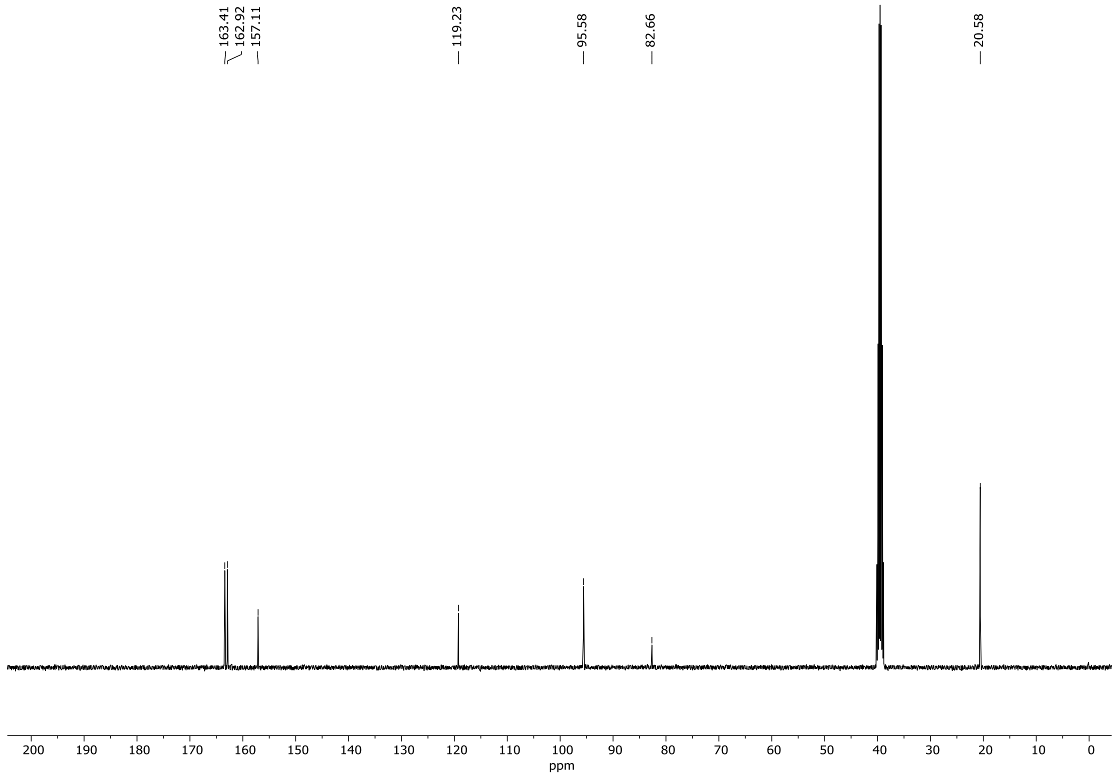

Figure S42: ^{13}C-NMR spectrum of compound **31**.

2,6-Dichloro-4-methylnicotinonitrile (**32**)

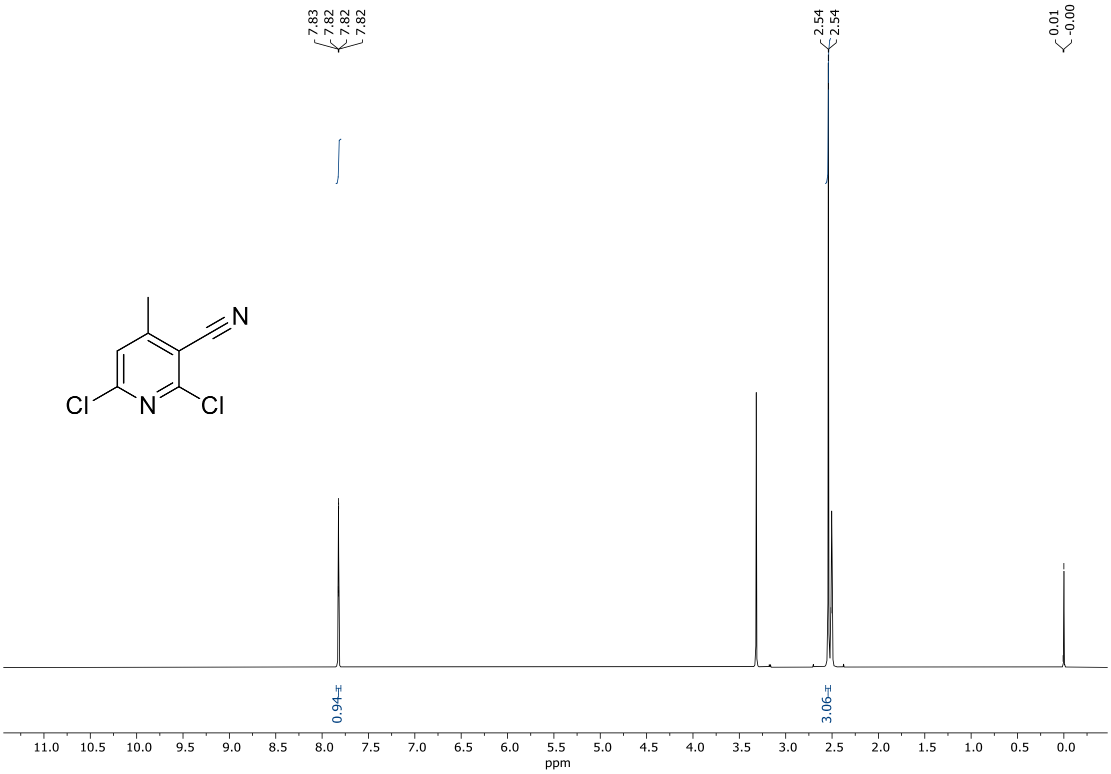

Figure S43: ^{1}H-NMR spectrum of compound **32**.

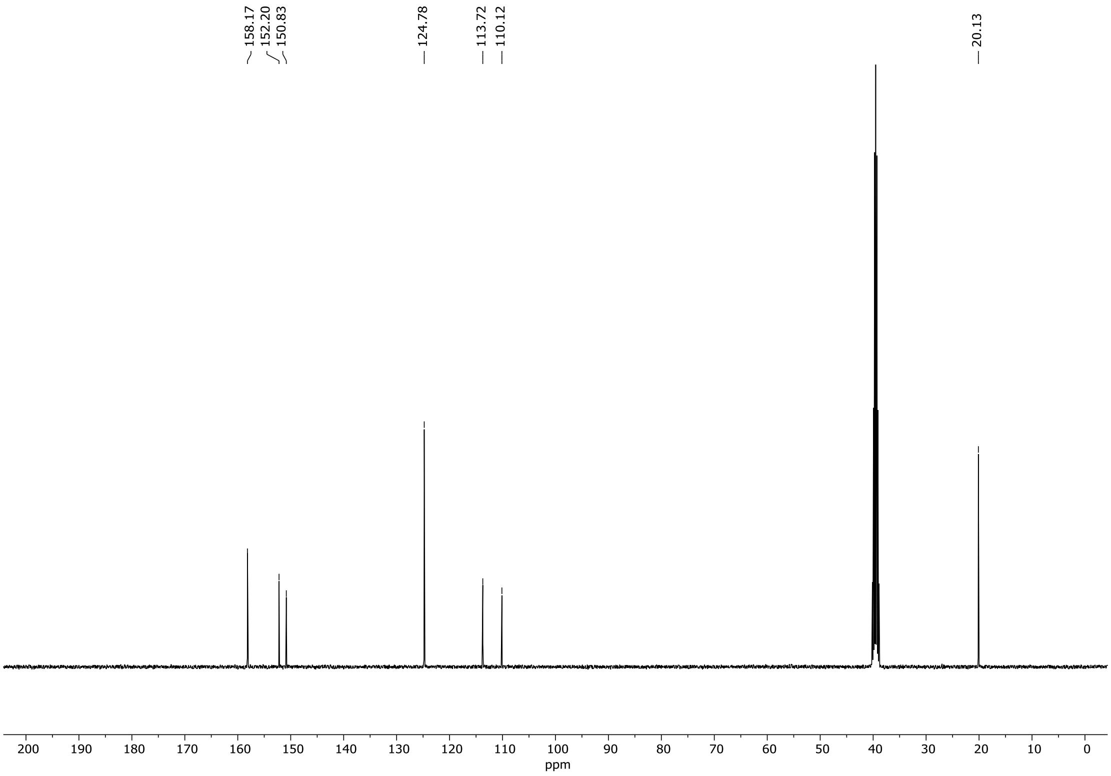

Figure S44: ^{13}C-NMR spectrum of compound **32**.

2,6-Dichloro-4-methylnicotinic acid (**33**)

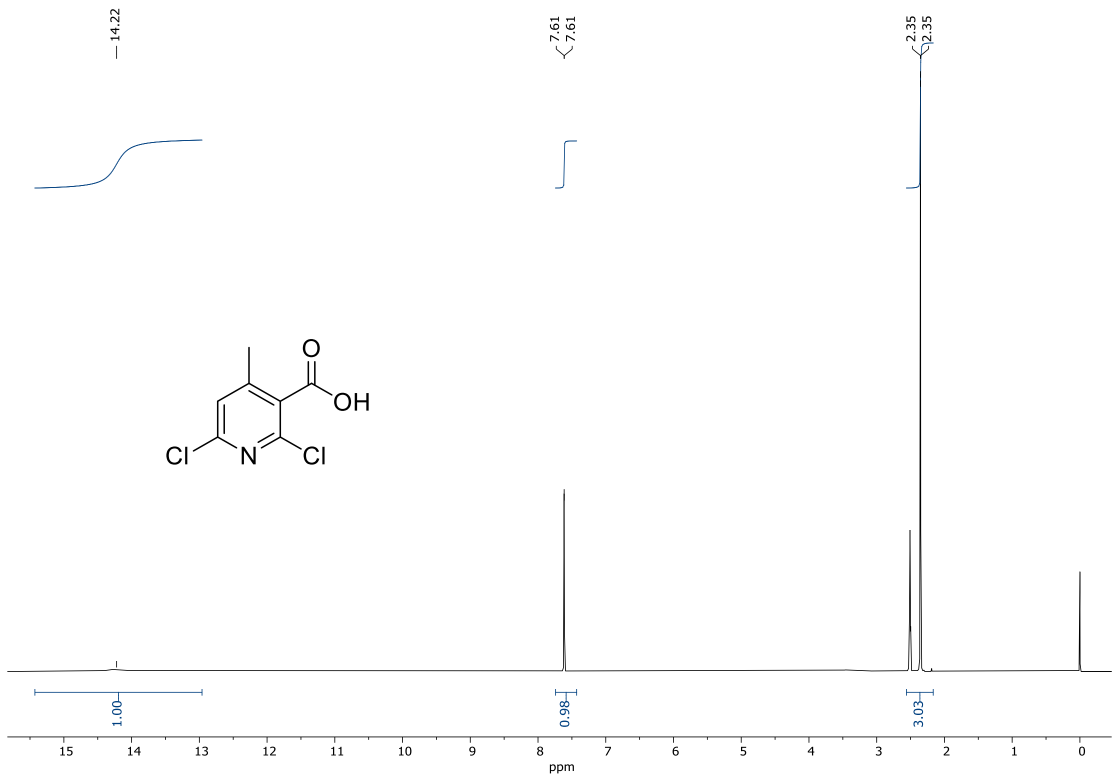

Figure S45: ^{1}H-NMR spectrum of compound **33**.

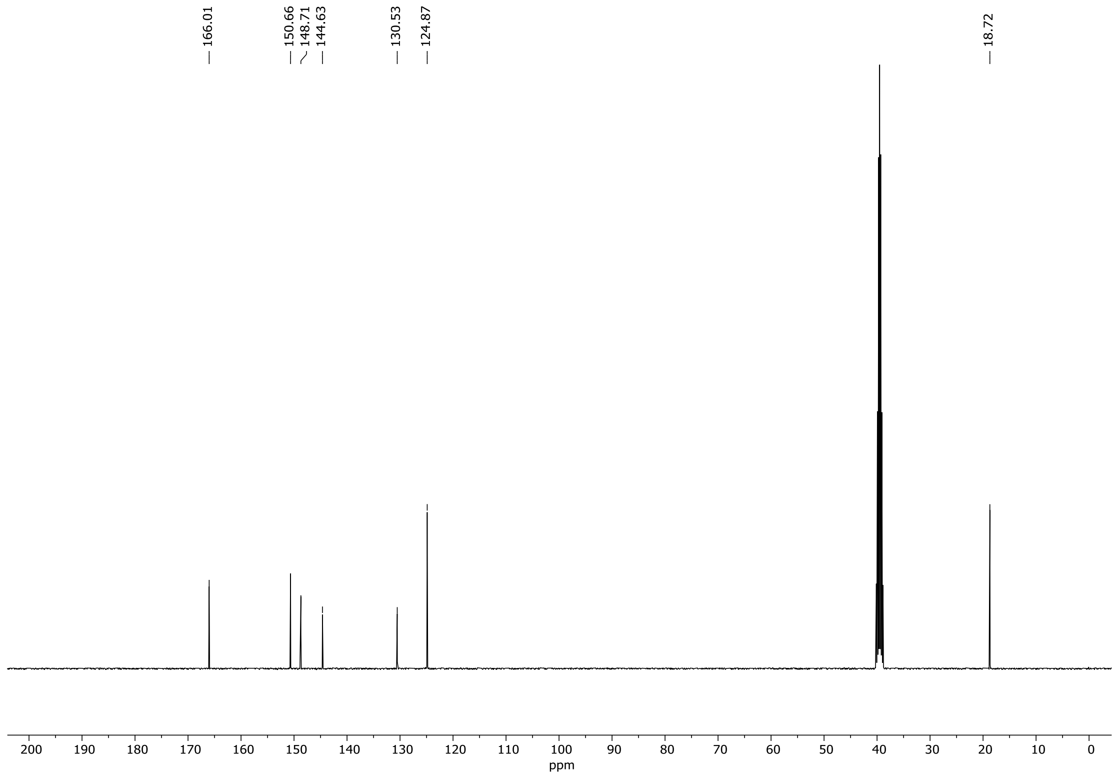

Figure S46: ^{13}C-NMR spectrum of compound **33**.

6-Chloro-2-methoxy-4-methylnicotinic acid (**34**)

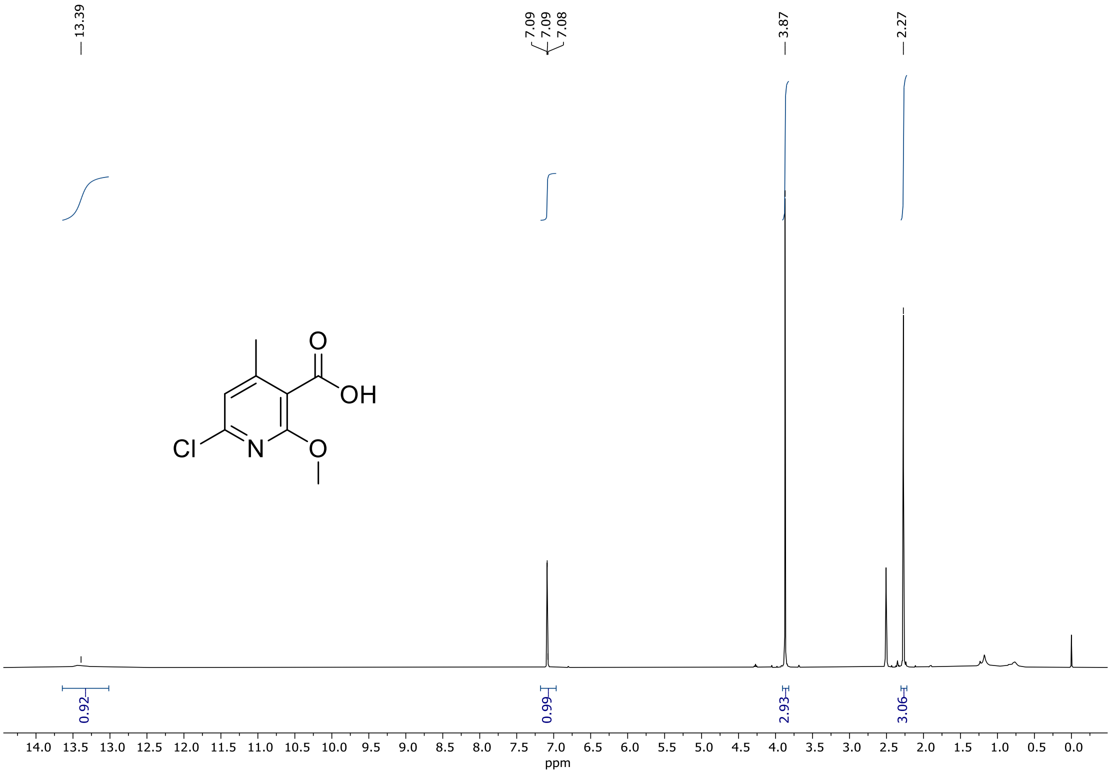

Figure S47: ^{1}H-NMR spectrum of compound **34**.

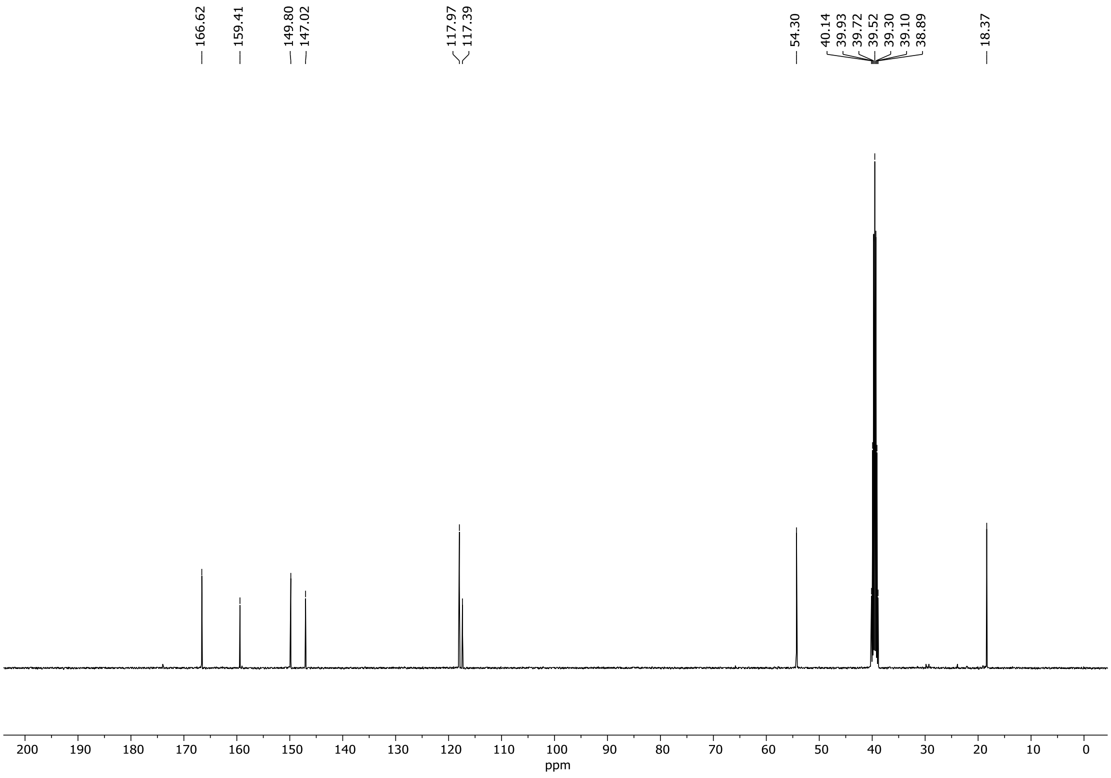

Figure S48: ^{13}C-NMR spectrum of compound **34**.

N-Butyl-6-chloro-2-methoxy-4-methylnicotinamide (**35a**)

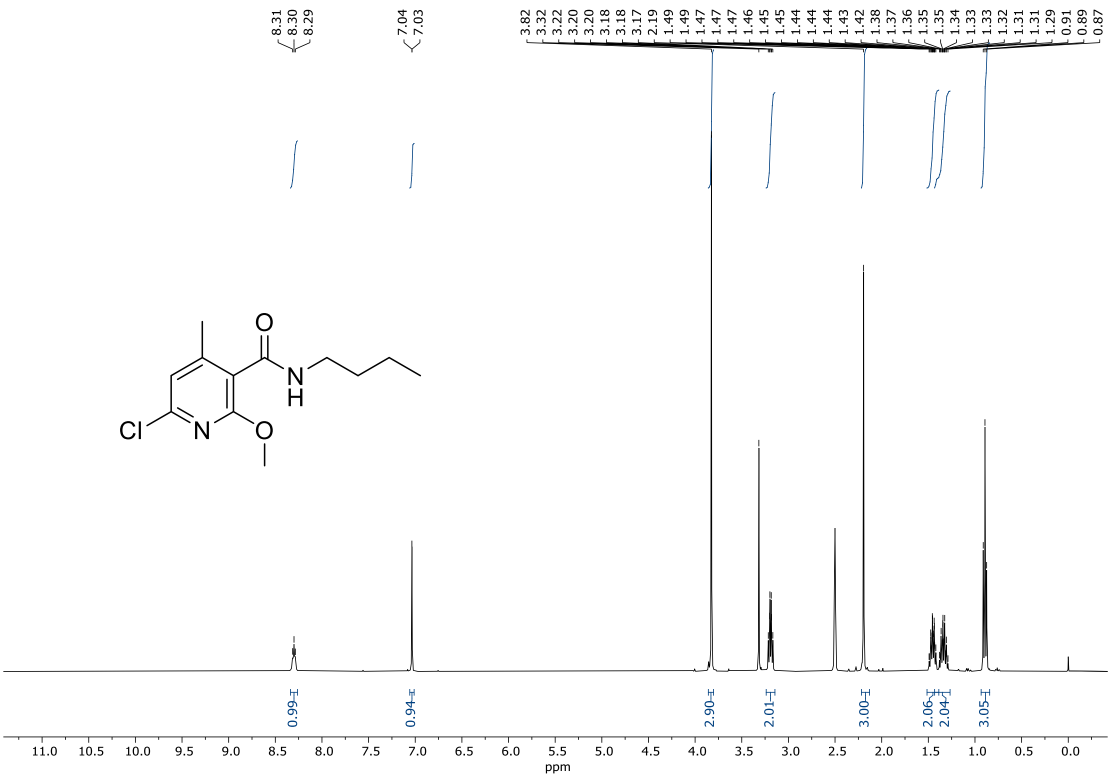

Figure S49: ^{1}H-NMR spectrum of compound **35a**.

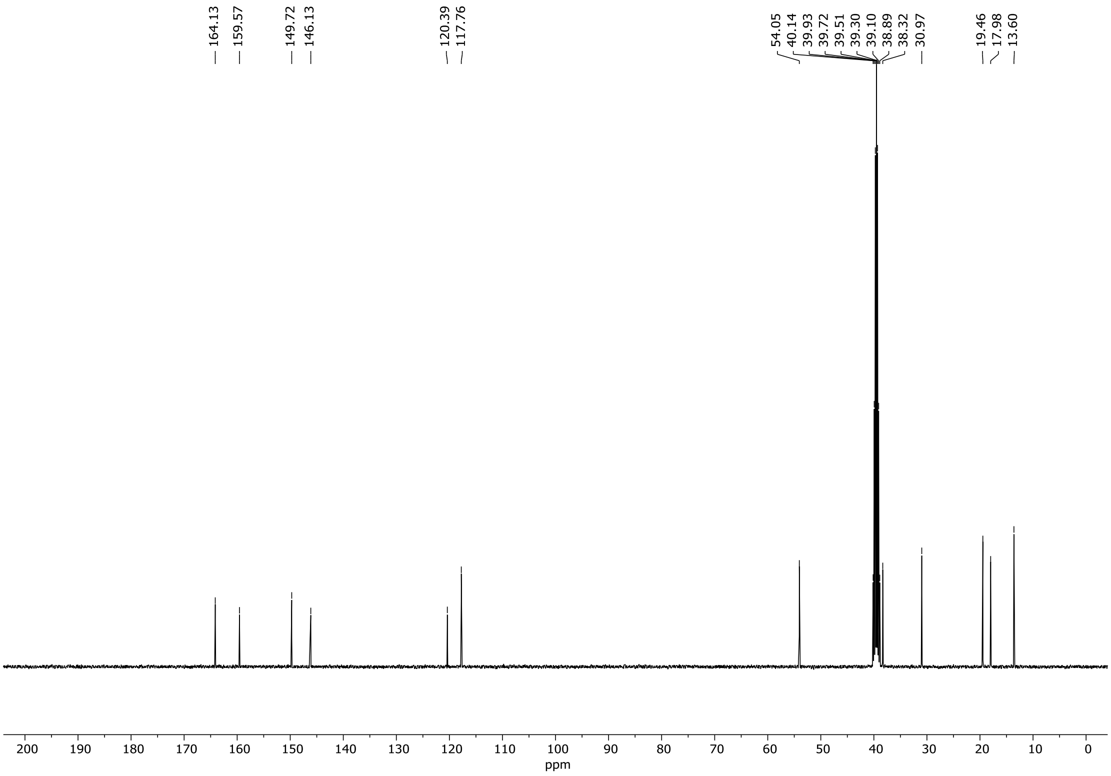

Figure S50: ^{13}C-NMR spectrum of compound **35a**.

6-Chloro-*N*-(4-fluorobenzyl)-2-methoxy-4-methylnicotinamide (**35b**)

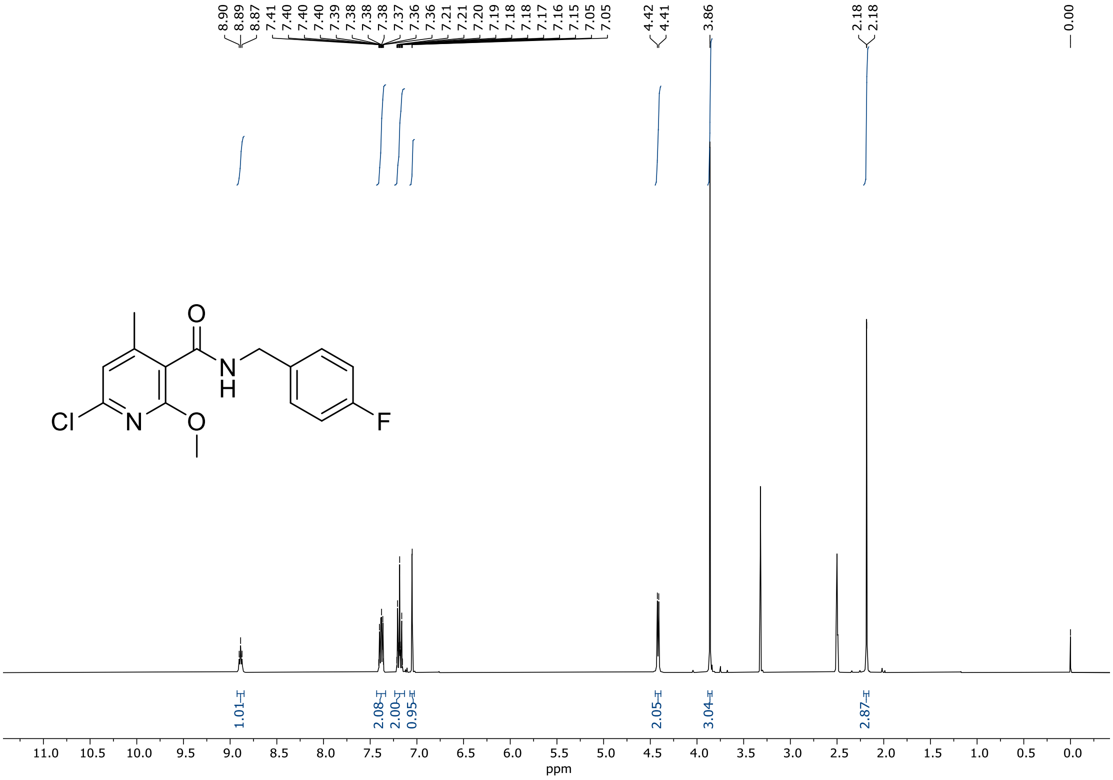

Figure S51: ^{1}H-NMR spectrum of compound **35b**.

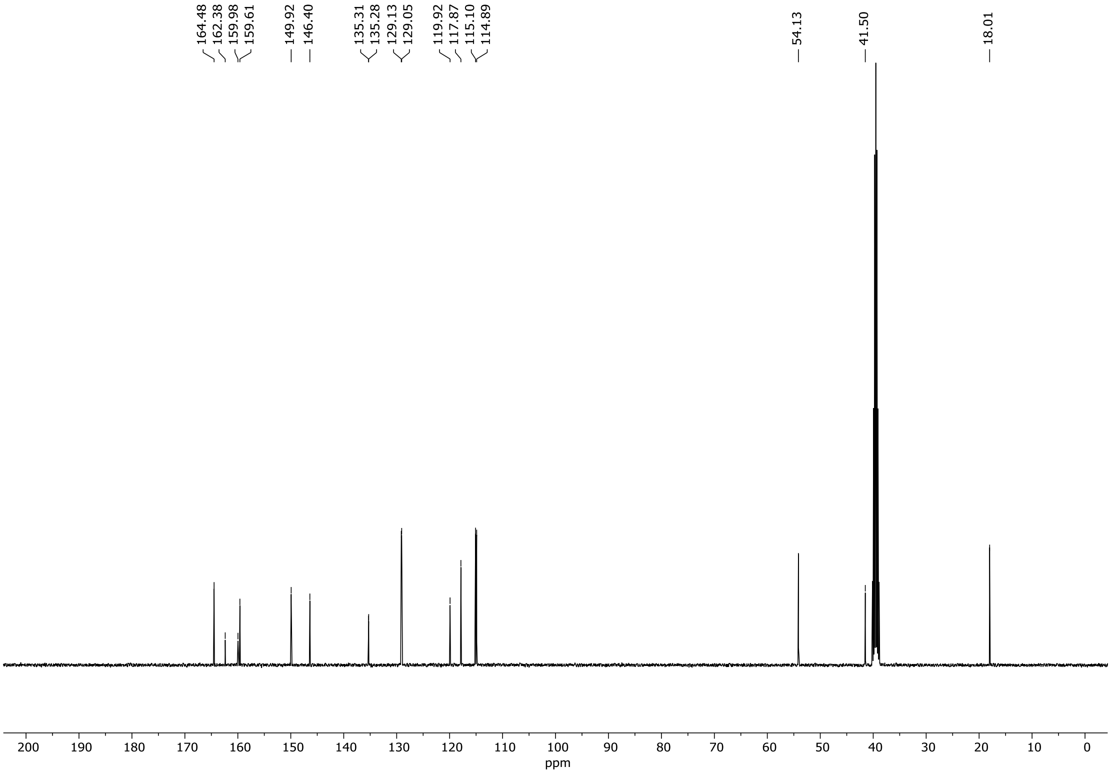

Figure S52: ^{13}C-NMR spectrum of compound **35b**.

N-Butyl-6-[(4-fluorobenzyl)amino]-2-methoxy-4-methylnicotinamide (**36a**)

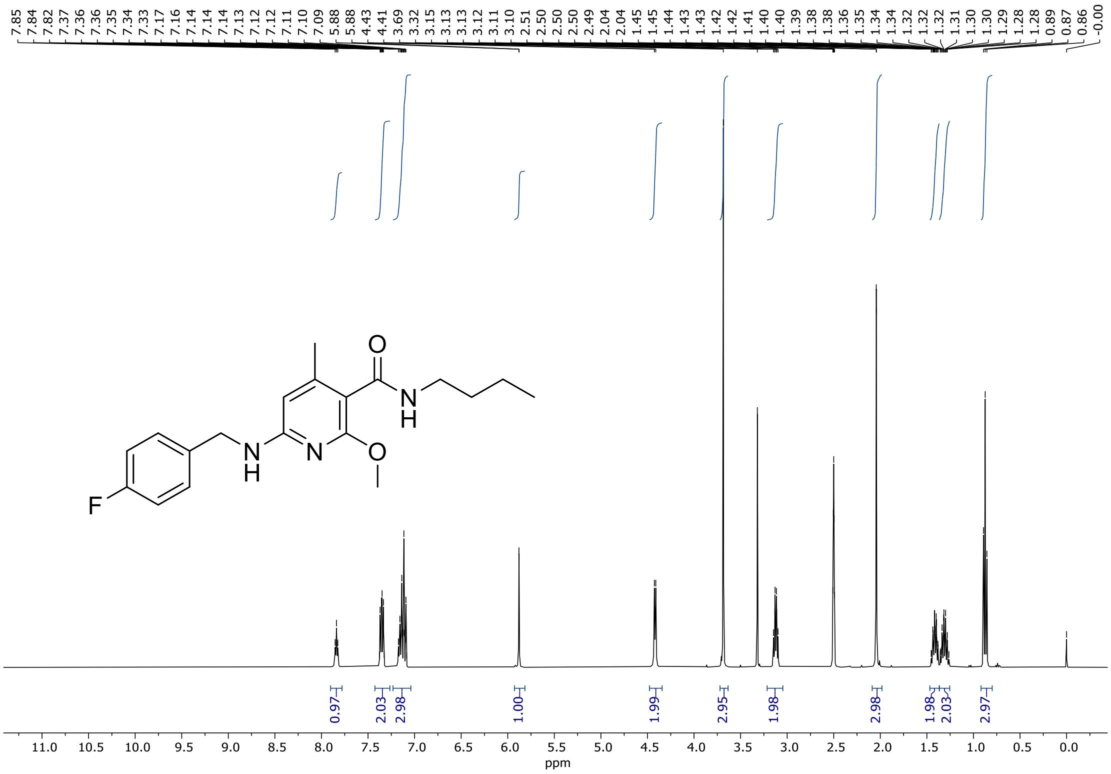

Figure S53: ^{1}H-NMR spectrum of compound **36a**.

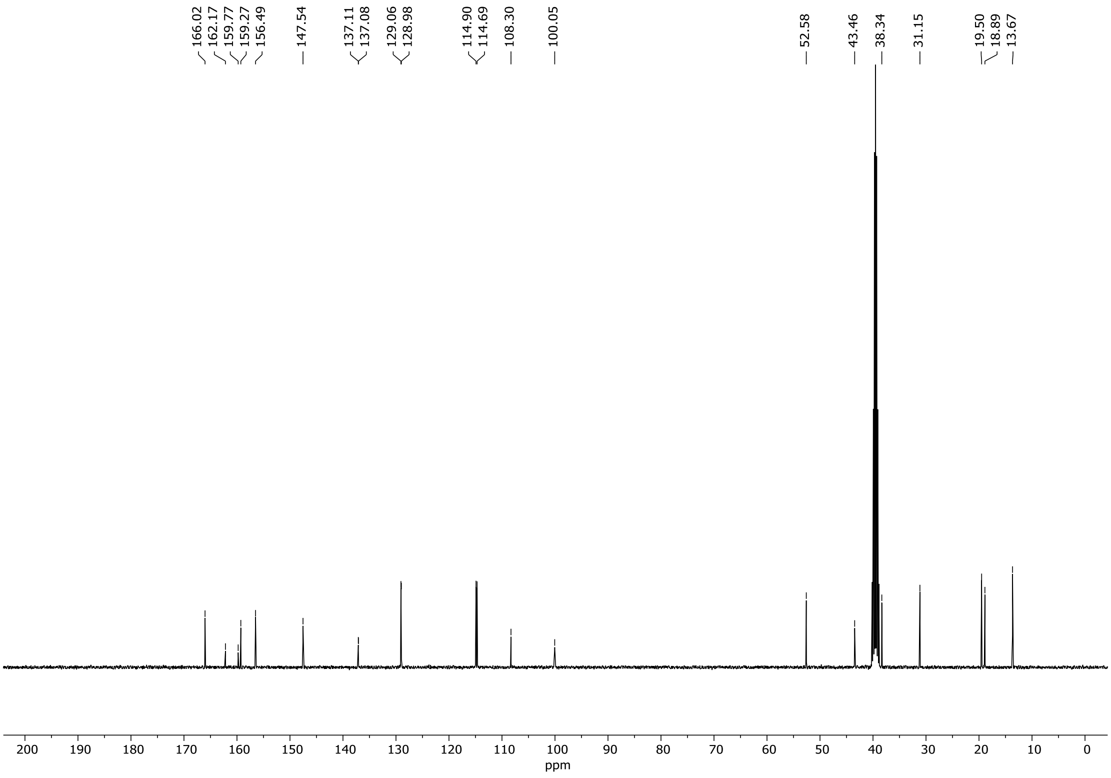

Figure S54: ^{13}C-NMR spectrum of compound **36a**.

N-(4-Fluorobenzyl)-6-[(4-fluorobenzyl)amino]-2-methoxy-4-methylnicotinamide (**36b**)

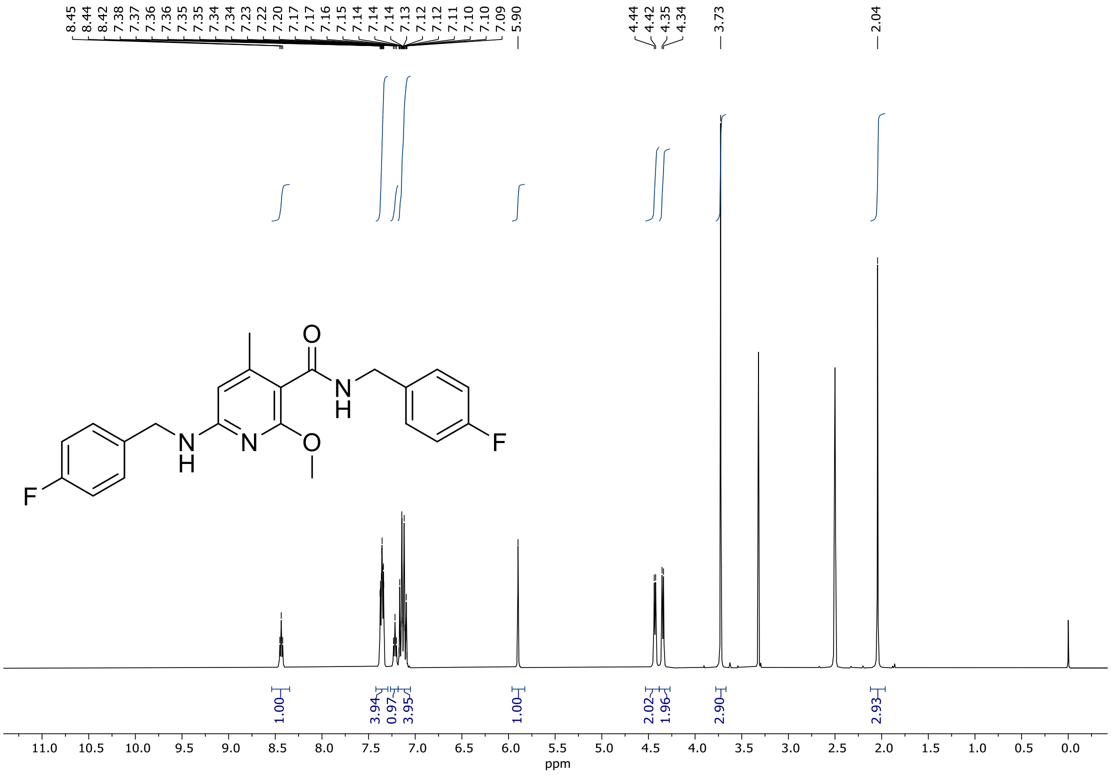

Figure S55: ^{1}H-NMR spectrum of compound **36b**.

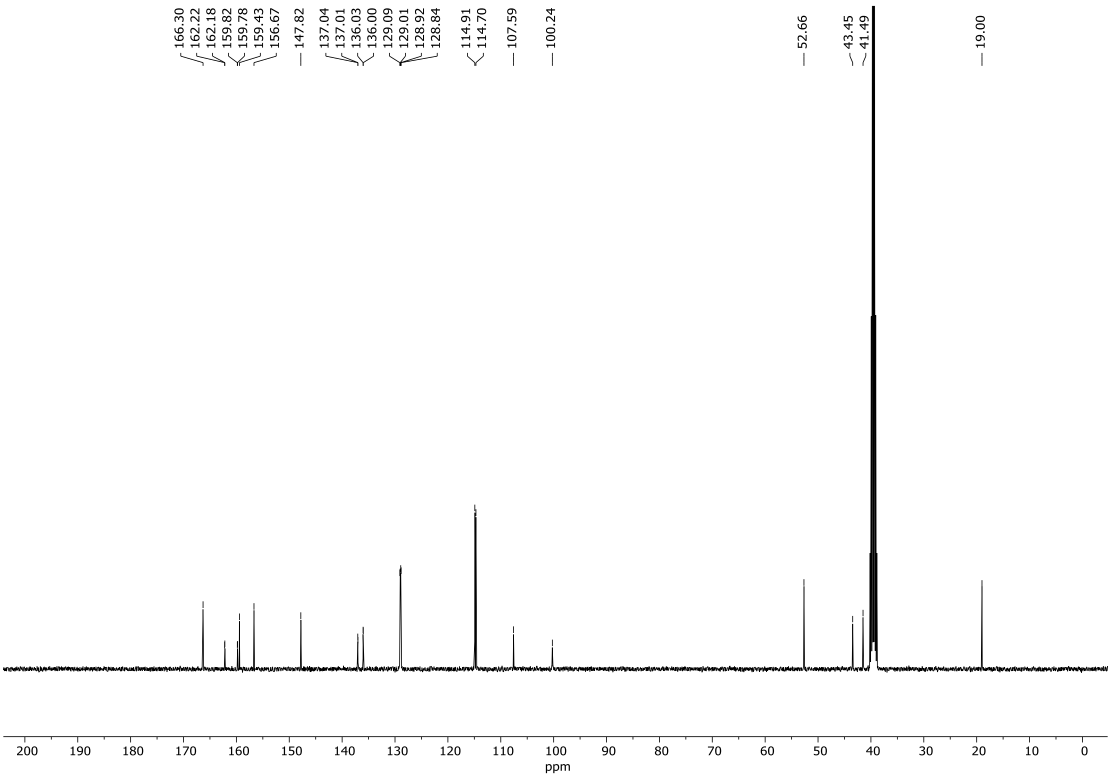

Figure S56: ^{13}C-NMR spectrum of compound **36b**.

N-(4-Fluorobenzyl)-6-[(4-fluorobenzyl)amino]-4-methyl-2-oxo-1,2-dihydropyridine-3-carboxamide (**37**)

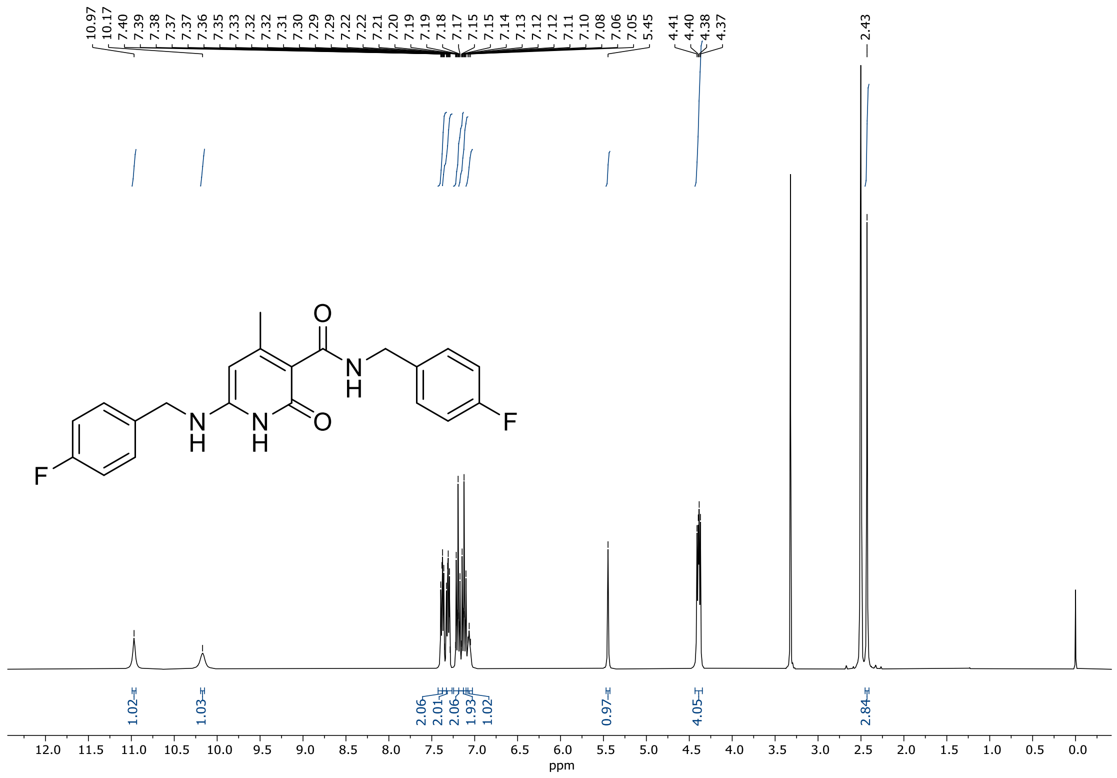

Figure S57: ^{1}H-NMR spectrum of compound **37**.

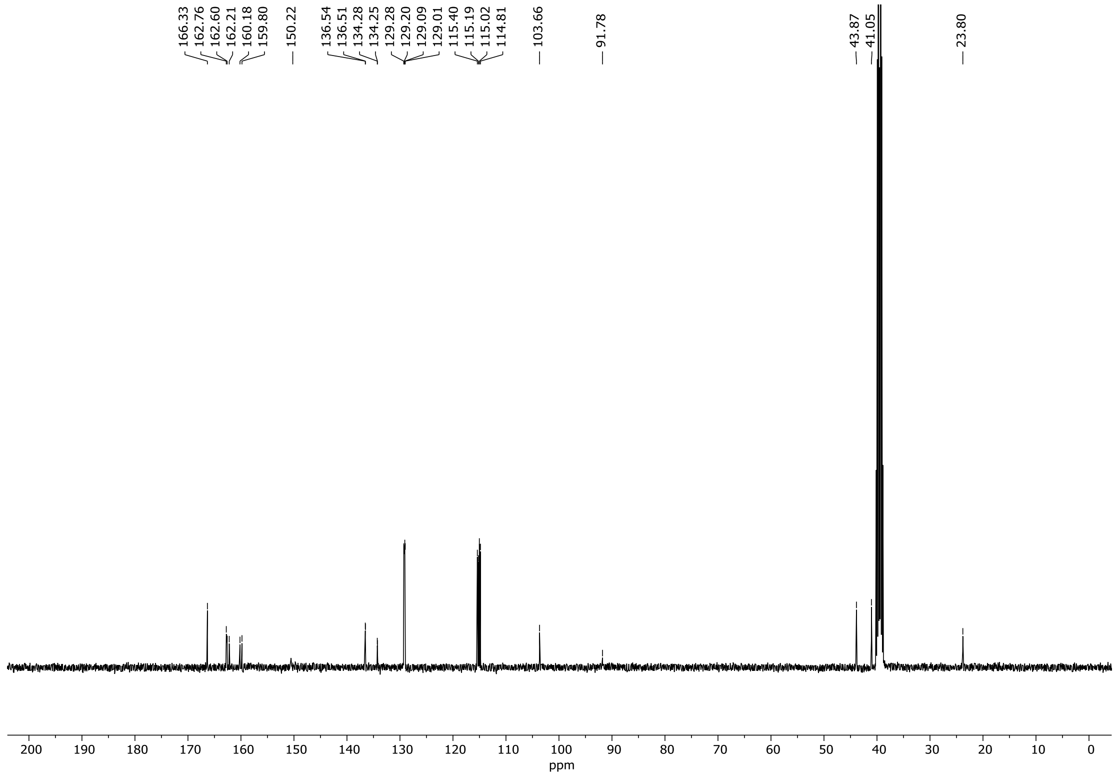

Figure S58: ^{13}C-NMR spectrum of compound **37**.

Methyl 4-amino-2-bromobenzoate (**39**)

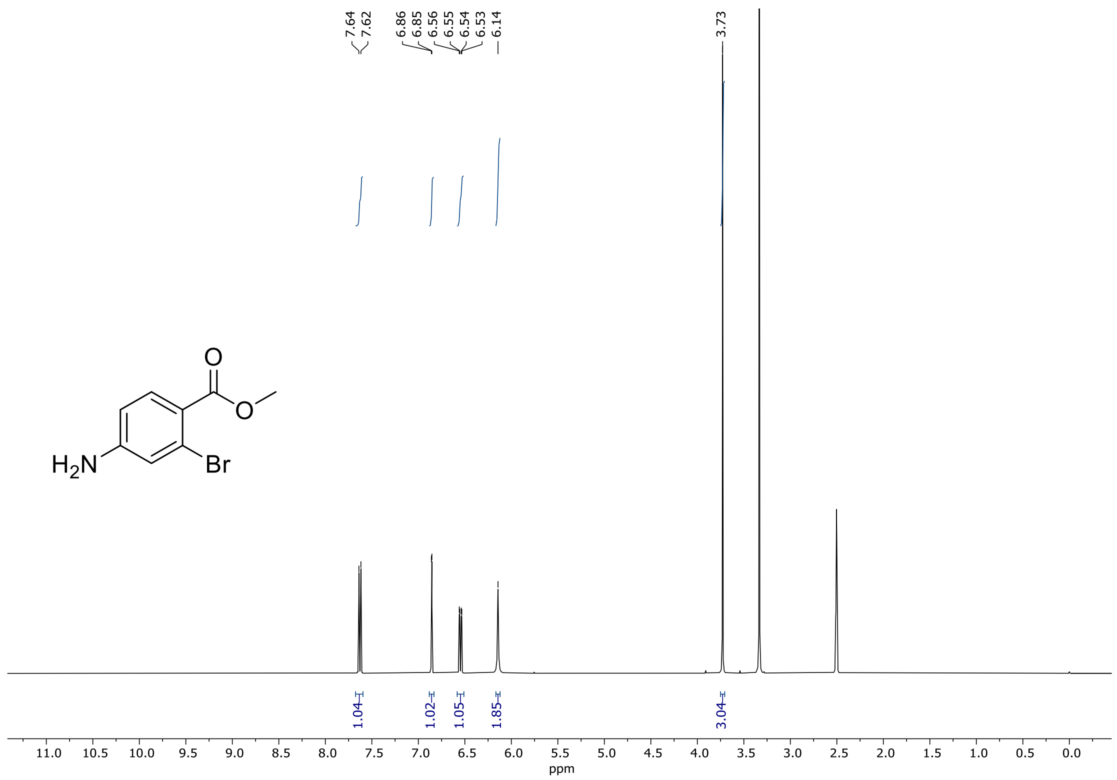

Figure S59: ^{1}H-NMR spectrum of compound **39**.

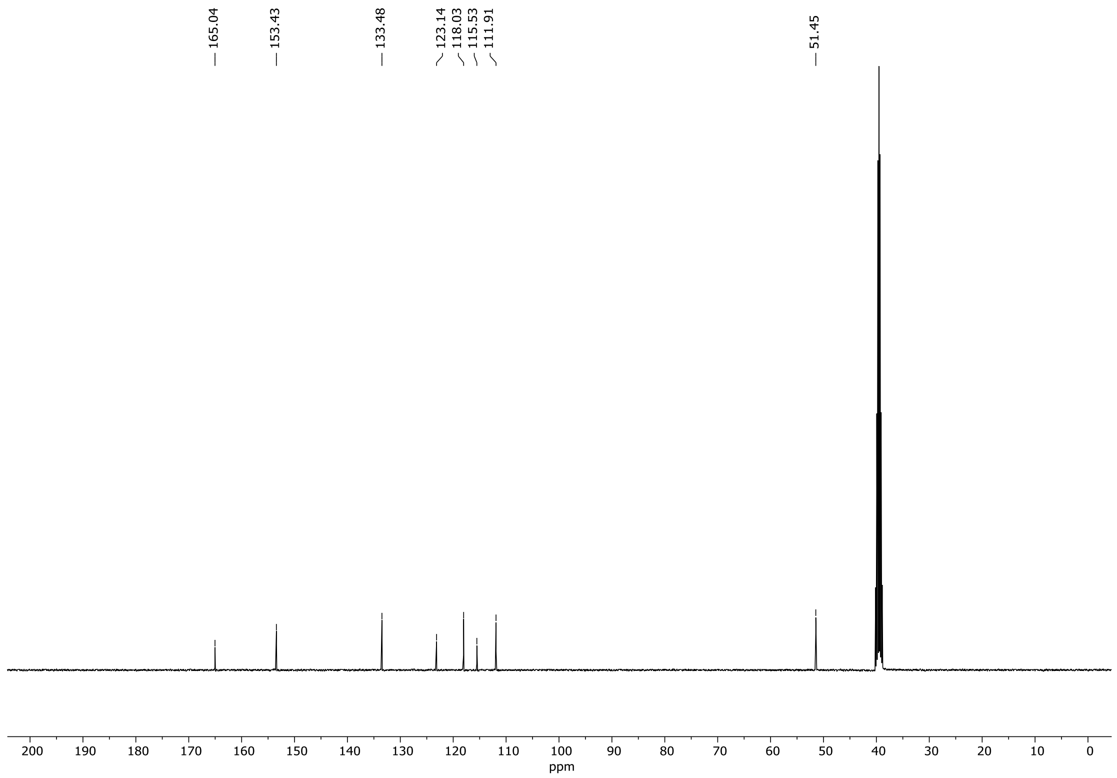

Figure S60: ^{13}C-NMR spectrum of compound **39**.

Methyl 2-bromo-4-[(4-fluorobenzyl)amino]benzoate (**40**)

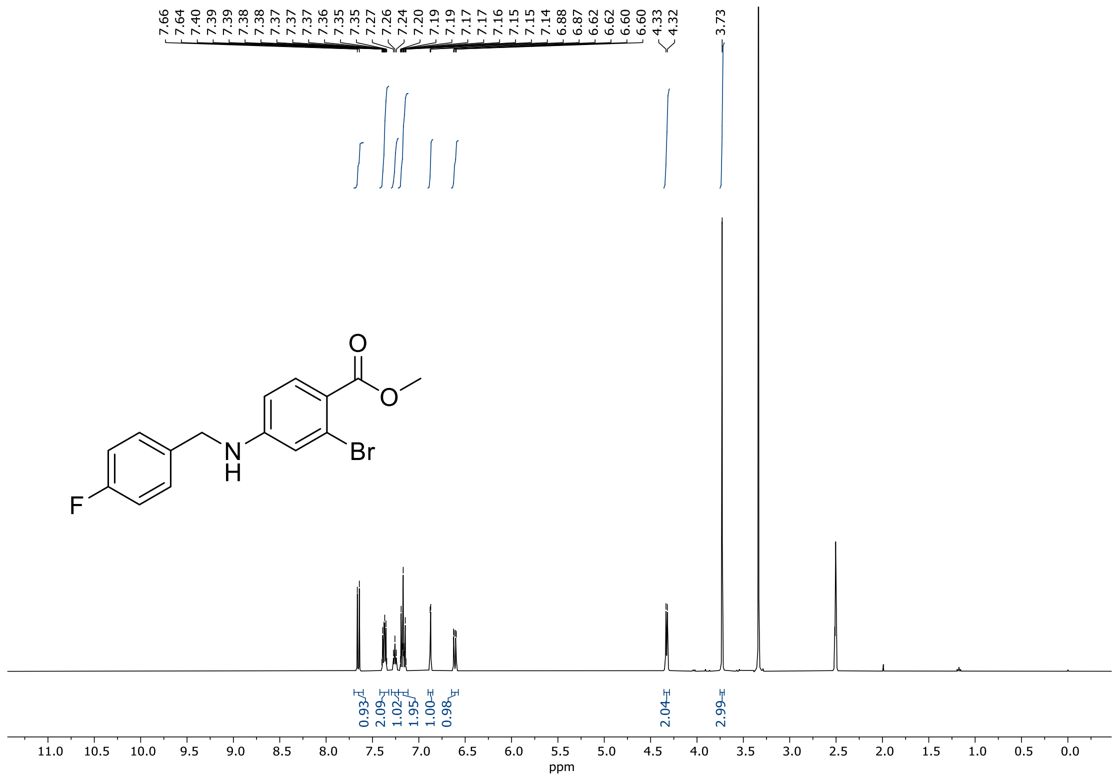

Figure S61: ^{1}H-NMR spectrum of compound **40**.

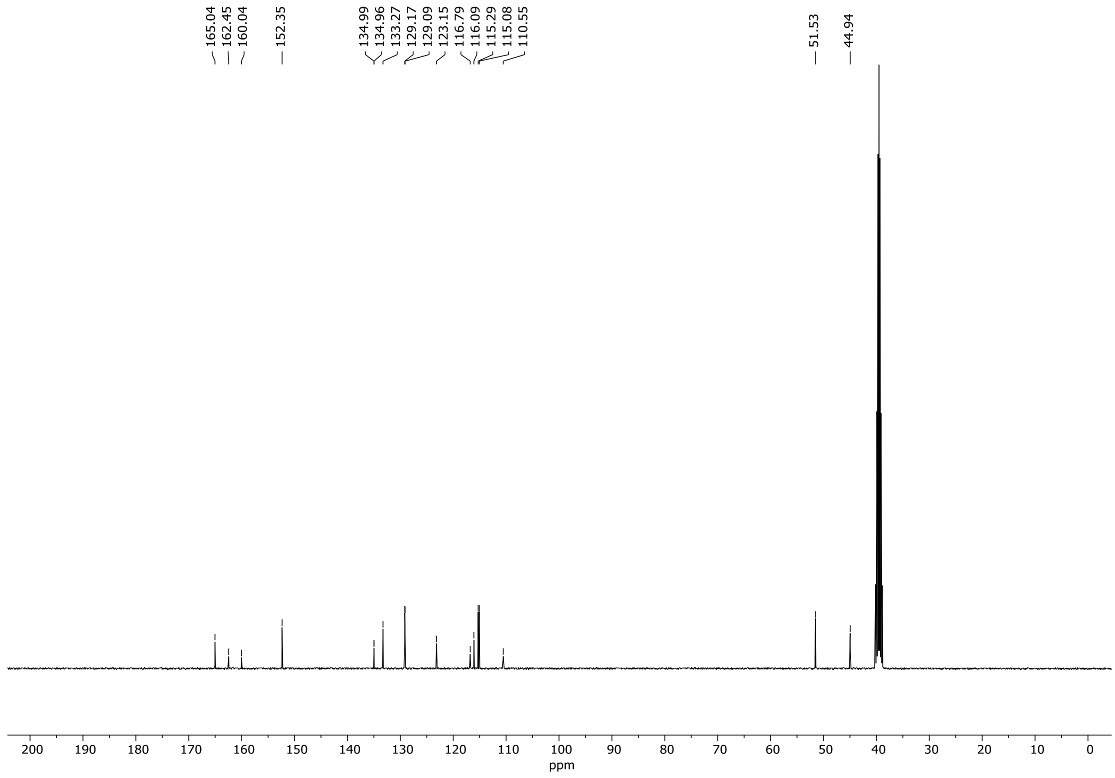

Figure S62: ^{13}C-NMR spectrum of compound **40**.

Methyl 2-bromo-4-[(*tert*-butoxycarbonyl)(4-fluorobenzyl)amino]benzoate (**41**)

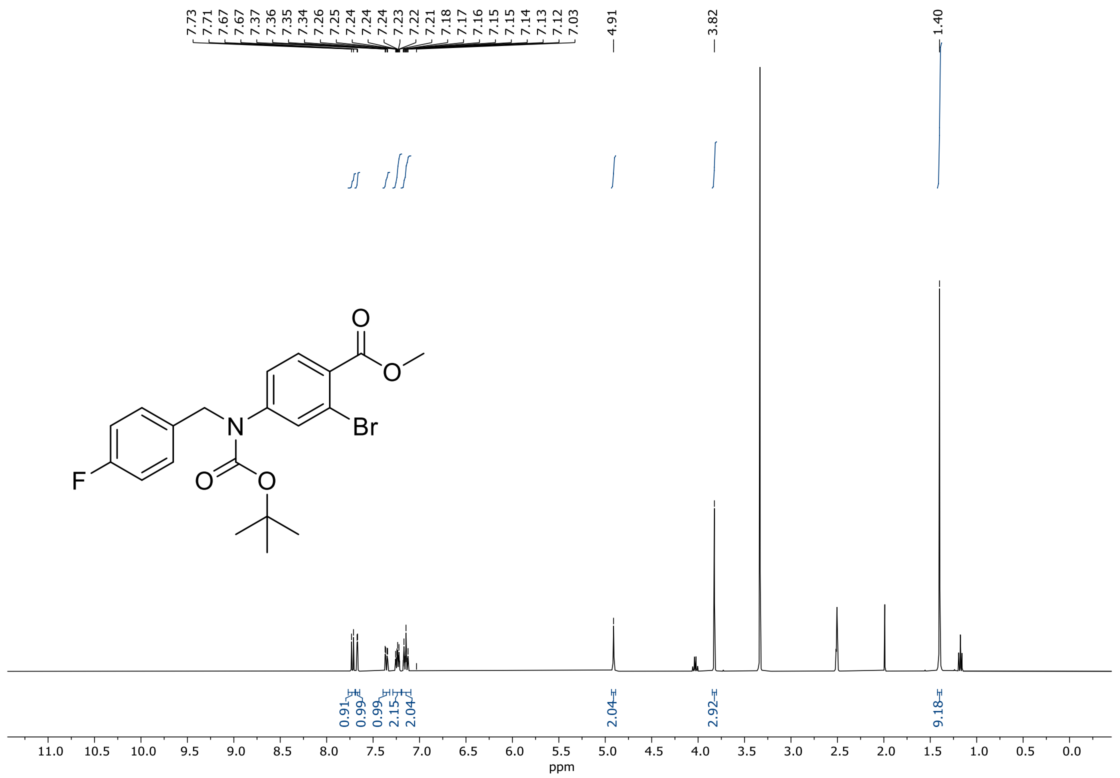

Figure S63: ^{1}H-NMR spectrum of compound **41**.

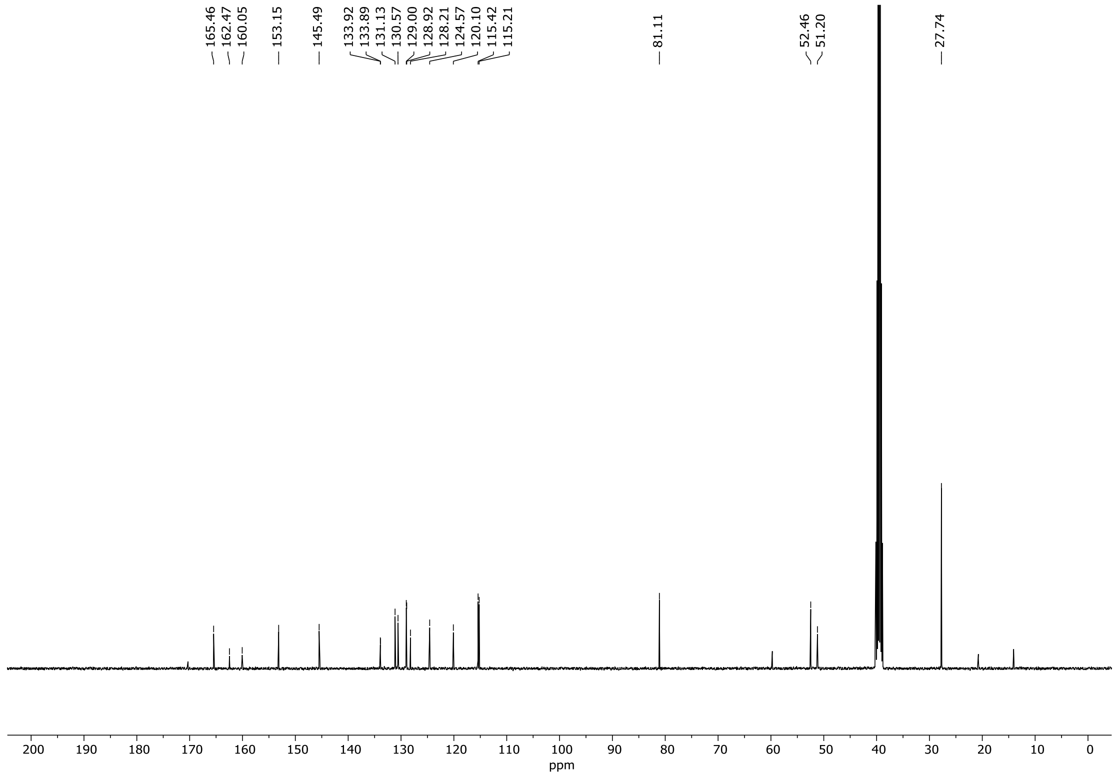

Figure S64: ^{13}C-NMR spectrum of compound **41**.

Methyl 4-[(*tert*-butoxycarbonyl)(4-fluorobenzyl)amino]-2-[(trimethylsilyl)ethynyl]benzoate (**42**)

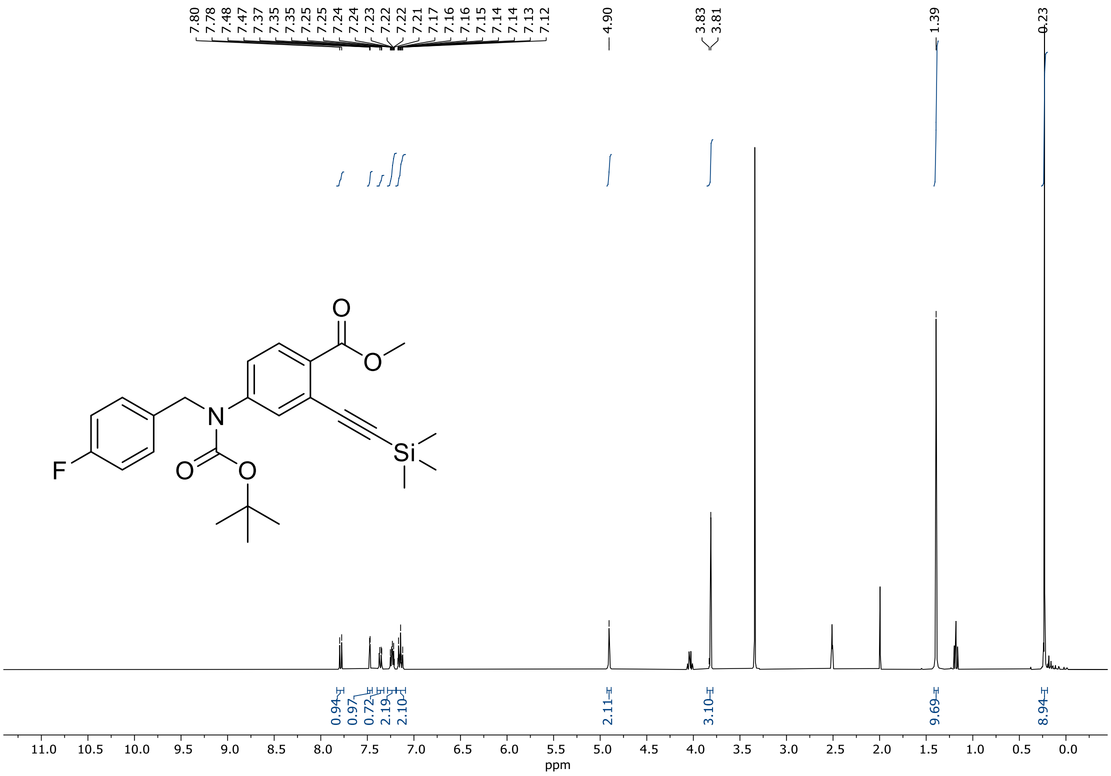

Figure S65: ^{1}H-NMR spectrum of compound **42**.

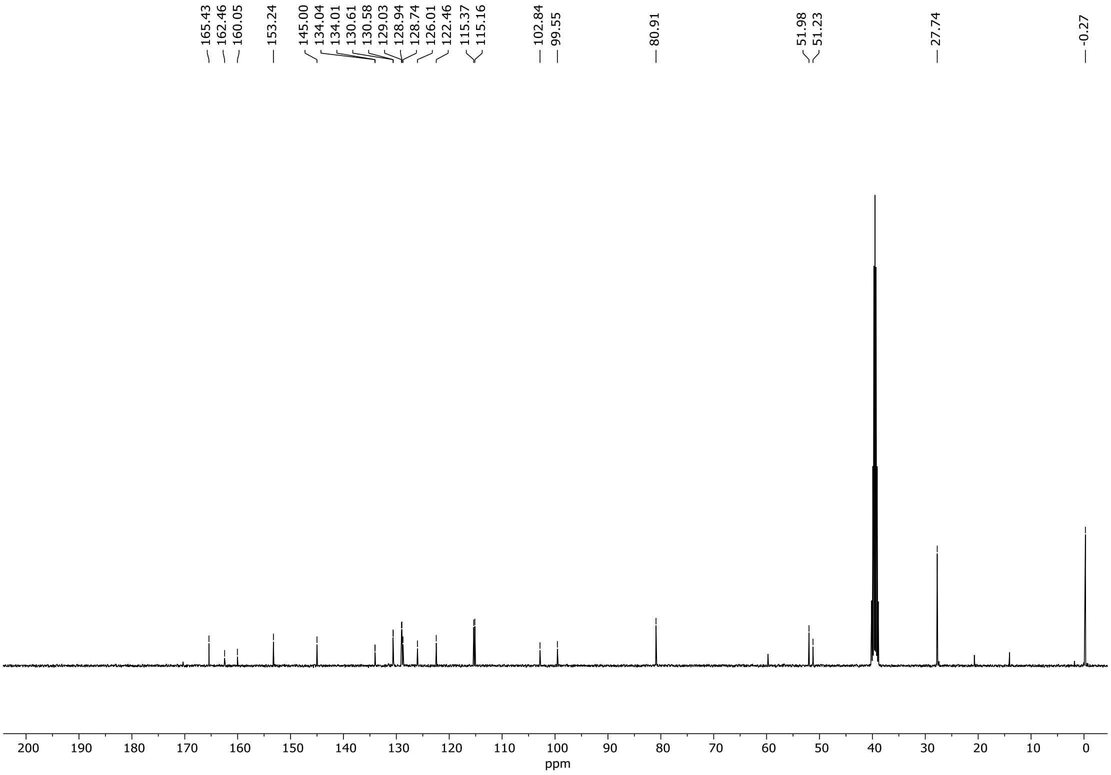

Figure S66: ^{13}C-NMR spectrum of compound **42**.

Methyl 4-[(*tert*-butoxycarbonyl)(4-fluorobenzyl)amino]-2-ethynylbenzoate (**43**)

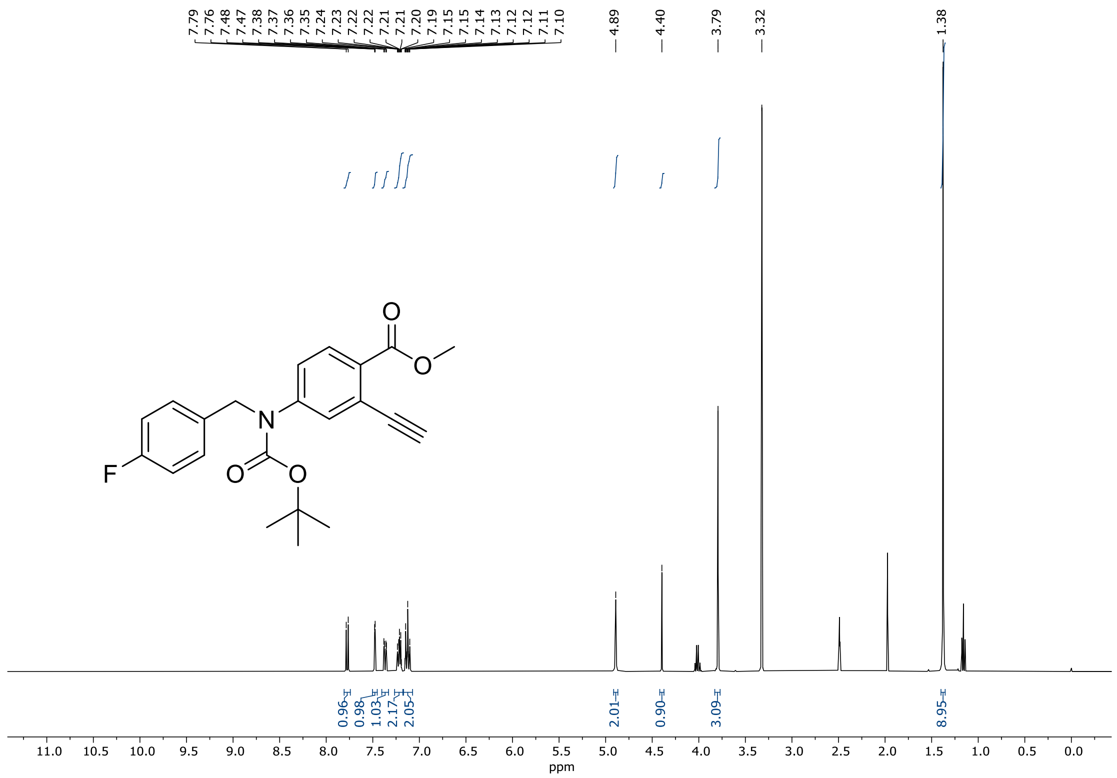

Figure S67: ^{1}H-NMR spectrum of compound **43**.

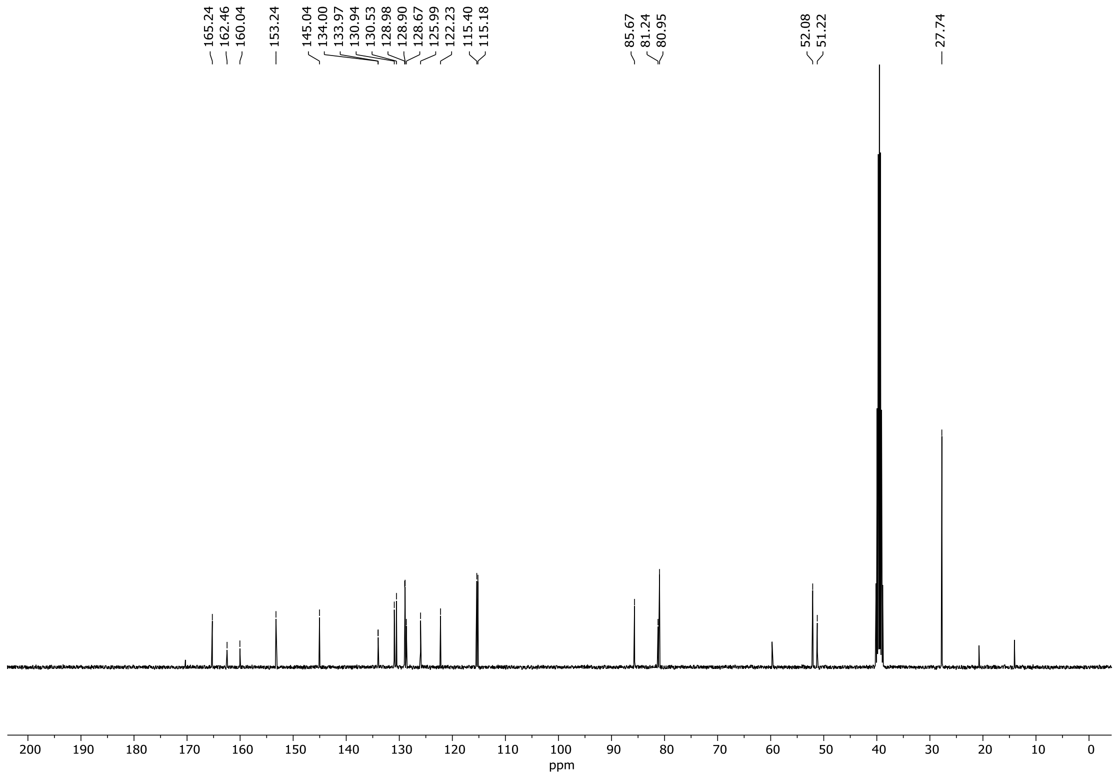

Figure S68: ^{13}C-NMR spectrum of compound **43**.

Methyl 4-[(*tert*-butoxycarbonyl)(4-fluorobenzyl)amino]-2-ethylbenzoate (**44**)

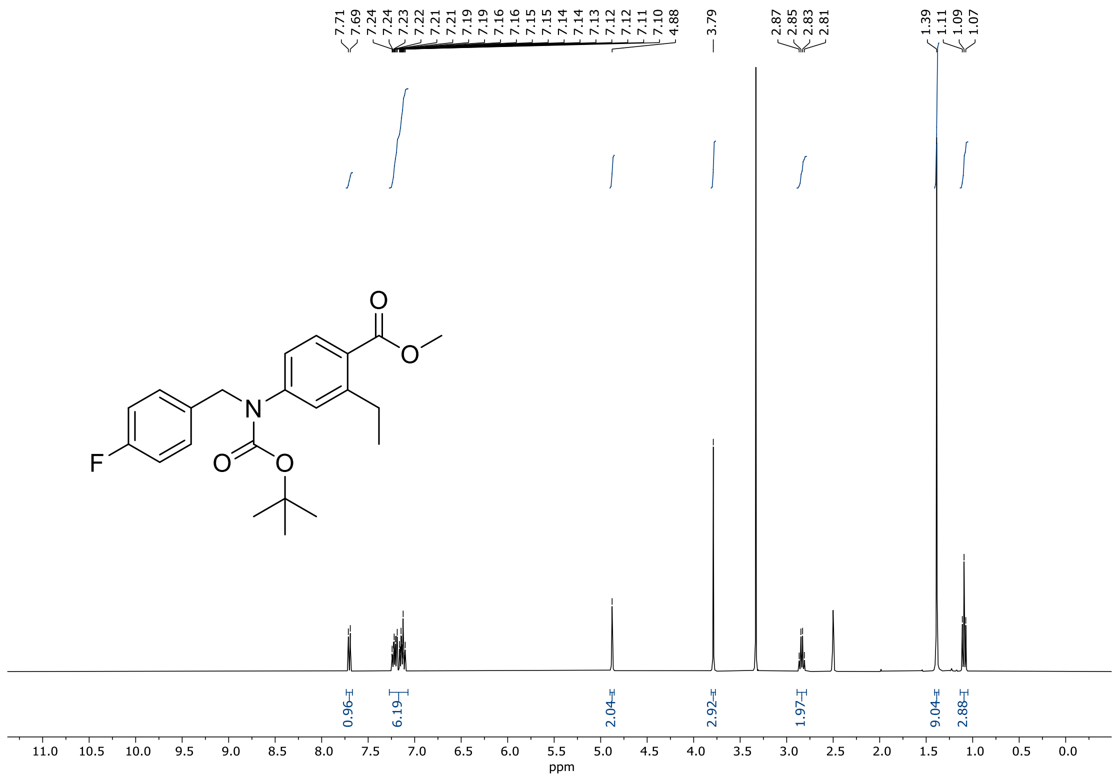

Figure S69: ^{1}H-NMR spectrum of compound **44**.

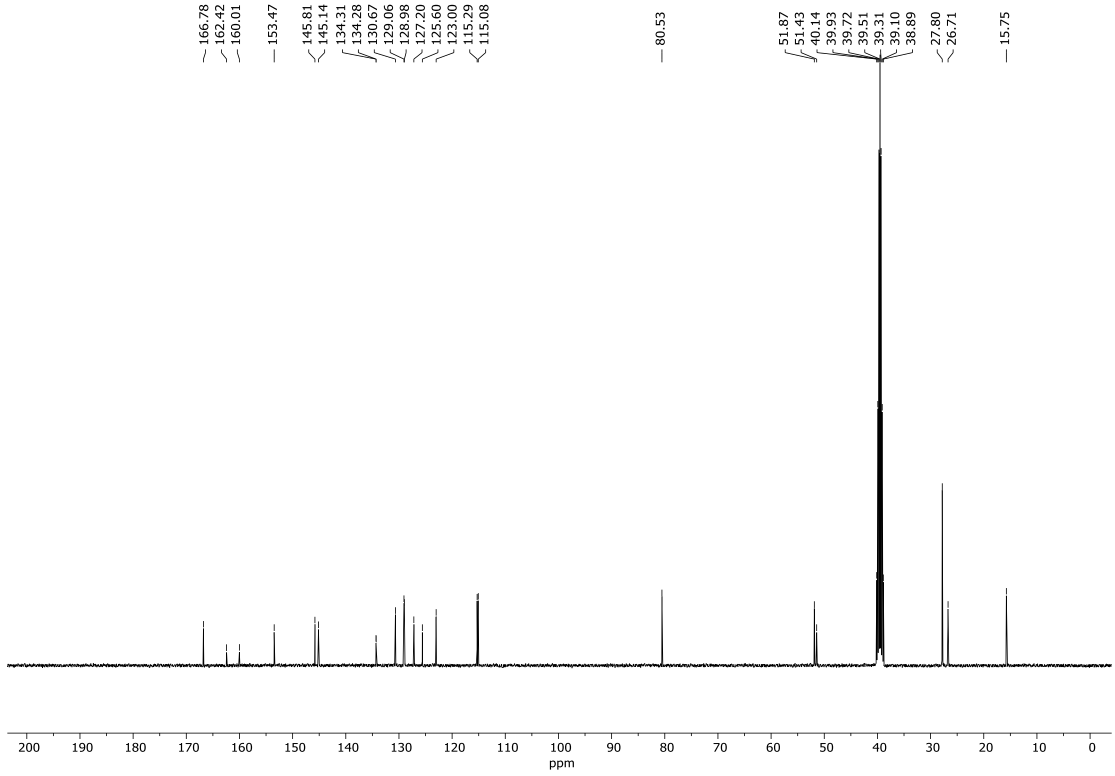

Figure S70: ^{13}C-NMR spectrum of compound **44**.

4-[(*tert*-Butoxycarbonyl)(4-fluorobenzyl)amino]-2-ethylbenzoic acid (**45**)

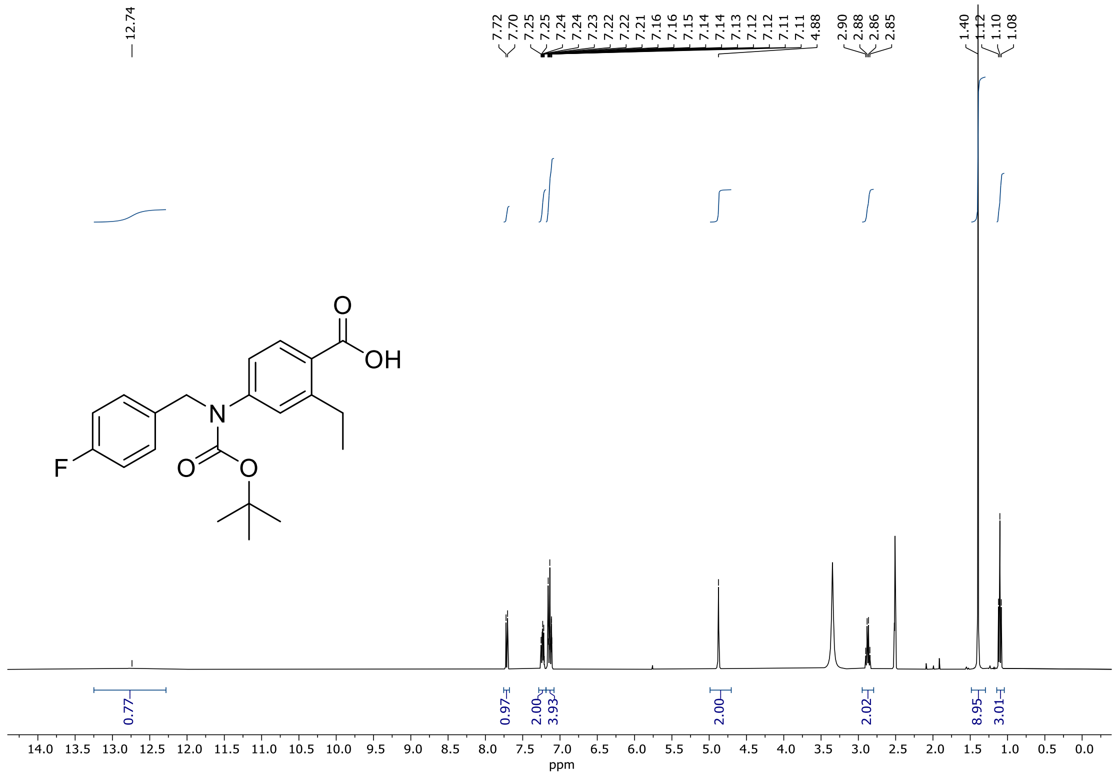

Figure S71: ^{1}H-NMR spectrum of compound **45**.

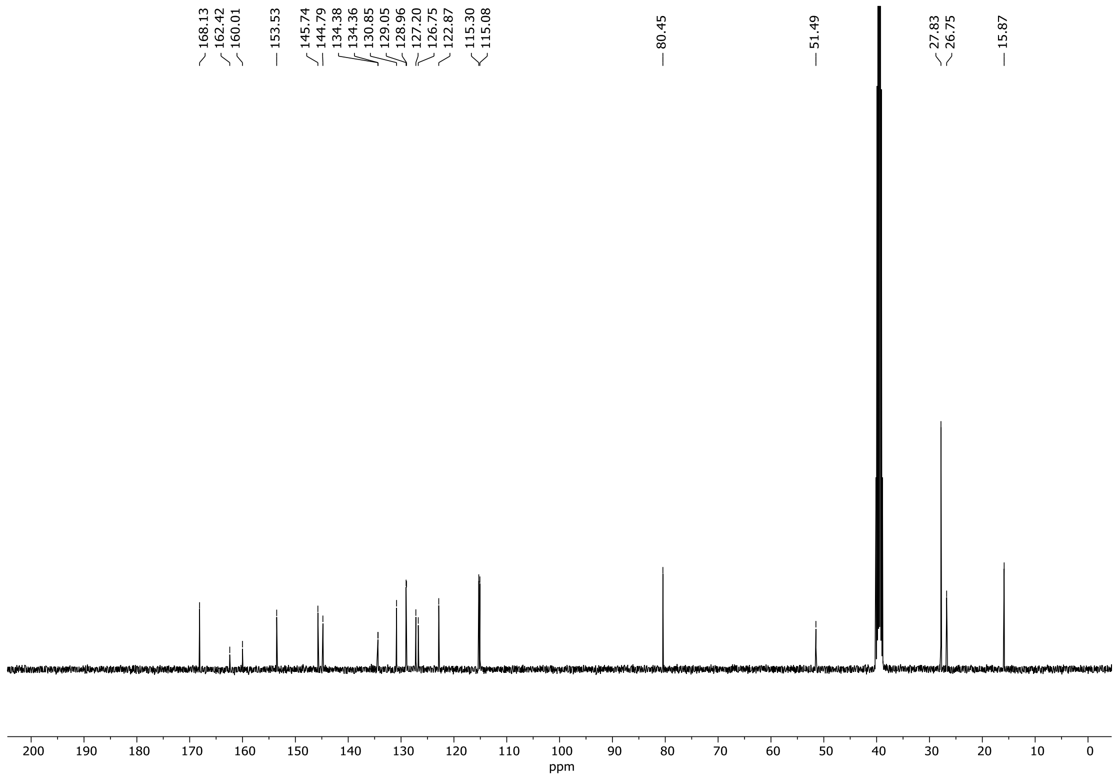

Figure S72: ^{13}C-NMR spectrum of compound **45**.

tert-Butyl [3-ethyl-4-(propylcarbamoyl)phenyl](4-fluorobenzyl)carbamate (**46**)

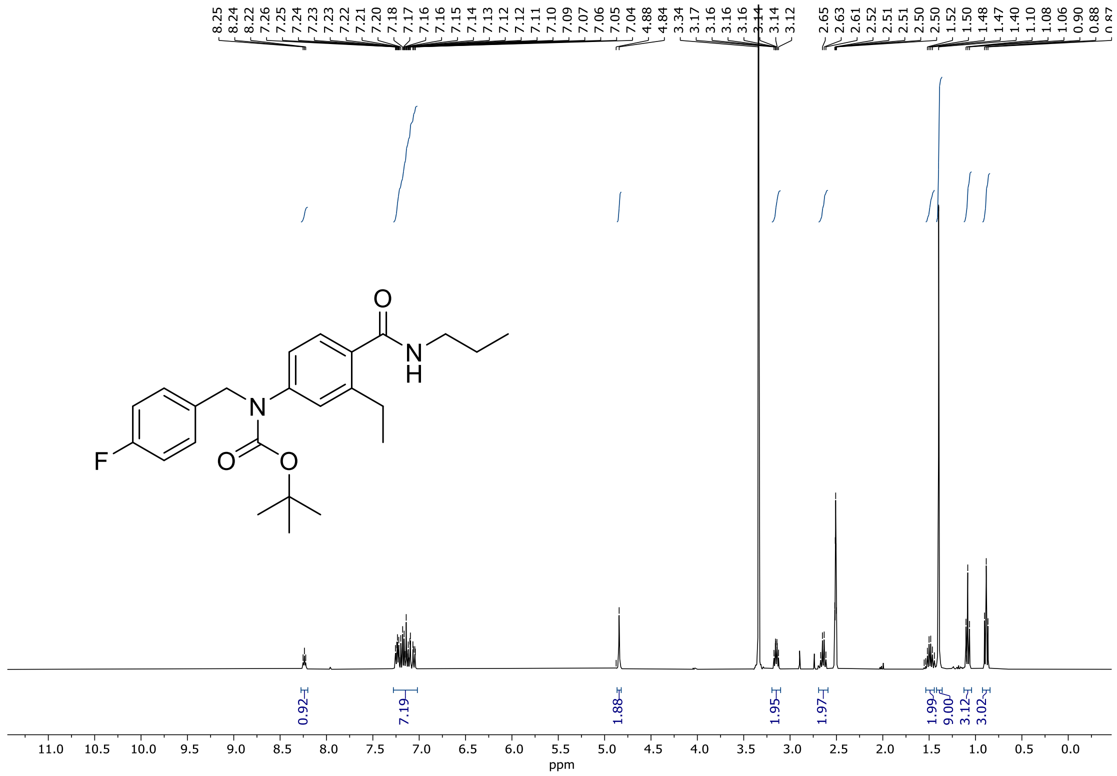

Figure S73: ^{1}H-NMR spectrum of compound **46**.

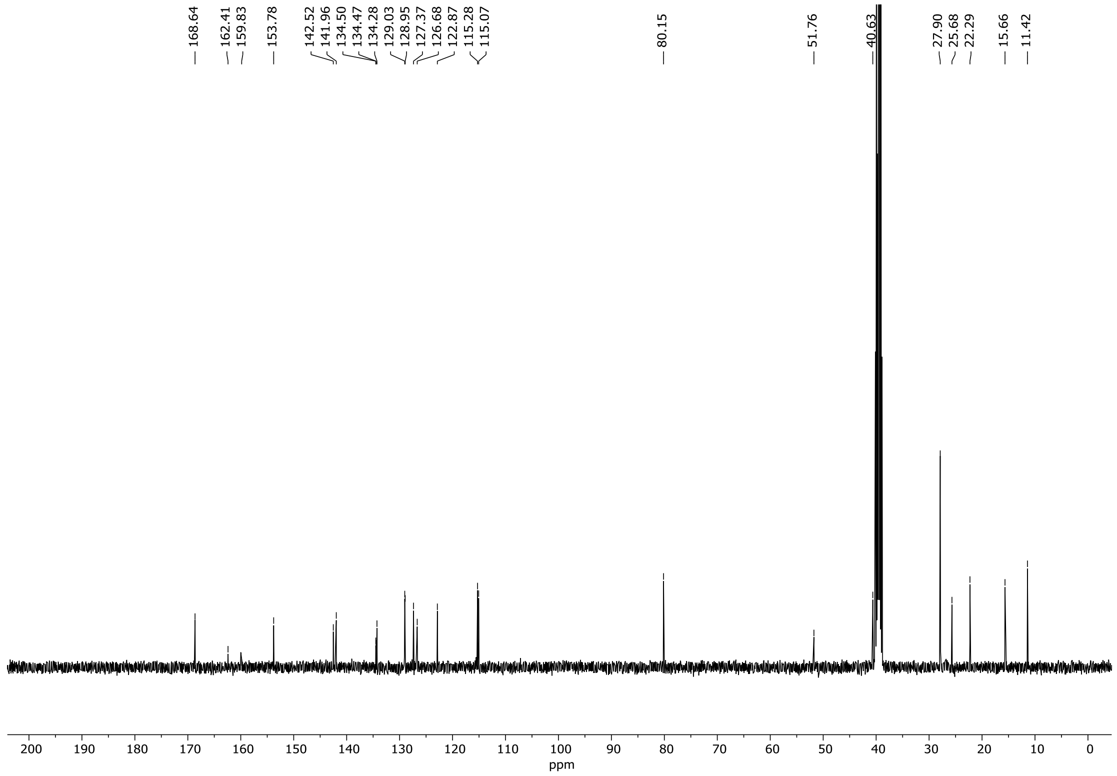

Figure S74: ^{13}C-NMR spectrum of compound **46**.

2-Ethyl-4-[(4-fluorobenzyl)amino]-*N*-propylbenzamide hydrochloride (**47**)

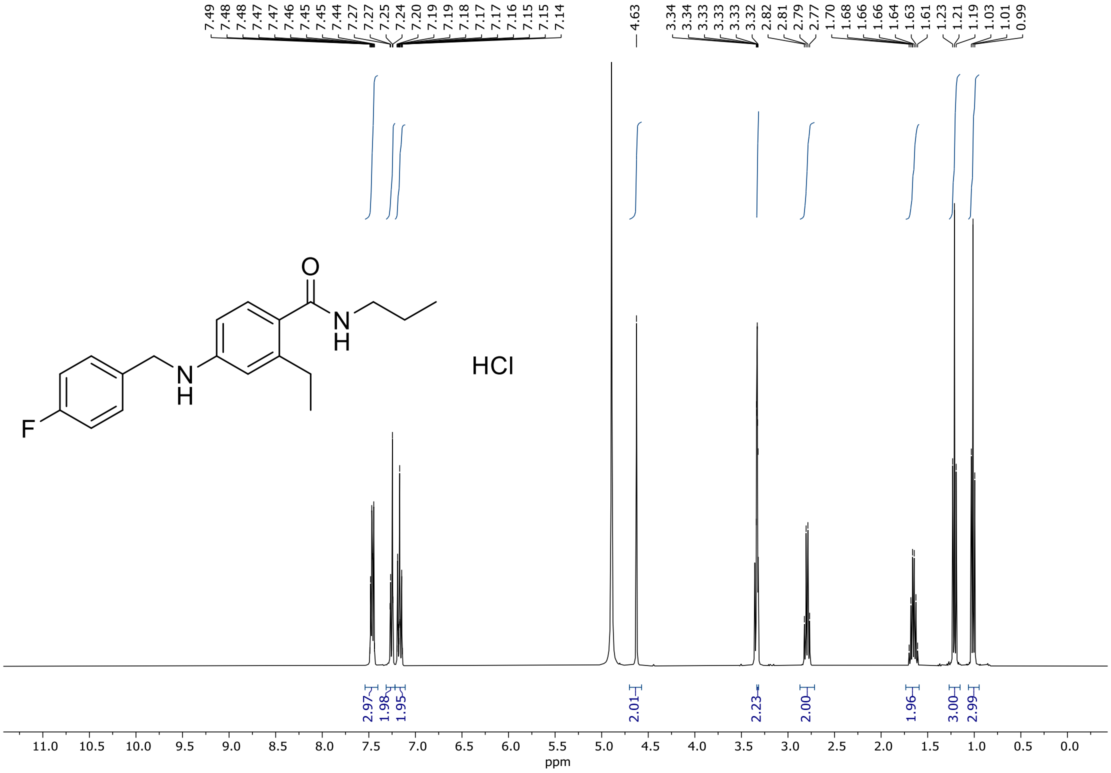

Figure S75: ^{1}H-NMR spectrum of compound **47**.

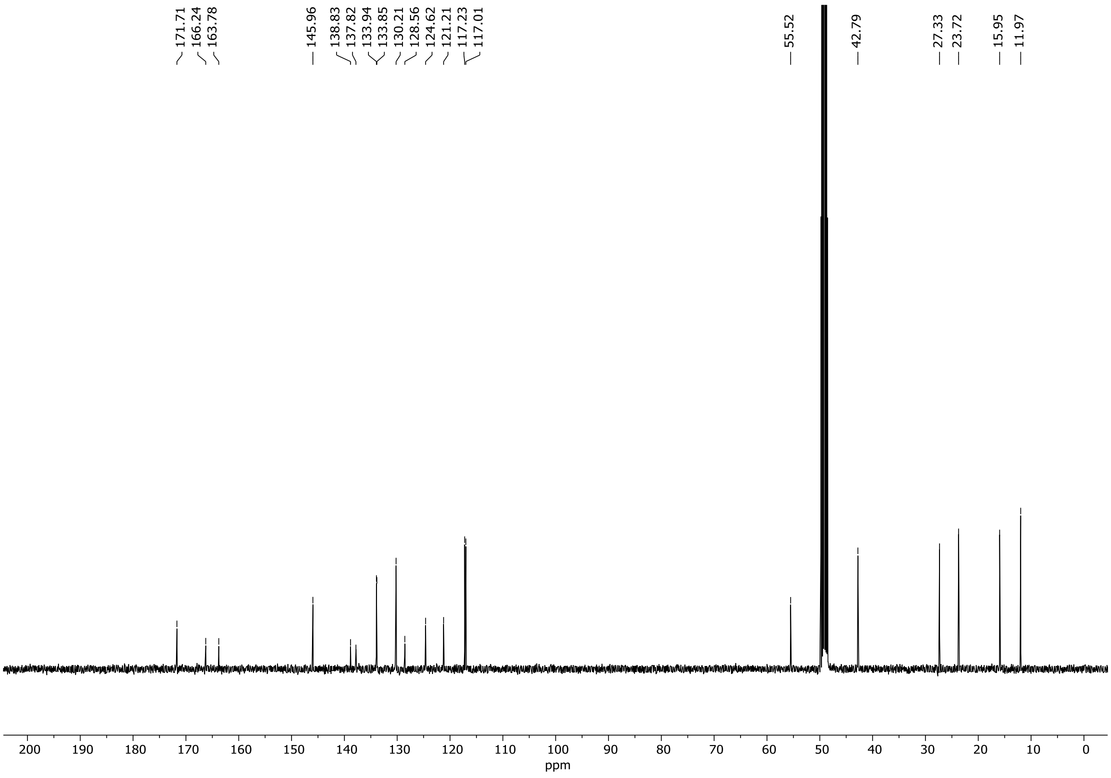

Figure S76: ^{13}C-NMR spectrum of compound **47**.

4-Methyl-2,6-dimorpholinonicotinonitrile (**53**)

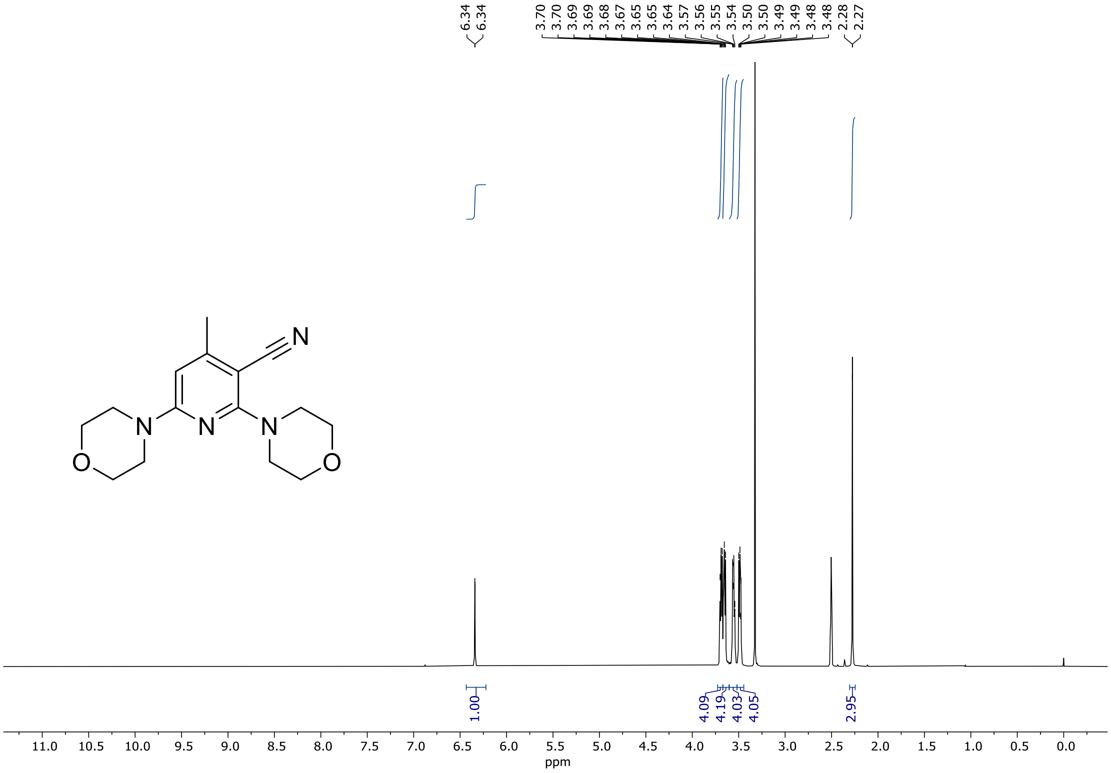

Figure S77: ^{1}H-NMR spectrum of compound **53**.

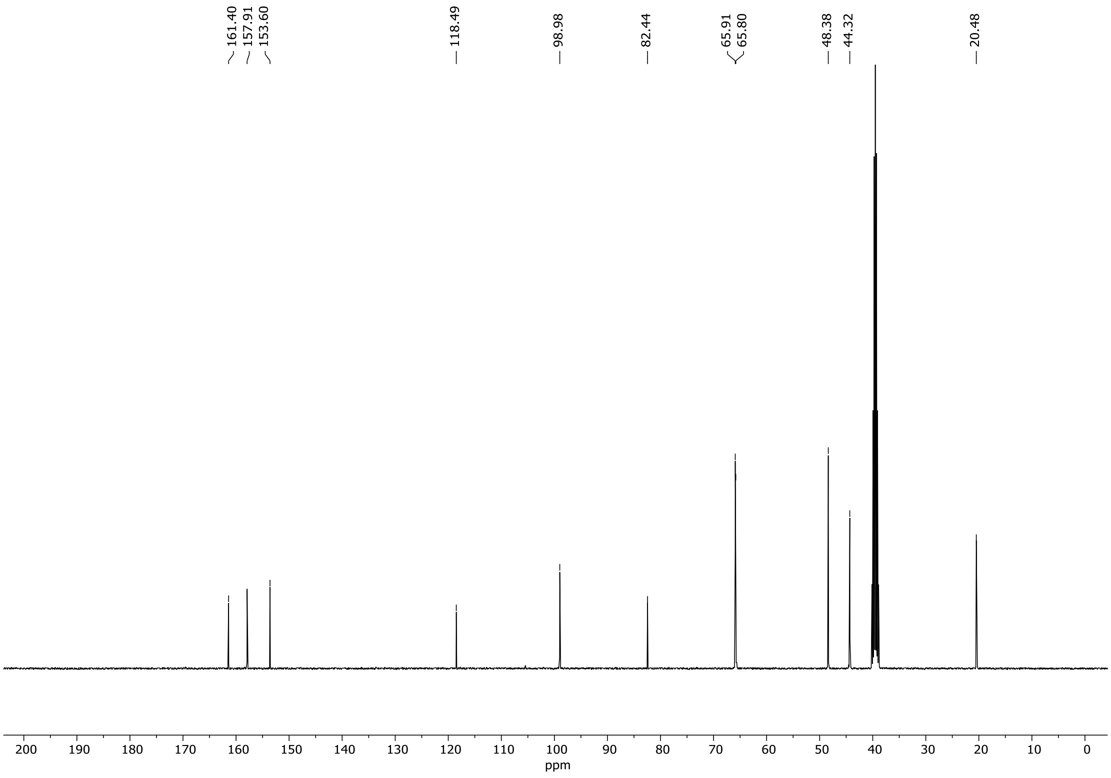

Figure S78: ^{13}C-NMR spectrum of compound **53**.

(4-Methyl-2,6-dimorpholinopyridin-3-yl)methanamine (**54**)

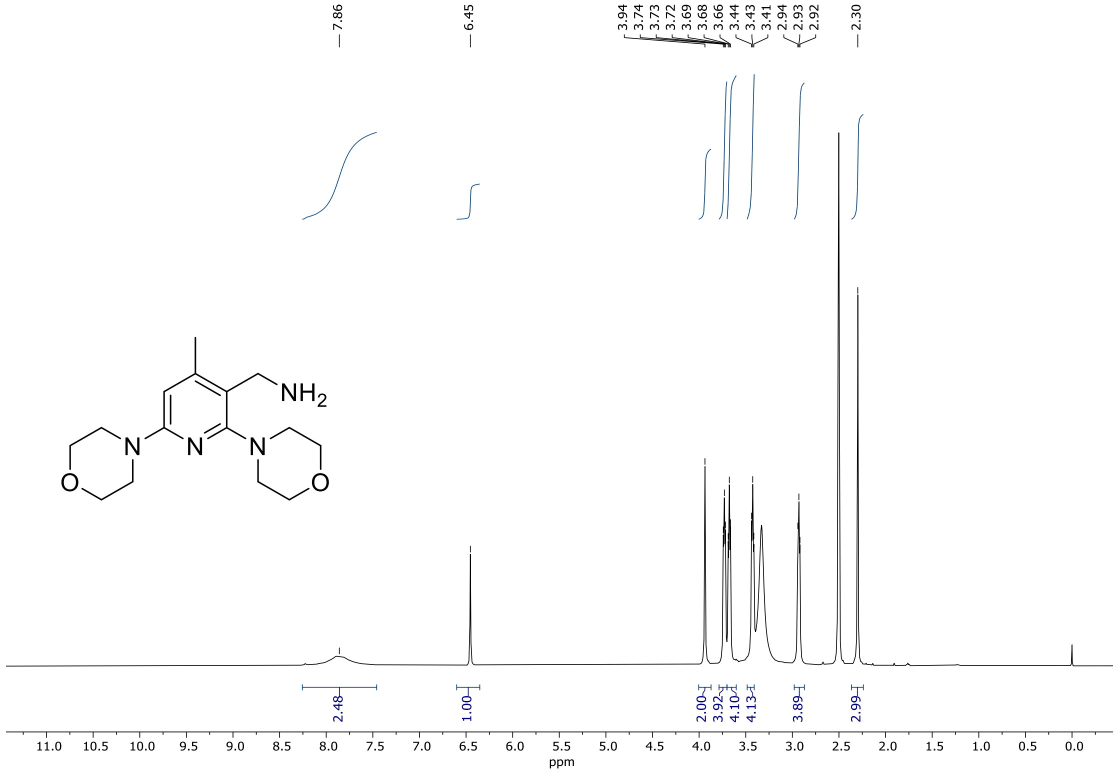

Figure S79: ^{1}H-NMR spectrum of compound **54**.

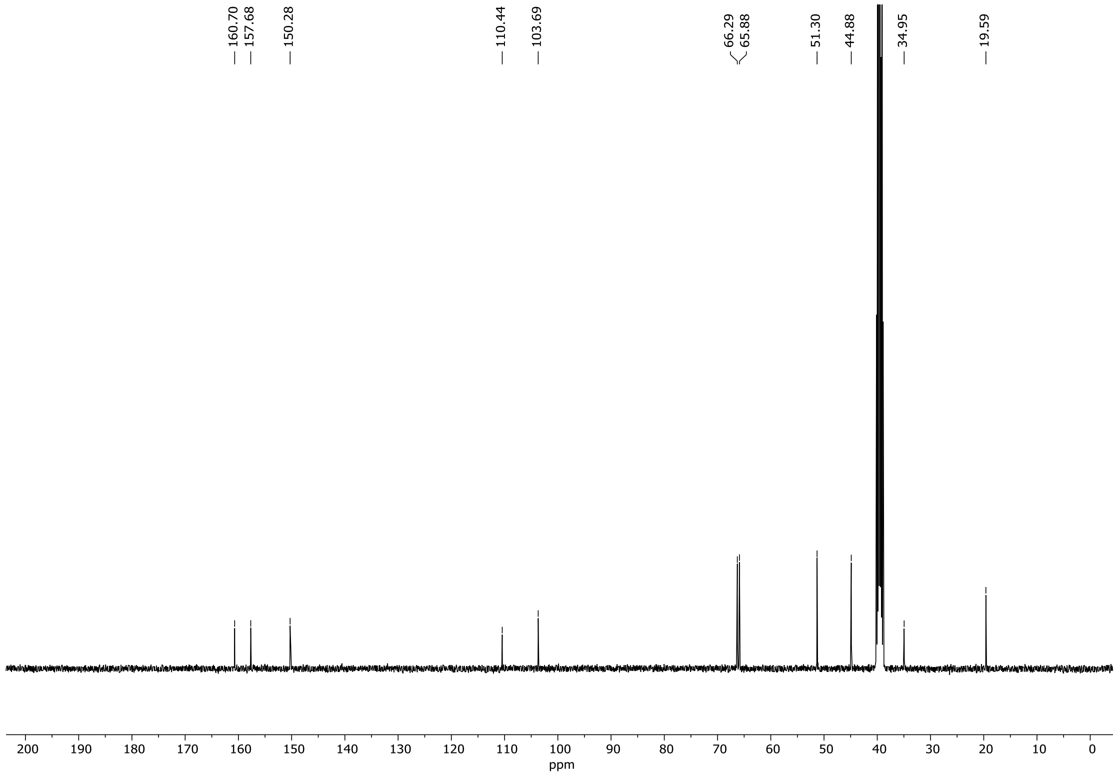

Figure S80: ^{13}C-NMR spectrum of compound **54**.

4-Fluoro-*N*-[(4-methyl-2,6-dimorpholinopyridin-3-yl)methyl]benzamide (**55a**)

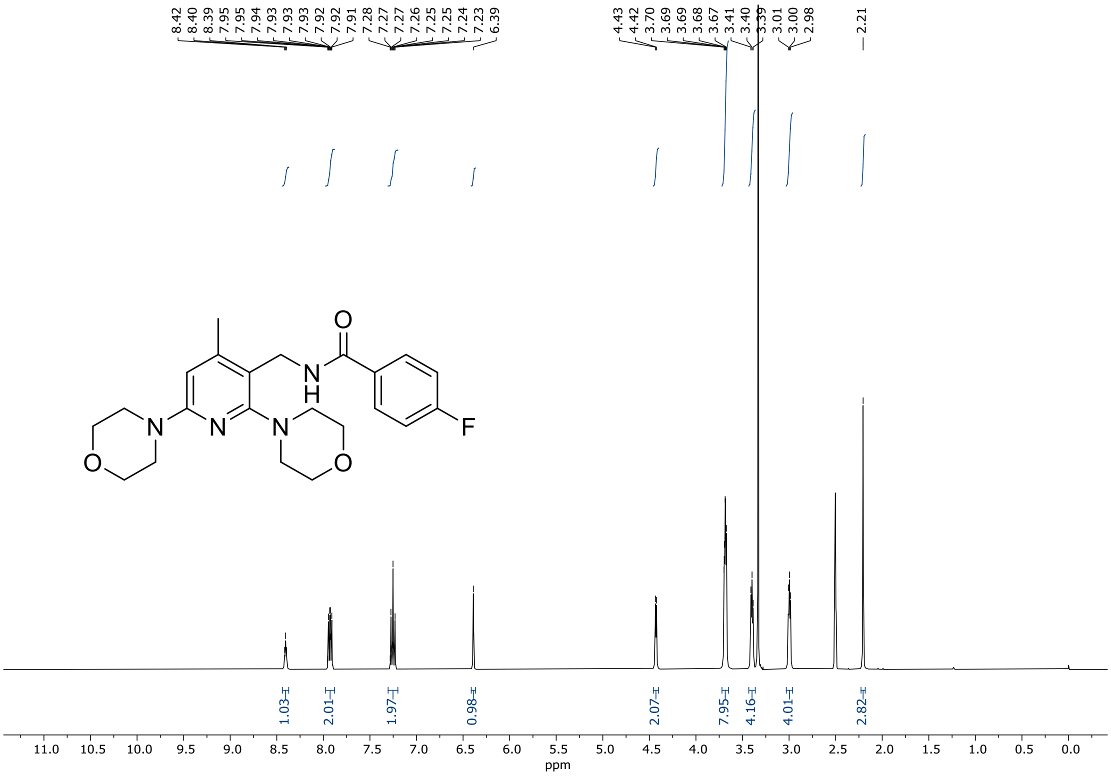

Figure S81: ^{1}H-NMR spectrum of compound **55a**.

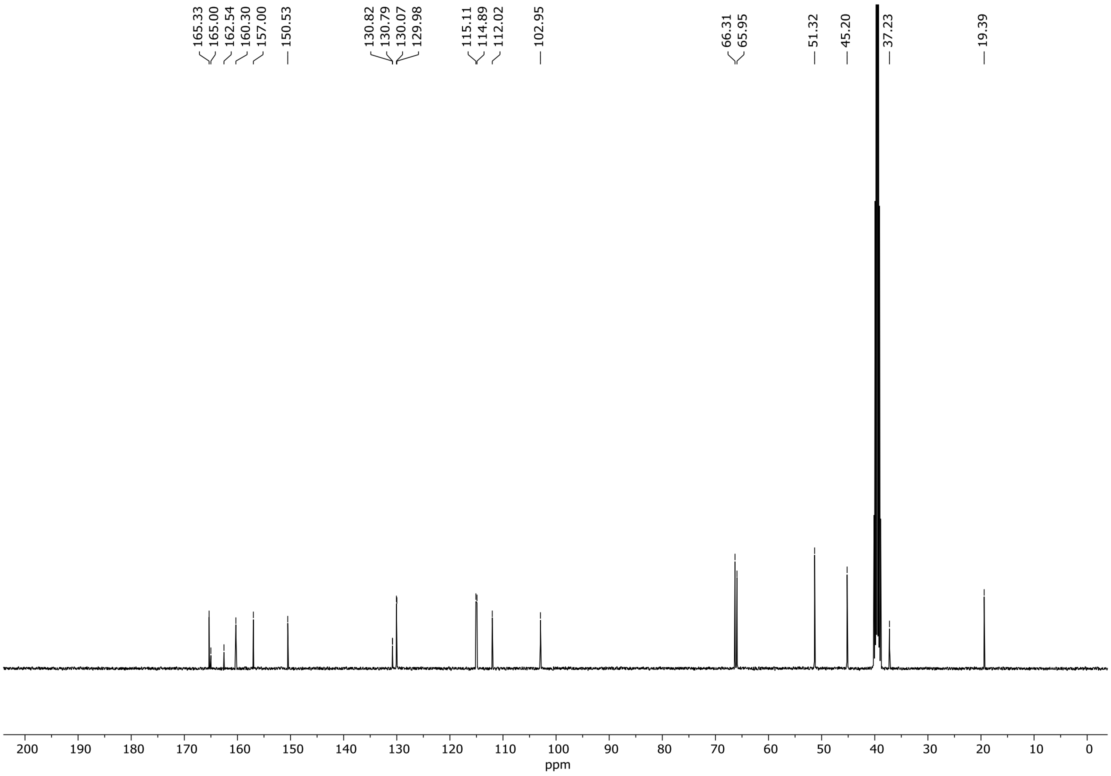

Figure S82: ^{13}C-NMR spectrum of compound **55a**.

2-(3,5-Difluorophenyl)-*N*-[(4-methyl-2,6-dimorpholinopyridin-3-yl)methyl]acetamide (**55b**)

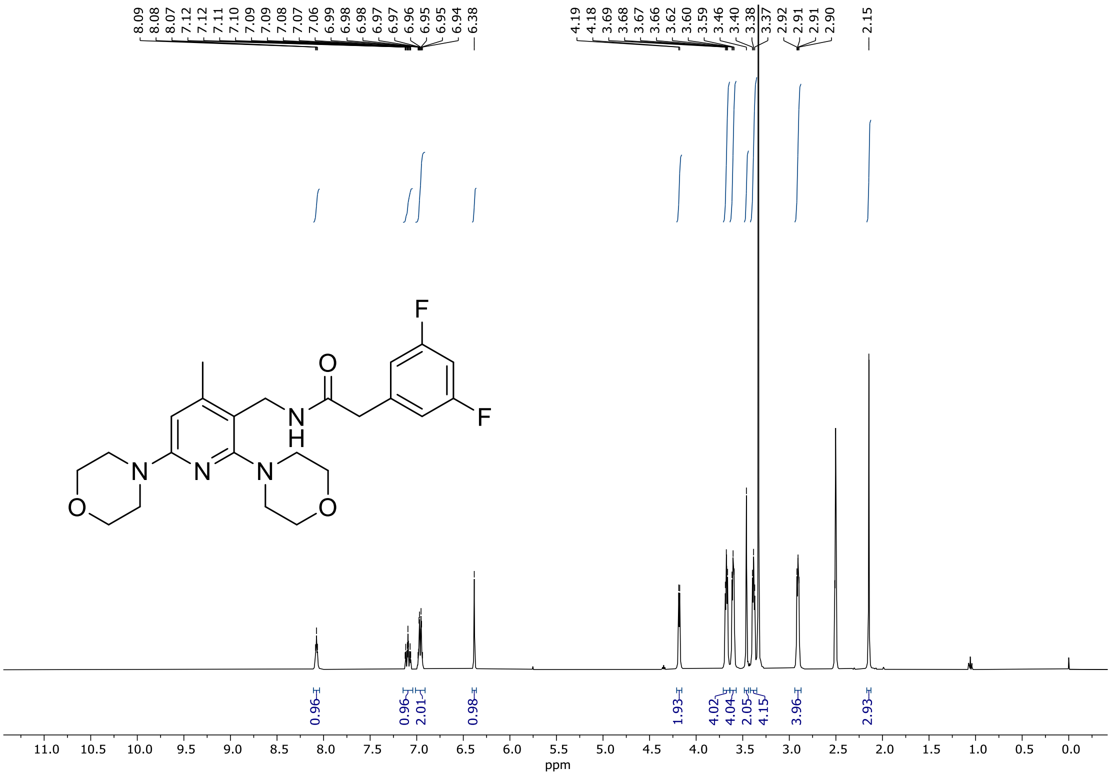

Figure S83: ^{1}H-NMR spectrum of compound **55b**.

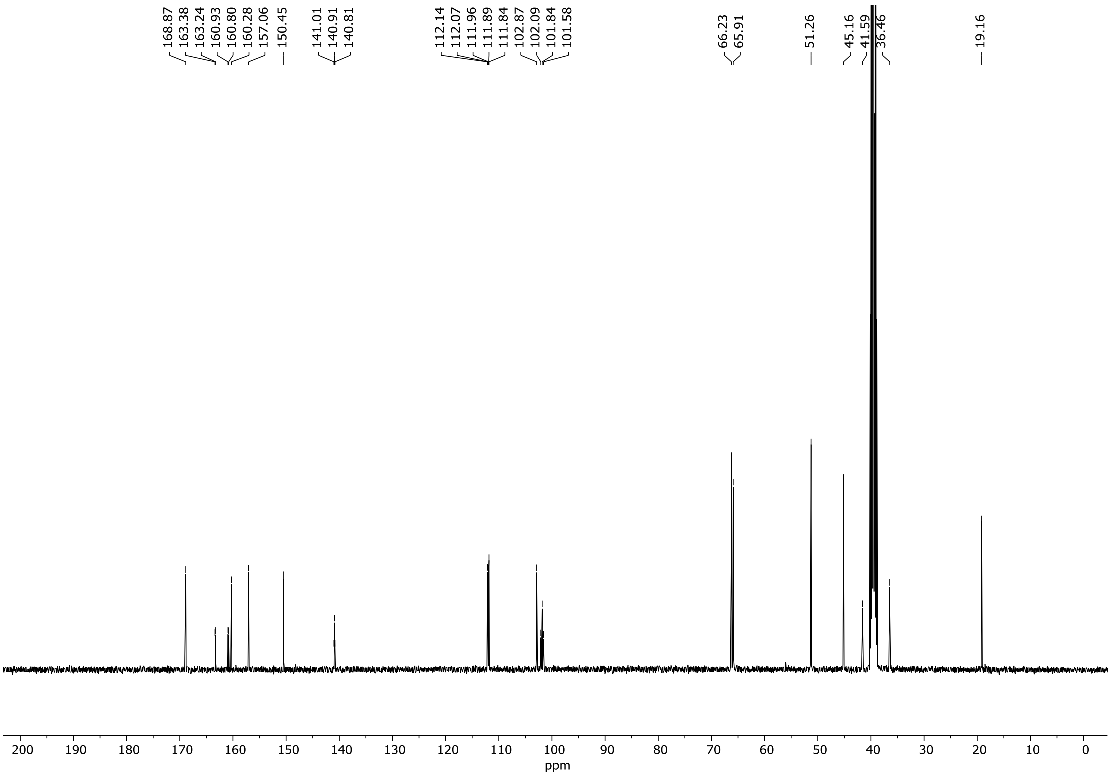

Figure S84: ^{13}C-NMR spectrum of compound **55b**.

4,4'-(4-Methylpyridine-2,6-diyl)dimorpholine (**56**)

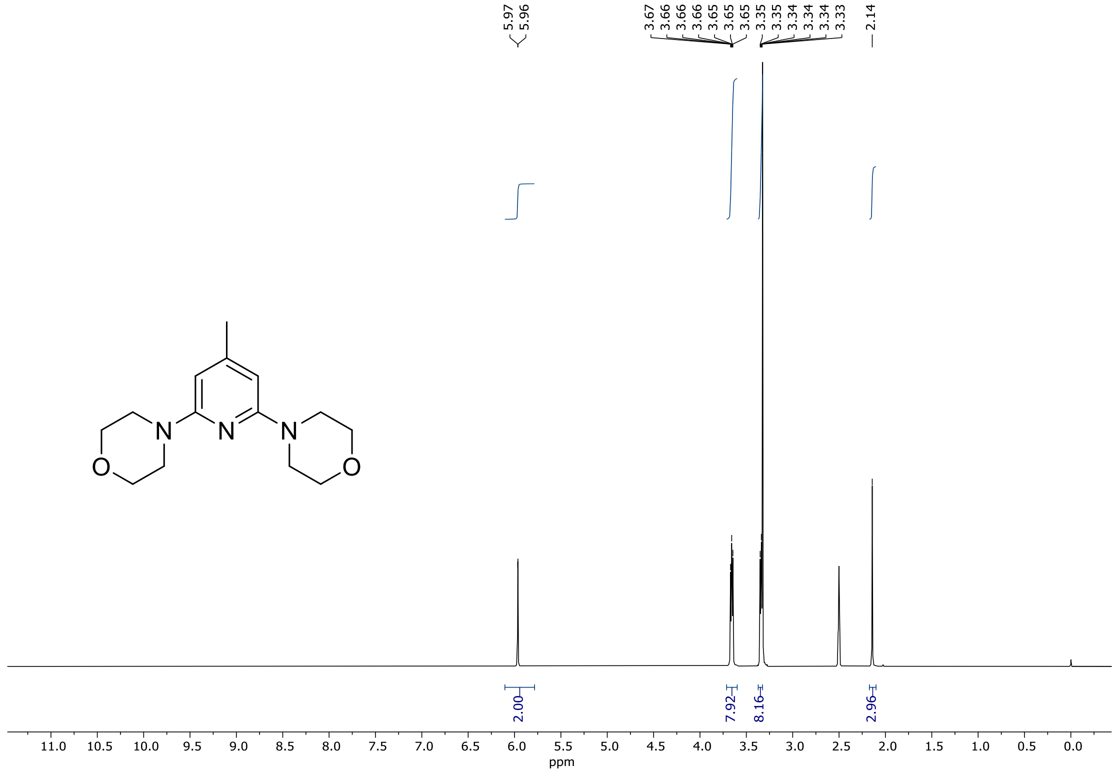

Figure S85: ^{1}H-NMR spectrum of compound **56**.

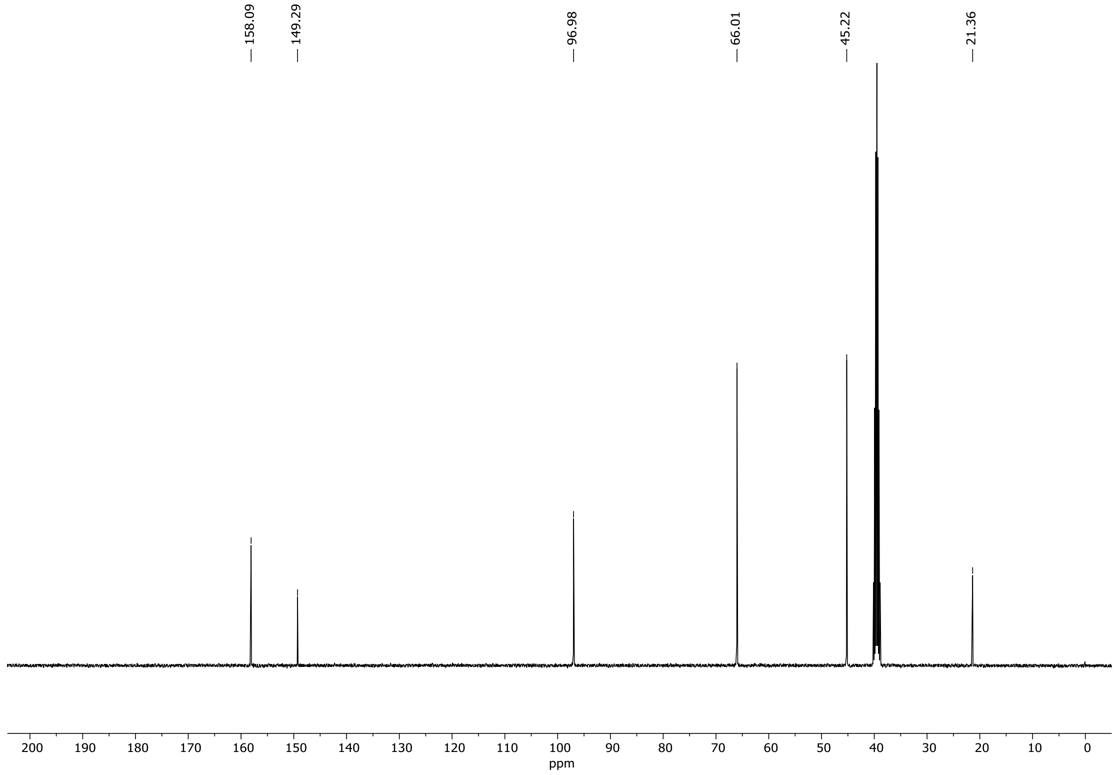

Figure S86: ^{13}C-NMR spectrum of compound **56**.

6-Amino-2-chloro-4-methylnicotinonitrile (**57**)

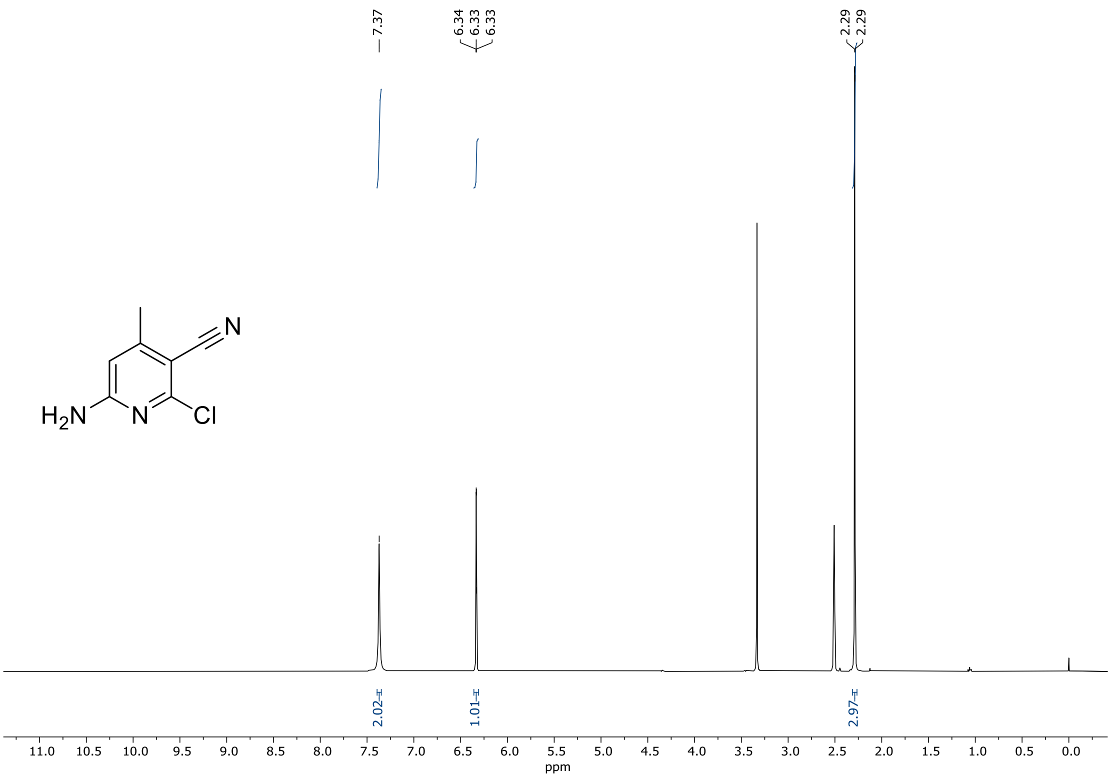

Figure S87: ^{1}H-NMR spectrum of compound **57**.

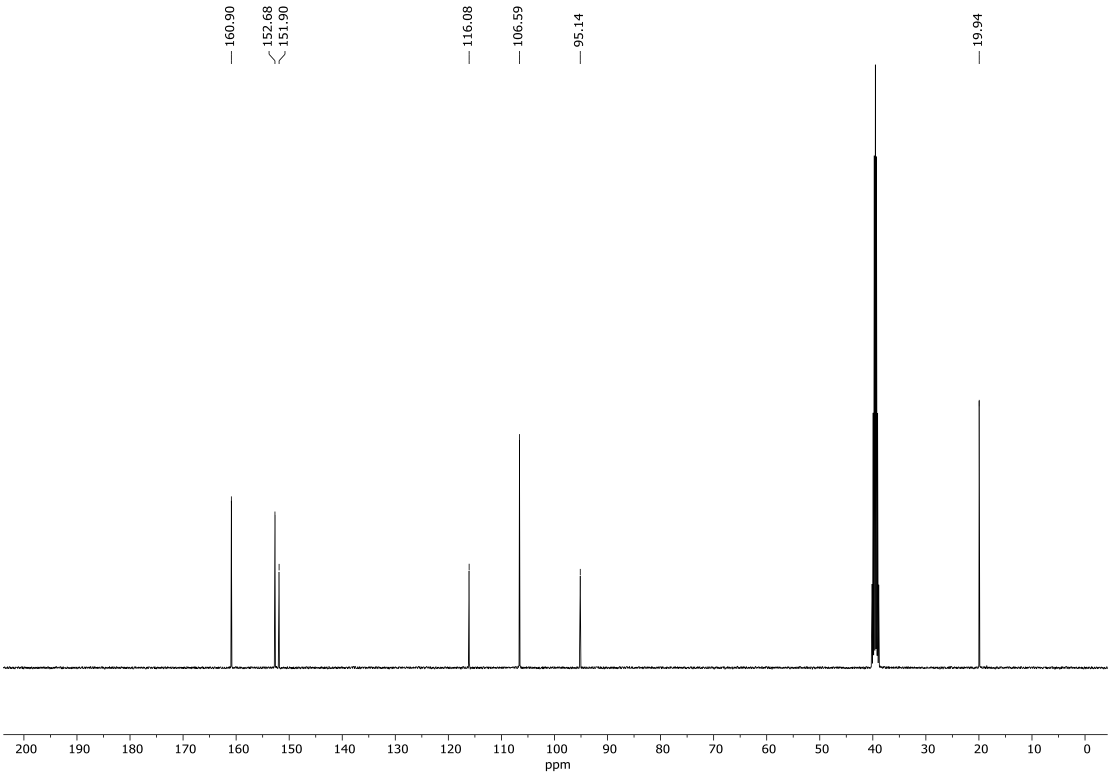

Figure S88: ^{13}C-NMR spectrum of compound **57**.

2-Amino-6-chloro-4-methylnicotinonitrile (**58**)

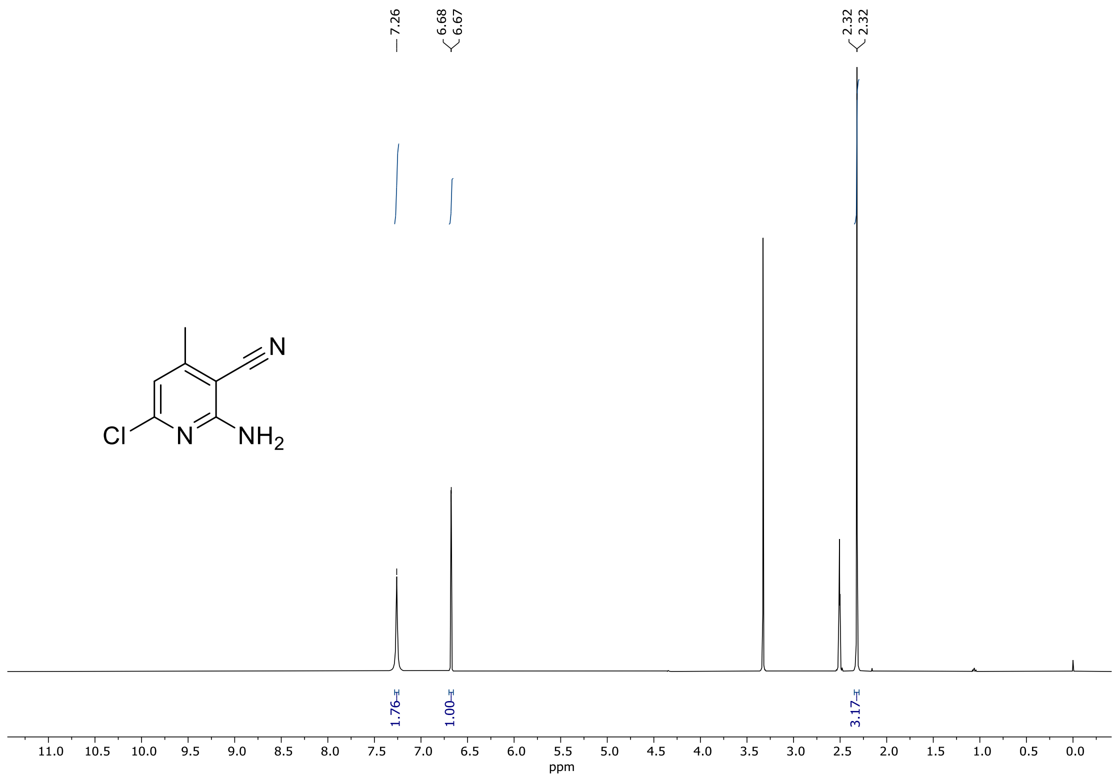

Figure S89: ^{1}H-NMR spectrum of compound **58**.

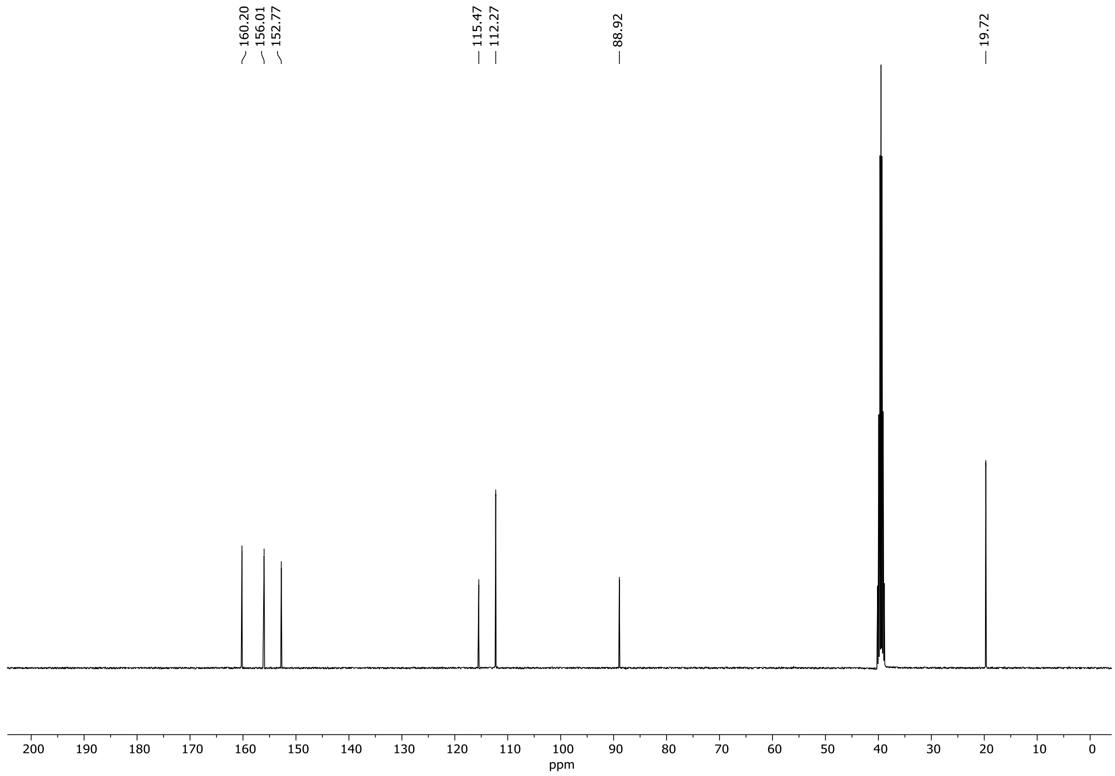

Figure S90: ^{13}C-NMR spectrum of compound **58**.

2-Amino-4-methyl-6-morpholinonicotinonitrile (**59**)

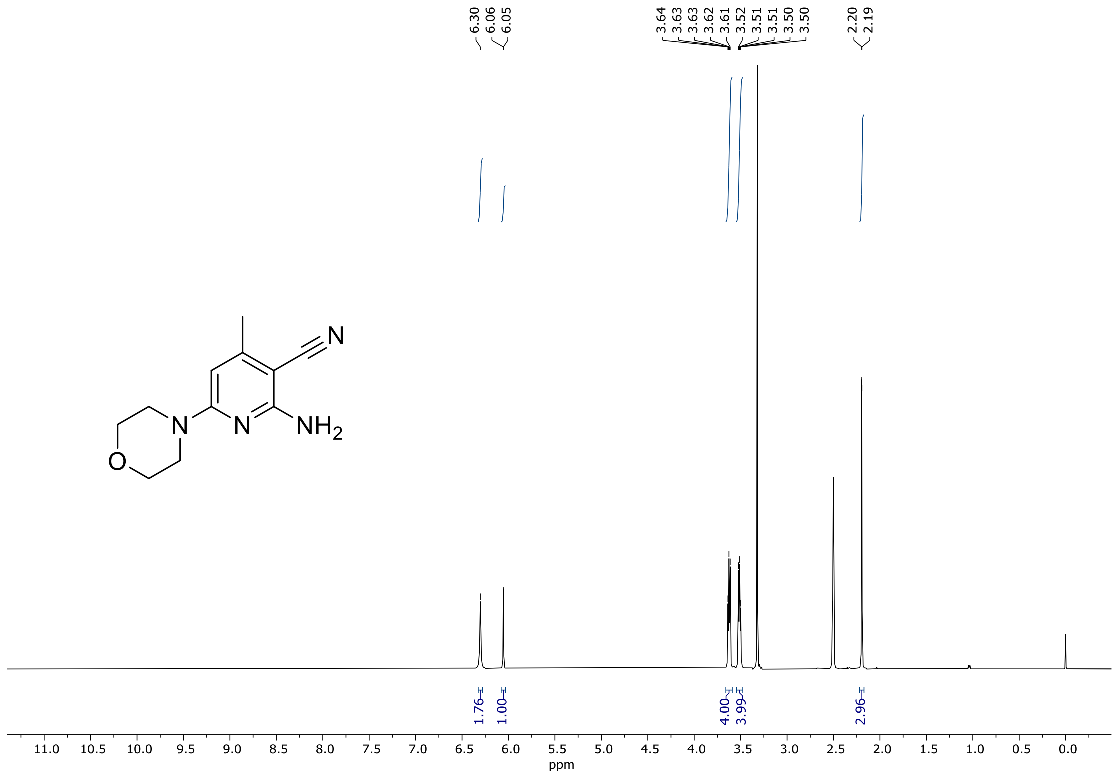

Figure S91: ^{1}H-NMR spectrum of compound **59**.

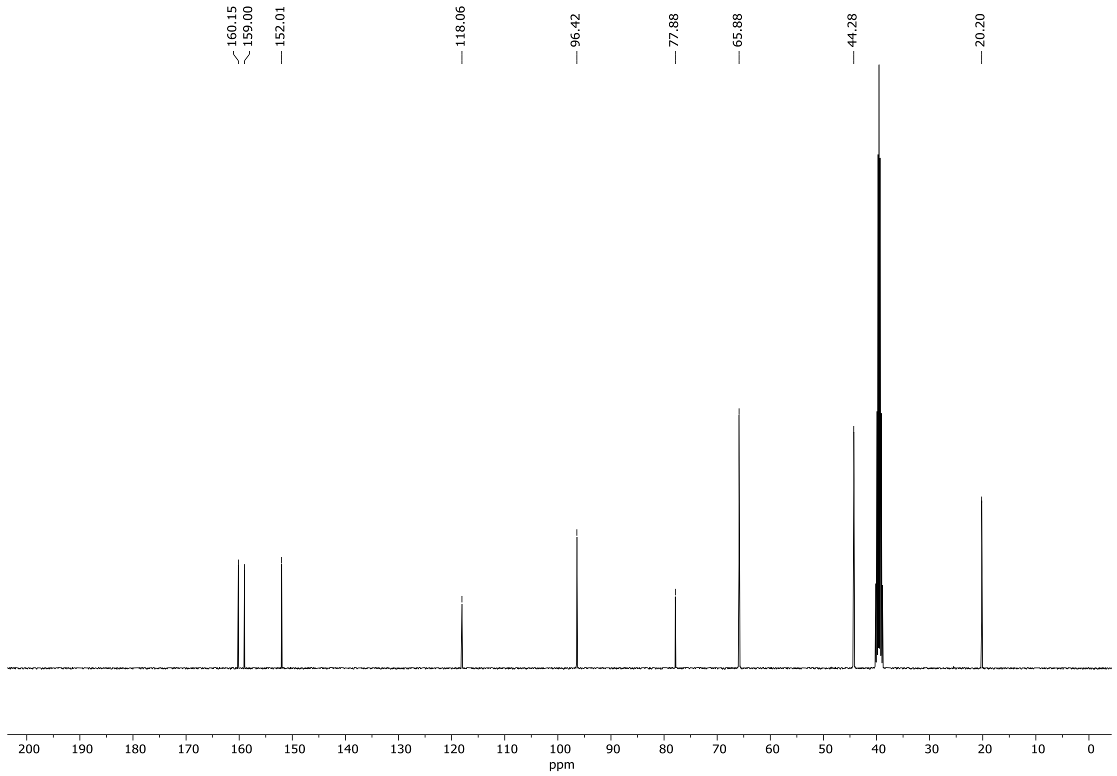

Figure S92: ^{13}C-NMR spectrum of compound **59**.

N-[(2-amino-4-methyl-6-morpholinopyridin-3-yl)methyl]-2-(3,5-difluorophenyl)acetamide (**61**)

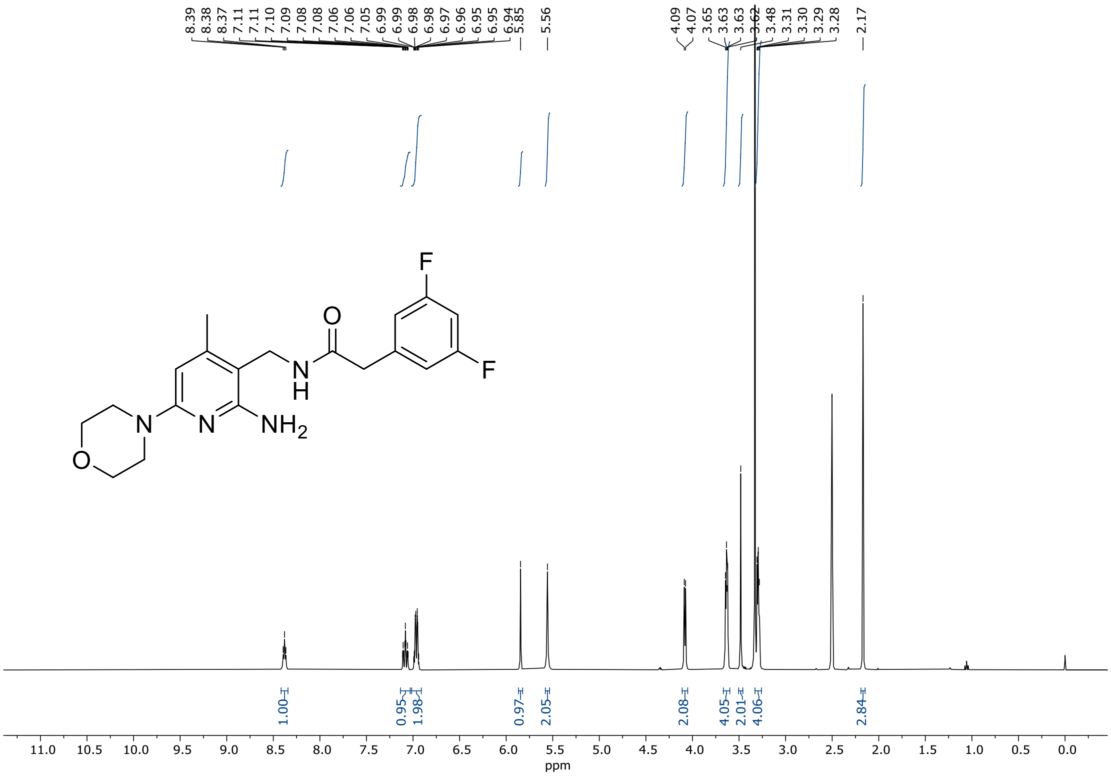

Figure S93: ^{1}H-NMR spectrum of compound **61**.

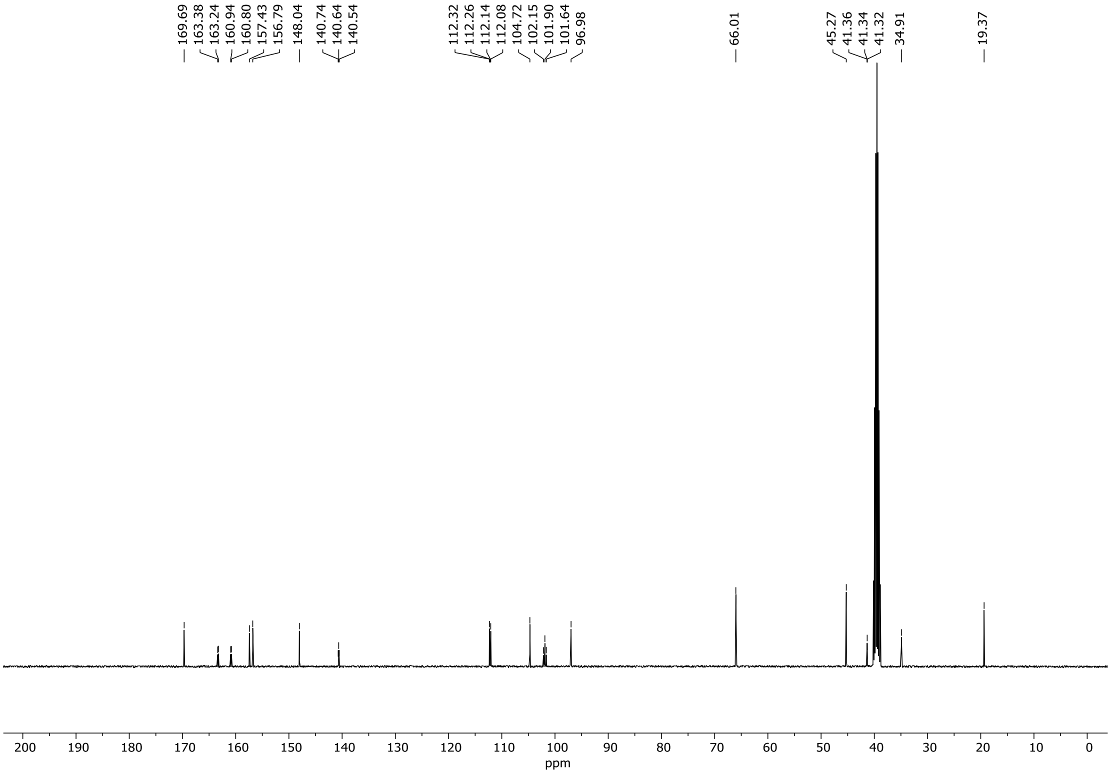

Figure S94: ^{13}C-NMR spectrum of compound **61**.

3,4-Dimethyl-6-morpholinopyridin-2-amine (**62**)

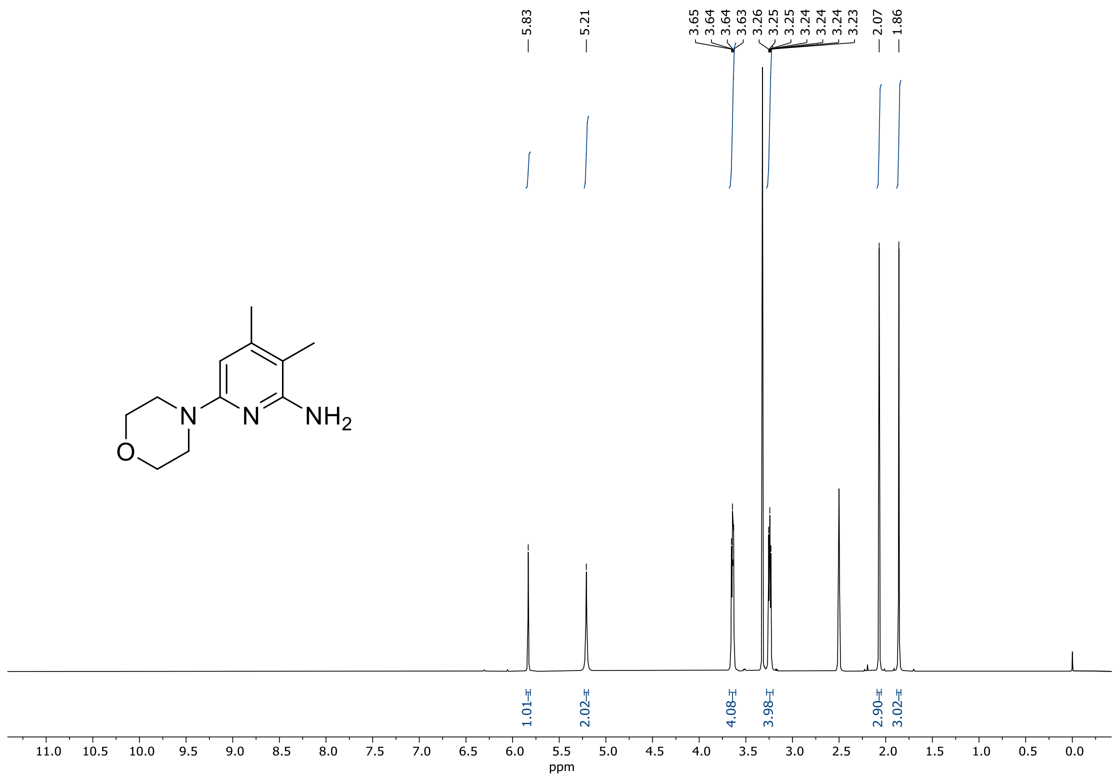

Figure S95: ^{1}H-NMR spectrum of compound **62**.

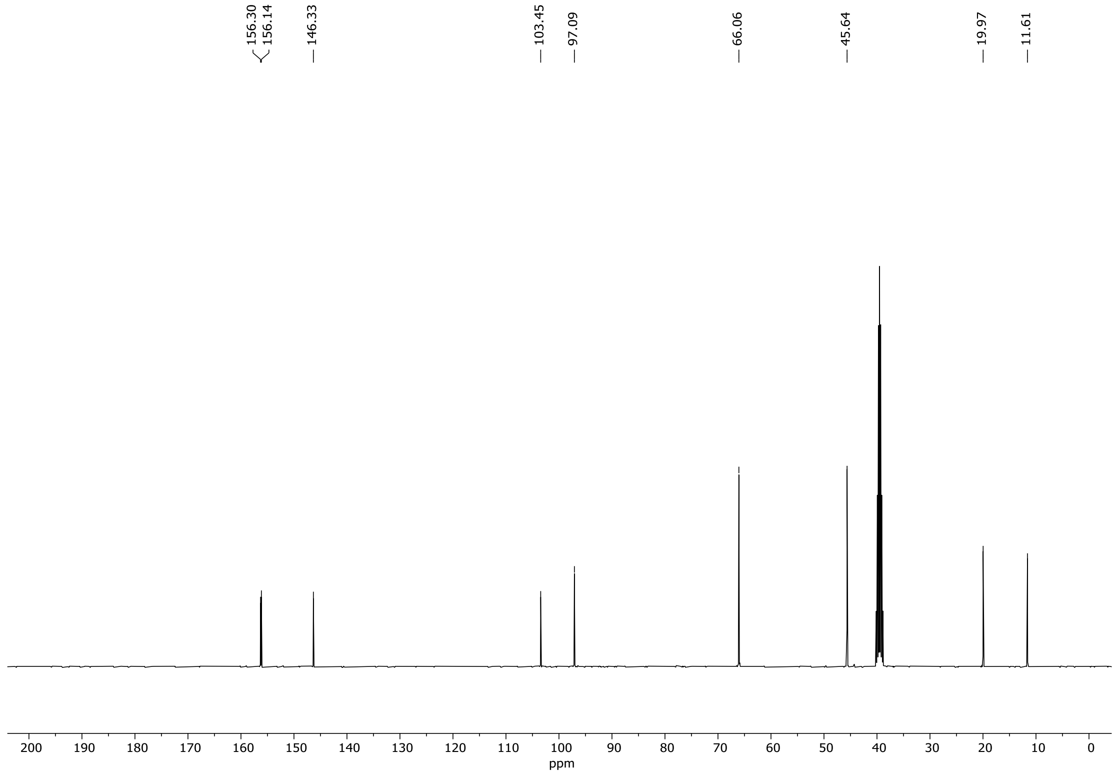

Figure S96: ^{13}C-NMR spectrum of compound **62**.

6-Amino-4-methyl-2-morpholinonicotinonitrile (**63**)

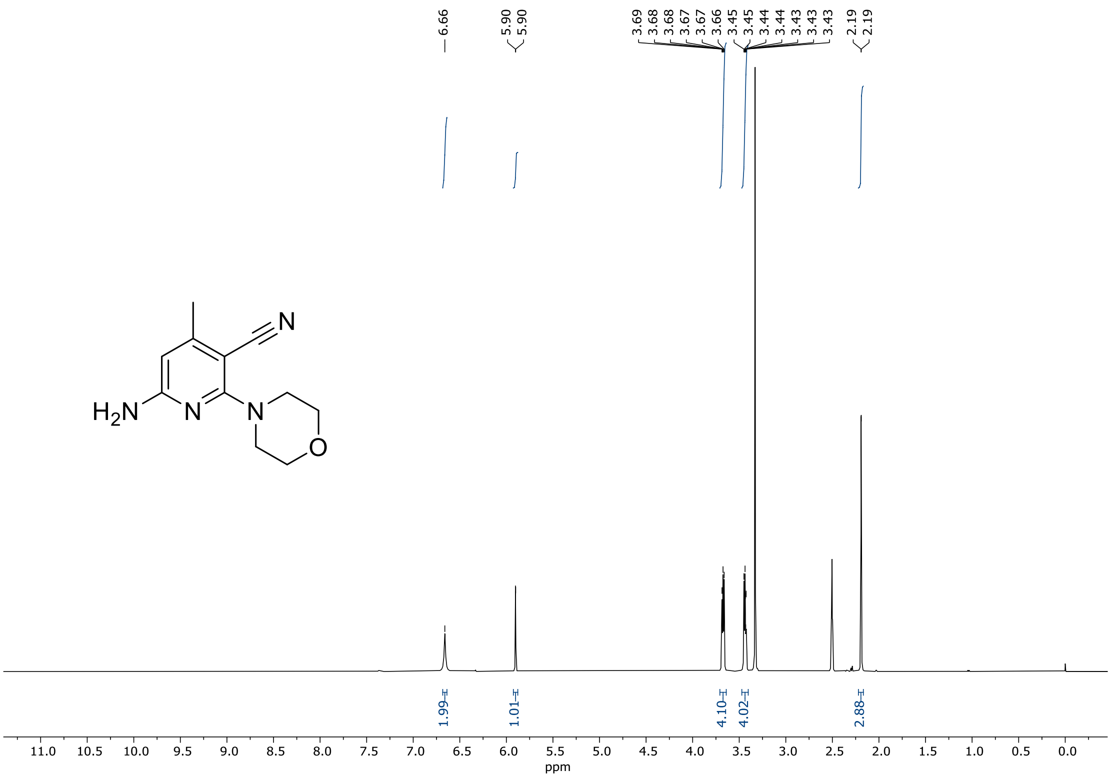

Figure S97: ^{1}H-NMR spectrum of compound **63**.

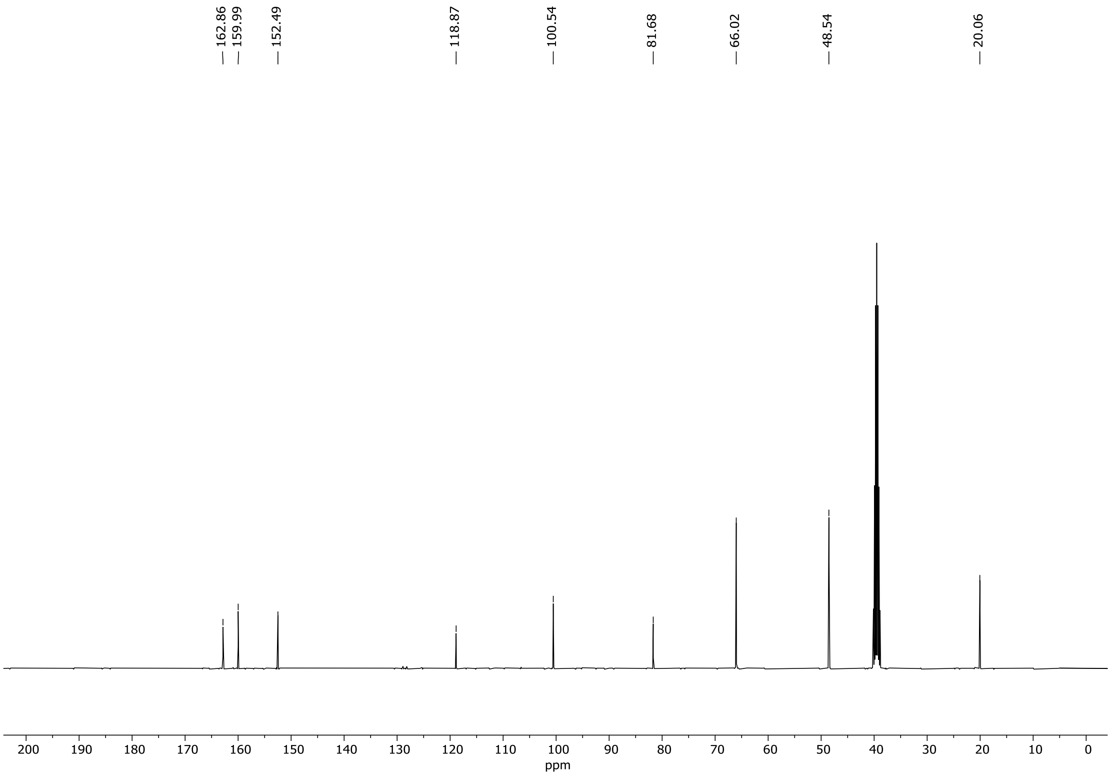

Figure S98: ^{13}C-NMR spectrum of compound **63**.

6-[(4-Fluorobenzyl)amino]-4-methyl-2-morpholinonicotinonitrile (**64**)

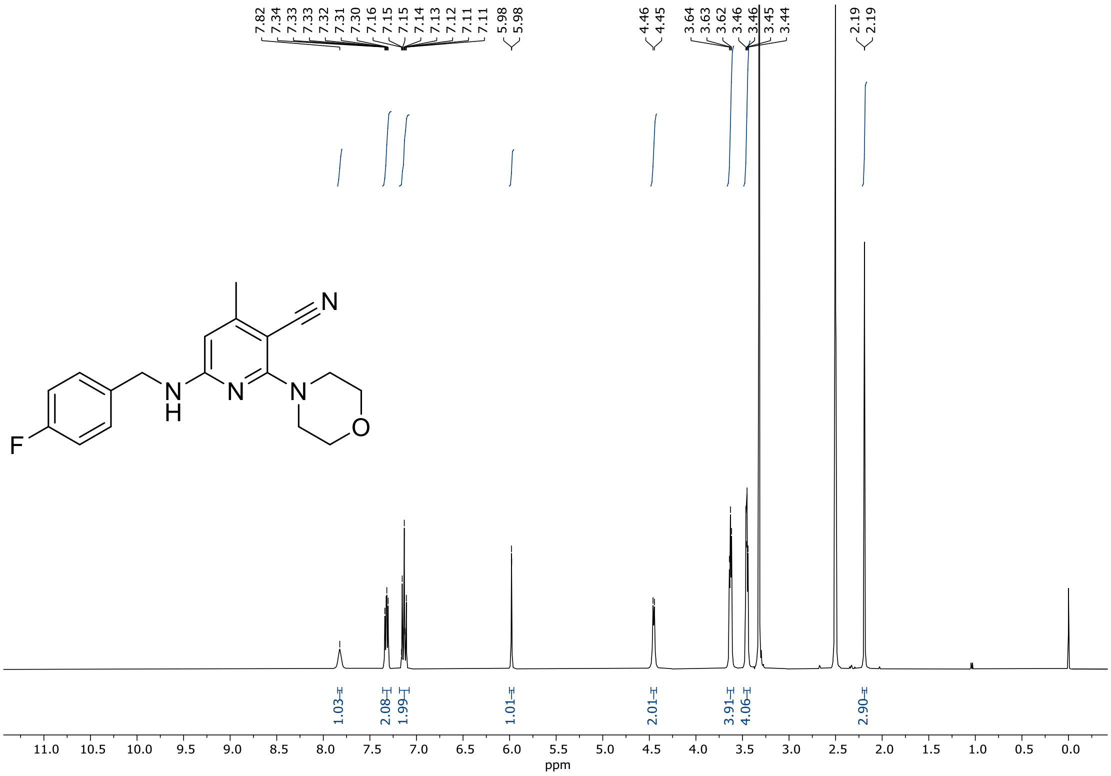

Figure S99: ^{1}H-NMR spectrum of compound **64**.

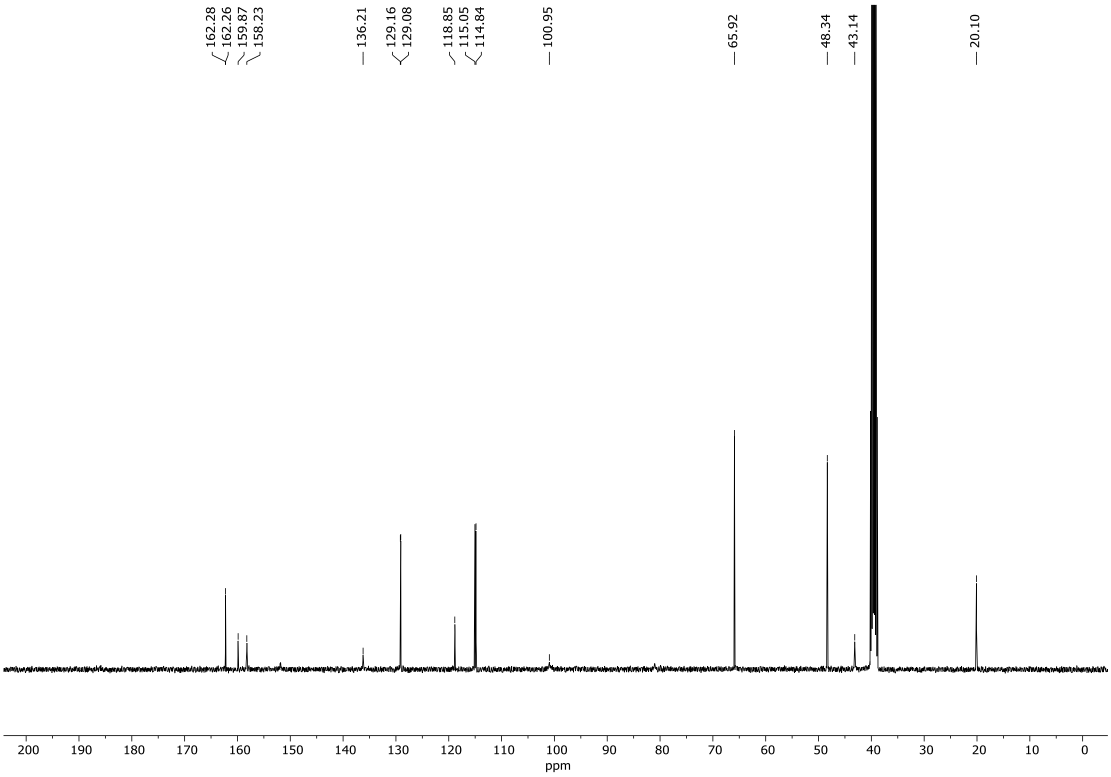

Figure S100: ^{13}C-NMR spectrum of compound **64**.

Isobutyl ({6-[(4-fluorobenzyl)amino]-4-methyl-2-morpholinopyridin-3-yl}methyl)carbamate (**66**)

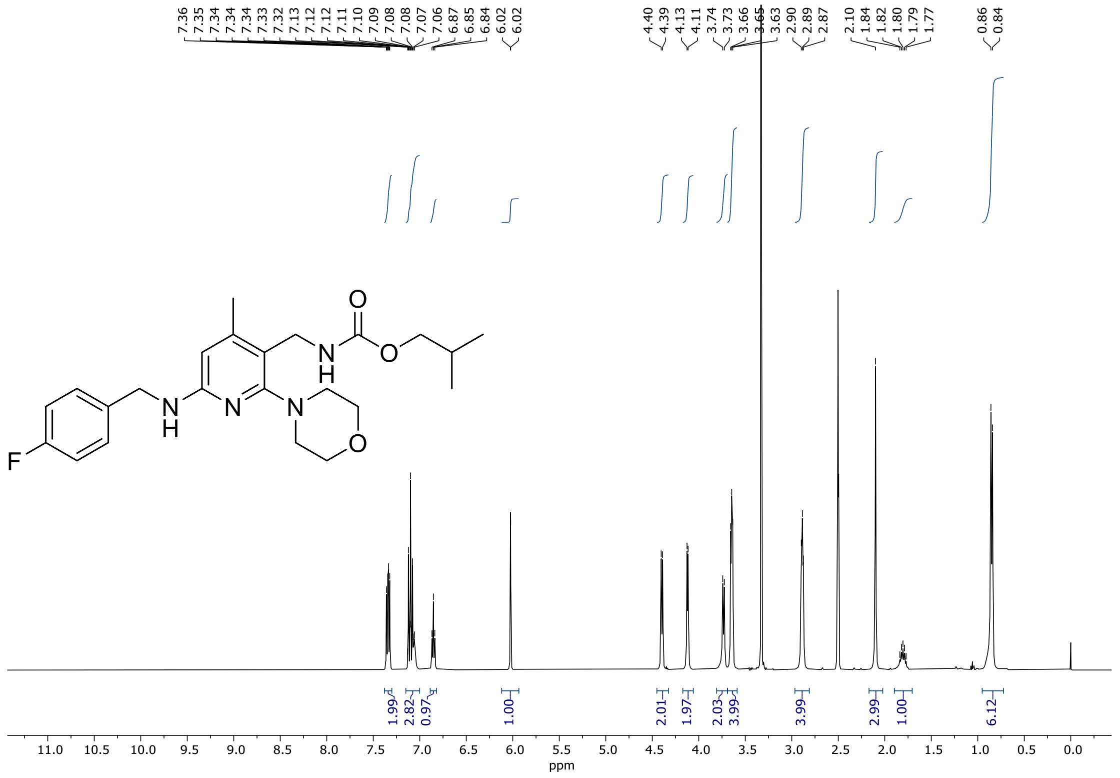

Figure S101: ^{1}H-NMR spectrum of compound **66**.

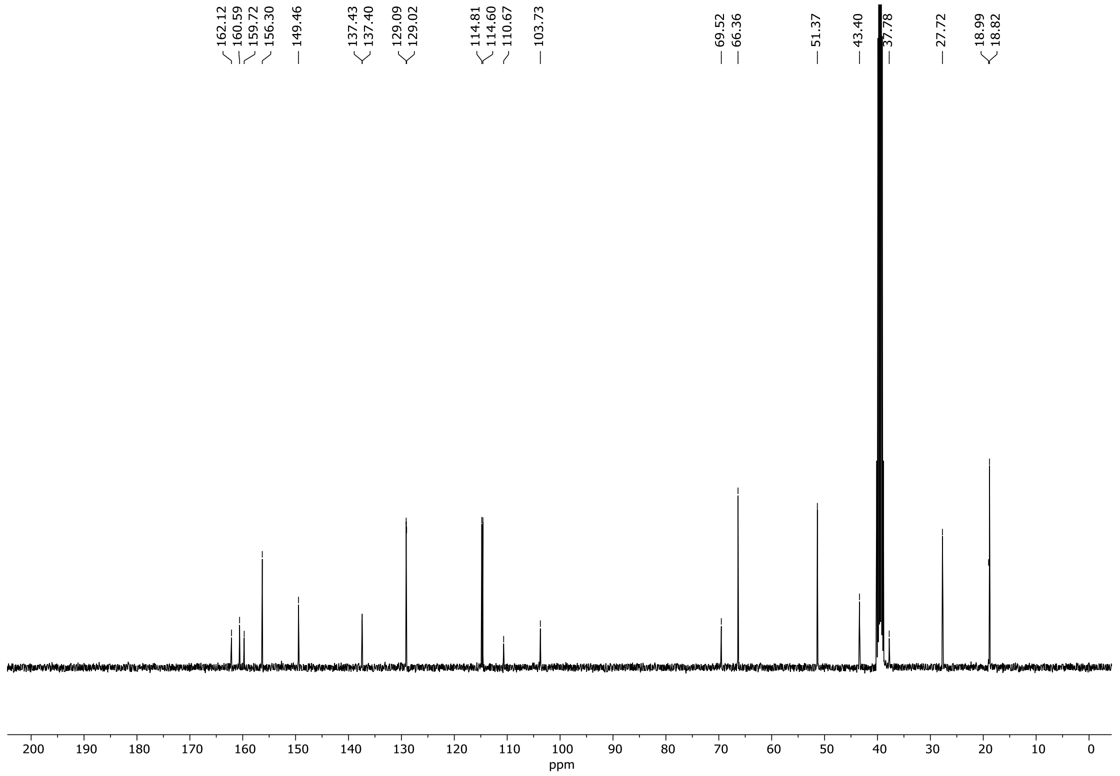

Figure S102: ^{13}C-NMR spectrum of compound **66**.

2 Assignment of NMR signals

2-(4-Methyl-3-nitrophenyl)isoindoline-1,3-dione (**10**)

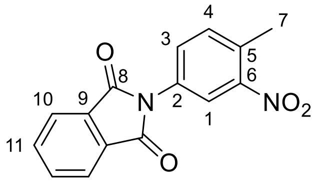

^{1}H-NMR (400 MHz, DMSO-d_6): δ(ppm) = 2.59 (s, 3H, 7-H), 7.68 (dd, J = 8.1, 1.0 Hz, 1H, 4-H), 7.77 (dd, J = 8.2, 2.1 Hz, 1H, 3-H), 7.93 (m, 2H, 10-H), 8.00 (m, 2H, 11-H), 8.18 (d, J = 2.1 Hz, 1H, 1-H); **^{13}C-NMR, DEPT135, HSQC, HMBC (100 MHz, DMSO-d_6):** δ(ppm) = 19.4 (7), 123.0 (1), 123.6 (11), 130.7 (5), 131.5 (3), 132.0 (4), 132.7 (2), 133.2 (9), 134.9 (10), 148.6 (6), 166.6 (8).

N-(4-Methyl-3-nitrophenyl)acetamide (**12**)

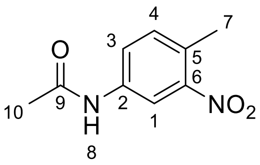

^{1}H-NMR (400 MHz, DMSO-d_6): δ(ppm) = 2.07 (s, 3H, 10-H), 2.46 (s, 3H, 7-H), 7.42 (d, J = 8.4 Hz, 1H, 4-H), 7.70 (dd, J = 8.3, 2.3 Hz, 1H, 3-H), 8.36 (d, J = 2.3 Hz, 1H, 1-H), 10.30 (s, 1H, 8-H); **^{13}C-NMR, DEPT135, HSQC, HMBC (100 MHz, DMSO-d_6):** δ(ppm) = 19.1 (7), 24.0 (10), 114.1 (1), 123.5 (3), 126.9 (5), 133.0 (4), 138.2 (2), 148.6 (6), 168.9 (9).

4-Acetamido-2-nitrobenzoic acid (**13**)

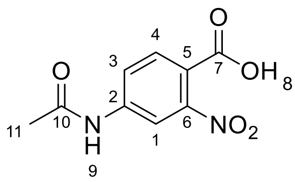

^{1}H-NMR (400 MHz, DMSO-d_6): δ(ppm) = 2.11 (s, 3H, 11-H), 7.78 (dd, J = 8.5, 2.1 Hz, 1H, 3-H), 7.86 (d, J = 8.5 Hz, 1H, 4-H), 8.19 (d, J = 2.0 Hz, 1H, 1-H), 10.61 (s, 1H, 9-H), 13.57 (s, 1H, 8-H); **^{13}C-NMR, DEPT135, HSQC, HMBC (100 MHz, DMSO-d_6):** δ(ppm) = 24.1 (11), 112.8 (1), 119.9 (5), 121.3 (3), 131.4 (4), 142.8 (2), 149.8 (6), 165.1 (8), 169.5 (10).

4-Acetamido-*N*-isobutyl-2-nitrobenzamide (**14**)

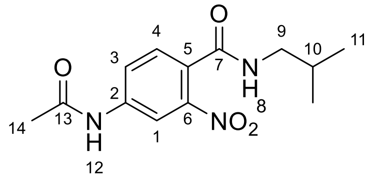

^{1}H-NMR (400 MHz, DMSO-d_6): δ(ppm) = 0.90 (d, *J* = 6.7 Hz, 6H, 11-H), 1.80 (m, 1H, 10-H), 2.10 (s, 3H, 14-H), 3.02 (dd, *J* = 6.8, 5.9 Hz, 2H, 9-H), 7.53 (d, *J* = 8.3 Hz, 1H, 4-H), 7.80 (dd, *J* = 8.4, 2.1 Hz, 1H, 3-H), 8.31 (d, *J* = 2.1 Hz, 1H, 1-H), 8.57 (t, *J* = 5.9 Hz, 1H, 8-H), 10.49 (s, 1H, 12-H); **^{13}C-NMR, DEPT135, HSQC, HMBC (100 MHz, DMSO-d_6):** δ(ppm) = 20.2 (11), 24.1 (13), 28.0 (10), 46.7 (9), 113.5 (1), 122.4 (3), 126.9 (5), 129.7 (4), 140.8 (6), 147.6 (2), 165.2 (7), 169.3 (13).

4-Amino-*N*-isobutyl-2-nitrobenzamide (**15**)

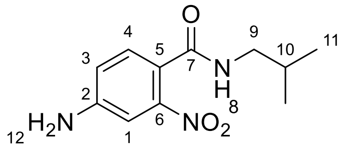

^{1}H-NMR (400 MHz, DMSO-d_6): δ(ppm) = 0.88 (d, *J* = 6.7 Hz, 6H, 11-H), 1.77 (m, 1H, 10-H), 2.97 (dd, *J* = 6.9, 5.9 Hz, 2H, 9-H), 6.04 (s, 2H, 12-H), 6.76 (dd, *J* = 8.4, 2.3 Hz, 1H, 3-H), 6.97 (d, *J* = 2.3 Hz, 1H, 1-H), 7.27 (d, *J* = 8.4 Hz, 1H, 4-H), 8.32 (t, *J* = 5.8 Hz, 1H, 8-H); **^{13}C-NMR, DEPT135, HSQC, HMBC (100 MHz, DMSO-d_6):** δ(ppm) = 20.2 (11), 28.1 (10), 46.5 (9), 107.5 (1), 115.9 (3), 118.4 (5), 129.9 (4), 149.6 (2), 150.9 (6), 165.4 (7).

4-[(4-Fluorobenzyl)amino]-*N*-isobutyl-2-nitrobenzamide (**16**)

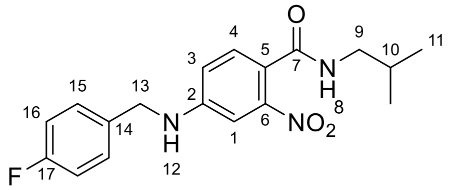

^{1}H-NMR (400 MHz, DMSO-d_6): δ(ppm) = 0.86 (d, *J* = 6.7 Hz, 6H, 11-H), 1.76 (m, 1H, 10-H), 2.96 (dd, *J* = 6.9, 5.9 Hz, 2H, 9-H), 4.35 (d, *J* = 6.0 Hz, 2H, 13-H), 6.79 (dd, *J* = 8.5, 2.4 Hz, 1H, 3-H), 7.0 (d, *J* = 2.3 Hz, 1H, 1-H), 7.16 (m, 2H, 16-H), 7.21 (t, *J* = 6.1 Hz, 1H, 12-H), 7.30 (d, *J* = 8.5 Hz, 1H, 4-H), 7.38 (m, 2H, 15-H), 8.33 (t, *J* = 5.8 Hz, 1H, 8-H); **^{13}C-NMR, DEPT135, HSQC, HMBC (100 MHz, DMSO-d_6):** δ(ppm) = 20.1 (11), 28.1 (10), 45.1 (13), 46.5 (9), 106.4 (1), 114.3 (3), 115.2 (d, *J* = 21.3 Hz, 16), 118.5 (5), 129.1 (d, *J* = 8.1 Hz, 15), 129.8 (4), 135.0 (d, *J* = 3.0 Hz, 14), 149.7 (6), 150.0 (2), 161.2 (d, *J* = 242.4 Hz, 17), 165.2 (7).

2-Amino-4-[(4-fluorobenzyl)amino]-*N*-isobutylbenzamide (**17**)

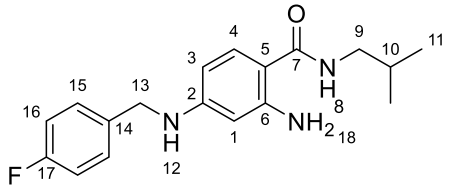

^{1}H-NMR (400 MHz, DMSO-d_6): δ(ppm) = 0.84 (d, *J* = 6.7 Hz, 6H, 11-H), 1.77 (m, 1H, 10-H), 2.95 (dd, *J* = 7.0, 5.8 Hz, 2H, 9-H), 4.21 (d, *J* = 6.1 Hz, 2H, 13-H), 5.76 (d, *J* = 2.3 Hz, 1H, 1-H), 5.83 (dd, 8.7, 2.3 Hz, 1H, 3-H), 6.34 (s, 2H, 18-H), 6.15 (t, *J* = 6.2 Hz, 1H, 12-H), 7.13 (m, 2H, 16-H), 7.26 (d, *J* = 8.8 Hz, 1H, 4-H), 7.34 (m, 2H, 15-H), 7.70 (t, *J* = 5.8 Hz, 1H, 8-H); **^{13}C-NMR, DEPT135, HSQC, HMBC (100 MHz, DMSO-d_6):** δ(ppm) = 20.2 (11), 28.1 (10), 45.2 (13), 46.1 (9), 97.0 (1), 101.6 (3), 104.2 (5), 114.9 (d, *J* = 21.1 Hz, 16), 128.8 (d, *J* = 8.0 Hz, 15), 129.1 (4), 136.3 (d, *J* = 2.9 Hz, 14), 151.2 (6), 151.5 (2), 161.0 (d, *J* = 241.8 Hz, 17), 168.9 (7).

2-Amino-6-chloronicotinic acid (**19**)

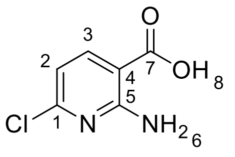

^{1}H-NMR (400 MHz, DMSO-d_6): δ(ppm) = 6.63 (d, *J* = 8.1 Hz, 1H, 2-H), 7.55 (s, 2H, 6-H), 8.03 (d, *J* = 8.1 Hz, 1H, 3-H), 13.14 (s, 1H, 8-H); **^{13}C-NMR, DEPT135, HSQC, HMBC (100 MHz, DMSO-d_6):** δ(ppm) = 104.5 (1), 111.0 (2), 143.2 (3), 153.2 (4), 159.7 (5), 167.9 (7).

2-Amino-*N*-butyl-6-chloronicotinamide (**20**)

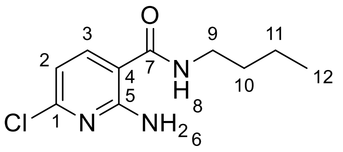

^{1}H-NMR (400 MHz, DMSO-d_6): δ(ppm) = 0.89 (t, *J* = 7.3 Hz, 3H, 12-H), 1.31 (m, 2H, 11-H), 1.47 (m, 2H, 10-H), 3.21 (td, *J* = 7.1, 5.6 Hz, 2H, 9-H), 6.62 (d, *J* = 8.0 Hz, 1H, 2-H), 7.47 (s, 2H, 6-H), 7.91 (d, *J* = 8.1 Hz, 1H, 3-H), 8.42 (t, *J* = 5.6 Hz, 1H, 8-H); **^{13}C-NMR, DEPT135, HSQC, HMBC (100 MHz, DMSO-d_6):** δ(ppm) = 13.7 (12), 19.6 (11), 31.0 (10), 38.7 (9), 108.4 (1), 110.0 (2), 139.4 (3), 150.9 (4), 159.0 (5), 166.5 (7).

2-Amino-*N*-butyl-6-[(4-fluorobenzyl)amino]nicotinamide (**21**)

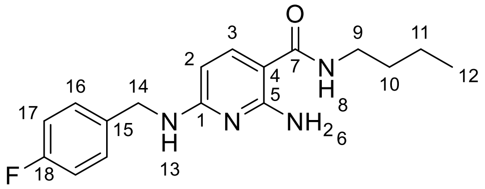

^{1}H-NMR (400 MHz, DMSO-d_6): δ(ppm) = 0.88 (t, *J* = 7.32 Hz, 3H, 12-H), 1.29 (m, 2H, 11-H), 1.44 (tt, *J* = 7.9, 6.4 Hz, 2H, 10-H), 3.15 (td, *J* = 7.1, 5.6 Hz, 2H, 9-H), 4.44 (d, *J* = 6.0 Hz, 2H, 14-H), 5.71 (d, *J* = 8.6 Hz, 1H, 2-H), 7.02 (s, 2H, 6-H), 7.13 (m, 3H, 13-H, 17-H), 7.35 (m, 2H, 16-H), 7.60 (d, *J* = 8.6 Hz, 1H, 3-H), 7.73 (t, *J* = 5.6 Hz, 1H, 8-H); **^{13}C-NMR, DEPT135, HSQC, HMBC (100 MHz, DMSO-d_6):** δ(ppm) = 13.7 (12), 19.7 (11), 31.5 (10), 38.4 (9), 43.0 (14), 96.0 (2), 97.1 (4), 114.8 (d, *J* = 21.1 Hz, 17), 129.2 (d, *J* = 7.9 Hz, 16), 136.7 (d, *J* = 3.0 Hz, 15), 137.1 (3), 159.1 (1), 159.3 (5), 161.0 (d, *J* = 241.8 Hz, 18), 167.7 (7).

2-Bromo-1-methyl-4-nitrobenzene (**23**)

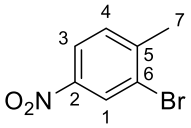

^{1}H-NMR (400 MHz, DMSO-d_6): δ(ppm) = 2.47 (d, *J* = 0.6 Hz, 3H, 7-H), 7.66 (dd, *J* = 8.4, 0.8 Hz, 1H, 4-H), 8.16 (dd, *J* = 8.4, 2.4 Hz, 1H, 3-H), 8.38 (d, *J* = 2.4 Hz, 1H, 1-H); **^{13}C-NMR, DEPT135, HSQC, HMBC (100 MHz, DMSO-d_6):** δ(ppm) = 22.6 (7), 122.5 (3), 124.2 (6), 126.7 (1), 131.8 (4), 145.7 (5), 146.3 (2).

2-Bromo-4-nitrobenzoic acid (**24**)

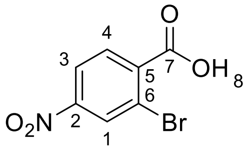

^{1}H-NMR (400 MHz, DMSO-d_6): δ(ppm) = 7.95 (d, *J* = 8.5 Hz, 1H, 4-H), 8.22 (dd, *J* = 8.5, 2.2 Hz, 1H, 3-H), 8.48 (d, *J* = 2.2 Hz, 1H, 1-H), 14.06 (s, 1H, 8-H); **^{13}C-NMR, DEPT135, HSQC, HMBC (100 MHz, DMSO-d_6):** δ(ppm) = 119.9 (6), 122.8 (3), 128.2 (1), 131.1 (4), 140.1 (5), 148.7 (2), 166.5 (7).

2-Bromo-*N*-isobutyl-4-nitrobenzamide (**25a**)

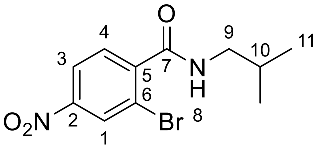

^{1}H-NMR (400 MHz, DMSO-*d*$_6$): δ(ppm) = 0.93 (d, *J* = 6.7 Hz, 6H, 11-H), 1.82 (dq, *J* = 13.4, 6.7 Hz, 1H, 10-H), 3.08 (dd, *J* = 6.8, 5.9 Hz, 2H, 9-H), 7.64 (d, *J* = 8.4 Hz, 1H, 4-H), 8.26 (dd, *J* = 8.4, 2.3 Hz, 1H, 3-H), 8.45 (d, *J* = 2.2 Hz, 1H, 1-H), 8.66 (t, *J* = 5.9 Hz, 1H, 8-H); **^{13}C-NMR, DEPT135, HSQC, HMBC (100 MHz, DMSO-*d*$_6$):** δ(ppm) = 20.2 (11), 28.0 (10), 46.5 (9), 119.4 (6), 122.8 (3), 127.4 (1), 129.7 (4), 145.3 (5), 147.9 (2), 165.9 (7).

2-Bromo-*N*-(4-fluorobenzyl)-4-nitrobenzamide (**25b**)

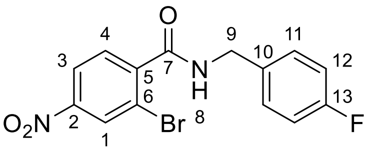

^{1}H-NMR (400 MHz, DMSO-*d*$_6$): δ(ppm) = 4.47 (d, *J* = 5.92 Hz, 2H, 9-H), 7.19 (m, 2H, 12-H), 7.42 (m, 2H, 11-H), 7.70 (d, *J* = 8.4 Hz, 1H, 4-H), 8.28 (dd, *J* = 8.4, 2.2 Hz, 1H, 3-H), 8.46 (d, *J* = 2.2 Hz, 1H, 1-H), 9.20 (t, *J* = 6.0 Hz, 1H, 8-H); **^{13}C-NMR, DEPT135, HSQC, HMBC (100 MHz, DMSO-*d*$_6$):** δ(ppm) = 41.8 (10), 115.1 (d, *J* = 21.4 Hz, 12), 119.5 (6), 122.8 (3), 127.5 (1), 129.4 (d, *J* = 8.1 Hz, 11), 129.8 (4), 134.8 (d, *J* = 3.0 Hz, 10), 144.6 (5), 148.1 (2), 161.3 (d, *J* = 242.6 Hz, 13), 165.9 (7).

N-Isobutyl-2-methoxy-4-nitrobenzamide (**26a**)

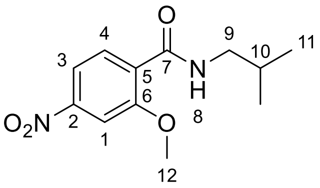

^{1}H-NMR (400 MHz, DMSO-*d*$_6$): δ(ppm) = 0.91 (d, *J* = 6.7 Hz, 6H, 11-H), 1.82 (m, 1H, 10-H), 3.10 (dd, *J* = 6.9, 6.0 Hz, 2H, 9-H), 3.97 (s, 3H, 12-H) 7.75 (m, 1H, 4-H), 7.86 (s, 1H, 1-H), 7.87 (m, 1H, 3-H), 8.34 (t, *J* = 5.6 Hz, 1H, 8-H); **^{13}C-NMR, DEPT135, HSQC, HMBC (100 MHz, DMSO-*d*$_6$):** δ(ppm) = 20.0 (11), 28.0 (10), 46.5 (9), 56.5 (12), 106.7 (1), 115.4 (3), 130.5 (4), 131.3 (5), 149.1 (2), 156.8 (6), 164.0 (7).

N-(4-Fluorobenzyl)-2-methoxy-4-nitrobenzamide (**26b**)

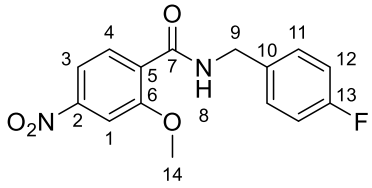

^{1}H-NMR (400 MHz, DMSO-*d*$_6$): δ(ppm) = 3.99 (s, 3H, 14-H), 4.47 (d, *J* = 6.1 Hz, 2H, 9-H), 7.17 (m, 2H, 12-H), 7.38 (m, 2H, 11-H), 7.82 (d, *J* = 8.9 Hz, 1H, 4-H), 7.88 (m, 2H, 1-H, 3-H), 8.93 (t, *J* = 6.0 Hz, 1H, 8-H); **^{13}C-NMR, DEPT135, HSQC, HMBC (100 MHz, DMSO-*d*$_6$):** δ(ppm) = 41.9 (9), 56.6 (14), 106.9 (1), 115.0 (d, *J* = 21.3 Hz, 12), 115.4 (3), 129.0 (d, *J* = 8.1 Hz, 11), 130.5 (5), 130.7 (4), 135.4 (d, *J* = 3.0 Hz, 10), 149.3 (2), 157.0 (6), 161.2 (d, *J* = 242.1 Hz, 13), 164.1 (7).

4-Amino-*N*-isobutyl-2-methoxybenzamide (**27a**)

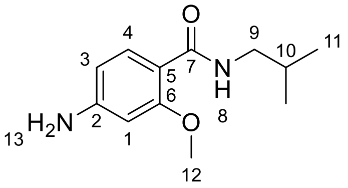

^{1}H-NMR (400 MHz, DMSO-*d*$_6$): δ(ppm) = 0.88 (d, *J* = 6.7 Hz, 6H, 11-H), 1.78 (m, 1H, 10-H), 3.09 (dd, *J* = 6.8, 5.8 Hz, 2H, 9-H), 3.82 (s, 3H, 12-H), 5.68 (s, 2H, 13-H), 6.19 (dd, *J* = 8.4, 2.0 Hz, 1H, 3-H), 6.24 (d, *J* = 2.0 Hz, 1H, 1-H), 7.60 (d, *J* = 8.4 Hz, 1H, 4-H), 7.81 (t, *J* = 5.8 Hz, 1H, 8-H); **^{13}C-NMR, DEPT135, HSQC, HMBC (100 MHz, DMSO-*d*$_6$):** δ(ppm) = 20.1 (11), 28.1 (10), 46.2 (9), 55.5 (12), 96.1 (1), 106.1 (3), 109.2 (5), 132.5 (3), 153.1 (2), 158.8 (6), 164.7 (7).

4-Amino-*N*-(4-fluorobenzyl)-2-methoxybenzamide (**27b**)

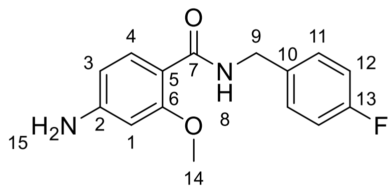

^{1}H-NMR (400 MHz, DMSO-*d*$_6$): δ(ppm) = 3.81 (s, 3H, 14-H), 4.44 (d, *J* = 6.1 Hz, 2H, 9-H), 5.72 (s, 2H, 15-H), 6.19 (dd, *J* = 8.6, 2.0 Hz, 1H, 3-H), 6.24 (d, *J* = 2.0 Hz, 1H, 1-H), 7.13 (m, 2H, 12-H), 7.32 (m, 2H, 11-H), 7.63 (d, *J* = 8.5 Hz, 1H, 3-H), 8.35 (t, *J* = 6.1 Hz, 1H, 8-H); **^{13}C-NMR, DEPT135, HSQC, HMBC (100 MHz, DMSO-*d*$_6$):** δ(ppm) = 41.7 (9), 55.4 (14), 95.9 (1), 106.1 (3), 108.7 (5), 114.9 (d, *J* = 21.1 Hz, 12), 129.0 (d, *J* = 8.0 Hz, 11), 132.6 (4), 136.6 (d, *J* = 2.9 Hz, 10), 153.3 (2), 159.1 (6), 161.0 (d, *J* = 241.7 Hz, 13), 164.9 (7).

4-[(4-Fluorobenzyl)amino]-*N*-isobutyl-2-methoxybenzamide (**28a**)

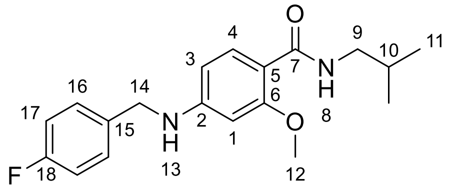

^{1}H-NMR (400 MHz, DMSO-*d*$_6$): δ(ppm) = 0.87 (d, *J* = 6.7 Hz, 6H, 11-H), 1.77 (m, 1H, 10-H), 3.08 (dd, *J* = 6.8, 5.9 Hz, 2H, 9-H), 3.80 (s, 3H, 12-H), 4.31 (d, *J* = 6.0 Hz, 2H, 14-H), 6.22 (dd, *J* = 8.5, 2.1 Hz, 1H, 3-H), 6.25 (d, *J* = 2.1 Hz, 1H, 1-H), 6.83 (t, *J* = 6.1 Hz, 1H, 13-H), 7.15 (m, 2H, 17-H), 7.39 (m, 2H, 16-H), 7.61 (d, *J* = 8.5 Hz, 1H, 4-H), 7.80 (t, *J* = 5.8 Hz, 1H, 8-H); **^{13}C-NMR, DEPT135, HSQC, HMBC (100 MHz, DMSO-*d*$_6$):** δ(ppm) = 20.1 (11), 28.1 (10), 45.3 (14), 46.2 (9), 55.5 (12), 95.0 (1), 104.5 (3), 109.6 (5), 115.0 (d, *J* = 21.2 Hz, 17), 129.1 (d, *J* = 8.1 Hz, 16), 132.3 (4), 135.8 (d, *J* = 3.1 Hz, 15), 152.3 (2), 158.7 (6), 161.1 (d, *J* = 242.0 Hz, 18), 164.7 (7).

N-(4-Fluorobenzyl)-4-[(4-fluorobenzyl)amino]-2-methoxybenzamide (**28b**)

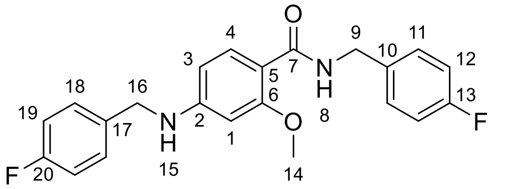

^{1}H-NMR (400 MHz, DMSO-*d*$_6$): δ(ppm) = 3.79 (s, 3H, 14-H), 4.32 (d, *J* = 6.0 Hz, 2H, 16-H), 4.43 (d, *J* = 6.1 Hz, 2H, 9-H), 6.24 (m, 2H, 1-H, 3-H), 6.87 (t, *J* = 6.0 Hz, 1H, 8-H), 7.13 (m, 4H, 12-H, 19-H), 7.31 (m, 2H, 11-H), 7.39 (m, 2H, 18-H), 7.64 (d, *J* = 8.4 Hz, 1H, 4-H), 8.35 (t, *J* = 6.1 Hz, 1H, 8-H); **^{13}C-NMR, DEPT135, HSQC, HMBC (100 MHz, DMSO-*d*$_6$):** δ(ppm) = 41.7 (9), 45.3 (16), 55.4 (14), 94.9 (1), 104.5 (3), 109.2 (5), 114.8 (d, *J* = 18.5 Hz, 12), 115.1 (d, *J* = 18.5 Hz, 19), 129.0 (d, *J* = 8.1 Hz, 11), 129.2 (d, *J* = 8.1 Hz, 18), 132.5 (4), 135.8 (d, *J* = 2.9 Hz, 17), 136.5 (d, *J* = 2.9 Hz, 10), 152.6 (2), 158.9 (6), 161.0 (d, *J* = 240.0 Hz, 20), 161.1 (d, *J* = 240.0 Hz, 13), 164.8 (7).

2,6-Dihydroxy-4-methylnicotinonitrile (**31**)

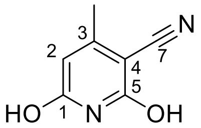

^{1}H-NMR (400 MHz, DMSO-*d*$_6$): δ(ppm) = 2.09 (s, 3H, 6-H), 5.25 (s, 1H, 2-H); **^{13}C-NMR, DEPT135, HSQC, HMBC (100 MHz, DMSO-*d*$_6$):** δ(ppm) = 20.6 (6), 82.7 (4), 95.6 (2), 119.2 (7), 157.1 (3), 162.9 (1), 163.4 (5).

2,6-Dichloro-4-methylnicotinic acid (**33**)

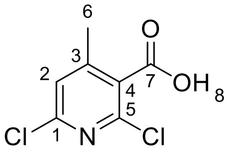

^{1}H-NMR (400 MHz, DMSO-d_6): δ(ppm) = 2.35 (s, 3H, 6-H), 7.61 (s, 1H, 2-H), 14.22 (s, 1H, 8-H); **^{13}C-NMR, DEPT135, HSQC, HMBC (100 MHz, DMSO-d_6):** δ(ppm) = 18.7 (6), 124.9 (2), 130.5 (4), 144.6 (5), 148.7 (1), 150.7 (3), 166.0 (7).

6-Chloro-2-methoxy-4-methylnicotinic acid (**34**)

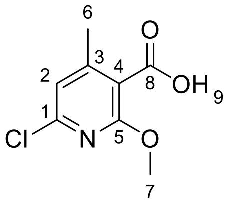

^{1}H-NMR (400 MHz, DMSO-d_6): δ(ppm) = 2.17 (s, 3H, 6-H), 3.87 (s, 3H, 7-H), 7.09 (s, 1H, 2-H), 13.39 (s, 1H, 9-H); **^{13}C-NMR, DEPT135, HSQC, HMBC (100 MHz, DMSO-d_6):** δ(ppm) = 18.4 (6), 54.3 (7), 117.4 (2), 118.0 (4), 147.0 (1), 149.8 (3), 159.4 (5), 166.6 (8).

N-Butyl-6-chloro-2-methoxy-4-methylnicotinamide (**35a**)

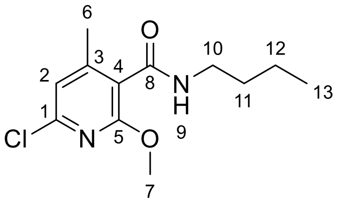

^{1}H-NMR (400 MHz, DMSO-d_6): δ(ppm) = 0.89 (t, *J* = 7.3 Hz, 3H, 13-H), 1.34 (m, 2H, 12-H), 1.45 (m, 2H, 11-H), 2.19 (s, 3H, 6-H), 3.19 (td, *J* = 6.9, 5.7 Hz, 2H, 10-H), 3.82 (s, 3H, 7-H). 7.04 (s, 1H, 2-H), 8.30 (t, *J* = 5.7 Hz, 1H, 9-H); **^{13}C-NMR, DEPT135, HSQC, HMBC (100 MHz, DMSO-d_6):** δ(ppm) = 13.6 (13), 18.0 (6), 19.5 (12), 31.0 (11), 38.3 (10), 54.0 (7), 117.8 (2), 120.4 (4), 146.1 (1), 149.7 (3), 159.6 (5), 164.1 (8).

6-Chloro-*N*-(4-fluorobenzyl)-2-methoxy-4-methylnicotinamide (**35b**)

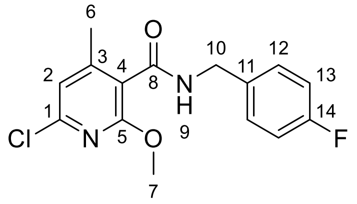

^{1}H-NMR (400 MHz, DMSO-d_6): δ(ppm) = 2.18 (d, *J* = 0.6 Hz, 3H, 6-H), 3.86 (s, 3H, 7-H). 4.42 (d, *J* = 6.0 Hz, 2H, 10-H), 7.05 (d, *J* = 0.6 Hz, 1H, 2-H), 7.18 (m, 2H, 13-H), 7.38 (m, 2H, 12-H), 8.89 (t, *J* = 6.0 Hz, 1H, 9-H); **^{13}C-NMR, DEPT135, HSQC, HMBC (100 MHz, DMSO-d_6):** δ(ppm) = 18.0 (6), 41.5 (10), 54.1 (7), 115.0 (d, *J* = 21.3 Hz, 13), 117.9 (2), 119.9 (4), 129.1 (d, *J* = 8.1 Hz, 12), 135.3 (d, *J* = 3.0 Hz, 11), 146.4 (1), 149.9 (3), 159.6 (5), 161.2 (d, *J* = 242.1 Hz, 14), 164.5 (8).

N-Butyl-6-[(4-fluorobenzyl)amino]-2-methoxy-4-methylnicotinamide (**36a**)

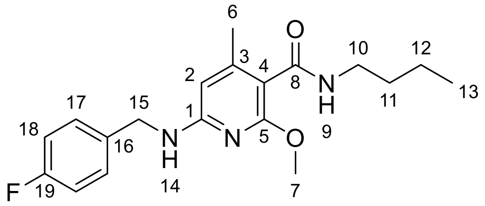

^{1}H-NMR (400 MHz, DMSO-d_6): δ(ppm) = 0.87 (t, *J* = 7.3 Hz, 3H, 13-H), 1.31 (m, 2H, 12-H), 1.41 (m, 2H, 11-H), 2.04 (s, 3H, 6-H), 3.12 (td, *J* = 6.8, 5.7 Hz, 2H, 10-H), 3.69 (s, 3H, 7-H), 4.42 (d, *J* = 6.1 Hz, 2H, 15-H), 5.88 (s, 1H, 2-H), 7.11 (m, 2H, 18-H), 7.16 (t, *J* = 5.7 Hz, 1H, 14-H), 7.35 (m, 2H, 17-H), 7.84 (t, *J* = 5.7 Hz, 1H, 9-H); **^{13}C-NMR, DEPT135, HSQC, HMBC (100 MHz, DMSO-d_6):** δ(ppm) = 13.7 (13), 18.9 (6), 19.5 (12), 31.2 (11), 38.3 (10), 43.5 (15), 52.6 (7), 100.0 (2), 108.3 (4), 114.8 (d, *J* = 21.1 Hz, 18), 129.0 (d, *J* = 8.0 Hz, 17), 137.1 (d, *J* = 3.0 Hz, 16), 147.5 (3), 156.5 (1), 159.3 (5), 161.0 (d, *J* = 241.65 Hz, 19), 166.0 (8).

N-(4-Fluorobenzyl)-6-[(4-fluorobenzyl)amino]-2-methoxy-4-methylnicotinamide (**36b**)

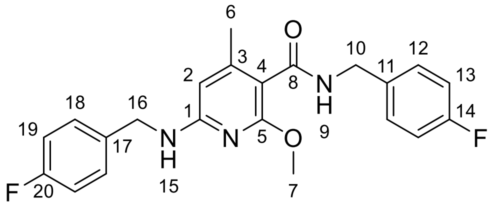

^{1}H-NMR (400 MHz, DMSO-d_6): δ(ppm) = 2.04 (s, 3H, 6-H), 3.73 (s, 3H, 7-H), 4.35 (d, *J* = 6.1 Hz, 2H, 10-H), 4.43 (d, *J* = 6.0 Hz, 2H, 16-H), 5.90 (s, 1H, 2-H), 7.14 (m, 4H, 13-H, 19-H), 7.22 (t, *J* = 6.2 Hz, 1H, 15-H), 7.36 (m, 4H, 12-H, 18-H), 8.44 (t, *J* = 6.2 Hz, 1H, 9-H); **^{13}C-NMR, DEPT135, HSQC, HMBC (100 MHz, DMSO-d_6):** δ(ppm) = 19.0 (6), 41.5 (10), 43.5 (16), 52.7 (7), 100.2 (2), 107.6 (4), 114.8 (d, *J* = 21.2 Hz,

13, 19), 128.9 (d, *J* = 8.1 Hz, 12), 129.1 (d, *J* = 8.1 Hz, 18), 136.0 (d, *J* = 2.9 Hz, 11), 137.0 (d, *J* = 2.9 Hz, 17), 147.8 (3), 156.7 (1), 159.4 (5), 161.0 (d, *J* = 240.0 Hz, 14), 161.0 (d, *J* = 240.0 Hz, 20), 166.3 (8).

N-(4-Fluorobenzyl)-6-[(4-fluorobenzyl)amino]-4-methyl-2-oxo-1,2-dihydropyridine-3-carboxamide (**37**)

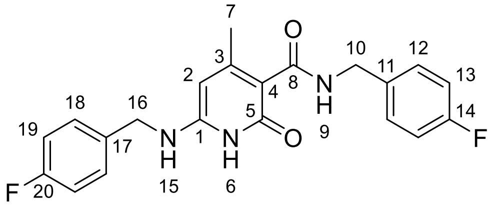

^{1}H-NMR (400 MHz, DMSO-*d*$_6$): δ(ppm) = 2.43 (s, 3H, 7-H), 4.38 (d, *J* = 5.8 Hz, 2H, 10-H), 4.40 (d, *J* = 6.1 Hz, 2H, 16-H), 5.45 (s, 1H, 2-H), 7.06 (t, *J* = 5.8 Hz, 1H, 15-H), 7.13 (m, 2H, 13-H), 7.19 (m, 2H, 19-H), 7.31 (m, 2H, 12-H), 7.38 (m, 2H, 12-H), 10.17 (s, 1H, 9-H), 10.97 (s, 1H, 6-H); **^{13}C-NMR, DEPT135, HSQC, HMBC (100 MHz, DMSO-*d*$_6$):** δ(ppm) = 23.8 (7), 41.1 (10), 43.9 (16), 91.8 (2), 103.7 (3), 114.9 (d, *J* = 21.0 Hz, 13), 115.3 (d, *J* = 21.4 Hz, 19), 129.1 (d, *J* = 8.0 Hz, 12), 129.2 (d, *J* = 8.2 Hz, 18), 134.3 (d, *J* = 3.0 Hz, 17), 136.5 (d, *J* = 3.0 Hz, 11), 150.6 (1), 161.0 (d, *J* = 241.0 Hz, 14), 161.4 (d, *J* = 242.0 Hz, 17), 162.8 (5), 166.3 (8), (signal of 4 is missing, perhaps due to peak overlap).

Methyl 4-amino-2-bromobenzoate (**39**)

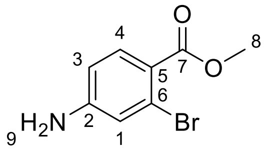

^{1}H-NMR (400 MHz, DMSO-*d*$_6$): δ(ppm) = 3.73 (s, 3H, 8-H), 6.14 (s, 2H, 9-H), 6.55 (dd, *J* = 8.6, 2.3 Hz, 1H, 3-H), 6.86 (d, *J* = 2.2 Hz, 1H, 1-H), 7.63 (d, *J* = 8.6 Hz, 1H, 4-H); **^{13}C-NMR, DEPT135, HSQC, HMBC (100 MHz, DMSO-*d*$_6$):** δ(ppm) = 51.5 (8), 111.9 (3), 115.5 (2), 118.0 (1), 123.1 (5), 133.5 (4), 153.4 (6), 165.0 (7).

Methyl 2-bromo-4-[(4-fluorobenzyl)amino]benzoate (**40**)

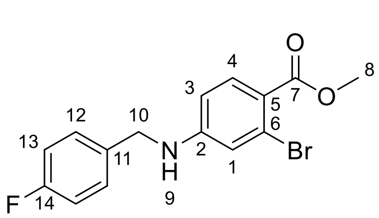

^{1}H-NMR (400 MHz, DMSO-*d*$_6$): δ(ppm) = 3.73 (s, 3H, 8-H), 4.33 (d, *J* = 5.9 Hz, 2H, 10-H), 6.61 (dd, *J* = 8.8, 2.4 Hz, 1H, 3-H), 6.87 (d, *J* = 2.4 Hz, 1H, 1-H), 7.17 (m, 2H, 13-H), 7.26 (t, *J* = 6.0 Hz, 1H, 9-H), 7.37 (m, 2H, 12-H), 7.65 (d, *J* = 8.7 Hz, 1H, 4-H); **^{13}C-NMR, DEPT135, HSQC, HMBC (100 MHz, DMSO-*d*$_6$):**

δ(ppm) = 44.9 (19), 51.5 (8), 110.6 (3), 115.2 (d, *J* = 21.3 Hz, 13), 116.1 (1), 116.8 (6), 123.2 (5), 129.1 (d, *J* = 8.1 Hz, 12), 133.3 (4), 135.0 (d, *J* = 2.9 Hz, 11), 152.4 (2), 161.2 (d, *J* = 242.4 Hz, 14), 165.0 (7).

Methyl 2-bromo-4-[(*tert*-butoxycarbonyl)(4-fluorobenzyl)amino]benzoate (**41**)

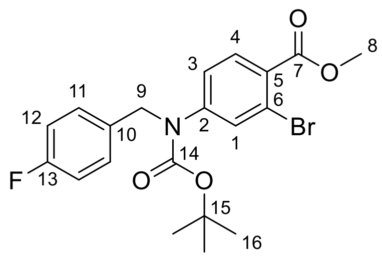

^{1}H-NMR (400 MHz, DMSO-d_6): δ(ppm) = 1.40 (s, 9H, 16-H), 3.82 (s, 3H, 8-H), 4.91 (s, 2H, 9-H), 7.14 (m, 2H, 12-H), 7.24 (m, 2H, 11-H), 7.36 (dd, *J* = 8.5, 2.2 Hz, 1H, 3-H), 7.67 (d, *J* = 2.1 Hz, 1H, 1-H), 7.72 (d, *J* = 8.5 Hz, 1H, 4-H); **^{13}C-NMR, DEPT135, HSQC, HMBC (100 MHz, DMSO-d_6):** δ(ppm) = 27.7 (16), 51.2 (9), 52.5 (8), 81.22 (15), 115.3 (d, *J* = 21.3 Hz, 12), 120.1 (6), 124.6 (3), 128.2 (5), 129.0 (d, *J* = 8.3 Hz, 11), 130.6 (1), 131.1 (4), 133.9 (d, *J* = 3.0 Hz, 10), 145.5 (2), 153.2 (14), 161.3 (d, *J* = 243.0 Hz, 13), 165.5 (7).

Methyl 4-[(*tert*-butoxycarbonyl)(4-fluorobenzyl)amino]-2-[(trimethylsilyl)ethynyl]benzoate (**42**)

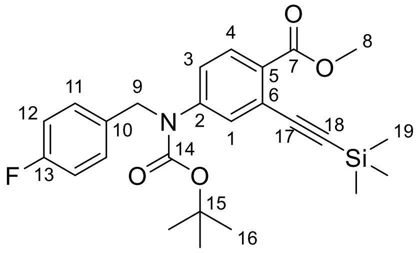

^{1}H-NMR (400 MHz, DMSO-d_6): δ(ppm) = 0.23 (s, 9H, 19-H), 1.39 (s, 9H, 16-H), 3.81 (s, 3H, 8-H), 4.90 (s, 2H, 9-H), 7.14 (m, 2H, 12-H), 7.23 (m, 2H, 11-H), 7.36 (dd, *J* = 8.6, 2.3 Hz, 1H, 3-H), 7.47 (d, *J* = 2.2 Hz, 1H, 1-H), 7.79 (d, *J* = 8.6 Hz, 1H, 4-H); **^{13}C-NMR, DEPT135, HSQC, HMBC (100 MHz, DMSO-d_6):** δ(ppm) = -0.27 (19), 27.7 (16), 51.2 (10), 52.0 (8), 80.9 (15), 99.6 (17), 102.8 (18), 115.3 (d, *J* = 21.4 Hz, 12), 122.5 (5), 126.0 (3), 128.7 (6), 129.0 (d, *J* = 8.1 Hz, 11), 130.6 (4), 130.6 (1), 134.0 (d, *J* = 3.0 Hz, 10), 145.0 (2), 153.2 (15), 161.3 (d, *J* = 242.8 Hz, 13), 165.4 (7).

Methyl 4-[(*tert*-butoxycarbonyl)(4-fluorobenzyl)amino]-2-ethynylbenzoate (**43**)

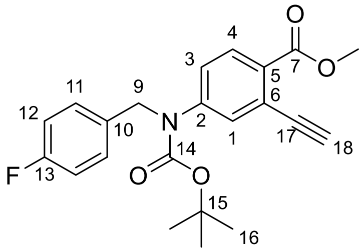

^{1}H-NMR (400 MHz, DMSO-d_6): δ(ppm) = 1.38 (s, 9H, 16-H), 3.79 (s, 3H, 8-H), 4.40 (s, 1H, 18-H), 4.89 (s, 2H, 9-H), 7.12 (m, 2H, 12-H), 7.21 (m, 2H, 11-H), 7.37 (dd, *J* = 8.6, 2.3 Hz, 1H, 3-H), 7.48 (d, *J* = 2.3 Hz, 1H, 1-H), 7.77 (d, *J* = 8.6 Hz, 1H, 4-H); **^{13}C-NMR, DEPT135, HSQC, HMBC (100 MHz, DMSO-d_6):** δ(ppm) = 27.7 (16), 51.2 (9), 52.1 (8), 81.0 (15), 81.2 (17), 85.7 (18), 115.3 (d, *J* = 21.6 Hz, 12), 122.2 (5), 126.0 (3), 128.7 (6), 128.9 (d, *J* = 8.4 Hz, 11), 130.5 (4), 130.9 (1), 134.0 (d, *J* = 3.0 Hz, 10), 145.0 (2), 153.2 (14), 161.3 (d, *J* = 242.8 Hz, 13), 165.2 (7).

Methyl 4-[(*tert*-butoxycarbonyl)(4-fluorobenzyl)amino]-2-ethylbenzoate (**44**)

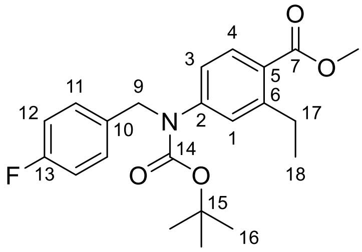

^{1}H-NMR (400 MHz, DMSO-d_6): δ(ppm) = 1.09 (t, *J* = 7.5 Hz, 3H, 18-H), 1.39 (s, 9H, 16-H), 2.84 (q, *J* = 7.5 Hz, 2H, 17-H), 3.79 (s, 3H, 8-H), 4.88 (s, 2H, 9-H), 7.17 (m, 6H, 1-H, 3-H, 11-H, 12-H), 7.70 (d, *J* = 8.4 Hz, 1H, 4-H); **^{13}C-NMR, DEPT135, HSQC, HMBC (100 MHz, DMSO-d_6):** δ(ppm) = 15.8 (18), 26.7 (17), 27.8 (16), 51.4 (9), 51.9 (8), 80.5 (15), 115.2 (d, *J* = 21.3 Hz, 12), 123.0 (3), 125.6 (5), 127.2 (1), 129.0 (d, *J* = 8.2 Hz, 11), 130.7 (4), 134.3 (d, *J* = 3.0 Hz, 10), 145.1 (1), 145.8 (2), 153.5 (14), 161.2 (d, *J* = 242.5 Hz, 13), 166.8 (7).

4-[(*tert*-Butoxycarbonyl)(4-fluorobenzyl)amino]-2-ethylbenzoic acid (**45**)

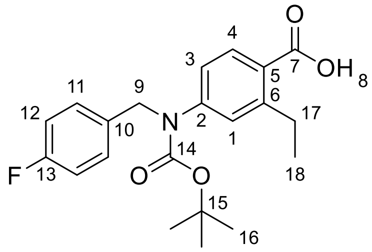

^{1}H-NMR (400 MHz, DMSO-*d*$_6$): δ(ppm) = 1.10 (t, *J* = 7.5 Hz, 3H, 18-H), 1.40 (s, 9H, 16-H), 2.87 (q, *J* = 7.5 Hz, 2H, 17-H), 4.88 (s, 2H, 9-H), 7.14 (m, 4H, 1-H, 3-H, 12-H), 7.23 (m, 2H, 11-H), 7.71 (d, *J* = 8.4 Hz, 1H, 4-H); **^{13}C-NMR, DEPT135, HSQC, HMBC (100 MHz, DMSO-*d*$_6$):** δ(ppm) = 15.9 (18), 26.8 (17), 27.8 (16), 51.5 (9), 80.5 (15), 115.2 (d, *J* = 21.4 Hz, 12), 122.9 (3), 126.8 (5), 127.2 (1), 129.0 (d, *J* = 8.2 Hz, 11), 130.9 (4), 134.4 (d, *J* = 3.0 Hz, 10), 144.8 (6), 145.7 (2), 153.5 (14), 161.2 (d, *J* = 242.7 Hz, 13), 168.1 (7).

tert-Butyl [3-ethyl-4-(propylcarbamoyl)phenyl](4-fluorobenzyl)carbamate (**46**)

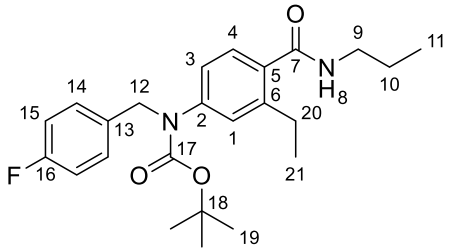

^{1}H-NMR (400 MHz, DMSO-*d*$_6$): δ(ppm) = 0.88 (t, *J* = 7.4 Hz, 3H, 11-H), 1.08 (t, *J* = 7.5 Hz, 3H, 21-H), 1.40 (s, 9H, 19-H), 1.48 (m, 2H, 10-H), 2.64 (q, *J* = 7.5 Hz, 2H, 20-H), 3.15 (td, *J* = 6.9, 5.7 Hz, 2H, 9-H), 4.84 (s, 2H, 12-H), 7.14 (m, 7H, 1-H, 3-H, 4-H, 14-H, 15-H), 8.24 (t, *J* = 5.7 Hz, 1H, 8-H); **^{13}C-NMR, DEPT135, HSQC, HMBC (100 MHz, DMSO-*d*$_6$):** δ(ppm) = 11.4 (11), 15.7 (21), 22.3 (10), 25.7 (20), 27.9 (19), 40.6 (9), 51.8 (12), 80.2 (18), 115.2 (d, *J* = 21.3 Hz, 15), 122.9 (3), 126.7 (1), 127.4 (4), 129.0 (d, *J* = 8.3 Hz, 14), 134.3 (7), 134.5 (d, *J* = 3.0 Hz, 13), 142.0 (6), 142.5 (2), 153.8 (17), 161.2 (d, *J* = 242.5 Hz, 16), 168.6 (7).

2-Ethyl-4-[(4-fluorobenzyl)amino]-*N*-propylbenzamide hydrochloride (**47**)

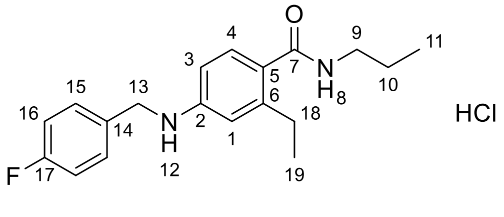

^{1}H-NMR (400 MHz, MeOH-*d*$_4$): δ(ppm) = 1.01 (t, *J* = 7.4 Hz, 3H, 11-H), 1.21 (t, *J* = 7.6 Hz, 3H, 19-H), 1.66 (m, 2H, 10-H), 2.80 (q, *J* = 7.6 Hz, 2H, 18-H), 3.35 (m, 2H, 9-H), 4.63 (s, 2H, 13-H), 7.17 (m, 2H, 16-H),

7.26 (m, 2H, 1-H, 3-H), 7.46 (m, 3H, 4-H, 15-H); **^{13}C-NMR, DEPT135, HSQC, HMBC (100 MHz, MeOH-*d*$_4$):** δ(ppm) = 12.0 (11), 16.0 (19), 23.7 (10), 27.3 (18), 42.8 (9), 55.5 (13), 117.1 (d, *J* = 22.0 Hz, 16), 121.2 (3), 124.6 (1), 128.6 (14), 130.2 (4), 133.9 (d, *J* = 8.8 Hz, 15), 137.8 (2), 138.8 (6), 146.0 (5), 165.0 (d, *J* = 248.0 Hz, 17), 171.7 (7).

4-Methyl-2,6-dimorpholinonicotinonitrile (**53**)

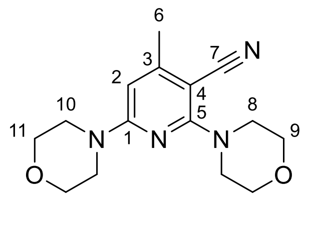

^{1}H-NMR (400 MHz, DMSO-*d*$_6$): δ(ppm) = 2.28 (d, *J* = 0.7 Hz, 3H, 6-H), 3.49 (m, 4H, 10-H), 3.55 (m, 4H, 8-H), 3.65 (m, 4H, 11-H), 3.69 (m, 4H, 9-H), 6.34 (d, *J* = 0.9 Hz, 1H, 2-H); **^{13}C-NMR, DEPT135, HSQC, HMBC (100 MHz, DMSO-*d*$_6$):** δ(ppm) = 20.5 (6), 44.3 (8), 48.4 (10), 65.8 (9), 65.9 (11), 82.4 (4), 100.0 (2), 118.5 (7), 153.6 (3), 157.9 (1), 161.4 (5).

(4-Methyl-2,6-dimorpholinopyridin-3-yl)methanamine (**54**)

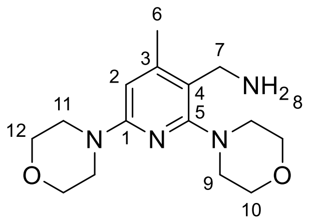

^{1}H-NMR (400 MHz, DMSO-*d*$_6$): δ(ppm) = 2.30 (s, 3H, 6-H), 2.93 (m, 4H, 9-H), 3.43 (m, 4H, 11-H), 3.68 (m, 4H, 12-H), 3.70 (m, 4H, 10-H), 3.94 (s, 2H, 7-H), 6.45 (s, 1H, 2-H), 7.86 (s, 2H, 8-H); **^{13}C-NMR, DEPT135, HSQC, HMBC (100 MHz, DMSO-*d*$_6$):** δ(ppm) = 19.6 (6), 35.0 (7), 44.9 (11), 51.3 (9), 65.9 (12), 66.3 (10), 103.7 (2), 110.4 (4), 150.3 (3), 157.7 (1), 160.7 (5).

4-Fluoro-*N*-[(4-methyl-2,6-dimorpholinopyridin-3-yl)methyl]benzamide (**55a**)

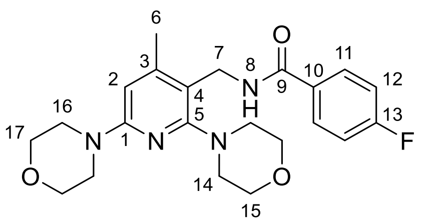

^{1}H-NMR (400 MHz, DMSO-*d*$_6$): δ(ppm) = 2.21 (s, 3H, 6-H), 3.00 (m, 4H, 14-H), 3.40 (m, 4H, 16-H), 3.69 (m, 8H, 15-H, 17-H), 4.43 (d, *J* = 4.2 Hz, 2H, 7-H), 6.39 (s, 1H, 2-H), 7.25 (m, 2H, 12-H), 7.93 (m, 2H, 11-H), 8.40 (t, *J* = 4.2 Hz, 1H, 8-H); **^{13}C-NMR, DEPT135, HSQC, HMBC (100 MHz, DMSO-*d*$_6$):** δ(ppm) = 19.4

(6), 37.2 (7), 45.2 (16), 51.3 (14), 66.0 (15), 66.3 (16), 103.0 (2), 112.0 (4), 115.0 (d, *J* = 21.6 Hz, 12), 130.0 (d, *J* = 8.9 Hz, 11), 130.8 (d, *J* = 2.9 Hz, 10), 150.5 (3), 157.0 (1), 160.3 (5), 163.8 (d, *J* = 248.1 Hz, 13), 165.3 (9).

2-(3,5-Difluorophenyl)-*N*-[(4-methyl-2,6-dimorpholinopyridin-3-yl)methyl]acetamide (**55b**)

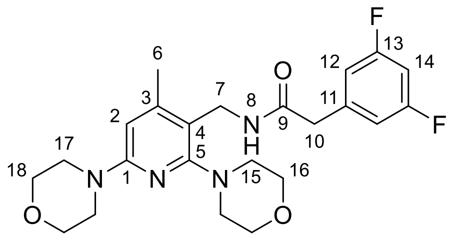

^{1}H-NMR (400 MHz, DMSO-*d*$_6$): δ(ppm) = 2.15 (s, 3H, 6-H), 2.91 (m, 4H, 14-H), 3.38 (m, 4H, 17-H), 3.46 (s, 2H, 10-H), 3.60 (m, 4H, 16-H), 3.67 (m, 4H, 18-H), 4.18 (d, *J =* 4.2 Hz, 2H, 7-H), 6.38 (s, 1H, 2-H), 6.96 (m, 2H, 12-H), 7.09 (m, 1H, 14-H), 8.08 (t, *J =* 4.3 Hz, 1H, 8-H); **^{13}C-NMR, DEPT135, HSQC, HMBC (100 MHz, DMSO-*d*$_6$):** δ(ppm) = 19.2 (6), 36.5 (7), 41.6 (10), 45.2 (17), 51.3 (15), 65.9 (18), 66.2 (16), 101.8 (t, *J =* 25.7 Hz, 14), 102.9 (2), 111.8 (4), 112.0 (dd, *J =* 18.3, 6.5 Hz, 12), 140.9 (t, *J =* 9.9 Hz, 11), 150.5 (3), 157.1 (1), 160.3 (5), 162.1 (dd, *J =* 245.5, 13.5 Hz, 13), 168.9 (9).

4,4'-(4-Methylpyridine-2,6-diyl)dimorpholine (**56**)

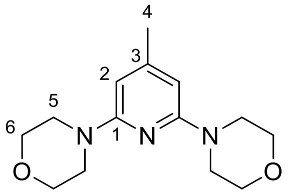

^{1}H-NMR (400 MHz, DMSO-*d*$_6$): δ(ppm) = 2.14 (s, 3H, 4-H), 3.34 (m, 8H, 5-H), 3.66 (m, 8H, 6-H), 5.96 (s, 2H, 2-H); **^{13}C-NMR, DEPT135, HSQC, HMBC (100 MHz, DMSO-*d*$_6$):** δ(ppm) = 21.4 (4), 45.2 (5), 66.0 (6), 97.0 (2), 149.3 (3), 158.1 (1).

6-Amino-2-chloro-4-methylnicotinonitrile (**57**)

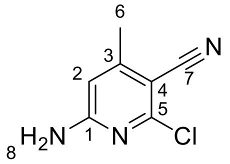

^{1}H-NMR (400 MHz, DMSO-*d*$_6$): δ(ppm) = 2.29 (s, 3H, 6-H), 6.33 (s, 1H, 2-H), 7.37 (s, 2H, 8-H); **^{13}C-NMR, DEPT135, HSQC, HMBC (100 MHz, DMSO-*d*$_6$):** δ(ppm) = 19.9 (6), 95.1 (4), 106.6 (2), 116.1 (7), 151.9 (1), 152.7 (3), 160.9 (5).

2-Amino-6-chloro-4-methylnicotinonitrile (**58**)

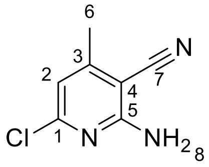

^{1}H-NMR (400 MHz, DMSO-*d*$_6$): δ(ppm) = 2.32 (s, 3H, 6-H), 6.68 (s, 1H, 2-H), 7.26 (s, 2H, 8-H); **^{13}C-NMR, DEPT135, HSQC, HMBC (100 MHz, DMSO-*d*$_6$):** δ(ppm) = 19.7 (6), 88.9 (4), 112.3 (2), 115.5 (7), 152.8 (1), 156.0 (3), 160.2 (5).

2-Amino-4-methyl-6-morpholinonicotinonitrile (**59**)

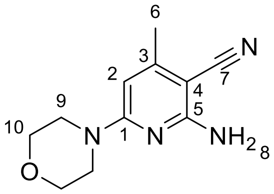

^{1}H-NMR (400 MHz, DMSO-*d*$_6$): δ(ppm) = 2.19 (s, 3H, 6-H), 3.51 (m, 4H, 9-H), 3.62 (m, 4H, 10-H), 6.06 (s, 1H, 2-H), 6.30 (s, 2H, 8-H); **^{13}C-NMR, DEPT135, HSQC, HMBC (100 MHz, DMSO-*d*$_6$):** δ(ppm) = 20.2 (6), 44.3 (9), 65.7 (10), 77.9 (4), 96.4 (2), 118.1 (7), 152.0 (3), 159.0 (1), 160.2 (5).

N-[(2-Amino-4-methyl-6-morpholinopyridin-3-yl)methyl]-2-(3,5-difluorophenyl)acetamide (**61**)

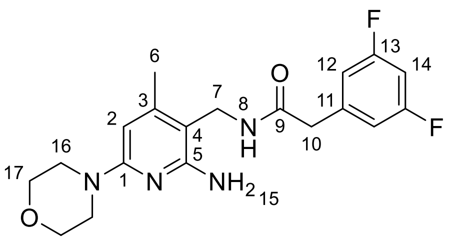

^{1}H-NMR (400 MHz, DMSO-*d*$_6$): δ(ppm) = 2.17 (s, 3H, 6-H), 3.29 (m, 4H, 16-H), 3.48 (s, 2H, 10-H), 3.63 (m, 4H, 17-H), 4.08 (d, *J* = 5.7 Hz, 2H, 7-H), 5.56 (s, 2H, 15-H), 5.85 (s, 1H, 2-H), 6.97 (m, 2H, 12-H), 7.08 (m, 1H, 14-H), 8.38 (t, *J* = 5.7 Hz, 1H, 8-H); **^{13}C-NMR, DEPT135, HSQC, HMBC (100 MHz, DMSO-*d*$_6$):** δ(ppm) = 19.4 (6), 34.9 (7), 41.3 (t, *J* = 2.2 Hz, 10), 45.3 (16), 66.0 (17), 97.0 (2), 101.9 (t, *J* = 25.8 Hz, 14), 104.7 (4), 112.2 (dd, *J* = 18.2 Hz, 6.4 Hz, 12), 140.6 (t, *J* = 9.9 Hz, 11), 148.0 (3), 156.8 (1), 157.4 (5), 162.1 (dd, *J* = 245.4 Hz, 13.5 Hz, 13), 169.7 (9).

3,4-Dimethyl-6-morpholinopyridin-2-amine (**62**)

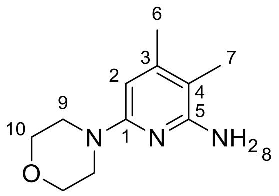

^{1}H-NMR (400 MHz, DMSO-*d*$_6$): δ(ppm) = 1.86 (s, 3H, 7-H), 2.07 (s, 3H, 6-H), 3.24 (m, 4H, 9-H), 3.64 (m, 4H, 10-H), 5.21 (s, 2H, 8-H), 5.83 (s, 1H, 2-H); **^{13}C-NMR, DEPT135, HSQC, HMBC (100 MHz, DMSO-*d*$_6$):** δ(ppm) = 11.6 (7), 20.0 (6), 45.6 (9), 66.1 (10), 97.1 (2), 103.5 (4), 146.3 (5), 156.1 (3), 156.3 (1).

6-Amino-4-methyl-2-morpholinonicotinonitrile (**63**)

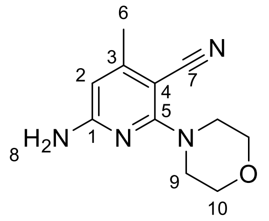

^{1}H-NMR (400 MHz, DMSO-*d*$_6$): δ(ppm) = 2.19 (s, 3H, 6-H), 3.44 (m, 4H, 9-H), 3.67 (m, 4H, 10-H), 5.90 (s, 1H, 2-H), 6.66 (s, 2H, 8-H); **^{13}C-NMR, DEPT135, HSQC, HMBC (100 MHz, DMSO-*d*$_6$):** δ(ppm) = 20.1 (6), 48.5 (9), 66.0 (10), 81.7 (4), 100.5 (2), 118.9 (7), 152.5 (3), 160.0 (5), 162.9 (1).

6-[(4-Fluorobenzyl)amino]-4-methyl-2-morpholinonicotinonitrile (**64**)

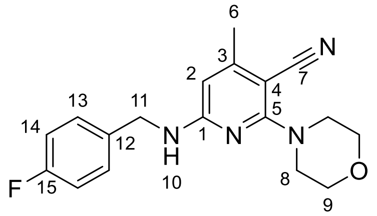

^{1}H-NMR (400 MHz, DMSO-*d*$_6$): δ(ppm) = 2.19 (s, 3H, 6-H), 3.45 (m, 4H, 8-H), 3.63 (m, 4H, 9-H), 4.45 (d, *J* = 5.9 Hz, 2H, 11-H), 5.98 (s, 1H, 2-H), 7.13 (m, 2H, 14-H), 7.32 (m, 2H, 13-H), 7.82 (s, 1H, 10-H); **^{13}C-NMR, DEPT135, HSQC, HMBC (100 MHz, DMSO-*d*$_6$):** δ(ppm) = 20.1 (6), 41.8 (11), 48.3 (8), 65.9 (9), 87.3 (4), 101.0 (2), 114.9 (d, *J* = 21.1 Hz, 14), 118.9 (7), 129.1 (d, *J* = 8.1 Hz, 13), 136.2 (12), 149.7 (3), 158.2 (1), 161.1 (d, *J* = 241.0 Hz, 15), 162.3 (5).

Isobutyl ({6-[(4-fluorobenzyl)amino]-4-methyl-2-morpholinopyridin-3-yl}methyl)carbamate (**66**)

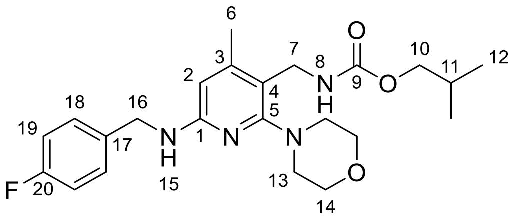

^{1}H-NMR (400 MHz, DMSO-*d*$_6$): δ(ppm) = 0.85 (d, *J* = 6.7 Hz, 6H, 12-H), 1.80 (m, 1H, 11-H), 2.10 (s, 3H, 6-H), 2.89 (m, 4H, 13-H), 3.65 (m, 4H, 14-H), 3.73 (d, *J* = 6.7 Hz, 2H, 10-H), 4.12 (d, *J* = 4.6 Hz, 2H, 7-H), 4.40 (d, *J* = 6.1 Hz, 2H, 16-H), 6.02 (s, 1H, 2-H), 6.85 (t, *J* = 6.2 Hz, 1H, 15-H), 7.06 (s, 1H, 8-H), 7.10 (m, 2H, 19-H), 7.34 (m, 2H, 18-H); **^{13}C-NMR, DEPT135, HSQC, HMBC (100 MHz, DMSO-*d*$_6$):** δ(ppm) = 18.8 (12), 19.0 (6), 27.7 (11), 37.8 (7), 43.4 (16), 51.4 (13), 66.4 (14), 69.5 (10), 103.7 (2), 110.7 (4), 114.7 (d, *J* = 21.2 Hz, 19), 129.1 (d, *J* = 7.9 Hz, 18), 137.4 (17), 137.4 (3), 149.5 (5), 156.3 (1), 160.6 (9), 160.9 (d, *J* = 240.0 Hz, 20).

3 Log$D_{7.4}$ estimation

Table S1: Values used for Calibration A.

Reference	t_R mean [min]	k'	logk'	log*D* (Lit.)
Uracil	1.803			
Acetophenone	2.907	0.612	-0.213	1.7
Benzene	3.399	0.885	-0.053	2.1
Ethyl benzoate	4.077	1.261	0.101	2.6
Benzophenone	5.279	1.928	0.285	3.2
Phenyl benzoate	6.126	2.398	0.380	3.6
Diphenyl ether	7.818	3.336	0.523	4.2
Bibenzyl	12.013	5.663	0.753	4.8
Triphenylamine	28.187	14.633	1.165	5.7

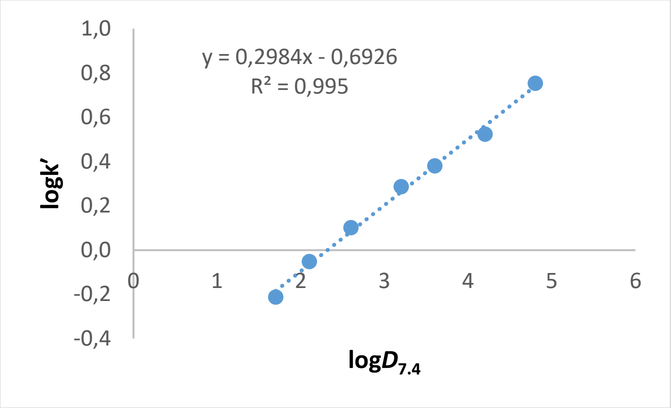

Figure S103: Calibration function A1.

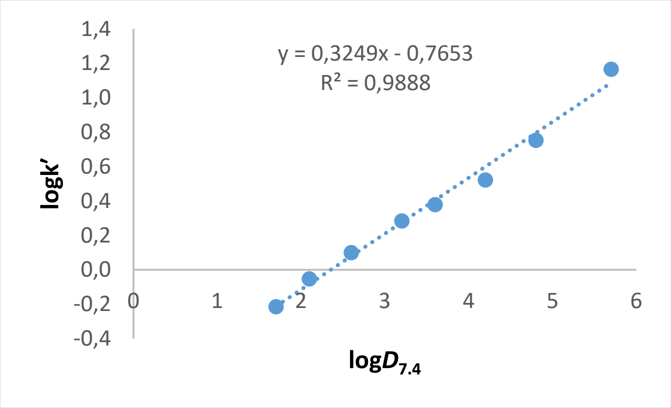

Figure S104: Calibration function A2.

Calibration B

Table S2: Values used for Calibration B.

Reference	t_R mean [min]	k'	logk'	log*D* (Lit.)
Uracil	1.7965			
Acetophenone	2.857	0.585	-0.233	1.7
Benzene	3.323	0.843	-0.074	2.1
Ethyl benzoate	3.955	1.194	0.077	2.6
Benzophenone	5.0765	1.816	0.259	3.2
Phenyl benzoate	5.8465	2.243	0.351	3.6
Diphenyl ether	7.3915	3.100	0.491	4.2
Bibenzyl	11.2105	5.218	0.717	4.8
Triphenylamine	25.6765	13.241	1.122	5.7

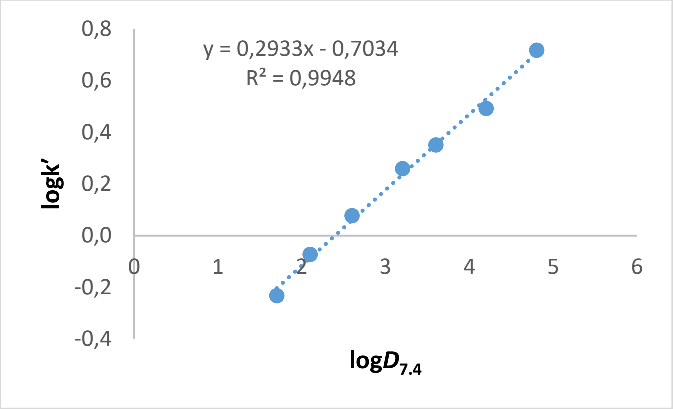

Figure S105: Calibration function B1.

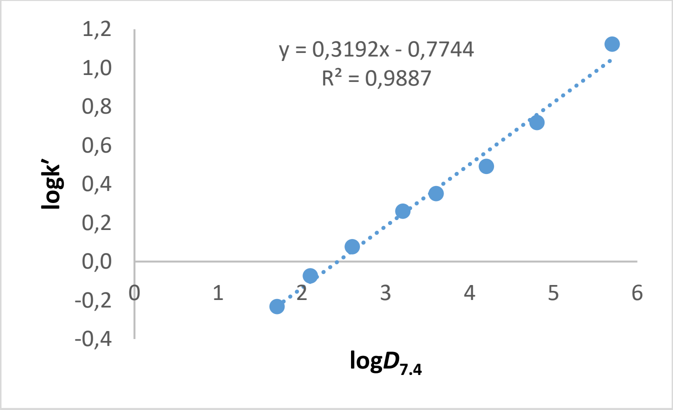

Figure S106: Calibration function B2.

Calibration C

Table S3: Values used for Calibration C.

Reference	t_R mean [min]	k'	logk'	log*D* (Lit.)
Uracil	1.850			
Acetophenone	2.968	0.604	-0.219	1.7
Benzene	3.471	0.876	-0.057	2.1
Ethyl benzoate	4.156	1.246	0.096	2.6
Benzophenone	5.377	1.906	0.280	3.2
Phenyl benzoate	6.226	2.365	0.374	3.6
Diphenyl ether	7.938	3.291	0.517	4.2
Bibenzyl	12.214	5.602	0.748	4.8

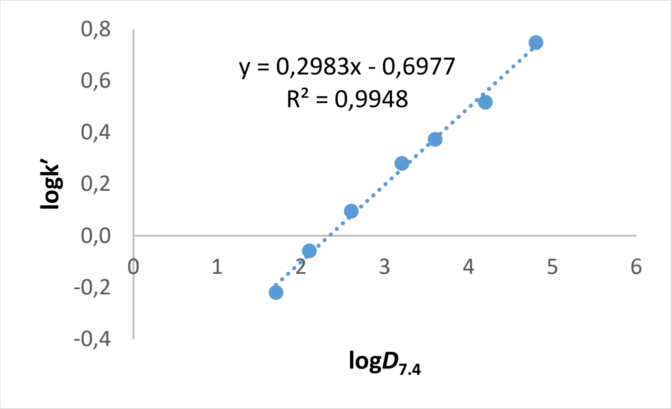

Figure S107: Calibration function C.

Calibration D

Table S4: Values used for Calibration D.

Reference	t_R mean [min]	k'	logk'	log*D* (Lit.)
Uracil	1.885			
Acetophenone	2.965	0.603	-0.220	1.7
Benzene	3.471	0.876	-0.057	2.1
Ethyl benzoate	4.155	1.246	0.095	2.6
Benzophenone	5.371	1.903	0.279	3.2
Phenyl benzoate	6.219	2.362	0.373	3.6
Diphenyl ether	7.928	3.285	0.517	4.2
Bibenzyl	12.186	5.587	0.747	4.8

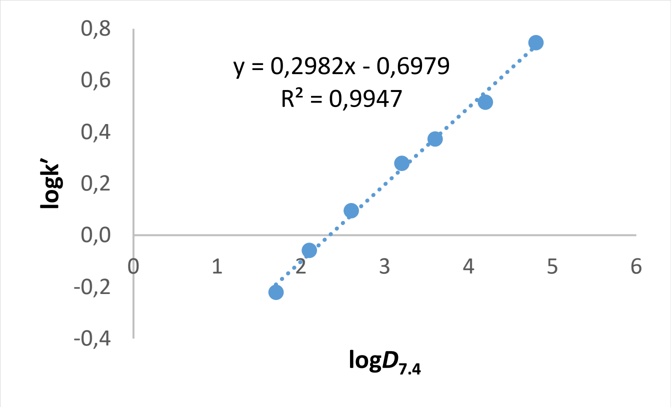

Figure S108: Calibration function D.

Log$D_{7.4}$ values

Table S5: Measured retention times and calculated log$D_{7.4}$ values.

Cmpd.	t_R [min]	k'	logk'	cali	log$D_{7.4}$	log$D_{7.4}$ (mean)
**1**	3.351	0.859	-0.066	A1	2.099	2.1
	3.242	0.798	-0.098	B1	2.064	
**2**	3.369	0.821	-0.086	C	2.052	2.1
	3.372	0.823	-0.085	D	2.056	
**17**	4.298	1.323	0.122	C	2.747	2.7
	4.297	1.323	0.121	D	2.748	
**21**	4.453	1.470	0.167	A1	2.882	2.9
	4.266	1.366	0.135	B1	2.860	
**28a**	6.880	2.719	0.434	C	3.795	3.8
	6.864	2.710	0.433	D	3.792	
**28b**	8.878	3.799	0.580	C	4.282	4.3
	8.884	3.802	0.580	D	4.285	
**36a**	6.444	2.574	0.411	A1	3.697	3.7
	6.020	2.339	0.369	B1	3.656	
**36b**	8.067	3.474	0.541	A1	4.134	4.1
	7.461	3.138	0.497	B1	4.092	
**47**	5.284	1.856	0.269	C	3.239	3.2
	5.269	1.848	0.267	D	3.235	
**55a**	5.720	2.172	0.337	A1	3.450	3.5
	5.486	2.043	0.310	B1	3.456	
**55b**	5.927	2.287	0.359	A1	3.525	3.5
	5.666	2.143	0.331	B1	3.527	
**61**	3.941	1.186	0.074	A1	2.569	2.6
	3.834	1.126	0.052	B1	2.575	
**66**	12.640	6.011	0.779	A2	4.753	4.8
	11.764	5.525	0.742	B2	4.752	

4 Analytical discrimination of regioisomers 57 and 58

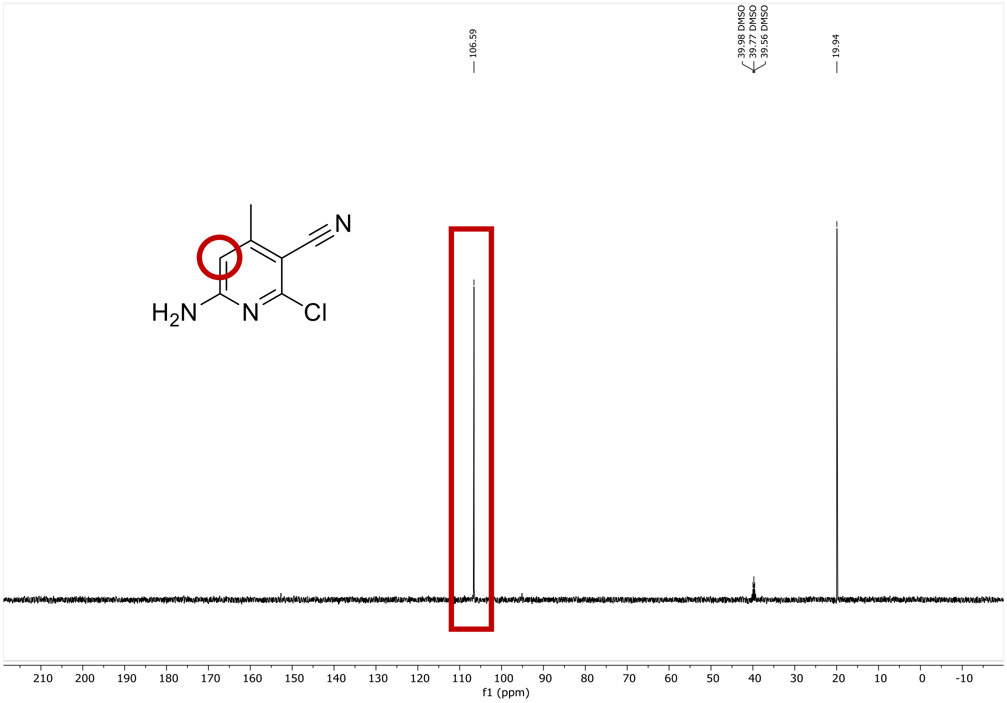

Figure S109: DEPT135 spectrum of compound **57** with highlighted signal of the pyridine CH carbon atom.

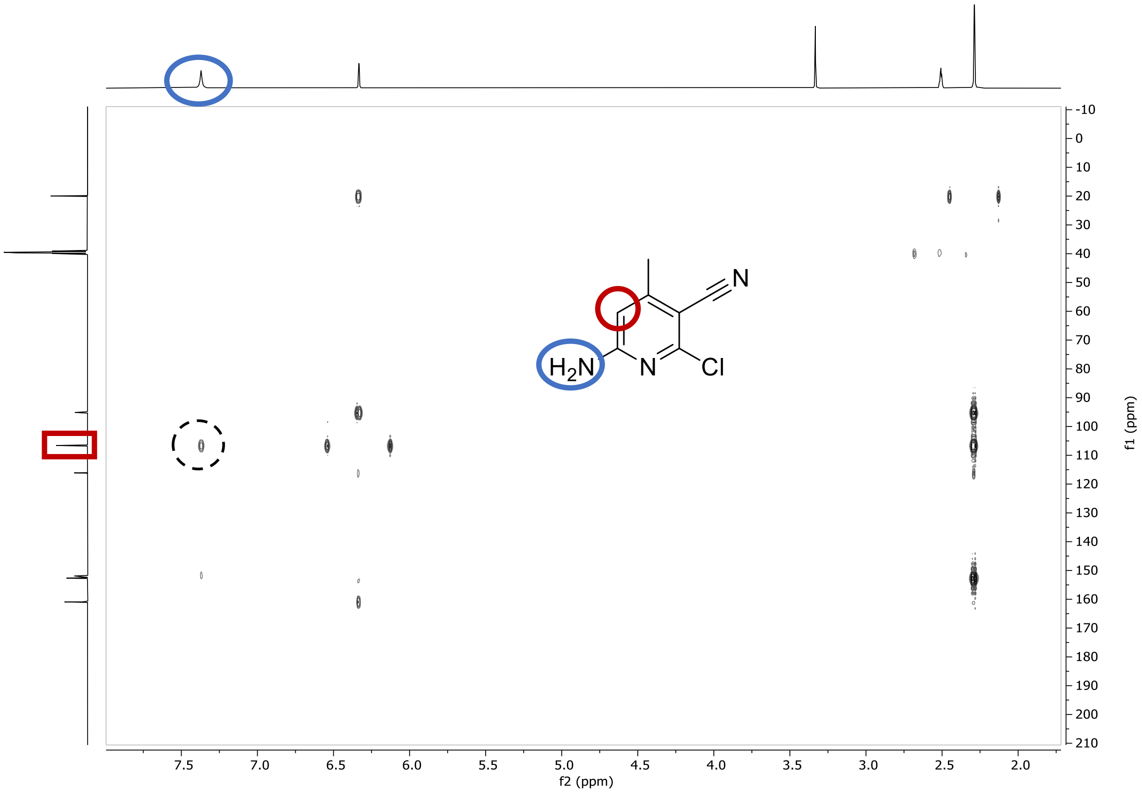

Figure S110: HMBC spectrum of compound **57** with highlighted correlation between pyridine CH carbon atom (red) and NH_2 protons (blue).

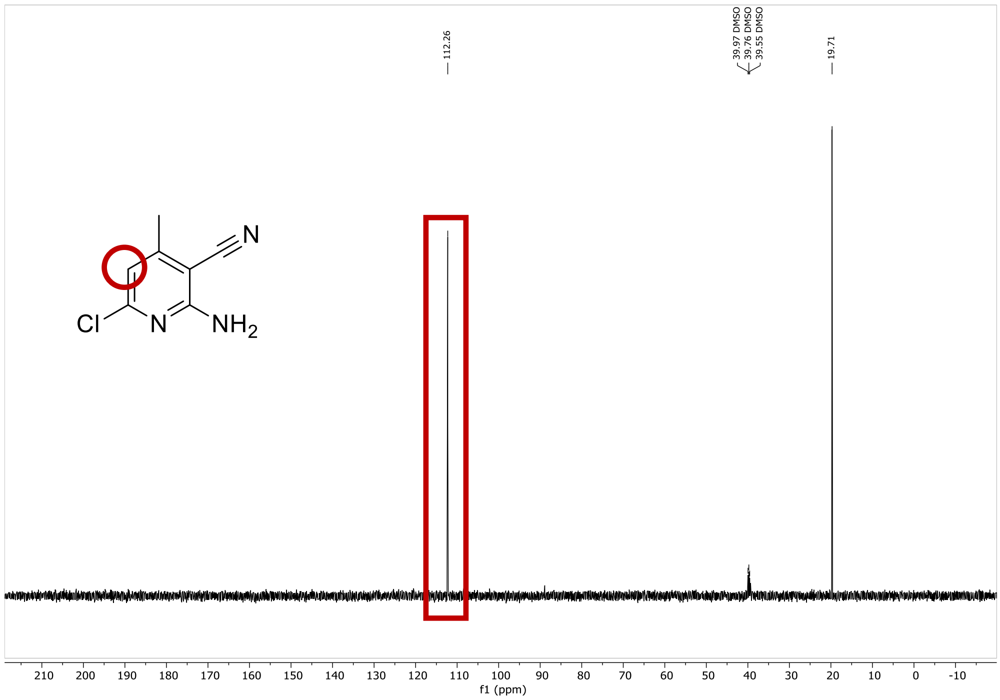

Figure S111: DEPT135 spectrum of compound **58** with highlighted signal of the pyridine CH carbon atom.

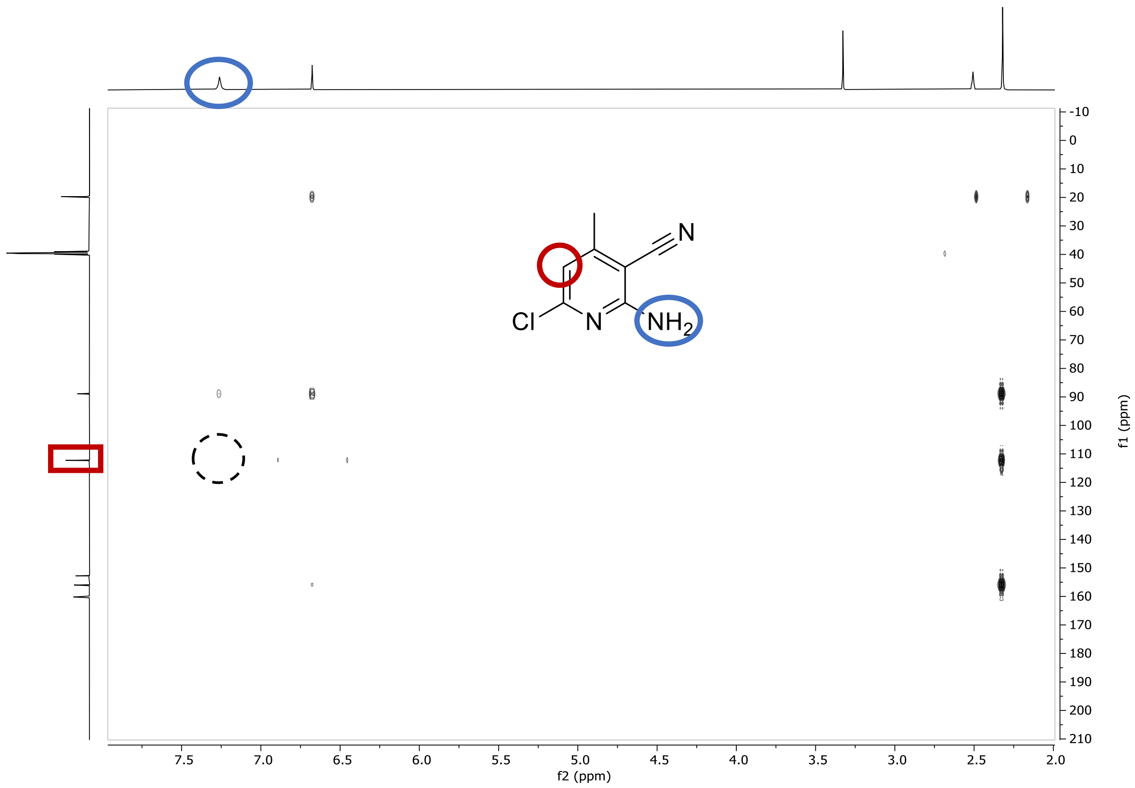

Figure S112: HMBC spectrum of compound **58** lacking the correlation between pyridine CH carbon atom (red) and NH_2 protons (blue).
